# Supplementary material for: Perspectives and experiences of researchers regarding feedback of incidental genomic research findings: A qualitative study
Source: PLoS One. 2022 Aug 29;17(8):e0273657. doi: 10.1371/journal.pone.0273657 (PMC9423610; doi:10.1371/journal.pone.0273657)
Supplement: S1 File — (DOCX) [file pone.0273657.s001.docx]

|  | | | | | | | | | | | | | | | | | |
| --- | --- | --- | --- | --- | --- | --- | --- | --- | --- | --- | --- | --- | --- | --- | --- | --- | --- |
| Coding Summary By Code | | | | | | | | | | | | | | | | | |
| Researchers GGR return of results and recommendations | | | | | | | | | | | | | | | | | |
|  | | | | | | | | | | | | | | | | | |
|  | | | **Aggregate** |  | **Classification** |  | **Coverage** |  | **Number Of Coding References** | |  | **Reference Number** |  | **Coded By Initials** |  | **Modified On** |  |
| **Node** | | | | | | | | | | | | | | | | |  |
|  | **Nodes\\Return of results\\Conditions for return of results** | | | | | | | | | | | | | | | |  |
|  | | **Document** | | | | | | | | | | | | | | |  |
|  | | | **Files\\Female\\R-003.female** | | | | | | | | | | | | | |  |
|  |  |  | Yes |  |  |  |  |  |  | |  | | | | | |  |
|  | | |  |  |  |  |  |  |  | |  | | | | | | |
|  | | | | | | | | | | | |  |  |  |  |  |  |
|  | RESPONDENT: one if it is going to improve their well being like in terms of care if it is clinical care, maybe even for counselling purposes of future relationships but I also know well maybe because here not so many people really reach out for insurance apart from affluent people the way to go for people. But if this information which is generated from a genetics or genomics study is ever to be known by insurance groups and what some times it raises the premium so it is sometimes a disadvantage the individuals themselves but still for me, I think people need to know, When I say that people need to know I am talking about the individuals the participants not the community like not saying that, that home or that village, that one I don’t accept it but like the individuals its their right to know | | | | | | | | | | | | | | | |  |
|  |  | | | | | | | | | | | | | | | |  |
|  | | | | | | | | | | | |  |  |  |  |  |  |
|  | INTERVIEWER: so, that is individual participants getting their results, how about aggregate results where by you don’t give individuals or you can’t give individuals individually but then there is this fact that you’re supposed to give back to research community doesn’t have to be that geographical?  RESPONDENT: as now no, even if it is anonymous now like if am just trying to think aloud if there was a genomics study of members of staff in the faculty of medicine at a university, the only good thing here is that we come from different backgrounds but lets say maybe the residents or the people who were born in this or that even if the data is aggreviated I don’t think I will give it out | | | | | | | | | | | | | | | |  |
|  |  | | | | | | | | | | | | | | | |  |
|  | | | **Files\\Female\\R026 female** | | | | | | | | | | | | | |  |
|  |  |  | Yes |  |  |  |  |  |  | |  | | | | | |  |
|  | | |  |  |  |  |  |  |  | |  | | | | | | |
|  | | | | | | | | | | | |  |  |  |  |  |  |
|  | INTERVIEWER: But you think at a particular stage when you are sure you can communicate the findings  RESPONDENT: Like all research, when you think it is of use, of interest to outcome of people, it is important to translate that. It is like vein tracking a drug you can do it in monkeys, then you can do a phase one study, you cant tell people we did this, then you can do a phase two study, then you can say we did this study over the last 10 years, now this drug works and we now want to study using it, but giving them the earlier work, its good so that they have an idea, it is important for people to have an idea why you did things | | | | | | | | | | | | | | | |  |
|  |  | | | | | | | | | | | | | | | |  |
|  | | | | | | | | | | | |  |  |  |  |  |  |
|  | INTERVIEWER: But you think at a particular stage when you are sure you can communicate the findings  RESPONDENT: Like all research, when you think it is of use of interest to outcome of people, it is important to translate that. It is like vein tracking a drug you can do it in monkeys, then you can do a phase one study, you cant tell people we did this, then you can do a phase two study, then you can say we did this study over the last 10 years, now this drug works and we now want to study using it, but giving them the earlier work, its good so that they have an idea, it is important for people to have an idea why you did things | | | | | | | | | | | | | | | |  |
|  |  | | | | | | | | | | | | | | | |  |
| Reports\\Coding Summary By Code Report | | | | | | | | | | Page 1 of 109 | | | | | | | |
|  | | | | | | | | | | | | | | | | | |
|  | | | **Aggregate** |  | **Classification** |  | **Coverage** |  | **Number Of Coding References** | |  | **Reference Number** |  | **Coded By Initials** |  | **Modified On** |  |
|  | | | | | | | | | | | | | | | | | |
|  | | | | | | | | | | | |  |  |  |  |  |  |
|  | INTERVIEWER: Okay so those are individual results, how about aggregate results?  RESPONDENT: That can even be more confusing, because the community won’t be community as in region A Versus region B in the country, its in Uganda we found that this gene is related to TB and in the US its not, so it doesn’t mean much again | | | | | | | | | | | | | | | |  |
|  |  | | | | | | | | | | | | | | | |  |
|  | | | **Files\\Female\\R027.female** | | | | | | | | | | | | | |  |
|  |  |  | Yes |  |  |  |  |  |  | |  | | | | | |  |
|  | | |  |  |  |  |  |  |  | |  | | | | | | |
|  | | | | | | | | | | | |  |  |  |  |  |  |
|  | INTERVIEWER: Do you feel any one can do that or there should be some group of people?  RESPONDENT: Meanwhile you can do that if there is a research and genotype you can simplify your language for a P7 child to understand. | | | | | | | | | | | | | | | |  |
|  |  | | | | | | | | | | | | | | | |  |
|  | | | | | | | | | | | |  |  |  |  |  |  |
|  | RESPONDENT: Well, there are some places where it might be a bit sensitive to, for example if you are dealing with a genotype in that if you tell someone for example that you have a certain disease that can affect your children, it is important to tell them before reproducing or marrying. Like if you are a sickle cell carrier or something and you now know so you tell them or if you can tell those participants that are sensitive that you have a determinant that you know, looking at the gene, it is possible that you are not related. So, there are somethings that will impact the patient directly. | | | | | | | | | | | | | | | |  |
|  |  | | | | | | | | | | | | | | | |  |
|  | | | | | | | | | | | |  |  |  |  |  |  |
|  | INTERVIEWER: So you distract something on return of individual results and then may be agree on results that pertain public members and their relatives but there are these aggregate results that inform bigger communities that may be the population?  RESPONDENT: But then you are not telling the individual only, you are entitled to tell bodies like if it is ministry of health and you tell them that you know what; in Buruli a hundred people have this genotype, there is a problem there or something or if they are there you go to higher bodies then the individual yes might be there but you might also need to inform the high bodies. Just like even sickle cells, if they need to know that in this area there is a lot of sickle cell so that they can tailor intervention | | | | | | | | | | | | | | | |  |
|  |  | | | | | | | | | | | | | | | |  |
|  | | | | | | | | | | | |  |  |  |  |  |  |
|  | INTERVIEWER: That is return of results  RESPONDENT: Do you want to know or you don’t want to know, do you understand the implications of these results? | | | | | | | | | | | | | | | |  |
|  |  | | | | | | | | | | | | | | | |  |
|  | | | | | | | | | | | |  |  |  |  |  |  |
|  | INTERVIEWER: I think that is like we cant just come in to a community, do your research without consulting the elders however, should we have like the way I have given out that consent to sign that I agree to participate and I will get this compensation and I have a right to know my results, to withdraw and all that, should we think that a community can at a community level? I can have a few participants but I will know about the whole village so do you think we still need the village to consent or we shall need them to know information about the study?  RESPONDENT: they will need to know because in the village you might have a hundred people but twenty might say yes and others say no, so let’s go to the leader and say yes then we continue with results and say yes if we have a community leadership so whenever you go to your community and say in a meeting, how many will agree? that’s a no. | | | | | | | | | | | | | | | |  |
|  |  |  |  |  |  |  |  |  |  |  |  |  |  |  |  |  |  |
|  |  | | | | | | | | | | | | | | | |  |
|  | | | | | | | | | | | |  |  |  |  |  |  |
|  | INTERVIEWER: I have ever worked with someone and they were doing a study on dead bodies actually the brain of dead bodies so there was this community accepting the family members consented to get a sample from the dead body but like a year in to the study some of them withdrew from the study so I don’t know whether someone came and informed the whole community about any repacations and what so they all pulled out of the study, so what about that case?  RESPONDENT: Really, they accepted as individual families or the leader of the family at least had agreed. That is just a representative of the family but not individual. I think its like when you an experimental drug I think and you have cancer like what they do in the USA because there is a lot of cancer there are always experimental drugs so they tell you they want this drug and then may be you go and discuss with your family like should I or should I not but at the end, it is one person who will sign off and some will not but the end result is that one person signs so I think that is still an individual consent signed. Even for post mortem studies, those corpses, it is the next of kin or one team member who will sign so I don’t know whether it is still community as we discussed but if its him as a signing person and the next of kin says no, it doesn’t involve all the siblings saying yes that he is the one who is legal still consent withdrawing an individual. So, it doesn’t represent the community because I am sure there are some people who are in that family who didn’t want that thing. | | | | | | | | | | | | | | | |  |
|  |  |  |  |  |  |  |  |  |  |  |  |  |  |  |  |  |  |
|  |  | | | | | | | | | | | | | | | |  |
|  | | | | | | | | | | | | | | | | | |
| Reports\\Coding Summary By Code Report | | | | | | | | | | Page 2 of 109 | | | | | | | |
|  | | | | | | | | | | | | | | | | | |
|  | | | **Aggregate** |  | **Classification** |  | **Coverage** |  | **Number Of Coding References** | |  | **Reference Number** |  | **Coded By Initials** |  | **Modified On** |  |
|  | | | **Files\\Female\\R029 female** | | | | | | | | | | | | | |  |
|  |  |  | Yes |  |  |  |  |  |  | |  | | | | | |  |
|  | | |  |  |  |  |  |  |  | |  | | | | | | |
|  | | | | | | | | | | | |  |  |  |  |  |  |
|  | INTERVIEWER: But for example, if genetic results were of clinical relevance and you had to return results to individual participants, under what conditions do you think you would return these results to them?  RESPONDENT:.. Under what conditions, I think if they were of clinical relevance, first of all am not a doctor, but if they were, I would consult the doctor who was attending to this particular person, and maybe we could discuss the results with them but of course communicating this information to the participants and most of them are not highly educated. It’s also another thing to break down for the results to make sense to them. So yes what I would do is to consult the attending doctor and we see how to approach this patient, even involving a genetic councilor who can to talk as well as they understand these results with them. And of course, communicating this genetic work to them yes | | | | | | | | | | | | | | | |  |
|  |  |  |  |  |  |  |  |  |  |  |  |  |  |  |  |  |  |
|  |  | | | | | | | | | | | | | | | |  |
|  | | | | | | | | | | | |  |  |  |  |  |  |
|  | INTERVIEWER: what about aggregate results ?  RESPONDENT: Aggregate results?  INTERVIEWER: If you are to return aggregate results. Aggregate results are not direct to individuals but like a group of people like a community yaa aggregate.  RESPONDENT: Then that is good like if the study participants participated in the study, they gave an informed consent after explaining to them what we are going to do during collection of samples, I think it’s good to explain to them what you eventually found if it is not and individual and you have an opportunity to gather these people and you are able to explain from the point of view, it would be a good thing. | | | | | | | | | | | | | | | |  |
|  |  |  |  |  |  |  |  |  |  |  |  |  |  |  |  |  |  |
|  |  | | | | | | | | | | | | | | | |  |
|  | | | | | | | | | | | |  |  |  |  |  |  |
|  | INTERVIEWER: In your view as the researcher  RESPONDENT: in my view as a researcher?  INTERVIEWER: would you return or communicate to the participants?  RESPONDENT: I think I would, yah if the conditions allow I think I would, because if their life will depend on that, or they are likely to improve their quality of life. I think its humane to communicate and if they can get treatment | | | | | | | | | | | | | | | |  |
|  |  | | | | | | | | | | | | | | | |  |
|  | | | | | | | | | | | |  |  |  |  |  |  |
|  | INTERVIEWER: In another example such a thing may not be clinically relevant to the patients. As you are doing your work you realize you are looking out for genes for pre-eclampsia?  RESPONDENT: Yes pre-eclampsia  INTERVIEWER: But then you are doing what you are doing, you realize that a certain gene this individual has, that gene may be makes this individual susceptible to breast cancer or susceptible to something dangerous condition that could impact her life or the life of her family, but remember this person consented to what the study required, he dint consent to other findings and this person is susceptible to this gene and if found it how to communicate to them may I would get it. So what would you recommend in such scenario? You found it but it was not your primary intended finding.  RESPONDENT: In your scenario may be included it in the consent form,  INTERVIEWER: you had not included it in the consent form but you feel this person if not communicated to, he/she may die in the next year, or something like that or something bad might happen because it is something that might affect her life. These are risk factors.  RESPONDENT: I again being humane, I think I would report such a finding but the way handle such also matters. I think you need a counsellor to explain if you are not a counselor yourself. Maybe you need a genetic counsellor or a counsellor who can break it down to the patient. Again it maybe an advantage to the patient that they have found such results to prevent occurrence of such diseases. | | | | | | | | | | | | | | | |  |
|  |  |  |  |  |  |  |  |  |  |  |  |  |  |  |  |  |  |
|  |  |  |  |  |  |  |  |  |  |  |  |  |  |  |  |  |  |
|  |  | | | | | | | | | | | | | | | |  |
|  | | | | | | | | | | | |  |  |  |  |  |  |
|  | INTERVIEWER: so you can only disclose that information if they had consented to receiving it.  RESPONDENT: yaaaa if they had consented for it, but if they don’t consent then I think you don’t have to. | | | | | | | | | | | | | | | |  |
|  |  | | | | | | | | | | | | | | | |  |
|  | | | **Files\\Female\\R030.female** | | | | | | | | | | | | | |  |
|  |  |  | Yes |  |  |  |  |  |  | |  | | | | | |  |
|  | | |  |  |  |  |  |  |  | |  | | | | | | |
|  | | | | | | | | | | | |  |  |  |  |  |  |
|  | Qn.3 INTERVIEWER: Is any of your studies of such a nature that you have to report back to participants about what you discovered about their genetic makeup?  RESPONDENT: So, right now we in our consent forms we ask if they would want. But majority of them say that you see it is not going to help me, am here only looking at those we can help in the future but there is an option, they have an option if they want to know so it’s an informed decision that they as participants take. They say I am what I am but what I want to see is can I help other people? | | | | | | | | | | | | | | | |  |
|  |  | | | | | | | | | | | | | | | |  |
| Reports\\Coding Summary By Code Report | | | | | | | | | | Page 3 of 109 | | | | | | | |
|  | | | | | | | | | | | | | | | | | |
|  | | | **Aggregate** |  | **Classification** |  | **Coverage** |  | **Number Of Coding References** | |  | **Reference Number** |  | **Coded By Initials** |  | **Modified On** |  |
|  | | | | | | | | | | | | | | | | | |
|  | | | | | | | | | | | |  |  |  |  |  |  |
|  | INTERVIEWER: Do you have like procedures in place that your research team is supposed to follow in case they are giving back those genetic results?  RESPONDENT: We do have procedures SOPs on how to give results to clients whether they are genetics or HIV because we have specific counsellors that are designated for specific results so that they know how to live with each study. | | | | | | | | | | | | | | | |  |
|  |  | | | | | | | | | | | | | | | |  |
|  | | | | | | | | | | | |  |  |  |  |  |  |
|  | RESPONDENT: So far we have not done that, we have not had something that we needed but we able to do that because you still remember the participant has an informed consent on what you have agreed on so the process of information even if you found something that is detrimental you would have to ask to expedited to consult the IRB on how you handle that case.  INTERVIEWER: Are there any other conditions which you think are important to return results with these incidental findings much as they are not our primary objective but you found, under what condition would you give them?  RESPONDENT: If they are going to improve the patient outcome, it is because at the end of the day, patient’s state is the most important thing in the research. So, if you find something that you think will help the patient improve patient outcome it is important that to that IRB is communicated to the patient. | | | | | | | | | | | | | | | |  |
|  |  |  |  |  |  |  |  |  |  |  |  |  |  |  |  |  |  |
|  |  | | | | | | | | | | | | | | | |  |
|  | | | **Files\\Male\\R-001 male** | | | | | | | | | | | | | |  |
|  |  |  | Yes |  |  |  |  |  |  | |  | | | | | |  |
|  | | |  |  |  |  |  |  |  | |  | | | | | | |
|  | | | | | | | | | | | |  |  |  |  |  |  |
|  | I would return those results to Patient if i knew those results were going to help a patient to be treated well, or if i knew they were going to affect his Prognosis. If a Patient is not going to do well, it’s good to tell them that because of "A, B, and C", you are likely not to do very well in this state. | | | | | | | | | | | | | | | |  |
|  |  | | | | | | | | | | | | | | | |  |
|  | | | | | | | | | | | |  |  |  |  |  |  |
|  | So you return when they are going to be beneficial or when they are not going to do well?  RESPONDENT:  Still, they are beneficial. If you know that this person's genotype is usually affected negatively by this organism then you prepare the people. you say we are doing all we can, but from our results we see this might not be very helpful. We have seen a lot of people that have gone to India and come back to Die, simply because the people didn't tell them the truth, because why should you spend ugx.100 million, go to India and on your return, die two days later. Its basically false hope. i have a friend, she was our teacher here, she had a cancer and went to India but died two days after her return. so that's all money she wasted which would have been beneficial to the people she left behind. so, i still say, if its going to be beneficial to the person to manage the treatment then you tell them. But if it's also not really, we can't do much, then we should be able to tell. its hard but its some times better so that they have an informed decision to take, instead of saying go to India and do this surgery, and on returning you loose all hope. | | | | | | | | | | | | | | | |  |
|  |  |  |  |  |  |  |  |  |  |  |  |  |  |  |  |  |  |
|  |  | | | | | | | | | | | | | | | |  |
|  | | | | | | | | | | | |  |  |  |  |  |  |
|  | if you are to release these aggregated results, it has to be a comprehensive package, if you have this, then you are most likely to have this, but there is also this. its like a combination, most of this information comes through with cancer. we know now that if we do the genetics, there people who are predisposed to cancer because of genetics, but cancer doesn't just develop because you have a gene, there other factors around that gene that make you to suffer. so people have gone ahead to cut off their breasts because they have this gene, there people who have gone ahead and removed the uterus because they have some gene for cervical cancer. although that is good, sometimes, it might not be 100% true because there are also other factors that come into play to give you cancer. | | | | | | | | | | | | | | | |  |
|  |  | | | | | | | | | | | | | | | |  |
|  | | | **Files\\Male\\R-002. male** | | | | | | | | | | | | | |  |
|  |  |  | Yes |  |  |  |  |  |  | |  | | | | | |  |
|  | | |  |  |  |  |  |  |  | |  | | | | | | |
|  | | | | | | | | | | | |  |  |  |  |  |  |
|  | Respondent: I guess, I kind of generalize already but I think that other institutions cultural institutions and all of that would be very appropriate and important to certain aspects of the findings | | | | | | | | | | | | | | | |  |
|  |  | | | | | | | | | | | | | | | |  |
|  | | | | | | | | | | | | | | | | | |
| Reports\\Coding Summary By Code Report | | | | | | | | | | Page 4 of 109 | | | | | | | |
|  | | | | | | | | | | | | | | | | | |
|  | | | **Aggregate** |  | **Classification** |  | **Coverage** |  | **Number Of Coding References** | |  | **Reference Number** |  | **Coded By Initials** |  | **Modified On** |  |
|  | | | | | | | | | | | | | | | | | |
|  | | | | | | | | | | | | 2 |  | DES |  | 1/13/2020 8:37 AM |  |
|  | Respondent: I think as researchers it would be very important for us to, first of all look at the language you’re going to use as of the research findings, two, the members of the community in such a way that we should be able to break it down to a level that can be understood properly so the tools you would use would be if it is a meeting then we would just go and have like a presentation and then also we could use the print media especially the ones in the local language. I know that it becomes more complicated to translate to local languages and all that but we also have the ready that would also be ways. I know that a lot of the people deep down there were the research happens, they listen to radios quite a lot so there will be a deliberate program to leave some of these messages. A well-organized program so that people know that on such and such a day at this time, they are going to talk about our community or at least things that concern us yes so that would be one way in a very simplified language | | | | | | | | | | | | | | | |  |
|  |  |  |  |  |  |  |  |  |  |  |  |  |  |  |  |  |  |
|  |  | | | | | | | | | | | | | | | |  |
|  | | | **Files\\Male\\R-004.male** | | | | | | | | | | | | | |  |
|  |  |  | Yes |  |  |  |  |  |  | |  | | | | | |  |
|  | | |  |  |  |  |  |  |  | |  | | | | | | |
|  | | | | | | | | | | | |  |  |  |  |  |  |
|  | Respondent: if it is a family and the individual is a minor there, I would of course inform the parents but if it is an adult in a family still primarily, we inform the adult and if the adult wants to communicate it’s up to them | | | | | | | | | | | | | | | |  |
|  |  | | | | | | | | | | | | | | | |  |
|  | | | **Files\\Male\\R-017. male** | | | | | | | | | | | | | |  |
|  |  |  | Yes |  |  |  |  |  |  | |  | | | | | |  |
|  | | |  |  |  |  |  |  |  | |  | | | | | | |
|  | | | | | | | | | | | |  |  |  |  |  |  |
|  | Respondent: Like I said there are protocols, I will have to go through hospital management, give them the results because they are the ones who even allow us to take the samples from their facility, so I give information back to the management through the doctors, nurses, counselors, relay that information back to the patients. And maybe one thing I have to make clear is maybe as a researcher, there are avenues through which I can relay information to the actual individual who is affected but I can’t directly interface with that individual | | | | | | | | | | | | | | | |  |
|  |  | | | | | | | | | | | | | | | |  |
|  | **Nodes\\Return of results\\Conditions for return of results\Aggregate** | | | | | | | | | | | | | | | |  |
|  | | **Document** | | | | | | | | | | | | | | |  |
|  | | | **Files\\Female\\R-003.female** | | | | | | | | | | | | | |  |
|  |  |  | Yes |  |  |  |  |  |  | |  | | | | | |  |
|  | | |  |  |  |  |  |  |  | |  | | | | | | |
|  | | | | | | | | | | | |  |  |  |  |  |  |
|  | INTERVIEWER: so, that is individual participants getting their results, how about aggregate results where by you don’t give individuals or you can’t give individuals individually but then there is this fact that you’re supposed to give back to research community doesn’t have to be that geographical?  RESPONDENT: as now no, even if it is anonymous now like if am just trying to think aloud if there was a genomics study of members of staff in the faculty of medicine at a university the only good thing here is that we come from different backgrounds but lets say maybe the residents or the people who were born in this or that even if the data is aggreviated I don’t think I will give it out | | | | | | | | | | | | | | | |  |
|  |  | | | | | | | | | | | | | | | |  |
|  | | | | | | | | | | | | | | | | | |
| Reports\\Coding Summary By Code Report | | | | | | | | | | Page 5 of 109 | | | | | | | |
|  | | | | | | | | | | | | | | | | | |
|  | | | **Aggregate** |  | **Classification** |  | **Coverage** |  | **Number Of Coding References** | |  | **Reference Number** |  | **Coded By Initials** |  | **Modified On** |  |
|  | | | **Files\\Female\\R026 female** | | | | | | | | | | | | | |  |
|  |  |  | Yes |  |  |  |  |  |  | |  | | | | | |  |
|  | | |  |  |  |  |  |  |  | |  | | | | | | |
|  | | | | | | | | | | | |  |  |  |  |  |  |
|  | INTERVIEWER: But you think at a particular stage when you are sure you can communicate the findings  RESPONDENT: Like all research, when you think it is of use of interest to outcome of people, it is important to translate that. It is like vein tracking a drug you can do it in monkeys, then you can do a phase one study, you cant tell people we did this, then you can do a phase two study, then you can say we did this study over the last 10 years, now this drug works and we now want to study using it, but giving them the earlier work, its good so that they have an idea, it is important for people to have an idea why you did things | | | | | | | | | | | | | | | |  |
|  |  | | | | | | | | | | | | | | | |  |
|  | | | | | | | | | | | |  |  |  |  |  |  |
|  | INTERVIEWER: Okay so those are individual results, how about aggregate results?  RESPONDENT: That can even be more confusing, because the community won’t be community as in region A Versus region B in the country, its in Uganda we found that this gene is related to TB and in the US its not, so it doesn’t mean much again | | | | | | | | | | | | | | | |  |
|  |  | | | | | | | | | | | | | | | |  |
|  | | | **Files\\Female\\R027.female** | | | | | | | | | | | | | |  |
|  |  |  | Yes |  |  |  |  |  |  | |  | | | | | |  |
|  | | |  |  |  |  |  |  |  | |  | | | | | | |
|  | | | | | | | | | | | |  |  |  |  |  |  |
|  | INTERVIEWER: Do you feel any one can do that or there should be some group of people?  RESPONDENT: Meanwhile you can do that if there is a research and genotype you can simplify your language for a P7 child to understand. | | | | | | | | | | | | | | | |  |
|  |  | | | | | | | | | | | | | | | |  |
|  | | | | | | | | | | | |  |  |  |  |  |  |
|  | INTERVIEWER: So you distract something on return of individual results and then may be agree on results that pertain public members and their relatives but there are these aggregate results that inform bigger communities that may be the population?  RESPONDENT: But then you are not telling the individual only, you are entitled to tell bodies like if it is ministry of health and you tell them that you know what; in Buruli a hundred people have this genotype, there is a problem there or something or if they are there you go to higher bodies then the individual yes might be there but you might also need to inform the high bodies. Just like even sickle cells, if they need to know that in this area there is a lot of sickle cell so that they can tailor intervention | | | | | | | | | | | | | | | |  |
|  |  | | | | | | | | | | | | | | | |  |
|  | | | | | | | | | | | |  |  |  |  |  |  |
|  | INTERVIEWER: I think that is like we cant just come in to a community, do your research without consulting the elders however, should we have like the way I have given out that consent to sign that I agree to participate and I will get this compensation and I have a right to know my results to withdraw and all that, should we think that a community can at a community level? I can have a few participants but I will know about the whole village so do you think we still need the village to consent or we shall need them to know information about the study?  RESPONDENT: they will need to know because in the village you might have a hundred people but twenty might say yes and others say no so lets go to the leader and say yes then we continue with results and say yes if we have a community leadership so whenever you go to your community and say in a meeting, how many will agree? that’s a no. | | | | | | | | | | | | | | | |  |
|  |  |  |  |  |  |  |  |  |  |  |  |  |  |  |  |  |  |
|  |  | | | | | | | | | | | | | | | |  |
|  | | | | | | | | | | | |  |  |  |  |  |  |
|  | INTERVIEWER: I have ever worked with someone and they were doing a study on dead bodies actually the brain of dead bodies so there was this community accepting the family members consented to get a sample from the dead body but like a year in to the study some of them withdrew from the study so I don’t know whether someone came and informed the whole community about any repacations and what so they all pulled out of the study, so what about that case?  RESPONDENT: Really, they accepted as individual families or the leader of the family at least had agreed. That is just a representative of the family but not individual. I think its like when you an experimental drug I think and you have cancer like what they do in the USA because there is a lot of cancer there are always experimental drugs so they tell you they want this drug and then may be you go and discuss with your family like should I or should I not but at the end, it is one person who will sign off and some will not but the end result is that one person signs so I think that is still an individual consent signed. Even for post mortem studies, those corpses, it is the next of kin or one team member who will sign so I don’t know whether it is still community as we discussed but if its him as a signing person and the next of kin says no, it doesn’t involve all the siblings saying yes that he is the one who is legal still consent withdrawing an individual. So, it doesn’t represent the community because I am sure there are some people who are in that family who didn’t want that thing. | | | | | | | | | | | | | | | |  |
|  |  |  |  |  |  |  |  |  |  |  |  |  |  |  |  |  |  |
|  |  | | | | | | | | | | | | | | | |  |
|  | | | | | | | | | | | | | | | | | |
| Reports\\Coding Summary By Code Report | | | | | | | | | | Page 6 of 109 | | | | | | | |
|  | | | | | | | | | | | | | | | | | |
|  | | | **Aggregate** |  | **Classification** |  | **Coverage** |  | **Number Of Coding References** | |  | **Reference Number** |  | **Coded By Initials** |  | **Modified On** |  |
|  | | | **Files\\Female\\R029 female** | | | | | | | | | | | | | |  |
|  |  |  | Yes |  |  |  |  |  |  | |  | | | | | |  |
|  | | |  |  |  |  |  |  |  | |  | | | | | | |
|  | | | | | | | | | | | |  |  |  |  |  |  |
|  | INTERVIEWER: what about aggregate results ?  RESPONDENT: Aggregate results?  INTERVIEWER: If you are to return aggregate results. Aggregate results are not direct to individuals but like a group of people like a community yaa aggregate.  RESPONDENT: Then that is good like if the study participants participated in the study, they gave an informed consent after explaining to them what we are going to do during collection of samples, I think it’s good to explain to them what you eventually found if it is not and individual and you have an opportunity to gather these people and you are able to explain from the point of view, it would be a good thing. | | | | | | | | | | | | | | | |  |
|  |  |  |  |  |  |  |  |  |  |  |  |  |  |  |  |  |  |
|  |  | | | | | | | | | | | | | | | |  |
|  | | | **Files\\Male\\R-001 male** | | | | | | | | | | | | | |  |
|  |  |  | Yes |  |  |  |  |  |  | |  | | | | | |  |
|  | | |  |  |  |  |  |  |  | |  | | | | | | |
|  | | | | | | | | | | | |  |  |  |  |  |  |
|  | if you are to release these aggregated results, it has to be a comprehensive package, if you have this, then you are most likely to have this, but there is also this. its like a combination, most of this information comes through with cancer. we know now that if we do the genetics, there people who are predisposed to cancer because of genetics, but cancer doesn't just develop because you have a gene, there other factors around that gene that make you to suffer. so people have gone ahead to cut off their breasts because they have this gene, there people who have gone ahead and removed the uterus because they have some gene for cervical cancer. although that is good, sometimes, it might not be 100% true because there are also other factors that come into play to give you cancer. | | | | | | | | | | | | | | | |  |
|  |  | | | | | | | | | | | | | | | |  |
|  | | | **Files\\Male\\R-002. male** | | | | | | | | | | | | | |  |
|  |  |  | Yes |  |  |  |  |  |  | |  | | | | | |  |
|  | | |  |  |  |  |  |  |  | |  | | | | | | |
|  | | | | | | | | | | | |  |  |  |  |  |  |
|  | Respondent: I guess, I kind of generalize already but I think that other institutions cultural institutions and all of that would be very appropriate and important to certain aspects of the findings | | | | | | | | | | | | | | | |  |
|  |  | | | | | | | | | | | | | | | |  |
|  | | | | | | | | | | | |  |  |  |  |  |  |
|  | Respondent: I think as researchers it would be very important for us to, first of all look at the language you’re going to use as of the research findings, two, the members of the community in such a way that we should be able to break it down to a level that can be understood properly so the tools you would use would be if it is a meeting then we would just go and have like a presentation and then also we could use the print media especially the ones in the local language. I know that it becomes more complicated to translate to local languages and all that but we also have the ready that would also be ways. I know that a lot of the people deep down there were the research happens they listen to radios quite a lot so there will be a deliberate program to leave some of these messages a well-organized program so that people know that on such and such a day at this time, they are going to talk about our community or at least things that concern us yes so that would be one way in a very simplified language | | | | | | | | | | | | | | | |  |
|  |  |  |  |  |  |  |  |  |  |  |  |  |  |  |  |  |  |
|  |  | | | | | | | | | | | | | | | |  |
|  | | | **Files\\Male\\R-004.male** | | | | | | | | | | | | | |  |
|  |  |  | Yes |  |  |  |  |  |  | |  | | | | | |  |
|  | | |  |  |  |  |  |  |  | |  | | | | | | |
|  | | | | | | | | | | | |  |  |  |  |  |  |
|  | Respondent: if it is a family and the individual is a minor there, I would of course inform the parents but if it is an adult in a family still primarily, we inform the adult and if the adult wants to communicate it’s up to them | | | | | | | | | | | | | | | |  |
|  |  | | | | | | | | | | | | | | | |  |
| Reports\\Coding Summary By Code Report | | | | | | | | | | Page 7 of 109 | | | | | | | |
|  | | | | | | | | | | | | | | | | | |
|  | | | **Aggregate** |  | **Classification** |  | **Coverage** |  | **Number Of Coding References** | |  | **Reference Number** |  | **Coded By Initials** |  | **Modified On** |  |
|  | | | **Files\\Male\\R-017. male** | | | | | | | | | | | | | |  |
|  |  |  | Yes |  |  |  |  |  |  | |  | | | | | |  |
|  | | |  |  |  |  |  |  |  | |  | | | | | | |
|  | | | | | | | | | | | |  |  |  |  |  |  |
|  | Respondent: Like I said there are protocols, I will have to go through hospital management, give them the results because they are the ones who even allow us to take the samples from their facility, so I give information back to the management through the doctors, nurses, counselors, relay that information back to the patients. And maybe one thing I have to make clear is maybe as a researcher, there are avenues through which I can relay information to the actual individual who is affected but I can’t directly interface with that individual | | | | | | | | | | | | | | | |  |
|  |  | | | | | | | | | | | | | | | |  |
|  | **Nodes\\Return of results\\Conditions for return of results\individual** | | | | | | | | | | | | | | | |  |
|  | | **Document** | | | | | | | | | | | | | | |  |
|  | | | **Files\\Female\\R-003.female** | | | | | | | | | | | | | |  |
|  |  |  | Yes |  |  |  |  |  |  | |  | | | | | |  |
|  | | |  |  |  |  |  |  |  | |  | | | | | | |
|  | | | | | | | | | | | |  |  |  |  |  |  |
|  | RESPONDENT: one if it is going to improve their well being like in terms of care if it is clinical care, maybe even for counselling purposes of future relationships but I also know well maybe because here not so many people really reach out for insurance apart from affluent people the way to go people but if this information which is generated from a genetics or genomics study is ever to be known by insurance groups and what some times it raises the premium so it is sometimes a disadvantage the individuals themselves but still for me I think people need to know, when I say that people need to know I am talking about the individuals the participants not the community like not saying that , that home or that village, that one I don’t accept it but like the individuals its their right to know | | | | | | | | | | | | | | | |  |
|  |  | | | | | | | | | | | | | | | |  |
|  | | | **Files\\Female\\R026 female** | | | | | | | | | | | | | |  |
|  |  |  | Yes |  |  |  |  |  |  | |  | | | | | |  |
|  | | |  |  |  |  |  |  |  | |  | | | | | | |
|  | | | | | | | | | | | |  |  |  |  |  |  |
|  | INTERVIEWER: But you think at a particular stage when you are sure you can communicate the findings  RESPONDENT: Like all research, when you think it is of use of interest to outcome of people, it is important to translate that. It is like vein tracking a drug you can do it in monkeys, then you can do a phase one study, you cant tell people we did this, then you can do a phase two study, then you can say we did this study over the last 10 years, now this drug works and we now want to study using it, but giving them the earlier work, its good so that they have an idea, it is important for people to have an idea why you did things | | | | | | | | | | | | | | | |  |
|  |  | | | | | | | | | | | | | | | |  |
|  | | | **Files\\Female\\R027.female** | | | | | | | | | | | | | |  |
|  |  |  | Yes |  |  |  |  |  |  | |  | | | | | |  |
|  | | |  |  |  |  |  |  |  | |  | | | | | | |
|  | | | | | | | | | | | |  |  |  |  |  |  |
|  | RESPONDENT: Well, there are some places where it might be a bit sensitive to it for example if you are dealing with genotype in that if you tell someone for example that you have a certain disease that can affect your children, it is important to tell them before reproducing or marrying like if you are a sickle cell carrier or something and you now know so you tell them or if you can tell those participants that are sensitive that you have a determinant that you know, looking at the gene, it is possible that you are not related. So, there are somethings that will impact the patient directly. | | | | | | | | | | | | | | | |  |
|  |  | | | | | | | | | | | | | | | |  |
| Reports\\Coding Summary By Code Report | | | | | | | | | | Page 8 of 109 | | | | | | | |
|  | | | | | | | | | | | | | | | | | |
|  | | | **Aggregate** |  | **Classification** |  | **Coverage** |  | **Number Of Coding References** | |  | **Reference Number** |  | **Coded By Initials** |  | **Modified On** |  |
|  | | | | | | | | | | | | | | | | | |
|  | | | | | | | | | | | |  |  |  |  |  |  |
|  | INTERVIEWER: That is return of results  RESPONDENT: Do you want to know or you don’t want to know, do you understand the implications of these results? | | | | | | | | | | | | | | | |  |
|  |  | | | | | | | | | | | | | | | |  |
|  | | | **Files\\Female\\R029 female** | | | | | | | | | | | | | |  |
|  |  |  | Yes |  |  |  |  |  |  | |  | | | | | |  |
|  | | |  |  |  |  |  |  |  | |  | | | | | | |
|  | | | | | | | | | | | |  |  |  |  |  |  |
|  | INTERVIEWER: But for example if results were of clinical relevance and you had to return results to individual participants, under what conditions do you think you would return these results to them?  RESPONDENT:.. Under what conditions, I think if they were of clinical relevance, first of am not a doctor, but if they were, I would consult the doctor who was attending to this particular person. and may we could discuss the results with them but of course communicating this information to the participants and most of them are not highly educated. It’s also another thing to break down for the results to make sense to them. So yes what I would do is to consult the attending doctor and we see how to approach this patient, even involving a genetic councilor who can to talk as well as they understand these results with them. And of course, communicating this genetic work to them yes | | | | | | | | | | | | | | | |  |
|  |  |  |  |  |  |  |  |  |  |  |  |  |  |  |  |  |  |
|  |  | | | | | | | | | | | | | | | |  |
|  | | | | | | | | | | | |  |  |  |  |  |  |
|  | INTERVIEWER: In your view as the researcher  RESPONDENT: in my view as a researcher?  INTERVIEWER: would you return or communicate to the participants?  RESPONDENT: I think I would, yah if the conditions allow, I think I would, because if their life will depend on that, or they are likely to improve their quality of life. I think its humane to communicate and if they can get treatment | | | | | | | | | | | | | | | |  |
|  |  | | | | | | | | | | | | | | | |  |
|  | | | **Files\\Male\\R-001 male** | | | | | | | | | | | | | |  |
|  |  |  | Yes |  |  |  |  |  |  | |  | | | | | |  |
|  | | |  |  |  |  |  |  |  | |  | | | | | | |
|  | | | | | | | | | | | |  |  |  |  |  |  |
|  | I would return those results to Patient if i knew those results were going to help a patient to be treated well, or if i knew they were going to affect his Prognosis. If a Patient is not going to do well, its good to tell them that because of "A, B, and C", you are likely not to do very well in this state. | | | | | | | | | | | | | | | |  |
|  |  | | | | | | | | | | | | | | | |  |
|  | **Nodes\\Return of results\\mode of returning results** | | | | | | | | | | | | | | | |  |
|  | | **Document** | | | | | | | | | | | | | | |  |
|  | | | **Files\\Female\\R027.female** | | | | | | | | | | | | | |  |
|  |  |  | No |  |  |  |  |  |  | |  | | | | | |  |
|  | | |  |  |  |  |  |  |  | |  | | | | | | |
|  | | | | | | | | | | | |  |  |  |  |  |  |
|  | INTERVIEWER: So now when you feel there is need to report some of those results, would you advise on a way how may be this should be done?  RESPONDENT: In a simplified way as possible, many times we complicate things and we stick to complicated words but we can break it down to something easier then pictorial to slides then people can understand better than just using big words. | | | | | | | | | | | | | | | |  |
|  |  | | | | | | | | | | | | | | | |  |
| Reports\\Coding Summary By Code Report | | | | | | | | | | Page 9 of 109 | | | | | | | |
|  | | | | | | | | | | | | | | | | | |
|  | | | **Aggregate** |  | **Classification** |  | **Coverage** |  | **Number Of Coding References** | |  | **Reference Number** |  | **Coded By Initials** |  | **Modified On** |  |
|  | | | **Files\\Male\\R-017. male** | | | | | | | | | | | | | |  |
|  |  |  | No |  |  |  |  |  |  | |  | | | | | |  |
|  | | |  |  |  |  |  |  |  | |  | | | | | | |
|  | | | | | | | | | | | |  |  |  |  |  |  |
|  | Respondent: We don’t report the results directly, like I said there are different media of reporting back, publishing in international journals, seminars, but also to the community. You can report back to the community that we have these findings, however this being an health based research you find that there are always statistics that are obtained from the hospital about those particular diseases and our information plays part in those statistics, yes, like you go to a population and find that these number of individuals have this particular disease, it’s a report that is also used by the health demographic surveys, so those are different media through which we report our different results | | | | | | | | | | | | | | | |  |
|  |  | | | | | | | | | | | | | | | |  |
|  | | | | | | | | | | | |  |  |  |  |  |  |
|  | Respondent: Yes, the results we get we report through conferences-reporting to wider community, we publish this information in international journals, and also report back to the ministry | | | | | | | | | | | | | | | |  |
|  |  | | | | | | | | | | | | | | | |  |
|  | | | **Files\\Male\\R-023. male** | | | | | | | | | | | | | |  |
|  |  |  | No |  |  |  |  |  |  | |  | | | | | |  |
|  | | |  |  |  |  |  |  |  | |  | | | | | | |
|  | | | | | | | | | | | |  |  |  |  |  |  |
|  | Respondent: So the procedures that are there, essentially you write a report and disseminate it, and I don’t think it is enforced very much, but ideally you would either go back to the community, and call a meeting and maybe tell them, that this is what we found, but some times the challenge would be who does it, coz yes I might have done it but a lot of these studies some times are also nested in other studies, we are also looking at, so then maybe what is not clear is really who is responsible for going back and reporting to the community, I know the obligation is on the researcher, but probably everything needs to be streamlined such that, the duty is clearly, you know the guidelines are more clear, right now the guidelines are not close, it is like good practice, sometimes you do, sometimes you don’t. | | | | | | | | | | | | | | | |  |
|  |  | | | | | | | | | | | | | | | |  |
|  | | | **Files\\Male\\R-025 male..** | | | | | | | | | | | | | |  |
|  |  |  | No |  |  |  |  |  |  | |  | | | | | |  |
|  | | |  |  |  |  |  |  |  | |  | | | | | | |
|  | | | | | | | | | | | |  |  |  |  |  |  |
|  | Respondent: Ahaa not yet, we have found something in the group of Epistein-Barr bar virus, because we have found certain virus which are more common with cancer cells but this one we are just publishing and then we are trying to see if other centers also get similar results, so we have compared it with blood samples, and it looks like there are certain virus much associated with that, so this is the stage which is there. The next stage of course if it is conclusive, we have compared so far two, but if it come up now that it is this virus most associated with that, then we will just to interest those companies who are trying to develop the vaccines | | | | | | | | | | | | | | | |  |
|  |  | | | | | | | | | | | | | | | |  |
|  | | | | | | | | | | | | | | | | | |
|  | | | | | | | | | | | | | | | | | |
|  | | | | | | | | | | | | | | | | | |
| Reports\\Coding Summary By Code Report | | | | | | | | | | Page 10 of 109 | | | | | | | |
|  | | | | | | | | | | | | | | | | | |
|  | | | **Aggregate** |  | **Classification** |  | **Coverage** |  | **Number Of Coding References** | |  | **Reference Number** |  | **Coded By Initials** |  | **Modified On** |  |
|  | **Nodes\\Return of results\\Necessity** | | | | | | | | | | | | | | | |  |
|  | | **Document** | | | | | | | | | | | | | | |  |
|  | | | **Files\\Female\\R026 female** | | | | | | | | | | | | | |  |
|  |  |  | No |  |  |  |  |  |  | |  | | | | | |  |
|  | | |  |  |  |  |  |  |  | |  | | | | | | |
|  | | | | | | | | | | | |  |  |  |  |  |  |
|  | INTERVIEWER: So, you think it is not necessary to return such results to participants?  RESPONDENT: They are early and they don’t mean much to either the community or the individual, and it would be much better if we were at stage four research where we now show that people with this gene are more likely to get disease and then we can look at how we translate it in day today life but when they are early ones that on chromosome 6 we found three genes related, and even when I put it in simple language, we all know nothing much from now, we are doing more research | | | | | | | | | | | | | | | |  |
|  |  | | | | | | | | | | | | | | | |  |
|  | | | | | | | | | | | |  |  |  |  |  |  |
|  | RESPONDENT: No, it is good to give people some information because if a person says he is involved in the research, and afterwards people just walk away. It is good to come back and say the research we did shows some interesting results but not conclusive, but some times to some people they will say why did you come, and yet its as if you finished went away and then you have come back when you just need samples. And that is why we do it in a very brief way | | | | | | | | | | | | | | | |  |
|  |  | | | | | | | | | | | | | | | |  |
|  | | | **Files\\Female\\R029 female** | | | | | | | | | | | | | |  |
|  |  |  | No |  |  |  |  |  |  | |  | | | | | |  |
|  | | |  |  |  |  |  |  |  | |  | | | | | | |
|  | | | | | | | | | | | |  |  |  |  |  |  |
|  | INTERVIEWER: Ok. But do you think it is necessary to return results for individuals?  RESPONDENT: My particular results may be not but if some results are of clinical relevance, may be its important but I don’t this could be of clinical relevance. I don’t think they determine the kind of treatment such people will need. Because we just want to see if there are any polymorphism that could be associated but could not be confirmatory that these are. For my particular study I dint think that it is necessary to return results to individual participants.  INTERVIEWER: but it is necessary to return them to the hospital?  RESPONDENT: Yes it necessary because we did the study in the hospital, I think they should know what we found. | | | | | | | | | | | | | | | |  |
|  |  |  |  |  |  |  |  |  |  |  |  |  |  |  |  |  |  |
|  |  | | | | | | | | | | | | | | | |  |
|  | | | **Files\\Female\\R030.female** | | | | | | | | | | | | | |  |
|  |  |  | No |  |  |  |  |  |  | |  | | | | | |  |
|  | | |  |  |  |  |  |  |  | |  | | | | | | |
|  | | | | | | | | | | | |  |  |  |  |  |  |
|  | RESPONDENT: So far, we have not but the principle is, if anybody wants to know it is their right and I think it is important. | | | | | | | | | | | | | | | |  |
|  |  | | | | | | | | | | | | | | | |  |
|  | | | **Files\\Male\\R-002. male** | | | | | | | | | | | | | |  |
|  |  |  | No |  |  |  |  |  |  | |  | | | | | |  |
|  | | |  |  |  |  |  |  |  | |  | | | | | | |
|  | | | | | | | | | | | |  |  |  |  |  |  |
|  | Respondent: yes, I think it is very important because it depends on what information to be returned, I think it is important especially if it concerns an individual and there is a potential for future problem coming from generations to come because we know that genetics information and diseases and all this | | | | | | | | | | | | | | | |  |
|  |  | | | | | | | | | | | | | | | |  |
| Reports\\Coding Summary By Code Report | | | | | | | | | | Page 11 of 109 | | | | | | | |
|  | | | | | | | | | | | | | | | | | |
|  | | | **Aggregate** |  | **Classification** |  | **Coverage** |  | **Number Of Coding References** | |  | **Reference Number** |  | **Coded By Initials** |  | **Modified On** |  |
|  | | | **Files\\Male\\R-004.male** | | | | | | | | | | | | | |  |
|  |  |  | No |  |  |  |  |  |  | |  | | | | | |  |
|  | | |  |  |  |  |  |  |  | |  | | | | | | |
|  | | | | | | | | | | | |  |  |  |  |  |  |
|  | Respondent: I think it is because when there is a finding the owner of the results has that right to know to get that information | | | | | | | | | | | | | | | |  |
|  |  | | | | | | | | | | | | | | | |  |
|  | | | | | | | | | | | |  |  |  |  |  |  |
|  | Respondent: I would imagine whether it is good or bad the owner of the results has that right to know when there is an important family or when there is a negative finding, they should be given enough information, counseling then information and if possible, a corrective measure can be instituted | | | | | | | | | | | | | | | |  |
|  |  | | | | | | | | | | | | | | | |  |
|  | | | **Files\\Male\\R-006. male** | | | | | | | | | | | | | |  |
|  |  |  | No |  |  |  |  |  |  | |  | | | | | |  |
|  | | |  |  |  |  |  |  |  | |  | | | | | | |
|  | | | | | | | | | | | |  |  |  |  |  |  |
|  | I think it is very important, I would have to say, it also depends on what genetic results we are talking about here, because if like i said it has to do with someone not responding to a treatment properly, it’s really critical that this person knows because if they take this message back home it could help a relative, it could help them also to handle things differently. To handle treatment differently. | | | | | | | | | | | | | | | |  |
|  |  | | | | | | | | | | | | | | | |  |
|  | | | **Files\\Male\\R-007 male** | | | | | | | | | | | | | |  |
|  |  |  | No |  |  |  |  |  |  | |  | | | | | |  |
|  | | |  |  |  |  |  |  |  | |  | | | | | | |
|  | | | | | | | | | | | |  |  |  |  |  |  |
|  | Respondent: Ahm, in terms of their genomics, ahm, at the moment I think it’s too risky, I don’t think its necessary at the moment, No. If I know that this kind of gene, XZY gene makes somebody more susceptible to XZY and , at the it doesn’t require me particularly to return back because I think the implications are still, well, the field is still very virgin I really wouldn’t want to do that kind of thing at the moment. I would want a general. | | | | | | | | | | | | | | | |  |
|  |  | | | | | | | | | | | | | | | |  |
|  | | | **Files\\Male\\R-009.male** | | | | | | | | | | | | | |  |
|  |  |  | No |  |  |  |  |  |  | |  | | | | | |  |
|  | | |  |  |  |  |  |  |  | |  | | | | | | |
|  | | | | | | | | | | | |  |  |  |  |  |  |
|  | RESPONDENT: I think so if the results which, I think you should able to return results to me which am meaning, it could be difficult to return results which don’t mix to the participants I mean for example what would the patient understand with sequence they would only return when it makes sense so am not sure but the community may be can be aware that there were no major findings something like that but it is a very interesting question which we have not obligated ourselves that every result should be returned we have only said the results makes a meaning | | | | | | | | | | | | | | | |  |
|  |  | | | | | | | | | | | | | | | |  |
|  | | | | | | | | | | | |  |  |  |  |  |  |
|  | INTERVIEWER: so, when is it necessary to return for example aggregate results?  RESPONDENT: to who? | | | | | | | | | | | | | | | |  |
|  |  | | | | | | | | | | | | | | | |  |
|  | | | | | | | | | | | | | | | | | |
| Reports\\Coding Summary By Code Report | | | | | | | | | | Page 12 of 109 | | | | | | | |
|  | | | | | | | | | | | | | | | | | |
|  | | | **Aggregate** |  | **Classification** |  | **Coverage** |  | **Number Of Coding References** | |  | **Reference Number** |  | **Coded By Initials** |  | **Modified On** |  |
|  | | | **Files\\Male\\R-025 male..** | | | | | | | | | | | | | |  |
|  |  |  | No |  |  |  |  |  |  | |  | | | | | |  |
|  | | |  |  |  |  |  |  |  | |  | | | | | | |
|  | | | | | | | | | | | |  |  |  |  |  |  |
|  | Interviewer: Is it necessary to return genetic results?  Respondent: Sure it should be necessary to return if a particular aspect is discovered, that there is the down side of it, when you return results and the people are not able to swap themselves, I mean you have no solution for, or you don’t even, you just say there is a lot of genetic defect here, does it mean it is for this individual, or it is all for the community? So anyway but we think that it is necessary to share certain results in a way that is understandable by the community, and then for the Burkitt’s lymphoma we hope that unfortunately this cancer also a bit, the cure rate is not yet very good in this country, it is still at 70% but we hope that those study participants will still be able to get them but then, maybe I should mention this, the only thing that came from that study is that we found people who are more interested in the social aspect of Burkitt’s lymphoma so they have started a firm development here but now they follow the patients of that lymphoma for a longer period of time and also the children within the hospital are given education primary, education mostly and then they are also given some skills, so we are hoping that we do not lose contact with these people. We might give them some information which becomes good | | | | | | | | | | | | | | | |  |
|  |  |  |  |  |  |  |  |  |  |  |  |  |  |  |  |  |  |
|  |  | | | | | | | | | | | | | | | |  |
|  | **Nodes\\Return of results\\Necessity\Individual results** | | | | | | | | | | | | | | | |  |
|  | | **Document** | | | | | | | | | | | | | | |  |
|  | | | **Files\\Male\\R-002. male** | | | | | | | | | | | | | |  |
|  |  |  | No |  |  |  |  |  |  | |  | | | | | |  |
|  | | |  |  |  |  |  |  |  | |  | | | | | | |
|  | | | | | | | | | | | |  |  |  |  |  |  |
|  | Respondent: yes, I think it is very important because it depends on what information to be returned, I think it is important especially if it concerns an individual and there is a potential for future problem coming from generations to come because we know that genetics information and diseases and all this | | | | | | | | | | | | | | | |  |
|  |  | | | | | | | | | | | | | | | |  |
|  | | | | | | | | | | | |  |  |  |  |  |  |
|  | Respondent: especially if it is going to be something that will help them make some decisions or change some things when they come to know about lets say disease you know potential for disease or future disease due to pesticides. I will imagine that to be important one of the things I know for example we have sickle cells, albinism and all this, we have not done a study about these are very wild thoughts but if people get to know about this and the other thing also there is less regular information about genetics but we can always get it and imagine in situations if you know about your genetic status and then maybe later on avoid, you will get a partner who will not be a carrier like you because if both of you are carriers eventually some of your off springs will end up with the condition. Ethically that’s something am not sure how to go about but I think, yes we are heading there especially now that you guys are here we are beginning to think about these kinds of things and a similar study may help | | | | | | | | | | | | | | | |  |
|  |  |  |  |  |  |  |  |  |  |  |  |  |  |  |  |  |  |
|  |  | | | | | | | | | | | | | | | |  |
|  | | | | | | | | | | | |  |  |  |  |  |  |
|  | Respondent: no, if it is an individual | | | | | | | | | | | | | | | |  |
|  |  | | | | | | | | | | | | | | | |  |
|  | | | | | | | | | | | |  |  |  |  |  |  |
|  | Respondent: but it will be important to have like those members of the family to look at it but I think first of all the individual and getting to know if they want to share with their other family members because it is a bit personal | | | | | | | | | | | | | | | |  |
|  |  | | | | | | | | | | | | | | | |  |
|  | | | | | | | | | | | |  |  |  |  |  |  |
|  | Respondent: it’s a trend because it is good for the community to know what is circulating with in or what is going on with them but also once they get to know always there should be a way out so as we go out to bring these results back we should always have at the back of our mind what is the way out or how can they handle this because that question will come so we can share our thoughts with them and leave to them to make decisions | | | | | | | | | | | | | | | |  |
|  |  | | | | | | | | | | | | | | | |  |
| Reports\\Coding Summary By Code Report | | | | | | | | | | Page 13 of 109 | | | | | | | |
|  | | | | | | | | | | | | | | | | | |
|  | | | **Aggregate** |  | **Classification** |  | **Coverage** |  | **Number Of Coding References** | |  | **Reference Number** |  | **Coded By Initials** |  | **Modified On** |  |
|  | | | **Files\\Male\\R-004.male** | | | | | | | | | | | | | |  |
|  |  |  | No |  |  |  |  |  |  | |  | | | | | |  |
|  | | |  |  |  |  |  |  |  | |  | | | | | | |
|  | | | | | | | | | | | |  |  |  |  |  |  |
|  | Respondent: I think it is not easy for because these genetic findings are going to be unique to an individual so for the community still, they have to be traced as individuals, the information shouldn’t be given or shared in the community about someone’s genetic makeup | | | | | | | | | | | | | | | |  |
|  |  | | | | | | | | | | | | | | | |  |
|  | | | **Files\\Male\\R-007 male** | | | | | | | | | | | | | |  |
|  |  |  | No |  |  |  |  |  |  | |  | | | | | |  |
|  | | |  |  |  |  |  |  |  | |  | | | | | | |
|  | | | | | | | | | | | |  |  |  |  |  |  |
|  | Interviewer: For individual results,  Respondent: No, no… | | | | | | | | | | | | | | | |  |
|  |  | | | | | | | | | | | | | | | |  |
|  | | | **Files\\Male\\R-013.male** | | | | | | | | | | | | | |  |
|  |  |  | No |  |  |  |  |  |  | |  | | | | | |  |
|  | | |  |  |  |  |  |  |  | |  | | | | | | |
|  | | | | | | | | | | | |  |  |  |  |  |  |
|  | RESPONDENT: very important , to return results to patients however it depends on the kind of study that you are doing most genetics studies here currently the one we do, don’t really involve results that are clinically important to the patient for example if you go and detect the kind of the TB genotypes in the population you’re not going to come and tell the TB patient that you have the Ugandan genotype or the berlin genotype because that information is not important to them because yes so what to the patient however you could say to the fellow clinicians that generally some of the patients have the Uganda genotype and this means these patients possibly are always prone to being resistant. So to the clinical community it could make more sense but individually it is very important that you think also about that kind of data information you’re going to because certain information could not be understandable to the patient and useful to them | | | | | | | | | | | | | | | |  |
|  |  |  |  |  |  |  |  |  |  |  |  |  |  |  |  |  |  |
|  |  | | | | | | | | | | | | | | | |  |
|  | | | | | | | | | | | |  |  |  |  |  |  |
|  | RESPONDENT: you see that information is based disseminated in like a conference or in scientific community here and all that but individual you will give the patients the results individually but the other aggregate information can always be, I think it shows the importance of disseminating your findings CMEs, in conferences in workshops and all that to increase the board of knowledge to the community basically to attract | | | | | | | | | | | | | | | |  |
|  |  | | | | | | | | | | | | | | | |  |
|  | | | | | | | | | | | |  |  |  |  |  |  |
|  | RESPONDENT: to the individual patients but however to the scientific community | | | | | | | | | | | | | | | |  |
|  |  | | | | | | | | | | | | | | | |  |
|  | | | **Files\\Male\\R-025 male..** | | | | | | | | | | | | | |  |
|  |  |  | No |  |  |  |  |  |  | |  | | | | | |  |
|  | | |  |  |  |  |  |  |  | |  | | | | | | |
|  | | | | | | | | | | | |  |  |  |  |  |  |
|  | Interviewer: And when would it be necessary to return individual results?  Respondent: For individual traceable, for this particular one the population is difficult but for the patients I think as soon as results are available for a particular group they return, for the study which we are doing for the diagnostic, this we should inform them as soon as results are available from the analysis. It will not wait for communication for sensitivity or whatever since we are not using it also as a means for treatment | | | | | | | | | | | | | | | |  |
|  |  | | | | | | | | | | | | | | | |  |
|  | | | | | | | | | | | | | | | | | |
| Reports\\Coding Summary By Code Report | | | | | | | | | | Page 14 of 109 | | | | | | | |
|  | | | | | | | | | | | | | | | | | |
|  | | | **Aggregate** |  | **Classification** |  | **Coverage** |  | **Number Of Coding References** | |  | **Reference Number** |  | **Coded By Initials** |  | **Modified On** |  |
|  | **Nodes\\Return of results\\Opinion on return of results** | | | | | | | | | | | | | | | |  |
|  | | **Document** | | | | | | | | | | | | | | |  |
|  | | | **Files\\Female\\R-003.female** | | | | | | | | | | | | | |  |
|  |  |  | Yes |  |  |  |  |  |  | |  | | | | | |  |
|  | | |  |  |  |  |  |  |  | |  | | | | | | |
|  | | | | | | | | | | | |  |  |  |  |  |  |
|  | INTERVIEWER: do you think it is necessary to return genetics results to individuals who have participated in the genetics studies?  RESPONDENT: I think it is a yes and no, it is good if these individuals have really been given basic information for them to understand. you know one of the things I have realized is that because of literacy, there are things which are difficult to explain but for me I know if it is beneficial really if it is not going to, its good for people to know because why would some one spend time in a study and then you get the information and you sit on it. if it is benefial certainly these individuals need to know but the other side of the coin is what happens if this is information that cannot be accepted for instance if it is a couple because it can also cause conflict in a home so it is like a fifty fifty but all the same, I feel that individuals need to know | | | | | | | | | | | | | | | |  |
|  |  |  |  |  |  |  |  |  |  |  |  |  |  |  |  |  |  |
|  |  | | | | | | | | | | | | | | | |  |
|  | | | | | | | | | | | |  |  |  |  |  |  |
|  | INTERVIEWER: do you think it is necessary to return genetics results to individuals who have participated in the genetics studies?  RESPONDENT: I think it is a yes and no, it is good if these individuals have really been given basic information for them to understand you know one of the things I have realized is that because of literacy, there are things which are difficult to explain but for me I know if it is beneficial really if it is not going to, its good for people to know because why would some one spend time in a study and then you get the information and you sit on it, if it is benefial certainly these individuals need to know but the other side of the coin is what happens if this is information that cannot be accepted for instance if it is a couple because it can also cause conflict in a home so it is like a fifty fifty but all the same I feel that individuals need to know | | | | | | | | | | | | | | | |  |
|  |  |  |  |  |  |  |  |  |  |  |  |  |  |  |  |  |  |
|  |  | | | | | | | | | | | | | | | |  |
|  | | | | | | | | | | | |  |  |  |  |  |  |
|  | INTERVIEWER: so, it shouldn’t be done  RESPONDENT: suppose the community is carrying a bad gene, in a sense that it is a gene that is related to obesity but is something that is ok but am trying to think of something weird maybe the tendency of, these days people tell us that lesbianism and gay is kind of runs in, there is some genetic inclination of, suppose they say that all those people in that community  INTERVIEWER: because when you return aggregate results is to maybe all the team or individuals in that community who have participated not that the outside community and probably when you return them it could help them plan or think about them, there is a way they can intervene some of them have no specific intervene or treatment that can help you understand your situation or adjust the environment so that it will not come back  RESPONDENT: may be | | | | | | | | | | | | | | | |  |
|  |  |  |  |  |  |  |  |  |  |  |  |  |  |  |  |  |  |
|  |  | | | | | | | | | | | | | | | |  |
|  | | | | | | | | | | | |  |  |  |  |  |  |
|  | INTERVIEWER: in case of incidental findings, how do you handle such results? Suppose you went for sickle cells you got it, its easier to handle because you have already obtained informed consent and then telling people about it but sometimes you get findings that were not in the target of our research, how do you handle those ones?  RESPONDENT: the unexpected findings, | | | | | | | | | | | | | | | |  |
|  |  | | | | | | | | | | | | | | | |  |
|  | | | | | | | | | | | |  |  |  |  |  |  |
|  | INTERVIEWER: would you then propose that during the informed consent it should also add a component of incidental findings  RESPONDENT: yes | | | | | | | | | | | | | | | |  |
|  |  | | | | | | | | | | | | | | | |  |
|  | | | **Files\\Female\\R026 female** | | | | | | | | | | | | | |  |
|  |  |  | Yes |  |  |  |  |  |  | |  | | | | | |  |
|  | | |  |  |  |  |  |  |  | |  | | | | | | |
|  | | | | | | | | | | | |  |  |  |  |  |  |
|  | INTERVIEWER: So, what has been the outcome so far from that communication?  RESPONDENT: Again, I didn’t get that feedback | | | | | | | | | | | | | | | |  |
|  |  | | | | | | | | | | | | | | | |  |
| Reports\\Coding Summary By Code Report | | | | | | | | | | Page 15 of 109 | | | | | | | |
|  | | | | | | | | | | | | | | | | | |
|  | | | **Aggregate** |  | **Classification** |  | **Coverage** |  | **Number Of Coding References** | |  | **Reference Number** |  | **Coded By Initials** |  | **Modified On** |  |
|  | | | | | | | | | | | | | | | | | |
|  | | | | | | | | | | | |  |  |  |  |  |  |
|  | INTERVIEWER: So, in your own opinion do you think it is necessary to return results to participants?  RESPONDENT: Hmmm, it is interesting. There are some results which are important to return to participants. You are testing drug A, drug B, we’ve showed that this drug is better, it doesn’t matter which drug you are on but you are well and all the drugs are not different. Now genetic make up is harder, coz even when once you show that this gene on this chromosome, people have a higher risk of getting this its is not safe they will get it or not. Again it is kind of process in evolution, genetic research is a bit slower. So we have some genes that we think are related to TB risk, but related to, we are not 100% sure, but they are more likely to have this gene  INTERVIEWER: So, it is a probability?  RESPONDENT: Exactly, so that is why I said it is a process in evolution, clinically we know that some families are more prone to disease than others, now the genes seem to show probably there is a genetic background to this | | | | | | | | | | | | | | | |  |
|  |  |  |  |  |  |  |  |  |  |  |  |  |  |  |  |  |  |
|  |  | | | | | | | | | | | | | | | |  |
|  | | | | | | | | | | | |  |  |  |  |  |  |
|  | INTERVIEWER: So, you think it is not necessary to return such results to participants?  RESPONDENT: They are early and they don’t mean much to either the community or the individual, and it would be much better if we were at stage four research where we now show that people with this gene are more likely to get disease and then we can look at how we translate it in day today life but when they are early ones that on chromosome 6 we found three genes related, and even when I put it in simple language, we all know nothing much from now, we are doing more research | | | | | | | | | | | | | | | |  |
|  |  | | | | | | | | | | | | | | | |  |
|  | | | | | | | | | | | |  |  |  |  |  |  |
|  | RESPONDENT: Okay let’s say theoretically, that in the study I look at these genes and I look at let’s say the gene is common in TB people but also found in people with colon cancer then I say, haaa 10% of the population had a gene that is not only in TB but that gene is also linked to colon cancer. Now one would think a lot of how you bring out that information, because having a gene doesn’t mean one will have the disease but then knowing that you have the gene and the risk, one can have preventive measures. Now if feel that this will impact on a person, I would divulge that information to an individual and to the family, that in your family there were six participants and 3 had a problem, and that information should not be given by a home visitor, it should be by the physician/doctor/PI because it is serious stuff and again you should come up with a reason for bringing up the information. So, we suggest in your family people should be tested for colon cancer by doing these tests on a regular basis or we found this gene for breast cancer so in your family people should test for breast cancer on a regular basis. Then there are some genes where, yes, I find a gene for people getting dementia at 60/70 years, I would wonder what is the probability that that person would get dementia and what will the effect be? Will it be to that person’s advantage? Here is a person, 60 years working, enjoying their life and then you tell them by the way you have a gene for dementia, aren’t I going to make that person’s life worse? So, I think one has to balance what it means and if it is a family it gets even it gets even more remarkable, especially if nothing can be done in that situation | | | | | | | | | | | | | | | |  |
|  |  |  |  |  |  |  |  |  |  |  |  |  |  |  |  |  |  |
|  |  | | | | | | | | | | | | | | | |  |
|  | | | | | | | | | | | |  |  |  |  |  |  |
|  | INTERVIEWER: So, if nothing can be done in that situation, you would recommend not to?  RESPONDENT: Again, it depends on when the disease surfaces, if a disease is common around 40 years, its is good for people to be aware that there is a disease that needs to shape in, it is common around 40 years, has anybody ever got it? And they say, oh yes, we once had an uncle, it is common in men and women and they’ve gotten this chance so be aware. But if you say you get dementia at 70 and it is common, then I would leave the person alone because I would say it is slow, it gradual, we can’t do anything but if we can do something that is different, so again there are many factors. It come at 40 years, you have the gene for getting prostate or breast cancer, I advise get regular tests, get an operation, so it depends on the situation | | | | | | | | | | | | | | | |  |
|  |  |  |  |  |  |  |  |  |  |  |  |  |  |  |  |  |  |
|  |  | | | | | | | | | | | | | | | |  |
|  | | | **Files\\Female\\R027.female** | | | | | | | | | | | | | |  |
|  |  |  | Yes |  |  |  |  |  |  | |  | | | | | |  |
|  | | |  |  |  |  |  |  |  | |  | | | | | | |
|  | | | | | | | | | | | |  |  |  |  |  |  |
|  | INTERVIEWER: You could not go back and give because you had this information that you are more susceptible or you may get more severe reactions compared to may be someone with another gene or something like that?  RESPONDENT: We could have but we didn’t. it is very scientific that some of those things don’t make sense to even scientists. | | | | | | | | | | | | | | | |  |
|  |  | | | | | | | | | | | | | | | |  |
|  | | | | | | | | | | | |  |  |  |  |  |  |
|  | INTERVIEWER: But generally, do you think it is necessary to return genetics genomics results?  RESPONDENT: It depends, there are some things that may not be useful to the patient then there are some that might be useful so if the group of people investigated to the some is useful for them to know then yes, you might be looking for some strange thing there that doesn’t really make sense and doesn’t matter to them | | | | | | | | | | | | | | | |  |
|  |  | | | | | | | | | | | | | | | |  |
| Reports\\Coding Summary By Code Report | | | | | | | | | | Page 16 of 109 | | | | | | | |
|  | | | | | | | | | | | | | | | | | |
|  | | | **Aggregate** |  | **Classification** |  | **Coverage** |  | **Number Of Coding References** | |  | **Reference Number** |  | **Coded By Initials** |  | **Modified On** |  |
|  | | | | | | | | | | | | | | | | | |
|  | | | | | | | | | | | |  |  |  |  |  |  |
|  | INTERVIEWER: Now, for example in case of incidental findings, how should they be handled?  RESPONDENT: Incidental, I don’t know what you are talking about because we don’t have that research where we are talking about those things that affect your child or your community.  INTERVIEWER: No, incidental findings are things that maybe you didn’t anticipate but along the way you realize or you find them out but then they are not addressing any of your basis but you feel it should be important. For example; I have given an example if you are doing something to do with sickle cell you may have to do with paternity but then along the way you realize that may be that child does not really belong to that family, it is not exactly what you were looking for but it came along the way and then you found out that this child doesn’t belong to this family. So, what would you advise, do you advise to communicate to such a family? | | | | | | | | | | | | | | | |  |
|  |  |  |  |  |  |  |  |  |  |  |  |  |  |  |  |  |  |
|  |  | | | | | | | | | | | | | | | |  |
|  | | | | | | | | | | | |  |  |  |  |  |  |
|  | INTERVIEWER: I think that is kind of anticipated in paternity however, if we were to look at things that are may be related to disease like may be someone prone to a specific disease that for you, you are dealing with another line of disease then you realize that this gene or this person might get another condition so in that case it is not something you anticipated because it is not obvious but it can cover.  RESPONDENT: But in every research you know that there are somethings that can come up that are not anticipated so I think there is a way that you can cover and ask your patients if they will find anything that is not anticipated, would you like to be informed? Because not every one would want to know that they are prone to breast cancer, you might have gone for cervical cancer screening and they may find a gene that you are prone to sudden death like these people who just collapse, they don’t want to know, they want to enjoy life and they might make the quality of their life worse and say that I would still want to know at the beginning if there is anything which you would like to know. Or if I find it, I can call and say that I found some results which I need to inform you of the results of the genotype that I don’t think there is some information that some people are better off without. I will give you an example; people who go to test and see if their babies have abnormalities, they do that a lot either to terminate or to continue. In Uganda here, we don’t have that culture of terminating the baby because you hear the baby has Downs syndrome or whatever so if you do that test usually the probability is fifty and you can have Downs syndrome or that, so if a doctor tells you, you have a 50% chance of giving birth to a child with down syndrome, you can terminate the pregnancy but again there is a 50% chance of not having it. But for us we already have the knowledge and you might even terminate because of you don’t want to go down that road then you terminate when actually the child was normal so I can’t just dish out information, I need to tell you that there is this test, you can find out. Do you want to know? | | | | | | | | | | | | | | | |  |
|  |  |  |  |  |  |  |  |  |  |  |  |  |  |  |  |  |  |
|  |  |  |  |  |  |  |  |  |  |  |  |  |  |  |  |  |  |
|  |  | | | | | | | | | | | | | | | |  |
|  | | | | | | | | | | | |  |  |  |  |  |  |
|  | INTERVIEWER: What do you say about community consent, like you said there are some studies where I come as an individual unlike in genetic studies, in some studies you realise that it is not just about my family, about my entire community so is it ethical for researchers to just get my consent?  RESPONDENT: I think it depends, if you are going to do the study in the community like I say I am going to Lira district and move in the villages and test people you definitely have to get their leadership and the community buying but if you are from Lira and you happen to be in Kampala and you are just a random person because you are from Mulago I test, you don’t know the need to go door and say we are doing it, but if it is just from our OPD out patient department and people are walking in then there I think it is okay but if you are doing community based studies, you always need to do that as opposed to hospital based study where everybody is from everywhere. | | | | | | | | | | | | | | | |  |
|  |  |  |  |  |  |  |  |  |  |  |  |  |  |  |  |  |  |
|  |  | | | | | | | | | | | | | | | |  |
|  | | | | | | | | | | | |  |  |  |  |  |  |
|  | INTERVIEWER: So doctor still on that, what do you have to say about sharing of benefits of the research findings with the community for example; there is this study that has come and there are the research and may be twenty years later there is that drug for those who participated?  RESPONDENT: They should get it for free. | | | | | | | | | | | | | | | |  |
|  |  | | | | | | | | | | | | | | | |  |
|  | | | | | | | | | | | |  |  |  |  |  |  |
|  | INTERVIEWER: For example; the people who could have participated some of them are dead.  RESPONDENT: If that trial was performed in a certain community and the research that we are doing as a community that you are going to do I think they should benefit. Just like oil, if they find oil, the drilling of the oil may be thirty years from now but all, those peopled are dead, who consented then they should bring the money back. | | | | | | | | | | | | | | | |  |
|  |  | | | | | | | | | | | | | | | |  |
|  | | | | | | | | | | | |  |  |  |  |  |  |
|  | INTERVIEWER: So if the community participated it doesn’t have to look out for those who are involved or the real participants and it is open to any one  RESPONDENT: For motivation for example if it is like a drug and they could use their genetic material and they get like a drug then that particular patient in that community if still alive should be getting for free and for the rest of the community you can subside if there were some community setting if it was some community involvement. | | | | | | | | | | | | | | | |  |
|  |  | | | | | | | | | | | | | | | |  |
|  | | | | | | | | | | | |  |  |  |  |  |  |
|  | RESPONDENT: Since you are talking about returning results  INTERVIEWER: It will influence issues and some of these guidelines will be incorporated in. | | | | | | | | | | | | | | | |  |
|  |  | | | | | | | | | | | | | | | |  |
| Reports\\Coding Summary By Code Report | | | | | | | | | | Page 17 of 109 | | | | | | | |
|  | | | | | | | | | | | | | | | | | |
|  | | | **Aggregate** |  | **Classification** |  | **Coverage** |  | **Number Of Coding References** | |  | **Reference Number** |  | **Coded By Initials** |  | **Modified On** |  |
|  | | | **Files\\Female\\R028.female** | | | | | | | | | | | | | |  |
|  |  |  | Yes |  |  |  |  |  |  | |  | | | | | |  |
|  | | |  |  |  |  |  |  |  | |  | | | | | | |
|  | | | | | | | | | | | |  |  |  |  |  |  |
|  | RESPONDENT: I think it depends on what the research is about, what diseases are involved, because most of the information we are getting now is not definite. It just helps us to understand this disease better. It does not mean that even if we found these high risky genes that they are necessarily a problem because there are many women with them and they don’t have a problem, so why am saying it depends on the disease is because there some now known genetic variants which are almost sure deal, you have them you are at very increased risk of that disease, I think like in cancer of the breast. Some of those it may be necessary to tell someone because there is something they can do about it, but in this case, there is even nothing that you can do about it even if you know your genetic variants at that time, so it makes no difference to know | | | | | | | | | | | | | | | |  |
|  |  |  |  |  |  |  |  |  |  |  |  |  |  |  |  |  |  |
|  |  | | | | | | | | | | | | | | | |  |
|  | | | | | | | | | | | |  |  |  |  |  |  |
|  | INTERVIEWER: So, in case of incidental findings, how do you handle them  RESPONDENT: Hmmm, well I don’t believe in opening unopened boxes | | | | | | | | | | | | | | | |  |
|  |  | | | | | | | | | | | | | | | |  |
|  | | | | | | | | | | | |  |  |  |  |  |  |
|  | INTERVIEWER: Okay now for example you said that you link your findings to the ethnic group of participants, so you realize sometimes that the alleged father of that child is not the real father, how would you handle such a case?  RESPONDENT: Well I did not come across such a case but we found incompatibilism between the mother and the child, like definitely where this mother can’t be the mother of that baby. But usually is the first issue being that there were mixing up of samples in the lab. And it is actually, I know because I did my PhD at Cambridge, we had a few cases here but it was not even rare in the UK or US to happen, and even but I think they say there is even an acceptable percentage. Those studies where you have mother-baby incompatible, but remember I don’t have the father’s DNA. So, what I have is mother, but I can be able to work out whether this mother is really the mother, I don’t know the father, so I didn’t really have to deal with paternity issues because I only had mother. So, assuming whatever is not for the mother is for the father whom I didn’t know. So that is what makes it a big difference. If you know both DNA, father, mother and baby, then you are likely to face those challenges but even if you did, those samples we got we attributed them to a mix up and of course some are not analyzed further. For example if a woman has blood group BB, and her baby is OO, definitely that is not her baby but at that moment, when you are seeing this 6 months, probably when the baby is up and running, and you are in some lab in the UK and you are saying there is incompatibility here, so one thing I know is that some people have done studies where paternity has been a challenge, but one thing I know is that they are not obliged to give that information. Am an obstetrician, many times we do know that a certain man who is busy around the woman is not the father of the child but it is not our role, the best we tell the woman is that I think you are playing with fire, you need to deal with the situation, but we do not feel obliged that this woman is lying to you. SO even if I had known that paternity was wrong, I was not obliged because of incidental findings to get them, the only thing you can ask is the person who knows. First of all, if both don’t know, that is their business, but what we also used to get information about ethnicity, we would then get to know, some times the woman would tell you that that man is not my husband, he’s not the father of this pregnancy. That information we have sometimes, but she would go on to tell us through ethnicity but we never though obliged to tell them  INTERVIEWER: Okay if you found that a patient was at risk, for example had a gene exposing them to a certain risk of cancer and may be there is something they can do about it….  RESPONDENT: That is why I said if there is anything you can do about it. But by the way remember, I know you have talked to many genetic researchers, when you are looking for genes, depending on what you are looking for, you are looking for your genes not all the genes, so it is not easy to come up with incidental findings because you are not scanning the entire genome, some of course are, the genome wide, but the kind I was doing, I was specific, all my focus was on a particular loci which makes the difference, but when you go looking for everything, then you are likely to come up with those issues | | | | | | | | | | | | | | | |  |
|  |  |  |  |  |  |  |  |  |  |  |  |  |  |  |  |  |  |
|  |  |  |  |  |  |  |  |  |  |  |  |  |  |  |  |  |  |
|  |  |  |  |  |  |  |  |  |  |  |  |  |  |  |  |  |  |
|  |  | | | | | | | | | | | | | | | |  |
|  | | | **Files\\Female\\R029 female** | | | | | | | | | | | | | |  |
|  |  |  | Yes |  |  |  |  |  |  | |  | | | | | |  |
|  | | |  |  |  |  |  |  |  | |  | | | | | | |
|  | | | | | | | | | | | |  |  |  |  |  |  |
|  | INTERVIEWER: What if it has no impact on their lives would it be good not to communicate it?  RESPONDENT: if they have no impact on their lives like what kind of scenario would have no impact? | | | | | | | | | | | | | | | |  |
|  |  | | | | | | | | | | | | | | | |  |
|  | | | | | | | | | | | | | | | | | |
| Reports\\Coding Summary By Code Report | | | | | | | | | | Page 18 of 109 | | | | | | | |
|  | | | | | | | | | | | | | | | | | |
|  | | | **Aggregate** |  | **Classification** |  | **Coverage** |  | **Number Of Coding References** | |  | **Reference Number** |  | **Coded By Initials** |  | **Modified On** |  |
|  | | | | | | | | | | | | | | | | | |
|  | | | | | | | | | | | |  |  |  |  |  |  |
|  | INTERVIEWER: Let me give you an example, like the sickle cell study that was carried out in mulago, and during the study they realized that the people they thought were fathers were not actually the fathers because they carried out the paternity tests though it was not the primary motive of the study. Even if you kept quiet as the researcher, these would have no impact because the father and mother are already living together, but in your heart as researcher, you know that the “father” is not the biological one. Would you go ahead and return such results?  RESPONDENT: well they are incidental, and these people are expecting such results in such a scenario, there repercussions of such a scenario and of course we don’t want to brake people’s families as researchers and you don’t want to spoil your participant’s lives. This is something critical but how about if include in the consent form that incase of any incidental findings may be can we return them to you if they consent to that, maybe there is a way you can approach it and may be if I had not put it in the consent form I don’t know what I would do about that. | | | | | | | | | | | | | | | |  |
|  |  |  |  |  |  |  |  |  |  |  |  |  |  |  |  |  |  |
|  |  | | | | | | | | | | | | | | | |  |
|  | | | **Files\\Female\\R030.female** | | | | | | | | | | | | | |  |
|  |  |  | Yes |  |  |  |  |  |  | |  | | | | | |  |
|  | | |  |  |  |  |  |  |  | |  | | | | | | |
|  | | | | | | | | | | | |  |  |  |  |  |  |
|  | RESPONDENT: Yes, if they do, we have them in aggregate through one of us and their medicine team because we already have information of what is it in the gene, we are due to get another presentation but it has been found that the genes of these people are like those of people who have high cholesterol because cholesterol is a gene since you and mean do not look big but we may have cholesterol so they are studying that further to understand why? Why is this relationship between the cholesterol gene and the long term progressors? | | | | | | | | | | | | | | | |  |
|  |  | | | | | | | | | | | | | | | |  |
|  | | | | | | | | | | | |  |  |  |  |  |  |
|  | INTERVIEWER: In case there are incidental findings, how do you handle them?  RESPONDENT: If there are incidental findings, so what exactly do you mean? Like the lab goes in and they find something they didn’t expect. | | | | | | | | | | | | | | | |  |
|  |  | | | | | | | | | | | | | | | |  |
|  | | | **Files\\Male\\R-001 male** | | | | | | | | | | | | | |  |
|  |  |  | Yes |  |  |  |  |  |  | |  | | | | | |  |
|  | | |  |  |  |  |  |  |  | |  | | | | | | |
|  | | | | | | | | | | | |  |  |  |  |  |  |
|  | I think it's a dilemma because sometimes you may report back something and it gets misunderstood, but usually it would be important if that affects the way you are going to manage the Patient different from another Patient, its important that you report and say, This is what we found and we think its going to help you if we do this for you. | | | | | | | | | | | | | | | |  |
|  |  | | | | | | | | | | | | | | | |  |
|  | | | | | | | | | | | |  |  |  |  |  |  |
|  | sometimes aggregate results might not be really beneficial, because who are you really giving them to? Unless you, in the end you want to drive a kind of test. ok, if you say we have done a study and found that People with this genotype don't usually do very well, then people will go and test if they really have that genotype. Probably that would be the benefit of aggregate results to the community. But i have found that sometimes, its really difficult to communicate some of this information because it easily gets misunderstood by people. if i say if you have blood group "o", you will not suffer from Malaria,maybe you will find people going to test for their blood groups and the hope is that if i have blood group " o" , i dont suffer from malaria, but we know that in science there is no 100% . i could be of the said blood group and suffer from malaria. | | | | | | | | | | | | | | | |  |
|  |  |  |  |  |  |  |  |  |  |  |  |  |  |  |  |  |  |
|  |  | | | | | | | | | | | | | | | |  |
|  | | | | | | | | | | | |  |  |  |  |  |  |
|  | Incidental fndings are also very difficult at some point because you didn't go for them but you have found them | | | | | | | | | | | | | | | |  |
|  |  | | | | | | | | | | | | | | | |  |
|  | | | | | | | | | | | | | | | | | |
| Reports\\Coding Summary By Code Report | | | | | | | | | | Page 19 of 109 | | | | | | | |
|  | | | | | | | | | | | | | | | | | |
|  | | | **Aggregate** |  | **Classification** |  | **Coverage** |  | **Number Of Coding References** | |  | **Reference Number** |  | **Coded By Initials** |  | **Modified On** |  |
|  | | | **Files\\Male\\R-002. male** | | | | | | | | | | | | | |  |
|  |  |  | Yes |  |  |  |  |  |  | |  | | | | | |  |
|  | | |  |  |  |  |  |  |  | |  | | | | | | |
|  | | | | | | | | | | | |  |  |  |  |  |  |
|  | Respondent: I think that would be the best way to general information to the concerned community not target a particular individual, to give that kind of result in the trend but that I would imagine some people might want to know more and when they are approached for the research may that is when you can you in the individual results if they were participants | | | | | | | | | | | | | | | |  |
|  |  | | | | | | | | | | | | | | | |  |
|  | | | **Files\\Male\\R-004.male** | | | | | | | | | | | | | |  |
|  |  |  | Yes |  |  |  |  |  |  | |  | | | | | |  |
|  | | |  |  |  |  |  |  |  | |  | | | | | | |
|  | | | | | | | | | | | |  |  |  |  |  |  |
|  | Respondent: you see something that is familiar there it has implications on the family and the family should know and I wonder whether the community comes in this case because the privacy of the family will be exposed but for something that is familial not just a mutant occurring then there a family should know | | | | | | | | | | | | | | | |  |
|  |  | | | | | | | | | | | | | | | |  |
|  | | | **Files\\Male\\R-005.male** | | | | | | | | | | | | | |  |
|  |  |  | Yes |  |  |  |  |  |  | |  | | | | | |  |
|  | | |  |  |  |  |  |  |  | |  | | | | | | |
|  | | | | | | | | | | | |  |  |  |  |  |  |
|  | Interviewer: So those are individual results, when would it be necessary to return aggregate results?  Respondent: I don’t know may be because what I know is that for us we were giving individual….  Interviewer: Would it be okay to return aggregate results?  Respondent: Now let me think about it, I think that needs some kind of preparation of the community, the people who are going to receive the results. Sometimes preparation involves preparing people like sometimes some things are related with stigma, if you are going to give information that is not good, so you need to prepare them | | | | | | | | | | | | | | | |  |
|  |  | | | | | | | | | | | | | | | |  |
|  | | | | | | | | | | | |  |  |  |  |  |  |
|  | Respondent: So the preparation is two way, e.g you yourself, are you ready to give the kind of results you are going to give? And then the question is that what are you going to say? And are you going to give a wrong information, because you need to mind that when you say whatever you say, society tends to interpret things differently, it’s not the same information like when someone comes and tells you, you have this mutation in a given gene let’s say the brac gene, and then someone would say, oh am going to die, the question doesn’t mean that because you have this mutation you are going to die. So you the person who is communicating this kind of information, how are you going to say it? Are you going to give the impression that someone is going to die, and all the breasts cut off? So I think the preparation is two way, preparing the people who are going to receive but even you as a researcher, you must prepare yourself, on how am I going to communicate, what do you think is going to be the response of the community and is it necessary to give that kind of response? | | | | | | | | | | | | | | | |  |
|  |  |  |  |  |  |  |  |  |  |  |  |  |  |  |  |  |  |
|  |  | | | | | | | | | | | | | | | |  |
|  | | | **Files\\Male\\R-006. male** | | | | | | | | | | | | | |  |
|  |  |  | Yes |  |  |  |  |  |  | |  | | | | | |  |
|  | | |  |  |  |  |  |  |  | |  | | | | | | |
|  | | | | | | | | | | | |  |  |  |  |  |  |
|  | RESPONDENT:  Yes, and to their families really. Direct benefits to the patients but also to their families. | | | | | | | | | | | | | | | |  |
|  |  | | | | | | | | | | | | | | | |  |
|  | | | | | | | | | | | |  |  |  |  |  |  |
|  | so i asked that question because ,i think if someone has a pathogen that has genetically changed its structure and now cannot respond to the treatment that a person is getting, it would be important to tell this person that we have to change this treatment because your infection is resistant to the treatment you were on because some time when that happens you have to give treatment that has maybe more side effects or a drug that’s more expensive ,so you have to tell this person why you are giving this treatment. | | | | | | | | | | | | | | | |  |
|  |  | | | | | | | | | | | | | | | |  |
| Reports\\Coding Summary By Code Report | | | | | | | | | | Page 20 of 109 | | | | | | | |
|  | | | | | | | | | | | | | | | | | |
|  | | | **Aggregate** |  | **Classification** |  | **Coverage** |  | **Number Of Coding References** | |  | **Reference Number** |  | **Coded By Initials** |  | **Modified On** |  |
|  | | | | | | | | | | | | | | | | | |
|  | | | | | | | | | | | |  |  |  |  |  |  |
|  | if it is a familiar kind of condition, or a kind of condition within a family then you would, it would be necessary to send the message to everyone in the family if it is possible, and if the procedures to do that are clear and well structured, but also in the case of drug resistance for example, if you have drug resistant bug in a particular community you would have to go back in the community and tell everyone that we have these, according to genetics, particular drugs are becoming resistant because of misuse of antibiotics or anything like that. so i think, when the genetic information affects the entire family or affects the general population, i think it will definitely be necessary to return results to the community as an aggregate and not necessarily to individuals. | | | | | | | | | | | | | | | |  |
|  |  | | | | | | | | | | | | | | | |  |
|  | | | | | | | | | | | |  |  |  |  |  |  |
|  | That is very interesting but complex question because if you found out something in your research and this is not necessarily. So it depends if what you are finding out in your research a particular condition you were not looking for, and you think it is linked to the ill health of the individual or the ill health of the community, I think it would be worthwhile to go back to discuss with especially the ethics bodies how such results would have to be disseminated because, this is not something you set out to do, and the ethical bodies didn't expect you to look for them, these things you found them incidentally. i think it would be better to go back to the ethics bodies and discuss how this information would have to go back because it would be beneficial to the individual and to the community. | | | | | | | | | | | | | | | |  |
|  |  | | | | | | | | | | | | | | | |  |
|  | | | **Files\\Male\\R-007 male** | | | | | | | | | | | | | |  |
|  |  |  | Yes |  |  |  |  |  |  | |  | | | | | |  |
|  | | |  |  |  |  |  |  |  | |  | | | | | | |
|  | | | | | | | | | | | |  |  |  |  |  |  |
|  | Respondent: Ahm, in terms of their genomics, ahm, at the moment I think it’s too risky, I don’t think its necessary at the moment, No. If I know that this kind of gene, XZY gene makes somebody more susceptible to XZY and, at the end it doesn’t require me particularly to return back because I think the implications are still, well, the field is still very virgin I really wouldn’t want to do that kind of thing at the moment. I would want a general. | | | | | | | | | | | | | | | |  |
|  |  | | | | | | | | | | | | | | | |  |
|  | | | | | | | | | | | |  |  |  |  |  |  |
|  | Respondent: For aggregate results, yes, we can, haa you know, aggregate results are not going to be pointing to any particular individuals, so whatever their implications to the individual, the family and on man exist at that level probably. But then as a community you never know, the community could be one community that shares you know, whatever you are trying to conclude about which might make them labeled in a way. So it is still debatable really. Yeah. | | | | | | | | | | | | | | | |  |
|  |  | | | | | | | | | | | | | | | |  |
|  | | | | | | | | | | | |  |  |  |  |  |  |
|  | Qn 3: INTERVIEWER: and incase of incidental findings, who handles such results?  RESPONDENT: So what do you mean by incidental findings for example?  INTERVIEWER: We went to, or we did this study to find out the kinetics of this particular drug in this individual, but as you do the simple thing you discover that the genome has maybe other important findings, they’re not related to your objective but since you do screening and you get the whole genome you’ve discovered there is something of significance to an individual or to your research participants. Do you report those ones, do you return those to the individuals?  RESPONDENT: Well, ahaa you can publish, and you, of course as I said it is all in the de-identified data and you shouldn’t be able to particularly know who is who, ahaa so for the start that can be published and people can find out about it. When it comes to returning to the individual, we again go back to the same situation we were in before which is really, you have to think carefully about what’s the implication to the individual, again family and society. | | | | | | | | | | | | | | | |  |
|  |  |  |  |  |  |  |  |  |  |  |  |  |  |  |  |  |  |
|  |  | | | | | | | | | | | | | | | |  |
|  | | | | | | | | | | | | | | | | | |
|  | | | | | | | | | | | | | | | | | |
| Reports\\Coding Summary By Code Report | | | | | | | | | | Page 21 of 109 | | | | | | | |
|  | | | | | | | | | | | | | | | | | |
|  | | | **Aggregate** |  | **Classification** |  | **Coverage** |  | **Number Of Coding References** | |  | **Reference Number** |  | **Coded By Initials** |  | **Modified On** |  |
|  | | | **Files\\Male\\R-008.male** | | | | | | | | | | | | | |  |
|  |  |  | Yes |  |  |  |  |  |  | |  | | | | | |  |
|  | | |  |  |  |  |  |  |  | |  | | | | | | |
|  | | | | | | | | | | | |  |  |  |  |  |  |
|  | INTERVIEWER: so, they always have that priority, so was your study or is your study of such a nature that you have to report back results? To the participants on what you discover on their genetics  RESPONDENT: I don’t, why would want to know  INTERVIEWER: what do you explain to them  RESPONDENT: you explain to them the study what the study is going to do but we don’t tell them that you’re coming back to tell you what we have found in your gene  INTERVIEWER: why, its not right? Because don’t you think I would want to know if I have that  RESPONDENT: then what?  INTERVIEWER: general curiosity  RESPONDENT: I have told you there is a policy called test and treat so for you do you want to defy that policy? that policy was brought because they realized some people were dying when we tell them go back and they would never come back for results, so everyone who is tested is treated. For you when you tell them to come I have realized this is what you were doing then when you tell them they stop taking the medication I think telling them is not right  INTERVIEWER: so, it is not necessary to return, what of if you give a general picture for example you go to a school or a community then you realise that of the people that have participated you give a general like a percentage instead of giving to an individual , you your like this, your stand is this  RESPONDENT: what I am telling you personally there is a publication I have written yes this one I think I have to do this but now the way people process or preserve that information can be dangerous, you may not have the right words to present it in what you want and then they get from other but here we are looking at the long term of the study but the immediate one test then get treatment then get the information about the disease but at the end of the day tell  INTERVIEWER: so, you let them know that probably these results won’t be disseminated to you, you just contributed to the knowledge  RESPONDENT: yeah, they know from science and what | | | | | | | | | | | | | | | |  |
|  |  |  |  |  |  |  |  |  |  |  |  |  |  |  |  |  |  |
|  |  |  |  |  |  |  |  |  |  |  |  |  |  |  |  |  |  |
|  |  |  |  |  |  |  |  |  |  |  |  |  |  |  |  |  |  |
|  |  | | | | | | | | | | | | | | | |  |
|  | | | **Files\\Male\\R-009.male** | | | | | | | | | | | | | |  |
|  |  |  | Yes |  |  |  |  |  |  | |  | | | | | |  |
|  | | |  |  |  |  |  |  |  | |  | | | | | | |
|  | | | | | | | | | | | |  |  |  |  |  |  |
|  | RESPONDENT: there are results which could make meaning for example if our hunt for genetic markers or genes which lead to rapid progression to HIV so we could if we found such genes and then we went and we knew who had genes in this population and for example if something could be done towards that then it makes a lot of sense to go back and tell such participants especially if there is some thing which could be done that is like in sicklers like I told you those really are either beneficial either prognostically or if something could be done | | | | | | | | | | | | | | | |  |
|  |  | | | | | | | | | | | | | | | |  |
|  | | | | | | | | | | | |  |  |  |  |  |  |
|  | INTERVIEWER: what if nothing can be done you don’t report?  RESPONDENT: those are the sensitivities we have to discuss with the community advisory board because you may think some people might be happy to know that is not going to survive in the next one year, another person might say don’t tell me that kind of information so I think it might you | | | | | | | | | | | | | | | |  |
|  |  | | | | | | | | | | | | | | | |  |
|  | | | | | | | | | | | |  |  |  |  |  |  |
|  | RESPONDENT: it is an interesting thing because most of these data is actually available to the public like now for us in genomics there is a time where we release this data as public information and it can be accessed of course may be I don’t know how, potentially the community has to access that is anonymized and it is not easy to link the people but of course if the information is about a community you begin to discern some things which can affect any body in that community so I don’t know we have not thought about returning because there are even some times there is no mechanism of returning this data how do you give a genetical data base to a person in kabulasoke (remove village) I don’t see how that happens and then again it might be the framework and feasibility and what would be its benefits visa veer putting it in the public domain | | | | | | | | | | | | | | | |  |
|  |  | | | | | | | | | | | | | | | |  |
|  | | | | | | | | | | | | | | | | | |
| Reports\\Coding Summary By Code Report | | | | | | | | | | Page 22 of 109 | | | | | | | |
| 11/11/2020 1:42 PM | | | | | | | | | | | | | | | | | |
|  | | | **Aggregate** |  | **Classification** |  | **Coverage** |  | **Number Of Coding References** | |  | **Reference Number** |  | **Coded By Initials** |  | **Modified On** |  |
|  | | | | | | | | | | | | | | | | | |
|  | | | | | | | | | | | |  |  |  |  |  |  |
|  | RESPONDENT: incidental means things you never really intended to discover but then you get them actually many times that is a discovery, many times you really get to incidental findings for example we had one of our students we were looking at, when you sequence the exosomes, the exosomes are the coding regions but our student was looking at intromoes these are regions in between the other coding regions and was trying to look for greater regions and the student really stumbled in finding that, that region 20% of the people in that region had viruses other viruses not HIV other viruses enriched there, there was one specific virus that was very common and I think that the people who had that virus were probably if its hypothesis go right like we are trying to seek are really long term non progressors they survive more than the other people who don’t have the virus so this is an incidental finding. we were never looking for the virus we were looking for other markers well it depends on how, now how do you take that information to that community? so but if it has benefits, may be if it is proven then it is not particularly your personal information but now this is a virus which is probably in your community which is very common so again that context may be a community might be informed if you don’t know these are complicated issues, those incidental findings again to me I would treat them like the other findings we have talked about, do they have meaning? Is that meaning benefial? If the meaning has a bad outcome, that case still I would handle it in the same way, I wouldn’t say I wasn’t looking for it therefore I am not going to disclose | | | | | | | | | | | | | | | |  |
|  |  |  |  |  |  |  |  |  |  |  |  |  |  |  |  |  |  |
|  |  | | | | | | | | | | | | | | | |  |
|  | | | **Files\\Male\\R-010. male** | | | | | | | | | | | | | |  |
|  |  |  | Yes |  |  |  |  |  |  | |  | | | | | |  |
|  | | |  |  |  |  |  |  |  | |  | | | | | | |
|  | | | | | | | | | | | |  |  |  |  |  |  |
|  | RESPONDENT: I think the decision to return results is partly depends on the sensitivity of the matter and on the consent. That’s why my argument is that it is important that that is included in the consenting process because if a patient has not consented and the matter is controversial and it’s a matter that is not going to take a person’s life then you urge that; maybe No, you are not going to return the results. | | | | | | | | | | | | | | | |  |
|  |  | | | | | | | | | | | | | | | |  |
|  | | | | | | | | | | | |  |  |  |  |  |  |
|  | Let’s take a scenario; person has not consented but the matter is a public health concern, you are going a whole genome sequence and then you find that this person is actually infected with Ebola, you must inform not only the patient but also the authorities, in fact you must follow up this patient. So, at the end of the day, I think the decision to return results is very contextual, several scenarios should be remolded and it’s important that the participants understand these scenarios because if a participant just says “I don’t want to know”. One of the big studies in genomics, there are what we call family trials, I use them a lot because when we get the father, the mother and then the child and then you wake up and this child is not the father’s child, what do you do! | | | | | | | | | | | | | | | |  |
|  |  | | | | | | | | | | | | | | | |  |
|  | | | | | | | | | | | |  |  |  |  |  |  |
|  | INTERVIEWER: have you ever returned aggregate results?  RESPONDENT: Like all results?  INTERVIEWER: Like you go to a community, you get some findings from your study but as you are returning you don’t go to individuals…  RESPONDENT: ideally that is very important. I have not done before because a lot of the studies I am involved in are not larger scale community studies except the CafGEN study actually it’s a very big study but we do a lot of engagement of community usually before we start the study we engage communities and even at the end of the study we engage communities because it is a requirement. Many times a lot of funders are very advanced, they demand that there are mechanisms for dissemination in which we engage communities as well. But apparently NO, we have not engaged in anything like that. Courts of law at times demand this kind of evidence even if there is no study eg; issues to do with paternity. | | | | | | | | | | | | | | | |  |
|  |  |  |  |  |  |  |  |  |  |  |  |  |  |  |  |  |  |
|  |  | | | | | | | | | | | | | | | |  |
|  | | | | | | | | | | | |  |  |  |  |  |  |
|  | INTERVIEWER: Like you go to a community, you get some findings from your study but as you are returning you don’t go to individuals…  RESPONDENT: ideally that is very important. I have not done before because a lot of the studies I am involved in are not larger scale community studies except the CafGEN study actually it’s a very big study but we do a lot of engagement of community usually before we start the study we engage communities and even at the end of the study we engage communities because it is a requirement. Many times a lot of funders are very advanced, they demand that there are mechanisms for dissemination in which we engage communities as well. But apparently NO, we have not engaged in anything like that. Courts of law at times demand this kind of evidence even if there is no study eg; issues to do with paternity. | | | | | | | | | | | | | | | |  |
|  |  |  |  |  |  |  |  |  |  |  |  |  |  |  |  |  |  |
|  |  | | | | | | | | | | | | | | | |  |
|  | | | | | | | | | | | | | | | | | |
| Reports\\Coding Summary By Code Report | | | | | | | | | | Page 23 of 109 | | | | | | | |
|  | | | | | | | | | | | | | | | | | |
|  | | | **Aggregate** |  | **Classification** |  | **Coverage** |  | **Number Of Coding References** | |  | **Reference Number** |  | **Coded By Initials** |  | **Modified On** |  |
|  | | | **Files\\Male\\R-011.male** | | | | | | | | | | | | | |  |
|  |  |  | Yes |  |  |  |  |  |  | |  | | | | | |  |
|  | | |  |  |  |  |  |  |  | |  | | | | | | |
|  | | | | | | | | | | | |  |  |  |  |  |  |
|  | Incidental findings; I think it just depend ,i think a lot of these things really, there is always a REC that reviews to see if what you are doing is ethical, if really there are some findings, i guess its not me as a researcher to decide if should release it, there’s somebody to decide the potential outcome of release of such results. Then there some communities that participate in genetic research, maybe this study is concerned with finding out their ancestry and migration patterns, and then somewhere along the way they find out oh actually what we know about this population, based on the genetic information is different from what is known, Group A ,occupied this land before Group B. Reporting such information to this community creates now land conflicts, so reporting such things is not for us, i wouldn’t say it’s for me to do ,its really, i would say we have to be sensitive about the societies that are there. | | | | | | | | | | | | | | | |  |
|  |  |  |  |  |  |  |  |  |  |  |  |  |  |  |  |  |  |
|  |  | | | | | | | | | | | | | | | |  |
|  | | | **Files\\Male\\R-012.male** | | | | | | | | | | | | | |  |
|  |  |  | Yes |  |  |  |  |  |  | |  | | | | | |  |
|  | | |  |  |  |  |  |  |  | |  | | | | | | |
|  | | | | | | | | | | | |  |  |  |  |  |  |
|  | RESPONDENT: I don’t think I would return results but the generalized results yes, not the individual results in that way yes | | | | | | | | | | | | | | | |  |
|  |  | | | | | | | | | | | | | | | |  |
|  | | | | | | | | | | | |  |  |  |  |  |  |
|  | RESPONDENT: I don’t think I would return results but the generalized results yes, not the individual results in that way yes | | | | | | | | | | | | | | | |  |
|  |  | | | | | | | | | | | | | | | |  |
|  | | | | | | | | | | | |  |  |  |  |  |  |
|  | RESPONDENT: like I have told that I do not coz this is a gene expression I don’t think we do really have incidental findings because its just like all of us have the same genes and for certain reasons not disease. there will always be some structures even for the same person in times of livelihood differences in gene expressions so I don’t expect them very unique and actually from the data I have worked on but not from anything that could be extreme that will require you to report back | | | | | | | | | | | | | | | |  |
|  |  | | | | | | | | | | | | | | | |  |
|  | | | **Files\\Male\\R-013.male** | | | | | | | | | | | | | |  |
|  |  |  | Yes |  |  |  |  |  |  | |  | | | | | |  |
|  | | |  |  |  |  |  |  |  | |  | | | | | | |
|  | | | | | | | | | | | |  |  |  |  |  |  |
|  | RESPONDENT: us we recommend to the clinicians upon seeing the patients because then if I see this I should evaluate for signs of resistance like raising PSA then recurrence of the tumor size and all that and may be any signs of worsening of the disease those could show the clinical signs of resistance in the chemotherapy otherwise this is not something that we can just enforce for change of therapy so we need to recommend clinicians in the course to make sure that they see it fit to follow up these patients more closely the ones that could be with the signs | | | | | | | | | | | | | | | |  |
|  |  | | | | | | | | | | | | | | | |  |
|  | | | | | | | | | | | |  |  |  |  |  |  |
|  | RESPONDENT: very important , to return results to patients however it depends on the kind of study that you are doing most genetics studies here currently the one we do, don’t really involve results that are clinically important to the patient for example if you go and detect the kind of the TB genotypes in the population you’re not going to come and tell the TB patient that you have the Ugandan genotype or the berlin genotype because that information is not important to them because yes so what to the patient however you could say to the fellow clinicians that generally some of the patients have the Uganda genotype and this means these patients possibly are always prone to being resistant, so to the clinical community it could make more sense but individually it is very important that you think also about that kind of data information you’re going to because certain information could not be understandable to the patient and useful to them | | | | | | | | | | | | | | | |  |
|  |  |  |  |  |  |  |  |  |  |  |  |  |  |  |  |  |  |
|  |  | | | | | | | | | | | | | | | |  |
|  | | | | | | | | | | | |  |  |  |  |  |  |
|  | RESPONDENT: I think it is important but it still depends on the setting that you’re in because aggregate results are from what circumstance of Uganda | | | | | | | | | | | | | | | |  |
|  |  | | | | | | | | | | | | | | | |  |
| Reports\\Coding Summary By Code Report | | | | | | | | | | Page 24 of 109 | | | | | | | |
|  | | | | | | | | | | | | | | | | | |
|  | | | **Aggregate** |  | **Classification** |  | **Coverage** |  | **Number Of Coding References** | |  | **Reference Number** |  | **Coded By Initials** |  | **Modified On** |  |
|  | | | | | | | | | | | | | | | | | |
|  | | | | | | | | | | | |  |  |  |  |  |  |
|  | RESPONDENT: you see that information is based disseminated in like a conference or in scientific community here and all that but individual you will give the patients the results individually but the other aggregate information can always be, I think it shows the importance of disseminating your findings CMEs, in conferences in workshops and all that to increase the board of knowledge to the community basically to attract | | | | | | | | | | | | | | | |  |
|  |  | | | | | | | | | | | | | | | |  |
|  | | | | | | | | | | | |  |  |  |  |  |  |
|  | RESPONDENT: I think it still depends, you see one thing is that when you are doing genomics work and basic science work there is work that is not really directly important to the patient but this work is the science that is used to build on, on to many things let’s say developing a vaccine basically its more into building a scientific board of knowledge so most of this kind of work is very hard to really report back to the patient because there is no direct link of its importance to the patient so I think it still depends for example on the kind of work you are doing because as you report this information to patients you only need to think about the so what as in yes you found this gene so what  INTERVIEWER: if you | | | | | | | | | | | | | | | |  |
|  |  | | | | | | | | | | | | | | | |  |
|  | | | **Files\\Male\\R-014 male** | | | | | | | | | | | | | |  |
|  |  |  | Yes |  |  |  |  |  |  | |  | | | | | |  |
|  | | |  |  |  |  |  |  |  | |  | | | | | | |
|  | | | | | | | | | | | |  |  |  |  |  |  |
|  | RESPONDENT: negative or positive | | | | | | | | | | | | | | | |  |
|  |  | | | | | | | | | | | | | | | |  |
|  | | | | | | | | | | | |  |  |  |  |  |  |
|  | RESPONDENT: negative or positive | | | | | | | | | | | | | | | |  |
|  |  | | | | | | | | | | | | | | | |  |
|  | | | | | | | | | | | |  |  |  |  |  |  |
|  | RESPONDENT: that’s why we publish because the biggest limitation for researchers especially if you are working on a post mortem sample is failure to return results. They are like; the patients are dead but if you know you are going to use the dead’s samples, then the people who are still living and those who will live in the future hence publishing data is very important. | | | | | | | | | | | | | | | |  |
|  |  | | | | | | | | | | | | | | | |  |
|  | | | **Files\\Male\\R-016. male** | | | | | | | | | | | | | |  |
|  |  |  | Yes |  |  |  |  |  |  | |  | | | | | |  |
|  | | |  |  |  |  |  |  |  | |  | | | | | | |
|  | | | | | | | | | | | |  |  |  |  |  |  |
|  | RESPONDENT: so, at the end of the study we will, you see the study is such hat we have genetic samples DNA samples from about five countries and then we have each country having like five sites so you have 40,000 DNA samples for you to make sense out of DNA samples genetic research you need 10,000s of results so reporting or knowing your results alone is not something that helps you directly because it is of no benefit you cannot use those results to do anything, the only way we can use those results is if we have that information from those 40,000 participants collected together I hope that helps | | | | | | | | | | | | | | | |  |
|  |  | | | | | | | | | | | | | | | |  |
|  | | | | | | | | | | | |  |  |  |  |  |  |
|  | RESPONDENT: yes, you report aggregate results, I think that is much better | | | | | | | | | | | | | | | |  |
|  |  | | | | | | | | | | | | | | | |  |
|  | | | | | | | | | | | |  |  |  |  |  |  |
|  | INTERVIEWER: are there any conditions, what are conditions in place for you to report such results?  RESPONDENT: to who?  INTERVIEWER: to those communities to inform the bigger communities about their findings  RESPONDENT: we are doing something called genomic wide association so you sequence the genome of a person you say you have 23 chromosomes or whatever it is may be on chromosome six there is this chromosome eight there is this chromosome nine there is this, that information has very little benefit to lay people. that information can only be used by scientists in the future for example to say I think schizophrenia as a disease is found on chromosome six the short arm of chromosome six or something like that now when they are making medications in the future they can now start targeting those molecules. so that finding per say is not helpful to even me myself who is a scientist, I don’t know whether that helps it like knowing the distance between here and moon you can know it but what you’re going to use it for | | | | | | | | | | | | | | | |  |
|  |  |  |  |  |  |  |  |  |  |  |  |  |  |  |  |  |  |
|  |  | | | | | | | | | | | | | | | |  |
| Reports\\Coding Summary By Code Report | | | | | | | | | | Page 25 of 109 | | | | | | | |
|  | | | | | | | | | | | | | | | | | |
|  | | | **Aggregate** |  | **Classification** |  | **Coverage** |  | **Number Of Coding References** | |  | **Reference Number** |  | **Coded By Initials** |  | **Modified On** |  |
|  | | | | | | | | | | | | | | | | | |
|  | | | | | | | | | | | |  |  |  |  |  |  |
|  | RESPONDENT: I cannot speak for the other people, I can only speak for myself and what am telling is that am not going to look for information that I don’t need, am only going to collect information from saliva samples from these individuals and then look for any association because its what we call central study. so you have people with mental illness and then you have people without mental illness then you match them and compare. so you look at the genetics of this individual and the genetics of that individual then you compare, the people who are going to be reading this information will not even know who these participants are, it is coded so that the only information we have is that this is a male 25 and may be Acholi just three questions so they can’t know who this person is they are not supposed to know so that also people don’t get back to you and give you that information in case somebody else hacks this information and get it then go around and say you know what person has a gene of bipolar syndrome or something like that and then you what that means that basically maybe people will not want to associate with you, may be you children with your family or whatever it is so we don’t look for anything else apart from that so usually we get incidental findings when for example you’re going to do genetics studies or something then you that this is person may be has a tumor in the brain and then you have to inform them about it coz it is ethically correct then you find that that’s not what we do | | | | | | | | | | | | | | | |  |
|  |  |  |  |  |  |  |  |  |  |  |  |  |  |  |  |  |  |
|  |  | | | | | | | | | | | | | | | |  |
|  | | | **Files\\Male\\R-017. male** | | | | | | | | | | | | | |  |
|  |  |  | Yes |  |  |  |  |  |  | |  | | | | | |  |
|  | | |  |  |  |  |  |  |  | |  | | | | | | |
|  | | | | | | | | | | | |  |  |  |  |  |  |
|  | Respondent: Yes, it’s the same was you go and seek medical help and they tell you that they are diagnosing certain things and you expect feed back about the sample that is taken. However, you have to have protocol of how you do this, you have to have special people like councilors who interface with the individuals, be it positive or negative results | | | | | | | | | | | | | | | |  |
|  |  | | | | | | | | | | | | | | | |  |
|  | | | | | | | | | | | |  |  |  |  |  |  |
|  | Respondent: I don’t think it is okay, it is subjective and at some point, you might be marginalized for being part of the incidental findings | | | | | | | | | | | | | | | |  |
|  |  | | | | | | | | | | | | | | | |  |
|  | | | | | | | | | | | |  |  |  |  |  |  |
|  | Respondent: I am not obliged to follow up an individual, and am not mandated to identify an individual in my research because one thing that happens to the data as we get it is that we index that data, anonymize the individual’s identity so that I can’t easily trace that this young girl in this village who is affected by this. So, I try to make sure that even if am disseminating this information to an outside collaborator, they do not know the actual identity of this individual | | | | | | | | | | | | | | | |  |
|  |  | | | | | | | | | | | | | | | |  |
|  | | | | | | | | | | | |  |  |  |  |  |  |
|  | Respondent: We would advise the hospital to have that kind of procedure, depending on the kind of research we are doing | | | | | | | | | | | | | | | |  |
|  |  | | | | | | | | | | | | | | | |  |
|  | | | | | | | | | | | |  |  |  |  |  |  |
|  | Interviewer: And do you think all incidental findings must be reported?  Respondent: Depending on the circumstances because incidental findings maybe one of those things that are relevant maybe, its not a life and death, it is an incidental finding relevant to the research but not to the individual, but it is relevant to the wider scientific community that is investigate that | | | | | | | | | | | | | | | |  |
|  |  | | | | | | | | | | | | | | | |  |
|  | | | | | | | | | | | |  |  |  |  |  |  |
|  | Respondent: Yes, that’s why I said you will have to categorize the incidental finding, in terms of is it a life and death, fatal in that if this person doesn’t know, they are at risk. Such finding when you get them, you also weigh the risks, because there is putting a person on a red flag yet it is your suspicion based on your intuition, but then you must first monitor, assess, inquire from other researchers and also may be interface with the clinicians until you are really confident and sure and you know beyond reasonable doubt that if I don’t tell this individual, this will happen. So, you have to give it some bit of reasoning and consultation  Interviewer: Okay | | | | | | | | | | | | | | | |  |
|  |  | | | | | | | | | | | | | | | |  |
|  | | | | | | | | | | | | | | | | | |
| Reports\\Coding Summary By Code Report | | | | | | | | | | Page 26 of 109 | | | | | | | |
|  | | | | | | | | | | | | | | | | | |
|  | | | **Aggregate** |  | **Classification** |  | **Coverage** |  | **Number Of Coding References** | |  | **Reference Number** |  | **Coded By Initials** |  | **Modified On** |  |
|  | | | **Files\\Male\\R-018.male** | | | | | | | | | | | | | |  |
|  |  |  | Yes |  |  |  |  |  |  | |  | | | | | |  |
|  | | |  |  |  |  |  |  |  | |  | | | | | | |
|  | | | | | | | | | | | |  |  |  |  |  |  |
|  | Respondent: Yes, it would be good to report to them what we found in them with respect to susceptibility, but we have not reached that stage, but at the same time there are what we call incidental findings, if we find some genetic effects for which there are remedies, then it becomes our duty to inform them. If you see that for example these days sickle cell carriage is an issue in Uganda. The carriage is rising which means that if a person knows it is more important than never before in Uganda, for people going to marry to know their sickle cell carriage status, so if you find this it would be our duty to inform the people that you see you have this trait, so be careful who you marry, because you might have problems with your kids. But there are those which you can’t tell them, it is something for which there is no remedy, why are you going to worry them? | | | | | | | | | | | | | | | |  |
|  |  |  |  |  |  |  |  |  |  |  |  |  |  |  |  |  |  |
|  |  | | | | | | | | | | | | | | | |  |
|  | | | | | | | | | | | |  |  |  |  |  |  |
|  | the results can help them get some information, like one for sickle cell testing, if you are able to tell some one that you are not a carrier, the mother is a carrier and you have a Sickler, how do you go about that?  Respondent: Depending on how much they know about carriage and whatever, we want to only look at it at the point of a young person who might want to marry some time, that information is most useful to that one rather than the parents | | | | | | | | | | | | | | | |  |
|  |  | | | | | | | | | | | | | | | |  |
|  | | | **Files\\Male\\R-022. male** | | | | | | | | | | | | | |  |
|  |  |  | Yes |  |  |  |  |  |  | |  | | | | | |  |
|  | | |  |  |  |  |  |  |  | |  | | | | | | |
|  | | | | | | | | | | | |  |  |  |  |  |  |
|  | RESPONDENT: of course it is necessary to report results and if someone has a condition that is going to affect their livelihood, you definitely need to report. And like I mentioned, our reports go directly to the clinicians who are helping out on these patients. | | | | | | | | | | | | | | | |  |
|  |  | | | | | | | | | | | | | | | |  |
|  | | | | | | | | | | | |  |  |  |  |  |  |
|  | RESPONDENT: not everything you see could be beneficial to the patients, certain things are entirely for research but in our setting, we are testing specifically for patients and that’s why it is important. | | | | | | | | | | | | | | | |  |
|  |  | | | | | | | | | | | | | | | |  |
|  | | | | | | | | | | | |  |  |  |  |  |  |
|  | RESPONDENT: it is a yes and no, it really depends on what the question is. So, it is a case by case. | | | | | | | | | | | | | | | |  |
|  |  | | | | | | | | | | | | | | | |  |
|  | | | | | | | | | | | |  |  |  |  |  |  |
|  | INTERVIEWER: let’s try to look at both cases; when would it be necessary to?  RESPONDENT: if it really has a big implication on public health that needs to be reported but sometimes it may not have a direct benefit. So, you weigh the risks and benefits.  INTERVIEWER: so, if it doesn’t have a public health implication you may not report?  RESPONDENT: no  INTERVIEWER: its not an obligation to report?  RESPONDENT: no | | | | | | | | | | | | | | | |  |
|  |  |  |  |  |  |  |  |  |  |  |  |  |  |  |  |  |  |
|  |  | | | | | | | | | | | | | | | |  |
|  | | | | | | | | | | | |  |  |  |  |  |  |
|  | RESPONDENT: as you know, findings may be incidental, that is part of research; sometime you can have a hypothesis, probably its believing in a certain thing; you say, this causes this but sometimes you may find the contrary. Those are all incidental and that’s why you do research anyway because you don’t actually know. | | | | | | | | | | | | | | | |  |
|  |  | | | | | | | | | | | | | | | |  |
|  | | | | | | | | | | | | | | | | | |
| Reports\\Coding Summary By Code Report | | | | | | | | | | Page 27 of 109 | | | | | | | |
|  | | | | | | | | | | | | | | | | | |
|  | | | **Aggregate** |  | **Classification** |  | **Coverage** |  | **Number Of Coding References** | |  | **Reference Number** |  | **Coded By Initials** |  | **Modified On** |  |
|  | | | **Files\\Male\\R-024.male** | | | | | | | | | | | | | |  |
|  |  |  | Yes |  |  |  |  |  |  | |  | | | | | |  |
|  | | |  |  |  |  |  |  |  | |  | | | | | | |
|  | | | | | | | | | | | |  |  |  |  |  |  |
|  | Respondent: Yes its very necessary and I can tell you of two instances that have social connotations one is a child whom we managed some years ago and that time we referred them for tests but because this was genetic tests the results were brought back to me as clinician at a time and when I gave the parents the results they managed to manage this child smoothly until this year when the child passed on. But the child was in medical school, you see they managed to manage the child up to adulthood so it’s very important and so is for all other conditions that we diagnose we tell parents on how to manage children with thalassemia, and then we tell them when they develop an illness what they should do. So, this very useful in terms of public health intervention but also very useful in terms of constructing cohorts of patients who have a condition like that. So, now like we have got like over 2000 well documented cases of sickle cell in the region, we have got like 530 well documented cases with G6PD deficiency like that. So, like we also tend to construct cohorts based on genetic material so it’s very useful for both clinical work and also the patients knowing their status. | | | | | | | | | | | | | | | |  |
|  |  |  |  |  |  |  |  |  |  |  |  |  |  |  |  |  |  |
|  |  | | | | | | | | | | | | | | | |  |
|  | | | **Files\\Male\\R-025 male..** | | | | | | | | | | | | | |  |
|  |  |  | Yes |  |  |  |  |  |  | |  | | | | | |  |
|  | | |  |  |  |  |  |  |  | |  | | | | | | |
|  | | | | | | | | | | | |  |  |  |  |  |  |
|  | Interviewer: So, when is it necessary to return aggregate results?  Respondent: Once the study has done the analysis, maybe the other this is that we do not have the capacity in country to do the analysis. Also the study somehow, like this one was under estimate of the amount of money involved in doing the genetic analysis, so I think once the overall genetic analysis is out you just say, unfortunately for communication you see if it comes before you give the feed back to the community, so at that stage when its clear view, the communications are out, then we discuss with the community but for the CAB we do discuss preliminary results | | | | | | | | | | | | | | | |  |
|  |  | | | | | | | | | | | | | | | |  |
|  | | | | | | | | | | | |  |  |  |  |  |  |
|  | Respondent: Ahaa not yet, we have found something in the group of Epstein-Barr virus, because we have found certain virus which are more common with cancer cells but this one we are just publishing and then we are trying to see if other centers also get similar results, so we have compared it with blood samples, and it looks like there are certain virus much associated with that, so this is the stage which is there. The next stage of course if it is conclusive, we have compared so far two, but if it come up now that it is this virus most associated with that, then we will just to interest those companies who are trying to develop the vaccines | | | | | | | | | | | | | | | |  |
|  |  | | | | | | | | | | | | | | | |  |
|  | | | | | | | | | | | |  |  |  |  |  |  |
|  | Interviewer: How about if that condition might be treatable somewhere?  Respondent: Yeah sure, I assume if it is a treatable thing, then you can do, but ours here is mainly cancer and you know cancer is for a longtime period, one has to detect it to screen and then probably later, but the individual must be available to undergo close follow up, but if it is treatable sure  Interviewer: Yeah because you are doing cancer and because you are doing the whole genome, you find some one has a gene for thalassemia or sickle cell or something else, would you report that one to the individual? because it is not what you targeted but as you do it it was inevitable you also had to look at that one  Respondent: Yeah sure, by the way, maybe I didn’t elaborate, this study also looks at non-genetic defect that protects against malaria. Now if, because the thing is that malaria is one of the factors that help decrease your immunity to Burkitt’s lymphoma and may predispose you to Burkitt’s lymphoma, so if you mix malaria, Epstein-Barr virus and then you genetic, it seems there is a genetic part to make. So we are looking at malaria resistance genes and that means that carriers of sickle cell or Sickler, we are also interested to see if there are many of them who have Burkitt’s lymphoma, so for those ones we give them the results. | | | | | | | | | | | | | | | |  |
|  |  |  |  |  |  |  |  |  |  |  |  |  |  |  |  |  |  |
|  |  | | | | | | | | | | | | | | | |  |
|  | | | | | | | | | | | | | | | | | |
|  | | | | | | | | | | | | | | | | | |
| Reports\\Coding Summary By Code Report | | | | | | | | | | Page 28 of 109 | | | | | | | |
|  | | | | | | | | | | | | | | | | | |
|  | | | **Aggregate** |  | **Classification** |  | **Coverage** |  | **Number Of Coding References** | |  | **Reference Number** |  | **Coded By Initials** |  | **Modified On** |  |
|  | **Nodes\\Return of results\\Opinion on return of results\Aggregate results** | | | | | | | | | | | | | | | |  |
|  | | **Document** | | | | | | | | | | | | | | |  |
|  | | | **Files\\Female\\R027.female** | | | | | | | | | | | | | |  |
|  |  |  | Yes |  |  |  |  |  |  | |  | | | | | |  |
|  | | |  |  |  |  |  |  |  | |  | | | | | | |
|  | | | | | | | | | | | |  |  |  |  |  |  |
|  | INTERVIEWER: What do you say about community consent, like you said there are some studies where I come as an individual unlike in genetic studies, in some studies you realise that it is not just about my family, about my entire community so is it ethical for researchers to just get my consent?  RESPONDENT: I think it depends, if you are going to do the study in the community like I say I am going to Lira district and move in the villages and test people you definitely have to get their leadership and the community buy-in, but if you are from Lira and you happen to be in Kampala and you are just a random person because you are from Mulago I test, you don’t know the need to go door and say we are doing it, but if it is just from our OPD out patient department and people are walking in then there I think it is okay but if you are doing community based studies, you always need to that as opposed to hospital based study where everybody is from everywhere. | | | | | | | | | | | | | | | |  |
|  |  |  |  |  |  |  |  |  |  |  |  |  |  |  |  |  |  |
|  |  | | | | | | | | | | | | | | | |  |
|  | | | | | | | | | | | |  |  |  |  |  |  |
|  | INTERVIEWER: For example; the people who could have participated some of them are dead.  RESPONDENT: If that trial was performed in a certain community and the research that we are doing as a community that you are going to do I think they should benefit. Just like oil, if they find oil, the drilling of the oil may be thirty years from now but al, those peopled are dead, who consented then they should bring the money back. | | | | | | | | | | | | | | | |  |
|  |  | | | | | | | | | | | | | | | |  |
|  | | | | | | | | | | | |  |  |  |  |  |  |
|  | INTERVIEWER: So if the community participated it doesn’t have to look out for those who are involved or the real participants and it is open to any one  RESPONDENT: For motivation for example if it is like a drug and they could use their genetic material and they get like a drug then that particular patient in that community if still alive should be getting for free and for the rest of the community you can subside if there were some community setting if it was some community involvement. | | | | | | | | | | | | | | | |  |
|  |  | | | | | | | | | | | | | | | |  |
|  | | | **Files\\Female\\R030.female** | | | | | | | | | | | | | |  |
|  |  |  | Yes |  |  |  |  |  |  | |  | | | | | |  |
|  | | |  |  |  |  |  |  |  | |  | | | | | | |
|  | | | | | | | | | | | |  |  |  |  |  |  |
|  | RESPONDENT: Yes, if they do, we have them in aggregate through one of us and their medicine team because we already have information of what is it in the gene, we are due to get another presentation but it has been found that the genes of these people are like those of people who have high cholesterol because cholesterol is a gene since you and mean do not look big but we may have cholesterol so they are studying that further to understand why? Why is this relationship between the cholesterol gene and the long term progressors? | | | | | | | | | | | | | | | |  |
|  |  | | | | | | | | | | | | | | | |  |
|  | | | **Files\\Male\\R-001 male** | | | | | | | | | | | | | |  |
|  |  |  | Yes |  |  |  |  |  |  | |  | | | | | |  |
|  | | |  |  |  |  |  |  |  | |  | | | | | | |
|  | | | | | | | | | | | |  |  |  |  |  |  |
|  | sometimes aggregate results might not be really beneficial, because who are you really giving them to? Unless you ,in the end you want to drive a kind of test. ok, if you say we have done a study and found that People with this genotype don't usually do very well, then people will go and test if they really have that genotype. Probably that would be the benefit of aggregate results to the community. But i have found that sometimes, its really difficult to communicate some of this information because it easily gets misunderstood by people. if i say if you have blood group "o", you will not suffer from Malaria, maybe you will find people going to test for their blood groups and the hope is that if i have blood group " o", i dont suffer from malaria, but we know that in science there is no 100%. i could be of the said blood group and suffer from malaria. | | | | | | | | | | | | | | | |  |
|  |  |  |  |  |  |  |  |  |  |  |  |  |  |  |  |  |  |
|  |  | | | | | | | | | | | | | | | |  |
| Reports\\Coding Summary By Code Report | | | | | | | | | | Page 29 of 109 | | | | | | | |
|  | | | | | | | | | | | | | | | | | |
|  | | | **Aggregate** |  | **Classification** |  | **Coverage** |  | **Number Of Coding References** | |  | **Reference Number** |  | **Coded By Initials** |  | **Modified On** |  |
|  | | | **Files\\Male\\R-002. male** | | | | | | | | | | | | | |  |
|  |  |  | Yes |  |  |  |  |  |  | |  | | | | | |  |
|  | | |  |  |  |  |  |  |  | |  | | | | | | |
|  | | | | | | | | | | | |  |  |  |  |  |  |
|  | Respondent: I think that would be the best way to general information to the concerned community not a target a particular individual, to give that kind of result in the trend but that I would imagine some people might want to know more and when they are approached for the research may that is when you can you in the individual results if they were participants | | | | | | | | | | | | | | | |  |
|  |  | | | | | | | | | | | | | | | |  |
|  | | | **Files\\Male\\R-004.male** | | | | | | | | | | | | | |  |
|  |  |  | Yes |  |  |  |  |  |  | |  | | | | | |  |
|  | | |  |  |  |  |  |  |  | |  | | | | | | |
|  | | | | | | | | | | | |  |  |  |  |  |  |
|  | Respondent: you see something that is familiar there it has implications on the family and the family should know and I wonder whether the community comes in this case because the privacy of the family will be exposed but for something that is familial not just a mutant occurring then there a family should know | | | | | | | | | | | | | | | |  |
|  |  | | | | | | | | | | | | | | | |  |
|  | | | **Files\\Male\\R-005.male** | | | | | | | | | | | | | |  |
|  |  |  | Yes |  |  |  |  |  |  | |  | | | | | |  |
|  | | |  |  |  |  |  |  |  | |  | | | | | | |
|  | | | | | | | | | | | |  |  |  |  |  |  |
|  | Interviewer: So those are individual results, when would it be necessary to return aggregate results?  Respondent: I don’t know may be because what I know is that for us we were giving individual….  Interviewer: Would it be okay to return aggregate results?  Respondent: Now let me think about it, I think that needs some kind of preparation of the community, the people who are going to receive the results. Sometimes preparation involves preparing people like sometimes some things are related with stigma, if you are going to give information that is not good, so you need to prepare them | | | | | | | | | | | | | | | |  |
|  |  | | | | | | | | | | | | | | | |  |
|  | | | | | | | | | | | |  |  |  |  |  |  |
|  | Respondent: So the preparation is two way, e.g you yourself, are you ready to give the kind of results you are going to give? And then the question is that what are you going to say? And are you going to give a wrong information, because you need to mind that when you say whatever you say, society tends to interpret things differently, it’s not the same information like when someone comes and tells you, you have this mutation in a given gene let’s say the brac gene, and then someone would say, oh am going to die, the question doesn’t mean that because you have this mutation you are going to die. So you the person who is communicating this kind of information, how are you going to say it? Are you going to give the impression that someone is going to die, and all the breasts cut off? So I think the preparation is two way, preparing the people who are going to receive but even you as a researcher, you must prepare yourself, on how am I going to communicate, what do you think is going to be the response of the community and is it necessary to give that kind of response? | | | | | | | | | | | | | | | |  |
|  |  |  |  |  |  |  |  |  |  |  |  |  |  |  |  |  |  |
|  |  | | | | | | | | | | | | | | | |  |
|  | | | **Files\\Male\\R-006. male** | | | | | | | | | | | | | |  |
|  |  |  | Yes |  |  |  |  |  |  | |  | | | | | |  |
|  | | |  |  |  |  |  |  |  | |  | | | | | | |
|  | | | | | | | | | | | |  |  |  |  |  |  |
|  | if it is a familiar kind of condition, or a kind of condition within a family then you would, it would be necessary to send the message to everyone in the family if it is possible, and if the procedures to do that are clear and well structured, but also in the case of drug resistance for example, if you have drug resistant bug in a particular community you would have to go back in the community and tell everyone that we have these, according to genetics, particular drugs are becoming resistant because of misuse of antibiotics or anything like that. so i think, when the genetic information affects the entire family or affects the general population, i think it will definitely be necessary to return results to the community as an aggregate and not necessarily to individuals. | | | | | | | | | | | | | | | |  |
|  |  | | | | | | | | | | | | | | | |  |
| Reports\\Coding Summary By Code Report | | | | | | | | | | Page 30 of 109 | | | | | | | |
|  | | | | | | | | | | | | | | | | | |
|  | | | **Aggregate** |  | **Classification** |  | **Coverage** |  | **Number Of Coding References** | |  | **Reference Number** |  | **Coded By Initials** |  | **Modified On** |  |
|  | | | **Files\\Male\\R-007 male** | | | | | | | | | | | | | |  |
|  |  |  | Yes |  |  |  |  |  |  | |  | | | | | |  |
|  | | |  |  |  |  |  |  |  | |  | | | | | | |
|  | | | | | | | | | | | |  |  |  |  |  |  |
|  | Respondent: For aggregate results, yes, we can, haa you know, aggregate results are not going to be pointing to any particular individuals, so whatever they implications to the individual, the family and on man exist at that level probably. But then as a community you never know, the community could be one community that shares you know, whatever you are trying to conclude about which might make them labeled in a way. So it is still debatable really. Yeah. | | | | | | | | | | | | | | | |  |
|  |  | | | | | | | | | | | | | | | |  |
|  | | | **Files\\Male\\R-009.male** | | | | | | | | | | | | | |  |
|  |  |  | Yes |  |  |  |  |  |  | |  | | | | | |  |
|  | | |  |  |  |  |  |  |  | |  | | | | | | |
|  | | | | | | | | | | | |  |  |  |  |  |  |
|  | RESPONDENT: it is an interesting thing because most of these data is actually available to the public like now for us in genomics there is a time where we release this data as public information and it can be accessed of course maybe I don’t know how, potentially the community has to access that is anonymized and it is not easy to link the people but of course if the information is about a community you begin to discern some things which can affect any body in that community. So I don’t know we have not thought about returning because there are even some times there is no mechanism of returning this data. how do you give a genetical data base to a person in kabulasoke I don’t see how that happens and then again it might be the framework and feasibility and what would be its benefits visa veer putting it in the public domain | | | | | | | | | | | | | | | |  |
|  |  | | | | | | | | | | | | | | | |  |
|  | | | **Files\\Male\\R-010. male** | | | | | | | | | | | | | |  |
|  |  |  | Yes |  |  |  |  |  |  | |  | | | | | |  |
|  | | |  |  |  |  |  |  |  | |  | | | | | | |
|  | | | | | | | | | | | |  |  |  |  |  |  |
|  | INTERVIEWER: have you ever returned aggregate results?  RESPONDENT: Like all results?  INTERVIEWER: Like you go to a community, you get some findings from your study but as you are returning you don’t go to individuals…  RESPONDENT: ideally that is very important. I have not done before because a lot of the studies I am involved in are not larger scale community studies except the CafGEN study actually it’s a very big study but we do a lot of engagement of community usually before we start the study we engage communities and even at the end of the study we engage communities because it is a requirement. Many times a lot of funders are very advanced, they demand that there are mechanisms for dissemination in which we engage communities as well. But apparently NO, we have not engaged in anything like that. Courts of law at times demand this kind of evidence even if there is no study eg; issues to do with paternity. | | | | | | | | | | | | | | | |  |
|  |  |  |  |  |  |  |  |  |  |  |  |  |  |  |  |  |  |
|  |  | | | | | | | | | | | | | | | |  |
|  | | | | | | | | | | | |  |  |  |  |  |  |
|  | INTERVIEWER: Like you go to a community, you get some findings from your study but as you are returning you don’t go to individuals…  RESPONDENT: ideally that is very important. I have not done before because a lot of the studies I am involved in are not larger scale community studies except the CafGEN study actually it’s a very big study but we do a lot of engagement of community usually before we start the study we engage communities and even at the end of the study we engage communities because it is a requirement. Many times a lot of funders are very advanced, they demand that there are mechanisms for dissemination in which we engage communities as well. But apparently NO, we have not engaged in anything like that. Courts of law at times demand this kind of evidence even if there is no study eg; issues to do with paternity. | | | | | | | | | | | | | | | |  |
|  |  |  |  |  |  |  |  |  |  |  |  |  |  |  |  |  |  |
|  |  | | | | | | | | | | | | | | | |  |
|  | | | **Files\\Male\\R-012.male** | | | | | | | | | | | | | |  |
|  |  |  | Yes |  |  |  |  |  |  | |  | | | | | |  |
|  | | |  |  |  |  |  |  |  | |  | | | | | | |
|  | | | | | | | | | | | |  |  |  |  |  |  |
|  | RESPONDENT: I don’t think I would return results but the generalized results yes, not the individual results in that way yes | | | | | | | | | | | | | | | |  |
|  |  | | | | | | | | | | | | | | | |  |
| Reports\\Coding Summary By Code Report | | | | | | | | | | Page 31 of 109 | | | | | | | |
|  | | | | | | | | | | | | | | | | | |
|  | | | **Aggregate** |  | **Classification** |  | **Coverage** |  | **Number Of Coding References** | |  | **Reference Number** |  | **Coded By Initials** |  | **Modified On** |  |
|  | | | **Files\\Male\\R-013.male** | | | | | | | | | | | | | |  |
|  |  |  | Yes |  |  |  |  |  |  | |  | | | | | |  |
|  | | |  |  |  |  |  |  |  | |  | | | | | | |
|  | | | | | | | | | | | |  |  |  |  |  |  |
|  | RESPONDENT: I think it is important but it still depends on the setting that you’re in because aggregate results are from what circumstance of Uganda | | | | | | | | | | | | | | | |  |
|  |  | | | | | | | | | | | | | | | |  |
|  | | | | | | | | | | | |  |  |  |  |  |  |
|  | RESPONDENT: you see that information is based disseminated in like a conference or in scientific community here and all that but individual you will give the patients the results individually but the other aggregate information can always be, I think it shows the importance of disseminating your findings CMEs, in conferences in workshops and all that to increase the board of knowledge to the community basically to attract | | | | | | | | | | | | | | | |  |
|  |  | | | | | | | | | | | | | | | |  |
|  | | | **Files\\Male\\R-016. male** | | | | | | | | | | | | | |  |
|  |  |  | Yes |  |  |  |  |  |  | |  | | | | | |  |
|  | | |  |  |  |  |  |  |  | |  | | | | | | |
|  | | | | | | | | | | | |  |  |  |  |  |  |
|  | RESPONDENT: so, at the end of the study we will, you see the study is such hat we have genetic samples DNA samples from about five countries and then we have each country having like five sites so you have 40,000 DNA samples for you to make sense out of DNA samples genetic research you need 10,000s of results so reporting or knowing your results alone is not something that helps you directly because it is of no benefit you cannot use those results to do anything, the only way we can use those results is if we have that information from those 40,000 participants collected together I hope that helps | | | | | | | | | | | | | | | |  |
|  |  | | | | | | | | | | | | | | | |  |
|  | | | | | | | | | | | |  |  |  |  |  |  |
|  | RESPONDENT: yes, you report aggregate results, I think that is much better | | | | | | | | | | | | | | | |  |
|  |  | | | | | | | | | | | | | | | |  |
|  | | | | | | | | | | | |  |  |  |  |  |  |
|  | INTERVIEWER: are there any conditions, what are conditions in place for you to report such results?  RESPONDENT: to who?  INTERVIEWER: to those communities to inform the bigger communities about their findings  RESPONDENT: we are doing something called genomic wide association so you sequence the genome of a person you say you have 23 chromosomes or whatever it is may be on chromosome six there is this chromosome eight there is this chromosome nine there is this, that information has very little benefit to lay people that information can only be used by scientists in the future for example to say I think schozophrenia as a disease is found on chromosome six the short arm of chromosome six or something like that now when they are making medications in the future they can now start targeting those molecules so that finding per say is not helpful to even me myself who is a scientist, I don’t know whether that helps it like knowing the distance between here and moon you can know it but what you’re going to use it for | | | | | | | | | | | | | | | |  |
|  |  |  |  |  |  |  |  |  |  |  |  |  |  |  |  |  |  |
|  |  | | | | | | | | | | | | | | | |  |
|  | | | **Files\\Male\\R-017. male** | | | | | | | | | | | | | |  |
|  |  |  | Yes |  |  |  |  |  |  | |  | | | | | |  |
|  | | |  |  |  |  |  |  |  | |  | | | | | | |
|  | | | | | | | | | | | |  |  |  |  |  |  |
|  | Respondent: I don’t think it is okay, it is subjective and at some point, you might be marginalized for being part of the incidental findings | | | | | | | | | | | | | | | |  |
|  |  | | | | | | | | | | | | | | | |  |
|  | | | **Files\\Male\\R-022. male** | | | | | | | | | | | | | |  |
|  |  |  | Yes |  |  |  |  |  |  | |  | | | | | |  |
|  | | |  |  |  |  |  |  |  | |  | | | | | | |
|  | | | | | | | | | | | |  |  |  |  |  |  |
|  | RESPONDENT: it is a yes and no, it really depends on what the question is. So, it is a case by case. | | | | | | | | | | | | | | | |  |
|  |  | | | | | | | | | | | | | | | |  |
| Reports\\Coding Summary By Code Report | | | | | | | | | | Page 32 of 109 | | | | | | | |
|  | | | | | | | | | | | | | | | | | |
|  | | | **Aggregate** |  | **Classification** |  | **Coverage** |  | **Number Of Coding References** | |  | **Reference Number** |  | **Coded By Initials** |  | **Modified On** |  |
|  | | | | | | | | | | | | | | | | | |
|  | | | | | | | | | | | |  |  |  |  |  |  |
|  | INTERVIEWER: let’s try to look at both cases; when would it be necessary to?  RESPONDENT: if it really has a big implication on public health that needs to be reported but sometimes it may not have a direct benefit. So, you weigh the risks and benefits.  INTERVIEWER: so, if it doesn’t have a public health implication you may not report?  RESPONDENT: no  INTERVIEWER: its not an obligation to report?  RESPONDENT: no | | | | | | | | | | | | | | | |  |
|  |  |  |  |  |  |  |  |  |  |  |  |  |  |  |  |  |  |
|  |  | | | | | | | | | | | | | | | |  |
|  | | | **Files\\Male\\R-025 male..** | | | | | | | | | | | | | |  |
|  |  |  | Yes |  |  |  |  |  |  | |  | | | | | |  |
|  | | |  |  |  |  |  |  |  | |  | | | | | | |
|  | | | | | | | | | | | |  |  |  |  |  |  |
|  | Interviewer: So, when is it necessary to return aggregate results?  Respondent: Once the study has done the analysis, maybe the other this is that we don’t have the capacity in country to do the analysis. Also the study somehow, like this one was under estimate of the amount of money involved in doing the genetic analysis, so I think once the overall genetic analysis is out you just say, unfortunately for communication you see if it comes before you give the feed back to the community, so at that stage when its clear view, the communications are out, then we discuss with the community but for the CAB we do discuss preliminary results | | | | | | | | | | | | | | | |  |
|  |  | | | | | | | | | | | | | | | |  |
|  | **Nodes\\Return of results\\Opinion on return of results\Aggregate results\Aggregate results** | | | | | | | | | | | | | | | |  |
|  | | **Document** | | | | | | | | | | | | | | |  |
|  | | | **Files\\Male\\R-002. male** | | | | | | | | | | | | | |  |
|  |  |  | No |  |  |  |  |  |  | |  | | | | | |  |
|  | | |  |  |  |  |  |  |  | |  | | | | | | |
|  | | | | | | | | | | | |  |  |  |  |  |  |
|  | Respondent: I think that would be the best way to general information to the concerned community not a target a particular individual, to give that kind of result in the trend but that I would imagine some people might want to know more and when they are approached for the research may that is when you can you in the individual results if they were participants | | | | | | | | | | | | | | | |  |
|  |  | | | | | | | | | | | | | | | |  |
|  | | | **Files\\Male\\R-005.male** | | | | | | | | | | | | | |  |
|  |  |  | No |  |  |  |  |  |  | |  | | | | | |  |
|  | | |  |  |  |  |  |  |  | |  | | | | | | |
|  | | | | | | | | | | | |  |  |  |  |  |  |
|  | Interviewer: So those are individual results, when would it be necessary to return aggregate results?  Respondent: I don’t know may be because what I know is that for us we were giving individual….  Interviewer: Would it be okay to return aggregate results?  Respondent: Now let me think about it, I think that needs some kind of preparation of the community, the people who are going to receive the results. Sometimes preparation involves preparing people like sometimes some things are related with stigma, if you are going to give information that is not good, so you need to prepare them | | | | | | | | | | | | | | | |  |
|  |  | | | | | | | | | | | | | | | |  |
| Reports\\Coding Summary By Code Report | | | | | | | | | | Page 33 of 109 | | | | | | | |
|  | | | | | | | | | | | | | | | | | |
|  | | | **Aggregate** |  | **Classification** |  | **Coverage** |  | **Number Of Coding References** | |  | **Reference Number** |  | **Coded By Initials** |  | **Modified On** |  |
|  | | | **Files\\Male\\R-006. male** | | | | | | | | | | | | | |  |
|  |  |  | No |  |  |  |  |  |  | |  | | | | | |  |
|  | | |  |  |  |  |  |  |  | |  | | | | | | |
|  | | | | | | | | | | | |  |  |  |  |  |  |
|  | if it is a familiar kind of condition, or a kind of condition within a family then you would, it would be necessary to send the message to everyone in the family if it is possible, and if the procedures to do that are clear and well structured, but also in the case of drug resistance for example, if you have drug resistant bug in a particular community you would have to go back in the community and tell everyone that we have these, according to genetics, particular drugs are becoming resistant because of misuse of antibiotics or anything like that. so i think, when the genetic information affects the entire family or affects the general population, i think it will definitely be necessary to return results to the community as an aggregate and not necessarily to individuals. | | | | | | | | | | | | | | | |  |
|  |  | | | | | | | | | | | | | | | |  |
|  | | | **Files\\Male\\R-007 male** | | | | | | | | | | | | | |  |
|  |  |  | No |  |  |  |  |  |  | |  | | | | | |  |
|  | | |  |  |  |  |  |  |  | |  | | | | | | |
|  | | | | | | | | | | | |  |  |  |  |  |  |
|  | Respondent: For aggregate results, yes, we can, haa you know, aggregate results are not going to be pointing to any particular individuals, so whatever they implications to the individual, the family and on man exist at that level probably. But then as a community you never know, the community could be one community that shares you know, whatever you are trying to conclude about which might make them labeled in a way. So it is still debatable really. Yeah. | | | | | | | | | | | | | | | |  |
|  |  | | | | | | | | | | | | | | | |  |
|  | | | **Files\\Male\\R-010. male** | | | | | | | | | | | | | |  |
|  |  |  | No |  |  |  |  |  |  | |  | | | | | |  |
|  | | |  |  |  |  |  |  |  | |  | | | | | | |
|  | | | | | | | | | | | |  |  |  |  |  |  |
|  | INTERVIEWER: Like you go to a community, you get some findings from your study but as you are returning you don’t go to individuals…  RESPONDENT: ideally that is very important. I have not done before because a lot of the studies I am involved in are not larger scale community studies except the CafGEN study actually it’s a very big study but we do a lot of engagement of community usually before we start the study we engage communities and even at the end of the study we engage communities because it is a requirement. Many times a lot of funders are very advanced, they demand that there are mechanisms for dissemination in which we engage communities as well. But apparently NO, we have not engaged in anything like that. Courts of law at times demand this kind of evidence even if there is no study eg; issues to do with paternity. | | | | | | | | | | | | | | | |  |
|  |  |  |  |  |  |  |  |  |  |  |  |  |  |  |  |  |  |
|  |  | | | | | | | | | | | | | | | |  |
|  | | | **Files\\Male\\R-013.male** | | | | | | | | | | | | | |  |
|  |  |  | No |  |  |  |  |  |  | |  | | | | | |  |
|  | | |  |  |  |  |  |  |  | |  | | | | | | |
|  | | | | | | | | | | | |  |  |  |  |  |  |
|  | RESPONDENT: I think it is important but it still depends on the setting that you’re in because aggregate results are from what circumstance of Uganda | | | | | | | | | | | | | | | |  |
|  |  | | | | | | | | | | | | | | | |  |
|  | | | | | | | | | | | |  |  |  |  |  |  |
|  | RESPONDENT: you see that information is based disseminated in like a conference or in scientific community here and all that but individual you will give the patients the results individually but the other aggregate information can always be, I think it shows the importance of disseminating your findings CMEs, in conferences in workshops and all that to increase the board of knowledge to the community basically to attract | | | | | | | | | | | | | | | |  |
|  |  | | | | | | | | | | | | | | | |  |
|  | | | | | | | | | | | | | | | | | |
| Reports\\Coding Summary By Code Report | | | | | | | | | | Page 34 of 109 | | | | | | | |
|  | | | | | | | | | | | | | | | | | |
|  | | | **Aggregate** |  | **Classification** |  | **Coverage** |  | **Number Of Coding References** | |  | **Reference Number** |  | **Coded By Initials** |  | **Modified On** |  |
|  | | | **Files\\Male\\R-016. male** | | | | | | | | | | | | | |  |
|  |  |  | No |  |  |  |  |  |  | |  | | | | | |  |
|  | | |  |  |  |  |  |  |  | |  | | | | | | |
|  | | | | | | | | | | | |  |  |  |  |  |  |
|  | INTERVIEWER: are there any conditions, what are conditions in place for you to report such results?  RESPONDENT: to who?  INTERVIEWER: to those communities to inform the bigger communities about their findings  RESPONDENT: we are doing something called genomic wide association so you sequence the genome of a person you say you have 23 chromosomes or whatever it is may be on chromosome six there is this chromosome eight there is this chromosome nine there is this, that information has very little benefit to lay people that information can only be used by scientists in the future for example to say I think schizophrenia as a disease is found on chromosome six the short arm of chromosome six or something like that now when they are making medications in the future they can now start targeting those molecules so that finding per say is not helpful to even me myself who is a scientist, I don’t know whether that helps it like knowing the distance between here and moon you can know it but what you’re going to use it for | | | | | | | | | | | | | | | |  |
|  |  |  |  |  |  |  |  |  |  |  |  |  |  |  |  |  |  |
|  |  | | | | | | | | | | | | | | | |  |
|  | | | **Files\\Male\\R-022. male** | | | | | | | | | | | | | |  |
|  |  |  | No |  |  |  |  |  |  | |  | | | | | |  |
|  | | |  |  |  |  |  |  |  | |  | | | | | | |
|  | | | | | | | | | | | |  |  |  |  |  |  |
|  | INTERVIEWER: let’s try to look at both cases; when would it be necessary to?  RESPONDENT: if it really has a big implication on public health that needs to be reported but sometimes it may not have a direct benefit. So, you weigh the risks and benefits.  INTERVIEWER: so, if it doesn’t have a public health implication you may not report?  RESPONDENT: no  INTERVIEWER: its not an obligation to report?  RESPONDENT: no | | | | | | | | | | | | | | | |  |
|  |  |  |  |  |  |  |  |  |  |  |  |  |  |  |  |  |  |
|  |  | | | | | | | | | | | | | | | |  |
|  | **Nodes\\Return of results\\Opinion on return of results\Depends** | | | | | | | | | | | | | | | |  |
|  | | **Document** | | | | | | | | | | | | | | |  |
|  | | | **Files\\Female\\R026 female** | | | | | | | | | | | | | |  |
|  |  |  | No |  |  |  |  |  |  | |  | | | | | |  |
|  | | |  |  |  |  |  |  |  | |  | | | | | | |
|  | | | | | | | | | | | |  |  |  |  |  |  |
|  | INTERVIEWER: So, you think it is not necessary to return such results to participants?  RESPONDENT: They are early and they don’t mean much to either the community or the individual, and it would be much better if we were at stage four research where we now show that people with this gene are more likely to get disease and then we can look at how we translate it in day today life but when they are early ones that on chromosome 6 we found three genes related, and even when I put it in simple language, we all know nothing much from now, we are doing more research | | | | | | | | | | | | | | | |  |
|  |  | | | | | | | | | | | | | | | |  |
|  | | | | | | | | | | | | | | | | | |
| Reports\\Coding Summary By Code Report | | | | | | | | | | Page 35 of 109 | | | | | | | |
|  | | | | | | | | | | | | | | | | | |
|  | | | **Aggregate** |  | **Classification** |  | **Coverage** |  | **Number Of Coding References** | |  | **Reference Number** |  | **Coded By Initials** |  | **Modified On** |  |
|  | | | | | | | | | | | | | | | | | |
|  | | | | | | | | | | | |  |  |  |  |  |  |
|  | INTERVIEWER: So, if nothing can be done in that situation, you would recommend not to?  RESPONDENT: Again, it depends on when the disease surfaces, if a disease is common around 40 years, its is good for people to be aware that there is a disease that needs to shape in, it is common around 40 years, has anybody ever got it? And they say, oh yes, we once had an uncle, it is common in men and women and they’ve gotten this chance so be aware. But if you say you get dementia at 70 and it is common, then I would leave the person alone because I would say it is slow, it gradual, we can’t do anything but if we can do something that is different, so again there are many factors. It come at 40 years, you have the gene for getting prostate or breast cancer, I advise get regular tests, get an operation, so it depends on the situation | | | | | | | | | | | | | | | |  |
|  |  |  |  |  |  |  |  |  |  |  |  |  |  |  |  |  |  |
|  |  | | | | | | | | | | | | | | | |  |
|  | | | **Files\\Female\\R027.female** | | | | | | | | | | | | | |  |
|  |  |  | No |  |  |  |  |  |  | |  | | | | | |  |
|  | | |  |  |  |  |  |  |  | |  | | | | | | |
|  | | | | | | | | | | | |  |  |  |  |  |  |
|  | INTERVIEWER: But generally, do you think it is necessary to return genetics genomics results?  RESPONDENT: It depends, there are some things that may not be useful to the patient then there are some that might be useful so if the group of people investigated to the some is useful for them to know then yes, you might be looking for some strange thing there that doesn’t really make sense and doesn’t matter to them | | | | | | | | | | | | | | | |  |
|  |  | | | | | | | | | | | | | | | |  |
|  | | | **Files\\Female\\R028.female** | | | | | | | | | | | | | |  |
|  |  |  | No |  |  |  |  |  |  | |  | | | | | |  |
|  | | |  |  |  |  |  |  |  | |  | | | | | | |
|  | | | | | | | | | | | |  |  |  |  |  |  |
|  | RESPONDENT: I think it depends on what the research is about, what diseases are involved, because most of the information we are getting now is not definite. It just helps us to understand this disease better. It does not mean that even if we found these high risky genes that they are necessarily a problem because there are many women with them and they don’t have a problem, so why am saying it depends on the disease is because there some now known genetic variants which are almost sure deal, you have them you are at very increased risk of that disease, I think like in cancer of the breast. Some of those it may be necessary to tell someone because there is something they can do about it, but in this case, there is even nothing that you can do about it even if you know your genetic variants at that time, so it makes no difference to know | | | | | | | | | | | | | | | |  |
|  |  |  |  |  |  |  |  |  |  |  |  |  |  |  |  |  |  |
|  |  | | | | | | | | | | | | | | | |  |
|  | | | **Files\\Male\\R-001 male** | | | | | | | | | | | | | |  |
|  |  |  | No |  |  |  |  |  |  | |  | | | | | |  |
|  | | |  |  |  |  |  |  |  | |  | | | | | | |
|  | | | | | | | | | | | |  |  |  |  |  |  |
|  | I think it's a dilemma because sometimes you may report back something and it gets misunderstood, but usually it would be important if that affects the way you are going to manage the Patient different from another Patient, its important that you report and say, This is what we found and we think its going to help you if we do this for you. | | | | | | | | | | | | | | | |  |
|  |  | | | | | | | | | | | | | | | |  |
|  | | | **Files\\Male\\R-006. male** | | | | | | | | | | | | | |  |
|  |  |  | No |  |  |  |  |  |  | |  | | | | | |  |
|  | | |  |  |  |  |  |  |  | |  | | | | | | |
|  | | | | | | | | | | | |  |  |  |  |  |  |
|  | RESPONDENT:  Yes, and to their families really. Direct benefits to the patients but also to their families. | | | | | | | | | | | | | | | |  |
|  |  | | | | | | | | | | | | | | | |  |
| Reports\\Coding Summary By Code Report | | | | | | | | | | Page 36 of 109 | | | | | | | |
|  | | | | | | | | | | | | | | | | | |
|  | | | **Aggregate** |  | **Classification** |  | **Coverage** |  | **Number Of Coding References** | |  | **Reference Number** |  | **Coded By Initials** |  | **Modified On** |  |
|  | | | **Files\\Male\\R-009.male** | | | | | | | | | | | | | |  |
|  |  |  | No |  |  |  |  |  |  | |  | | | | | |  |
|  | | |  |  |  |  |  |  |  | |  | | | | | | |
|  | | | | | | | | | | | |  |  |  |  |  |  |
|  | INTERVIEWER: what if nothing can be done you don’t report?  RESPONDENT: those are the sensitivities we have to discuss with the community advisory board because you may think some people might be happy to know that is not going to survive in the next one year, another person might say don’t tell me that kind of information so I think it might you | | | | | | | | | | | | | | | |  |
|  |  | | | | | | | | | | | | | | | |  |
|  | | | **Files\\Male\\R-010. male** | | | | | | | | | | | | | |  |
|  |  |  | No |  |  |  |  |  |  | |  | | | | | |  |
|  | | |  |  |  |  |  |  |  | |  | | | | | | |
|  | | | | | | | | | | | |  |  |  |  |  |  |
|  | Let’s take a scenario; person has not consented but the matter is a public health concern, you are doing a whole genome sequence and then you find that this person is actually infected with Ebola, you must inform not only the patient but also the authorities, in fact you must follow up this patient. So, at the end of the day, I think the decision to return results is very contextual, several scenarios should be remolded and it’s important that the participants understand these scenarios because if a participant just says “I don’t want to know”. One of the big studies in genomics, there are what we call family trials, I use them a lot because when we get the father, the mother and then the child and then you wake up and this child is not the father’s child, what do you do! | | | | | | | | | | | | | | | |  |
|  |  | | | | | | | | | | | | | | | |  |
|  | | | **Files\\Male\\R-011.male** | | | | | | | | | | | | | |  |
|  |  |  | No |  |  |  |  |  |  | |  | | | | | |  |
|  | | |  |  |  |  |  |  |  | |  | | | | | | |
|  | | | | | | | | | | | |  |  |  |  |  |  |
|  | Incidental findings; I think it just depends, i think a lot of these things really, there is always a REC that reviews to see if what you are doing is ethical, if really there are some findings, i guess its not me as a research to decide if should release it, there’s somebody to decide the potential outcome of release of such results. Then there some communities that participate in genetic research, maybe this study is concerned with finding out their ancestry and migration patterns, and then somewhere along the way they find out oh actually what we know about this population, based on the genetic information is different from what is known, Group A ,occupied this land before Group B. Reporting such information to this community creates now land conflicts, so reporting such things is not for us, i wouldn’t say it’s for me to do, its really, i would say we have to be sensitive about the societies that are there. | | | | | | | | | | | | | | | |  |
|  |  |  |  |  |  |  |  |  |  |  |  |  |  |  |  |  |  |
|  |  | | | | | | | | | | | | | | | |  |
|  | | | **Files\\Male\\R-013.male** | | | | | | | | | | | | | |  |
|  |  |  | No |  |  |  |  |  |  | |  | | | | | |  |
|  | | |  |  |  |  |  |  |  | |  | | | | | | |
|  | | | | | | | | | | | |  |  |  |  |  |  |
|  | RESPONDENT: I think it still depends, you see one thing is that when you are doing genomics work and basic science work there is work that is not really directly important to the patient but this work is the science that is used to build on, on to many things let’s say developing a vaccine basically its more into building a scientific board of knowledge so most of this kind of work is very hard to really report back to the patient because there is no direct link of its importance to the patient. so I think it still depends for example on the kind of work you are doing because as you report this information to patients you only need to think about the so what as in yes you found this gene so what  INTERVIEWER: if you | | | | | | | | | | | | | | | |  |
|  |  | | | | | | | | | | | | | | | |  |
|  | | | | | | | | | | | | | | | | | |
| Reports\\Coding Summary By Code Report | | | | | | | | | | Page 37 of 109 | | | | | | | |
|  | | | | | | | | | | | | | | | | | |
|  | | | **Aggregate** |  | **Classification** |  | **Coverage** |  | **Number Of Coding References** | |  | **Reference Number** |  | **Coded By Initials** |  | **Modified On** |  |
|  | | | **Files\\Male\\R-017. male** | | | | | | | | | | | | | |  |
|  |  |  | No |  |  |  |  |  |  | |  | | | | | |  |
|  | | |  |  |  |  |  |  |  | |  | | | | | | |
|  | | | | | | | | | | | |  |  |  |  |  |  |
|  | Interviewer: And do you think all incidental findings must be reported?  Respondent: Depending on the circumstances because incidental findings maybe one of those things that are relevant maybe, its not a life and death, it is an incidental finding relevant to the research but not to the individual, but it is relevant to the wider scientific community that is investigate that | | | | | | | | | | | | | | | |  |
|  |  | | | | | | | | | | | | | | | |  |
|  | | | | | | | | | | | |  |  |  |  |  |  |
|  | Respondent: Yes, that’s why I said you will have to categorize the incidental finding, in terms of is it a life and death, fatal in that if this person doesn’t know, they are at risk. Such finding when you get them, you also weigh the risks, because there is putting a person on a red flag yet it is your suspicion based on your intuition, but then you must first monitor, assess, inquire from other researchers and also may be interface with the clinicians until you are really confident and sure and you know beyond reasonable doubt that if I don’t tell this individual, this will happen. So, you have to give it some bit of reasoning and consultation  Interviewer: Okay | | | | | | | | | | | | | | | |  |
|  |  | | | | | | | | | | | | | | | |  |
|  | **Nodes\\Return of results\\Opinion on return of results\Incidental findings** | | | | | | | | | | | | | | | |  |
|  | | **Document** | | | | | | | | | | | | | | |  |
|  | | | **Files\\Female\\R-003.female** | | | | | | | | | | | | | |  |
|  |  |  | No |  |  |  |  |  |  | |  | | | | | |  |
|  | | |  |  |  |  |  |  |  | |  | | | | | | |
|  | | | | | | | | | | | |  |  |  |  |  |  |
|  | INTERVIEWER: in case of incidental findings, how do you handle such results? Suppose you went for sickle cells you got it, its easier to handle because you have already obtained informed consent and then telling people about it but sometimes you get findings that were not in the target of our research, how do you handle those ones?  RESPONDENT: the unexpected findings, | | | | | | | | | | | | | | | |  |
|  |  | | | | | | | | | | | | | | | |  |
|  | | | | | | | | | | | |  |  |  |  |  |  |
|  | INTERVIEWER: would you then propose that during the informed consent it should also add a component of incidental findings  RESPONDENT: yes | | | | | | | | | | | | | | | |  |
|  |  | | | | | | | | | | | | | | | |  |
|  | | | **Files\\Female\\R026 female** | | | | | | | | | | | | | |  |
|  |  |  | No |  |  |  |  |  |  | |  | | | | | |  |
|  | | |  |  |  |  |  |  |  | |  | | | | | | |
|  | | | | | | | | | | | |  |  |  |  |  |  |
|  | INTERVIEWER: So, what has been the outcome so far from that communication?  RESPONDENT: Agaain I didn’t get that feedback | | | | | | | | | | | | | | | |  |
|  |  | | | | | | | | | | | | | | | |  |
|  | | | | | | | | | | | | | | | | | |
| Reports\\Coding Summary By Code Report | | | | | | | | | | Page 38 of 109 | | | | | | | |
|  | | | | | | | | | | | | | | | | | |
|  | | | **Aggregate** |  | **Classification** |  | **Coverage** |  | **Number Of Coding References** | |  | **Reference Number** |  | **Coded By Initials** |  | **Modified On** |  |
|  | | | | | | | | | | | | | | | | | |
|  | | | | | | | | | | | |  |  |  |  |  |  |
|  | RESPONDENT: Okay let’s say theoretically, that in the study I look at these genes and I look at let’s say the gene is common in TB people but also found in people with colon cancer then I say, haaa 10% of the population had a gene that is not only in TB but that gene is also linked to colon cancer. Now one would think a lot of how you bring out that information, because having a gene doesn’t mean one will have the disease but then knowing that you have the gene and the risk, one can have preventive measures. Now if feel that this will impact on a person, I would divulge that information to an individual and to the family, that in your family there were six participants and 3 had a problem, and that information should not be given by a home visitor, it should be by the physician/doctor/PI because it is serious stuff and again you should come up with a reason for bringing up the information. So, we suggest in your family people should be tested for colon cancer by doing these tests on a regular basis or we found this gene for breast cancer so in your family people should test for breast cancer on a regular basis. Then there are some genes where, yes, I find a gene for people getting dementia at 60/70 years, I would wonder what is the probability that that person would get dementia and what will the effect be? Will it be to that person’s advantage? Here is a person, 60 years working, enjoying their life and then you tell them by the way you have a gene for dementia, aren’t I going to make that person’s life worse? So, I think one has to balance what it means and if it is a family it gets even it gets even more remarkable, especially if nothing can be done in that situation | | | | | | | | | | | | | | | |  |
|  |  |  |  |  |  |  |  |  |  |  |  |  |  |  |  |  |  |
|  |  | | | | | | | | | | | | | | | |  |
|  | | | **Files\\Female\\R027.female** | | | | | | | | | | | | | |  |
|  |  |  | No |  |  |  |  |  |  | |  | | | | | |  |
|  | | |  |  |  |  |  |  |  | |  | | | | | | |
|  | | | | | | | | | | | |  |  |  |  |  |  |
|  | INTERVIEWER: Now, for example in case of incidental findings, how should they be handled?  RESPONDENT: Incidental, I don’t know what you are talking about because we don’t have that research where we are talking about those things that affect your child or your community.  INTERVIEWER: No, incidental findings are things that maybe you didn’t anticipate but along the way you realize or you find them out but then they are not addressing any of your basis but you feel it should be important. For example; I have given an example if you are doing something to do with sickle cell you may have to do with paternity but then along the way you realize that may be that child does not really belong to that family, it is not exactly what you were looking for but it came along the way and then you found out that this child doesn’t belong to this family. So, what would you advise, do you advise to communicate to such a family? | | | | | | | | | | | | | | | |  |
|  |  |  |  |  |  |  |  |  |  |  |  |  |  |  |  |  |  |
|  |  | | | | | | | | | | | | | | | |  |
|  | | | | | | | | | | | |  |  |  |  |  |  |
|  | INTERVIEWER: I think that is kind of anticipated in paternity however, if we were to look at things that are may be related to disease like may be someone prone to a specific disease that for you, you are dealing with another line of disease then you realize that this gene or this person might get another condition so in that case it is not something you anticipated because it is not obvious but it can cover.  RESPONDENT: But in every research you know that there are somethings that can come up that are not anticipated so I think there is a way that you can cover and ask your patients if they will find anything that is not anticipated, would you like to be informed? Because not every one would want to know that they are prone to breast cancer, you might have gone for cervical cancer screening and they may find a gene that you are prone to certain death like these people who just collapse, they don’t want to know, they want to enjoy life and they might make the quality of their life worse and say that I would still want to know at the beginning if there is anything which you would like to know. Or if I find it, I can call and say that I found some results which I need to inform you of the results of the genotype that I don’t think there is some information that some people are better off without. I will give you an example; people who go to test and see if their babies have abnormalities, they do that a lot either to terminate or to continue. In Uganda here, we don’t have that culture of terminating the baby because you hear the baby has Downs syndrome or whatever so if you do that test usually the probability is fifty and you can have Downs syndrome or that, so if a doctor tells you, you have a 50% chance of giving birth to a child with Downs syndrome, you can terminate the pregnancy but again there is a 50% chance of not having it. But for us we already have the knowledge and you might even terminate because of you don’t want to go down that road then you terminate when actually the child was normal so I can’t just dish out information, I need to tell you that there is this test, you can find out. Do you want to know? | | | | | | | | | | | | | | | |  |
|  |  |  |  |  |  |  |  |  |  |  |  |  |  |  |  |  |  |
|  |  |  |  |  |  |  |  |  |  |  |  |  |  |  |  |  |  |
|  |  | | | | | | | | | | | | | | | |  |
|  | | | | | | | | | | | | | | | | | |
|  | | | | | | | | | | | | | | | | | |
| Reports\\Coding Summary By Code Report | | | | | | | | | | Page 39 of 109 | | | | | | | |
|  | | | | | | | | | | | | | | | | | |
|  | | | **Aggregate** |  | **Classification** |  | **Coverage** |  | **Number Of Coding References** | |  | **Reference Number** |  | **Coded By Initials** |  | **Modified On** |  |
|  | | | **Files\\Female\\R029 female** | | | | | | | | | | | | | |  |
|  |  |  | No |  |  |  |  |  |  | |  | | | | | |  |
|  | | |  |  |  |  |  |  |  | |  | | | | | | |
|  | | | | | | | | | | | |  |  |  |  |  |  |
|  | INTERVIEWER: Let me give you an example, like the sickle cell study that was carried out in mulago, and during the study they realized that the people they thought were fathers were not actually the fathers because of the tests conducted, it was not the primary motive of the study. Even if you kept quiet as the researcher, these would have no impact because the father and mother are already living together, but in your heart as researcher, you know that the “father” is not the biological one. Would you go ahead and return such results?  RESPONDENT: well they are incidental, and these people are expecting such results in such a scenario, there repercussions of such a scenario and of course we don’t want to brake people’s families as researchers and you don’t want to spoil your participant’s lives. This is something critical but how about if include in the consent form that incase of any incidental findings may be can we return them to you if they consent to that, maybe there is a way you can approach it and may be if I had not put it in the consent form I don’t know what I would do about that. | | | | | | | | | | | | | | | |  |
|  |  |  |  |  |  |  |  |  |  |  |  |  |  |  |  |  |  |
|  |  | | | | | | | | | | | | | | | |  |
|  | | | **Files\\Female\\R030.female** | | | | | | | | | | | | | |  |
|  |  |  | No |  |  |  |  |  |  | |  | | | | | |  |
|  | | |  |  |  |  |  |  |  | |  | | | | | | |
|  | | | | | | | | | | | |  |  |  |  |  |  |
|  | INTERVIEWER: In case there are incidental findings, how do you handle them?  RESPONDENT: If there are incidental findings, so what exactly do you mean? Like the lab goes in and they find something they didn’t expect. | | | | | | | | | | | | | | | |  |
|  |  | | | | | | | | | | | | | | | |  |
|  | | | **Files\\Male\\R-001 male** | | | | | | | | | | | | | |  |
|  |  |  | No |  |  |  |  |  |  | |  | | | | | |  |
|  | | |  |  |  |  |  |  |  | |  | | | | | | |
|  | | | | | | | | | | | |  |  |  |  |  |  |
|  | Incidental fndings are also very difficult at some point because you didn't go for them but you have found them | | | | | | | | | | | | | | | |  |
|  |  | | | | | | | | | | | | | | | |  |
|  | | | **Files\\Male\\R-006. male** | | | | | | | | | | | | | |  |
|  |  |  | No |  |  |  |  |  |  | |  | | | | | |  |
|  | | |  |  |  |  |  |  |  | |  | | | | | | |
|  | | | | | | | | | | | |  |  |  |  |  |  |
|  | That is very interesting but complex question because if you found out something in your research and this is not necessarily. So it depends if what you are finding out in your research a particular condition you were not looking for, and you think it is linked to the ill health of the individual or the ill health of the community, I think it would be worthwhile to go back to discuss with especially the ethics bodies how such results would have to be disseminated because, this is not something you set out to do, and the ethical bodies didn't expect you to look for them, these things you found them incidentally. i think it would be better to go back to the ethics bodies and discuss how this information would have to go back because it would be beneficial to the individual and to the community. | | | | | | | | | | | | | | | |  |
|  |  | | | | | | | | | | | | | | | |  |
|  | | | | | | | | | | | | | | | | | |
|  | | | | | | | | | | | | | | | | | |
| Reports\\Coding Summary By Code Report | | | | | | | | | | Page 40 of 109 | | | | | | | |
|  | | | | | | | | | | | | | | | | | |
|  | | | **Aggregate** |  | **Classification** |  | **Coverage** |  | **Number Of Coding References** | |  | **Reference Number** |  | **Coded By Initials** |  | **Modified On** |  |
|  | | | **Files\\Male\\R-007 male** | | | | | | | | | | | | | |  |
|  |  |  | No |  |  |  |  |  |  | |  | | | | | |  |
|  | | |  |  |  |  |  |  |  | |  | | | | | | |
|  | | | | | | | | | | | |  |  |  |  |  |  |
|  | Qn 3: INTERVIEWER: and incase of incidental findings, who handles such results?  RESPONDENT: So what do you mean by incidental findings for example?  INTERVIEWER: We went to, or we did this study to find out the kinetics of this particular drug in this individual, but as you do the simple thing you discover that the genome has maybe other important findings, they’re not related to your objective but since you do screening and you get the all genome you’ve discovered there is something of significance to an individual or to your research participants. Do you report those ones, do you return those to the individuals?  RESPONDENT: Well, ahaa you can publish, and you, of course as I said it is all in the identified data and you shouldn’t be able to particularly know who is who, ahaa so for the start that can be published and people can find out about it. When it comes to returning to the individual, we again go back to the same situation we were in before which is really, you have to think carefully about what’s the implication to the individual, again family and society. | | | | | | | | | | | | | | | |  |
|  |  |  |  |  |  |  |  |  |  |  |  |  |  |  |  |  |  |
|  |  | | | | | | | | | | | | | | | |  |
|  | | | **Files\\Male\\R-009.male** | | | | | | | | | | | | | |  |
|  |  |  | No |  |  |  |  |  |  | |  | | | | | |  |
|  | | |  |  |  |  |  |  |  | |  | | | | | | |
|  | | | | | | | | | | | |  |  |  |  |  |  |
|  | RESPONDENT: incidental means things you never really intended to discover but then you get them actually many times that is a discovery, many times you really get to incidental findings for example we had one of our students we were looking at, when you sequence the exosomes, the exosomes are the coding regions but our student was looking at intromoes these are regions in between the other coding regions and was trying to look for greater regions and the student really stumbled in finding that, that region 20% of the people in that region had viruses other viruses not HIV other viruses enriched there, there was one specific virus that was very common and I think that the people who had that virus were probably if its hypothesis go right like we are trying to seek are really long term non progressors they survive more than the other people who don’t have the virus so this is an incidental finding, we were never looking for the virus we were looking for other markers well it depends on how, now how do you take that information to that community so but if it has benefits, may be if it is proven then it is not particularly your personal information but now this is a virus which is probably in your community which is very common so again that context may be a community might be informed if you don’t know these are complicated issues, those incidental findings again to me I would treat them like the other findings we have talked about, do they have meaning? Is that meaning benefial? If the meaning has a bad outcome, that case still I would handle it in the same way, I wouldn’t say I wasn’t looking for it therefore I am not going to disclose | | | | | | | | | | | | | | | |  |
|  |  |  |  |  |  |  |  |  |  |  |  |  |  |  |  |  |  |
|  |  | | | | | | | | | | | | | | | |  |
|  | | | **Files\\Male\\R-012.male** | | | | | | | | | | | | | |  |
|  |  |  | No |  |  |  |  |  |  | |  | | | | | |  |
|  | | |  |  |  |  |  |  |  | |  | | | | | | |
|  | | | | | | | | | | | |  |  |  |  |  |  |
|  | RESPONDENT: like I have told that I do not coz this is a gene expression I don’t think we do really have incidental findings because its just like all of us have the same genes and for certain reasons not disease there will always be some structures even for the same person in times of livelihood differences in gene expressions so I don’t expect them very unique and actually from the data I have worked on but not from anything that could be extreme that will require you to report back | | | | | | | | | | | | | | | |  |
|  |  | | | | | | | | | | | | | | | |  |
|  | | | | | | | | | | | | | | | | | |
|  | | | | | | | | | | | | | | | | | |
| Reports\\Coding Summary By Code Report | | | | | | | | | | Page 41 of 109 | | | | | | | |
|  | | | | | | | | | | | | | | | | | |
|  | | | **Aggregate** |  | **Classification** |  | **Coverage** |  | **Number Of Coding References** | |  | **Reference Number** |  | **Coded By Initials** |  | **Modified On** |  |
|  | | | **Files\\Male\\R-016. male** | | | | | | | | | | | | | |  |
|  |  |  | No |  |  |  |  |  |  | |  | | | | | |  |
|  | | |  |  |  |  |  |  |  | |  | | | | | | |
|  | | | | | | | | | | | |  |  |  |  |  |  |
|  | RESPONDENT: I cannot speak for the other people, I can only speak for myself and what am telling is that am not going to look for information that I don’t need, am only going to collect information from saliva samples from these individuals and then look for any association because its what we call central study so you have people with mental illness and then you have people without mental illness then you match them and compare so you look at the genetics of this individual and the genetics of that individual then you compare, the people who are going to be reading this information will not even know who these participants are, it is corded so that the only information we have is that this is a male 25 and may be Acholi just three questions so they can’t know who this person is they are not supposed to know so that also people don’t get back to you and give you that information in case somebody else hacks this information and get it then go around and say you know what, so and so has a gene of bipolar syndrome or something like that and then you know what, that means that basically maybe people will not want to associate with you, may be you children with your family or whatever it is so we don’t look for anything else apart from that so usually we get incidental findings when for example you’re going to do genetics studies or something then you that this is person may be has a tumor in the brain and then you have to inform them about it coz it is ethically correct then you find that that’s not what we do | | | | | | | | | | | | | | | |  |
|  |  |  |  |  |  |  |  |  |  |  |  |  |  |  |  |  |  |
|  |  | | | | | | | | | | | | | | | |  |
|  | | | **Files\\Male\\R-017. male** | | | | | | | | | | | | | |  |
|  |  |  | No |  |  |  |  |  |  | |  | | | | | |  |
|  | | |  |  |  |  |  |  |  | |  | | | | | | |
|  | | | | | | | | | | | |  |  |  |  |  |  |
|  | Respondent: We would advise the hospital to have that kind of procedure, depending on the kind of research we are doing | | | | | | | | | | | | | | | |  |
|  |  | | | | | | | | | | | | | | | |  |
|  | | | **Files\\Male\\R-022. male** | | | | | | | | | | | | | |  |
|  |  |  | No |  |  |  |  |  |  | |  | | | | | |  |
|  | | |  |  |  |  |  |  |  | |  | | | | | | |
|  | | | | | | | | | | | |  |  |  |  |  |  |
|  | RESPONDENT: as you know, findings may be incidental, that is part of research; sometime you can have a hypothesis, probably its believing in a certain thing; you say, this causes this but sometimes you may find the contrary. Those are all incidental and that’s why you do research anyway because you don’t actually know. | | | | | | | | | | | | | | | |  |
|  |  | | | | | | | | | | | | | | | |  |
|  | | | **Files\\Male\\R-025 male..** | | | | | | | | | | | | | |  |
|  |  |  | No |  |  |  |  |  |  | |  | | | | | |  |
|  | | |  |  |  |  |  |  |  | |  | | | | | | |
|  | | | | | | | | | | | |  |  |  |  |  |  |
|  | Respondent: Ahaa not yet, we have found something in the group of Epistein-Barrr virus, because we have found certain virus which are more common with cancer cells but this one we are just publishing and then we are trying to see if other centers also get similar results, so we have compared it with blood samples, and it looks like there are certain virus much associated with that, so this is the stage which is there. The next stage of course if it is conclusive, we have compared so far two, but if it comes up now that it is this virus most associated with that, then we will just to interest those companies who are trying to develop the vaccines | | | | | | | | | | | | | | | |  |
|  |  | | | | | | | | | | | | | | | |  |
|  | | | | | | | | | | | | | | | | | |
| Reports\\Coding Summary By Code Report | | | | | | | | | | Page 42 of 109 | | | | | | | |
|  | | | | | | | | | | | | | | | | | |
|  | | | **Aggregate** |  | **Classification** |  | **Coverage** |  | **Number Of Coding References** | |  | **Reference Number** |  | **Coded By Initials** |  | **Modified On** |  |
|  | | | | | | | | | | | | | | | | | |
|  | | | | | | | | | | | |  |  |  |  |  |  |
|  | Interviewer: How about if that condition might be treatable somewhere?  Respondent: Yeah sure, I assume if it is a treatable thing, then you can do, but ours here is mainly cancer and you know cancer is for a longtime period, one has to detect it to screen and then probably later, but the individual must be available to undergo close follow up, but if it is treatable sure  Interviewer: Yeah because you are doing cancer and because you are doing the whole genome, you find some one has a gene for thalassemia or sickle cell or something else, would you report that one to the individual? because it is not what you targeted but as you do it, it was inevitable you also had to look at that one  Respondent: Yeah sure, by the way, maybe I didn’t elaborate, this study also looks at non-genetic defect that protects against malaria. Now if, because the thing is that malaria is one of the factors that help decrease your immunity to Burkitt’s lymphoma and may predispose you to Burkitt’s lymphoma, so if you mix malaria, Epistein-Barr virus and then you genetic, it seems there is a genetic part to make. So, we are looking at malaria resistance genes and that means that carriers of sickle cell or Sickler, we are also interested to see if there are many of them who have Burkitt’s lymphoma so for those ones we give them the results. | | | | | | | | | | | | | | | |  |
|  |  |  |  |  |  |  |  |  |  |  |  |  |  |  |  |  |  |
|  |  | | | | | | | | | | | | | | | |  |
|  | **Nodes\\Return of results\\Opinion on return of results\Incidental findings\No** | | | | | | | | | | | | | | | |  |
|  | | **Document** | | | | | | | | | | | | | | |  |
|  | | | **Files\\Female\\R-021 female** | | | | | | | | | | | | | |  |
|  |  |  | No |  |  |  |  |  |  | |  | | | | | |  |
|  | | |  |  |  |  |  |  |  | |  | | | | | | |
|  | | | | | | | | | | | |  |  |  |  |  |  |
|  | Respondent: What are incidental findings?  Interviewer: For example, your objective maybe determining those immune responses in HIV however along the way you realize some other information that may be relevant but however does not address your major objective  Respondent: We always get out of hand, okay let me call them incidental but something which is not expected, you expect that everybody who has been exposed to something has some immunity, then you find those who don’t have anything at all yet they are infected, and I don’t know whether you consider that to be an incidental finding? So, it takes you from your direction of looking at what are the good immune responses, why do some people never respond at all. So, you pull out those people to see what the difference is. Again, that takes us back to genetics, was it  certain genetics that some people never, never respond completely at all? | | | | | | | | | | | | | | | |  |
|  |  |  |  |  |  |  |  |  |  |  |  |  |  |  |  |  |  |
|  |  | | | | | | | | | | | | | | | |  |
|  | | | | | | | | | | | |  |  |  |  |  |  |
|  | Respondent: So, we don’t have to tell the people each and everything we are doing, we are investigating, we are giving them general findings, the consolidated findings where you draw graphs and they show in general, this is the response but those details where you get sub groups of people, we don’t. Its also difficult to explain to the population. We focus on our major objective but if there were some additional objectives arising from the findings, those are research points | | | | | | | | | | | | | | | |  |
|  |  | | | | | | | | | | | | | | | |  |
|  | | | **Files\\Female\\R029 female** | | | | | | | | | | | | | |  |
|  |  |  | No |  |  |  |  |  |  | |  | | | | | |  |
|  | | |  |  |  |  |  |  |  | |  | | | | | | |
|  | | | | | | | | | | | |  |  |  |  |  |  |
|  | RESPONDENT: if they have no impact on their lives like what kind of scenario would have no impact? | | | | | | | | | | | | | | | |  |
|  |  | | | | | | | | | | | | | | | |  |
|  | | | | | | | | | | | | | | | | | |
| Reports\\Coding Summary By Code Report | | | | | | | | | | Page 43 of 109 | | | | | | | |
|  | | | | | | | | | | | | | | | | | |
|  | | | **Aggregate** |  | **Classification** |  | **Coverage** |  | **Number Of Coding References** | |  | **Reference Number** |  | **Coded By Initials** |  | **Modified On** |  |
|  | | | **Files\\Male\\R-015.male** | | | | | | | | | | | | | |  |
|  |  |  | No |  |  |  |  |  |  | |  | | | | | |  |
|  | | |  |  |  |  |  |  |  | |  | | | | | | |
|  | | | | | | | | | | | |  |  |  |  |  |  |
|  | RESPONDENT: if it is not actionable then you leave it you forget about it in passing when you have given other results  INTERVIEWER: without considering the implication  RESPONDENT: of course you have to think about all this but ultimately keep the information because what is worse is when you find out that people knew this and after five years they are telling you that they knew about it. You find that as a research community you lose trust to the researcher although for now we may have few of those examples but I think one day we shall have a big example, what is going to be the impact of research?, how are people in certain community going to for example are doing a lot of research on the genetics of this individual which has received a lot of this that has been publicized a lot so what if you find there something important and you keep quiet about it when it has a negative connotation then after sometime somewhere it comes out how is that group that has been affected going to trust people again | | | | | | | | | | | | | | | |  |
|  |  |  |  |  |  |  |  |  |  |  |  |  |  |  |  |  |  |
|  |  | | | | | | | | | | | | | | | |  |
|  | | | **Files\\Male\\R-016. male** | | | | | | | | | | | | | |  |
|  |  |  | No |  |  |  |  |  |  | |  | | | | | |  |
|  | | |  |  |  |  |  |  |  | |  | | | | | | |
|  | | | | | | | | | | | |  |  |  |  |  |  |
|  | INTERVIEWER: how about incidental findings, how do you handle incidental findings?  RESPONDENT: there are no incidental findings that we expect in this research  INTERVIEWER: what if on the general picture of genetics and genomics there are usually incidental findings | | | | | | | | | | | | | | | |  |
|  |  | | | | | | | | | | | | | | | |  |
|  | | | | | | | | | | | |  |  |  |  |  |  |
|  | RESPONDENT: like which? This particular one I am doing it really needs to be educated about what that is  INTERVIEWER: I think now for example; since you relate with mental illnesses to see if it is genetically inherited, now as you’re conducting that you may find that may be this son of this family is not a real son of that family  RESPONDENT: we cannot get that information because we don’t collect information from relatives we don’t even know who these people’s relatives are, we are also ethically obliged not to share information that lead to like communities being stigmatized or persecuted so for example the data, in central Buganda we people, we have people in Gulu, we have people in Mbarara, we cant all of sudden wake up one day and say that the people in Mbarara or the people in Gulu have such a high incidence of mental illness which is genetically linked at some point if you’re careless to information basically what that means is that in future people will say we don’t have anything to do with those individuals that’s why we are extremely careful about what we are going to make the public aware about | | | | | | | | | | | | | | | |  |
|  |  |  |  |  |  |  |  |  |  |  |  |  |  |  |  |  |  |
|  |  | | | | | | | | | | | | | | | |  |
|  | **Nodes\\Return of results\\Opinion on return of results\Incidental findings\Yes** | | | | | | | | | | | | | | | |  |
|  | | **Document** | | | | | | | | | | | | | | |  |
|  | | | **Files\\Female\\R-003.female** | | | | | | | | | | | | | |  |
|  |  |  | No |  |  |  |  |  |  | |  | | | | | |  |
|  | | |  |  |  |  |  |  |  | |  | | | | | | |
|  | | | | | | | | | | | |  |  |  |  |  |  |
|  | RESPONDENT: you report them I think I would report them, still in relation to genetics you know am actually realizing this field is quite interesting but also challenging because we do research to seek findings, and research is meant to benefit at the end of the day there must be information so if someone has this information and they keep the information to themselves it is also not good. So, there should be a balance really, a balance of how much should be realized and how it should be packaged | | | | | | | | | | | | | | | |  |
|  |  | | | | | | | | | | | | | | | |  |
| Reports\\Coding Summary By Code Report | | | | | | | | | | Page 44 of 109 | | | | | | | |
|  | | | | | | | | | | | | | | | | | |
|  | | | **Aggregate** |  | **Classification** |  | **Coverage** |  | **Number Of Coding References** | |  | **Reference Number** |  | **Coded By Initials** |  | **Modified On** |  |
|  | | | | | | | | | | | | | | | | | |
|  | | | | | | | | | | | |  |  |  |  |  |  |
|  | INTERVIEWER: would you then propose that during the informed consent it should also add a component of incidental findings  RESPONDENT: yes | | | | | | | | | | | | | | | |  |
|  |  | | | | | | | | | | | | | | | |  |
|  | | | **Files\\Female\\R026 female** | | | | | | | | | | | | | |  |
|  |  |  | No |  |  |  |  |  |  | |  | | | | | |  |
|  | | |  |  |  |  |  |  |  | |  | | | | | | |
|  | | | | | | | | | | | |  |  |  |  |  |  |
|  | INTERVIEWER: So, supposing there were incidental findings that resulted from the study, and they had far reaching consequences to the participants and those incidental findings had clinical value, does the study take into consideration in communicating such incidental findings to the participants seeing that the consequences are beyond them?  RESPONDENT: You know our study is in the clinical stage. Pre-clinically there are genes that are associated with TB risk and no risk. Now we are trying to look at the different groups, and see how often does that gene occur in each population, so we don’t lose scope in looking at different genes and what there is, so we wouldn’t discover a person’s pieces because we are not looking for that | | | | | | | | | | | | | | | |  |
|  |  | | | | | | | | | | | | | | | |  |
|  | | | | | | | | | | | |  |  |  |  |  |  |
|  | RESPONDENT: As I said the work we do we trying to see whether a gene that was seen else where as a hand for TB is the same gene found in this population, so we are not scooping we are looking, does A appear here, yes or no? As opposed to what genes do they have or they have this gene or they have this gene what is it going to do. That’s not what we do, and our results come out saying yes, we also found these ones. You know the issue of genetic studies can be broad and that is when it gets…but its not black and white | | | | | | | | | | | | | | | |  |
|  |  | | | | | | | | | | | | | | | |  |
|  | | | **Files\\Female\\R028.female** | | | | | | | | | | | | | |  |
|  |  |  | No |  |  |  |  |  |  | |  | | | | | |  |
|  | | |  |  |  |  |  |  |  | |  | | | | | | |
|  | | | | | | | | | | | |  |  |  |  |  |  |
|  | INTERVIEWER: So, it depends on whether the results have a clinical significance?  RESPONDENT: Yes, or whether there is something you can change at that point. If you can’t fix a problem, you create less anxiety by not revealing | | | | | | | | | | | | | | | |  |
|  |  | | | | | | | | | | | | | | | |  |
|  | | | **Files\\Female\\R029 female** | | | | | | | | | | | | | |  |
|  |  |  | No |  |  |  |  |  |  | |  | | | | | |  |
|  | | |  |  |  |  |  |  |  | |  | | | | | | |
|  | | | | | | | | | | | |  |  |  |  |  |  |
|  | INTERVIEWER: What if you got incidental findings in the study how would you handle the results? You know what incidental findings are?  RESPONDENT: I think these are findings that you didn’t expect but then turn out as relevant. But then if it impacts on the treatment of this participant and is clinically relevant, then I think I would first approach the attending doctor and because we enrolled theses participants from the Hospital, so I would first discuss with the attending doctor and discuss the results and then from there we can take it on.  INTERVIEWER: In your view as the researcher  RESPONDENT: in my view as a researcher?  INTERVIEWER: would you return or communicate to the participants?  RESPONDENT:I think I would, yaa if the conditions allow I think I would , because if their life will depend on that, or they are likely to improve their quality of life. I think its humane to communicate and if they can get treatment. | | | | | | | | | | | | | | | |  |
|  |  |  |  |  |  |  |  |  |  |  |  |  |  |  |  |  |  |
|  |  | | | | | | | | | | | | | | | |  |
|  | | | | | | | | | | | |  |  |  |  |  |  |
|  | INTERVIEWER: you had not included it in the consent form but you feel this person if not communicated to, he/she may die in the next year, or something like that or something bad might happen because it is something that might affect her life. These are risk factors.  RESPONDENT: I again being humane, I think I would report such a finding but the way to handle such also matters. I think you need a counsellor to explain if you are not a counselor yourself. Maybe you need a genetic counsellor or a counsellor who can break it down to the patient. Again it may be an advantage to the patient that they have found such results to prevent occurrence of such diseases. | | | | | | | | | | | | | | | |  |
|  |  | | | | | | | | | | | | | | | |  |
| Reports\\Coding Summary By Code Report | | | | | | | | | | Page 45 of 109 | | | | | | | |
|  | | | | | | | | | | | | | | | | | |
|  | | | **Aggregate** |  | **Classification** |  | **Coverage** |  | **Number Of Coding References** | |  | **Reference Number** |  | **Coded By Initials** |  | **Modified On** |  |
|  | | | **Files\\Female\\R030.female** | | | | | | | | | | | | | |  |
|  |  |  | No |  |  |  |  |  |  | |  | | | | | |  |
|  | | |  |  |  |  |  |  |  | |  | | | | | | |
|  | | | | | | | | | | | |  |  |  |  |  |  |
|  | INTERVIEWER: But then like you said, what if it is detrimental, it may not be having a solution?  RESPONDENT: Yes, but you also will have to, if it is completely outside what you thought again it is IRB and then it is also the patient, we may have to review the consent form and see how to communicate that information if it is necessary to the participant. So, either way, participants participation either emotionally, socially and other wise is the first thing that you must consider. | | | | | | | | | | | | | | | |  |
|  |  | | | | | | | | | | | | | | | |  |
|  | | | **Files\\Male\\R-001 male** | | | | | | | | | | | | | |  |
|  |  |  | No |  |  |  |  |  |  | |  | | | | | |  |
|  | | |  |  |  |  |  |  |  | |  | | | | | | |
|  | | | | | | | | | | | |  |  |  |  |  |  |
|  | Incidental findings are also very difficult at some point because you didn't go for them but you have found them. But as i said, it still comes down to what is the benefit of giving these results to this person, some results are incidental but are really important. They are important that you need to give them to the person for them to be able to take action. i mean the incidental findings usually come up during exploration, you are just trying to screen then you find this, you didn't consent, no body consented for that. But i Would think one should take responsibility to share the information because in the end you are dealing with human Life, its not like you have found an incident reverse some where but you are dealing with human beings so i would think it would be prudent that you help them. | | | | | | | | | | | | | | | |  |
|  |  | | | | | | | | | | | | | | | |  |
|  | | | **Files\\Male\\R-002. male** | | | | | | | | | | | | | |  |
|  |  |  | No |  |  |  |  |  |  | |  | | | | | |  |
|  | | |  |  |  |  |  |  |  | |  | | | | | | |
|  | | | | | | | | | | | |  |  |  |  |  |  |
|  | Respondent: yeah, you’re not really expecting and then you land on this kind of information that is something, so incidental findings I think once you come across such information its important to because research is very determined by some policies ethics issues and stuff like that if it is not clear within the framework currently, I think it is best to may be discuss with the regulators | | | | | | | | | | | | | | | |  |
|  |  | | | | | | | | | | | | | | | |  |
|  | | | | | | | | | | | |  |  |  |  |  |  |
|  | Interviewer: so, you think its important to pass to this information to people?  Respondent: incidental findings  Interviewer: yes  Respondent: its good to investigate further to really get to the bottom before that because you can learn on an information and before you investigate it its not good to run down with it, its good to more consult more and then together you might find the best way to handle it, I am really not like sure what to do | | | | | | | | | | | | | | | |  |
|  |  | | | | | | | | | | | | | | | |  |
|  | | | **Files\\Male\\R-004.male** | | | | | | | | | | | | | |  |
|  |  |  | No |  |  |  |  |  |  | |  | | | | | |  |
|  | | |  |  |  |  |  |  |  | |  | | | | | | |
|  | | | | | | | | | | | |  |  |  |  |  |  |
|  | Respondent: my opinion will stay that if there is something incidental still the donor who was the source should be informed and be given whatever knowledge is adequate or worth depending on the consent of the development at that stage | | | | | | | | | | | | | | | |  |
|  |  | | | | | | | | | | | | | | | |  |
|  | | | | | | | | | | | |  |  |  |  |  |  |
|  |  | | | | | | | | | | | | | | | |  |
|  |  | | | | | | | | | | | | | | | |  |
| Reports\\Coding Summary By Code Report | | | | | | | | | | Page 46 of 109 | | | | | | | |
|  | | | | | | | | | | | | | | | | | |
|  | | | **Aggregate** |  | **Classification** |  | **Coverage** |  | **Number Of Coding References** | |  | **Reference Number** |  | **Coded By Initials** |  | **Modified On** |  |
|  | | | **Files\\Male\\R-005.male** | | | | | | | | | | | | | |  |
|  |  |  | No |  |  |  |  |  |  | |  | | | | | |  |
|  | | |  |  |  |  |  |  |  | |  | | | | | | |
|  | | | | | | | | | | | |  |  |  |  |  |  |
|  | Interviewer: Okay, so in case of incidental findings, how do you handle them?  Respondent: Actually incidental findings, you have an obligation to communicate them, but you don’t just wake up and just communicate them just like that. Some of us are not like PIs but you involve the guys who are in charge and say, hey we found this please, what do we have to do? And then those usually the big people know how to communicate them probably | | | | | | | | | | | | | | | |  |
|  |  | | | | | | | | | | | | | | | |  |
|  | | | **Files\\Male\\R-009.male** | | | | | | | | | | | | | |  |
|  |  |  | No |  |  |  |  |  |  | |  | | | | | |  |
|  | | |  |  |  |  |  |  |  | |  | | | | | | |
|  | | | | | | | | | | | |  |  |  |  |  |  |
|  | RESPONDENT: incidental means things you never really intended to discover but then you get them actually many times that is a discovery, many times you really get to incidental findings for example we had one of our students we were looking at, when you sequence the exosomes, the exosomes are the coding regions but our student was looking at intromoes these are regions in between the other coding regions and was trying to look for greater regions and the student really stumbled in finding that, that region 20% of the people in that region had viruses other viruses not HIV other viruses enriched there, there was one specific virus that was very common and I think that the people who had that virus were probably if its hypothesis go right like we are trying to seek are really long term non progressors they survive more than the other people who don’t have the virus so this is an incidental finding, we were never looking for the virus we were looking for other markers well it depends on how, now how do you take that information to that community so but if it has benefits, may be if it is proven then it is not particularly your personal information but now this is a virus which is probably in your community which is very common so again that context may be a community might be informed if you don’t know these are complicated issues, those incidental findings again to me I would treat them like the other findings we have talked about, do they have meaning? Is that meaning benefial? If the meaning has a bad outcome, that case still I would handle it in the same way, I wouldn’t say I wasn’t looking for it therefore I am not going to disclose | | | | | | | | | | | | | | | |  |
|  |  |  |  |  |  |  |  |  |  |  |  |  |  |  |  |  |  |
|  |  | | | | | | | | | | | | | | | |  |
|  | | | **Files\\Male\\R-012.male** | | | | | | | | | | | | | |  |
|  |  |  | No |  |  |  |  |  |  | |  | | | | | |  |
|  | | |  |  |  |  |  |  |  | |  | | | | | | |
|  | | | | | | | | | | | |  |  |  |  |  |  |
|  | RESPONDENT: what would I do, actually I would say the results for the study clinicians those ones I would tell them the incidental findings not everyone of course I would tell the person in charge like the PI or that one I would tell them but the patient I would tell them | | | | | | | | | | | | | | | |  |
|  |  | | | | | | | | | | | | | | | |  |
|  | | | **Files\\Male\\R-015.male** | | | | | | | | | | | | | |  |
|  |  |  | No |  |  |  |  |  |  | |  | | | | | |  |
|  | | |  |  |  |  |  |  |  | |  | | | | | | |
|  | | | | | | | | | | | |  |  |  |  |  |  |
|  | RESPONDENT: return of results is in two ways; we have incidental findings and expected findings, usually expected findings you may not return them because you are expected to find them | | | | | | | | | | | | | | | |  |
|  |  | | | | | | | | | | | | | | | |  |
|  | | | | | | | | | | | |  |  |  |  |  |  |
|  | RESPONDENT: we have the two things there is communications about the research this is that we found then there is other one which is actionable or incidental in terms of ending a research you would want to communicate back either through a study group; tell them exactly what you found in a very possible simple understandable way then of course the difficult ethical issue is incidental unexpected and probably sometimes actionable, for that one it is you people who have issues there but for me am thinking; if a result is actionable it should be communicated because knowledge is power, you give this person the knowledge and then they have the ability to decide on when to act especially now when we go into genetic findings because it is a new field and because it is close we can think about our own inferiority complex that anything that is found is because of the way we are and why people don’t like us. So for me I think any incidental finding whether actionable or not should be communicated back | | | | | | | | | | | | | | | |  |
|  |  |  |  |  |  |  |  |  |  |  |  |  |  |  |  |  |  |
|  |  | | | | | | | | | | | | | | | |  |
| Reports\\Coding Summary By Code Report | | | | | | | | | | Page 47 of 109 | | | | | | | |
|  | | | | | | | | | | | | | | | | | |
|  | | | **Aggregate** |  | **Classification** |  | **Coverage** |  | **Number Of Coding References** | |  | **Reference Number** |  | **Coded By Initials** |  | **Modified On** |  |
|  | | | **Files\\Male\\R-017. male** | | | | | | | | | | | | | |  |
|  |  |  | No |  |  |  |  |  |  | |  | | | | | |  |
|  | | |  |  |  |  |  |  |  | |  | | | | | | |
|  | | | | | | | | | | | |  |  |  |  |  |  |
|  | Respondent: Like I said, you just have to go through the procedure of getting back, because incidental findings some of them might be fatal, and it’s a life and death situation, we don’t have them in our studies but there are studies which are like that, its life and death, and you have to go through the procedure of the hospital where you got it from, inform them and then they also have their unique procedures how they actually get to the individual who is affected, but me the researcher I cant directly interface or get a person’s number and tell them that you, you have this | | | | | | | | | | | | | | | |  |
|  |  | | | | | | | | | | | | | | | |  |
|  | | | **Files\\Male\\R-022. male** | | | | | | | | | | | | | |  |
|  |  |  | No |  |  |  |  |  |  | |  | | | | | |  |
|  | | |  |  |  |  |  |  |  | |  | | | | | | |
|  | | | | | | | | | | | |  |  |  |  |  |  |
|  | INTERVIEWER: there is this other concern I have; something to be beneficial is also relative, what you may consider beneficial may not seem beneficial to that participant, leave alone the health benefits but more so the psychosocial benefits. Now, who judges/ decides on what is beneficial?  RESPONDENT: it is really relative, its like going for an HIV test, from a public health perspective, it is beneficial to know people’s status but it doesn’t really help directly the patient because the news is really never good. But on the wider perspective, the patient actually benefits though it will be very difficult for the patient; they will know their status, they will know that they have to take medication and from the wider perspective, the public; reduction of transmission, people be aware of what is happening. So, what may be beneficial to me may not be beneficial to the person Y or Z. so it very relative, you can’t really say this is beneficial but you need to holistically look at it. | | | | | | | | | | | | | | | |  |
|  |  |  |  |  |  |  |  |  |  |  |  |  |  |  |  |  |  |
|  |  | | | | | | | | | | | | | | | |  |
|  | | | **Files\\Male\\R-024.male** | | | | | | | | | | | | | |  |
|  |  |  | No |  |  |  |  |  |  | |  | | | | | |  |
|  | | |  |  |  |  |  |  |  | |  | | | | | | |
|  | | | | | | | | | | | |  |  |  |  |  |  |
|  | Interviewers: Okay, so any other cases of incidental findings that you have come across?  Respondent: yes, we’ve got cases where we’ve got triple inheritance, somebody has sickle cell, has alpha thalassemia and glucose-6-dehydrogenese. So you set out to survey a community, this was a community finding that you are now doing these innate xtics and one sample gives you a positive test for all those conditions and so you find yourself in a very big situation, because you are supposed to deliver a genetic finding of one type, but now for the same patient you are going to deliver all the 3 types  Interviewer: But they had consented for all the 3?  Respondent: They had consented for the procedure but not the 3 as individual, so those are very difficult cases in fact the particular incidence am talking about they are 11 patients with that, and even if you went to clinical setting, you find the clinician very confused, saying no, we normally don’t handle it that way, because they know a Sickler is a Sickler, glucose-6-dehydrogenese is glucose-6-dehydrogenese, but all of a sudden you get a patient with all the three in one  Interviewer: And probably you had not explained when you went to the community  Respondent: Actually, we looked back at literature and found that the dual inheritance tends to stabilize, so patients tend to be stable when they have a co-inheritance of this, but now the triple is what we don’t know  Interviewer: But you got this from the community? And you’ve been following them up?  Respondent: Yes, we got this in the community. Now we’ve followed since that time | | | | | | | | | | | | | | | |  |
|  |  |  |  |  |  |  |  |  |  |  |  |  |  |  |  |  |  |
|  |  |  |  |  |  |  |  |  |  |  |  |  |  |  |  |  |  |
|  |  | | | | | | | | | | | | | | | |  |
|  | | | | | | | | | | | |  |  |  |  |  |  |
|  | Interviewer: So just a follow up, do you return or communicate them to participants in case of incidental findings?  Respondent: Yes, we do | | | | | | | | | | | | | | | |  |
|  |  | | | | | | | | | | | | | | | |  |
|  | | | | | | | | | | | | | | | | | |
| Reports\\Coding Summary By Code Report | | | | | | | | | | Page 48 of 109 | | | | | | | |
|  | | | | | | | | | | | | | | | | | |
|  | | | **Aggregate** |  | **Classification** |  | **Coverage** |  | **Number Of Coding References** | |  | **Reference Number** |  | **Coded By Initials** |  | **Modified On** |  |
|  | | | **Files\\Male\\R-025 male..** | | | | | | | | | | | | | |  |
|  |  |  | No |  |  |  |  |  |  | |  | | | | | |  |
|  | | |  |  |  |  |  |  |  | |  | | | | | | |
|  | | | | | | | | | | | |  |  |  |  |  |  |
|  | Interviewer: Okay, and the participants, will you report back to them now?  Respondent: The participants we’ll just tell them, because it was also very difficult to tell them that this small germ is associated with Burkitt’s lymphoma, because their question was, say now how do you get this germ? And we mention to them maybe exchange of saliva and your parent or mother especially, so it is just that information we give. So even any information that we are going to give if the study dissemination is going to do is just going to be really general information | | | | | | | | | | | | | | | |  |
|  |  | | | | | | | | | | | | | | | |  |
|  | **Nodes\\Return of results\\Opinion on return of results\Incidental findings\Yes\Conditions** | | | | | | | | | | | | | | | |  |
|  | | **Document** | | | | | | | | | | | | | | |  |
|  | | | **Files\\Female\\R-003.female** | | | | | | | | | | | | | |  |
|  |  |  | Yes |  |  |  |  |  |  | |  | | | | | |  |
|  | | |  |  |  |  |  |  |  | |  | | | | | | |
|  | | | | | | | | | | | |  |  |  |  |  |  |
|  | INTERVIEWER: so, like under what conditions would you report or give back incidental findings of genomics research?  RESPONDENT: under what circumstances, to give them to who? | | | | | | | | | | | | | | | |  |
|  |  | | | | | | | | | | | | | | | |  |
|  | | | | | | | | | | | |  |  |  |  |  |  |
|  | RESPONDENT: still one if those findings can be used take for instance may be clinicians I would provide that information if it is going to be put to good use like that but even then I still feel it is a right if someone really wants their results if they want their results its their right to get their results whether they are benefial or not first of all before someone for instance before we have involved any project that is involving human participants I would request for their consent and indeed these people or this person will be seeing for him or her self so its their right to know but then I as a researcher I would take it upon my self to try and help them understand some of these things which are not well known but I believe it would be there but | | | | | | | | | | | | | | | |  |
|  |  | | | | | | | | | | | | | | | |  |
|  | | | | | | | | | | | |  |  |  |  |  |  |
|  | INTERVIEWER: incase we have come up with incidental findings then we might get back to you?  RESPONDENT: yes because then short of that its like a researcher gets information and then hides it from the students which is not good and for me I would still advocate for every consent to have a clear phrase that this study involves either genetics or genomics and we are going to get this sample and blab blab this information can easily help us know the real so that some one knows what they are getting themselves in to whether they want or not  INTERVIEWER: so that one would cover like if we get any useful information outside the main study then he will get back to you with that information  RESPONDENT: say it again | | | | | | | | | | | | | | | |  |
|  |  |  |  |  |  |  |  |  |  |  |  |  |  |  |  |  |  |
|  |  | | | | | | | | | | | | | | | |  |
|  | | | | | | | | | | | |  |  |  |  |  |  |
|  | INTERVIEWER: the consent will say that we are looking at gene ABCD but should we get something outside this gene that is of importance I don’t which importance now clinical or social then we can also report it back to you  RESPONDENT: as long as there are funds to disseminate or to reach out to all those people | | | | | | | | | | | | | | | |  |
|  |  | | | | | | | | | | | | | | | |  |
|  | | | | | | | | | | | | | | | | | |
| Reports\\Coding Summary By Code Report | | | | | | | | | | Page 49 of 109 | | | | | | | |
|  | | | | | | | | | | | | | | | | | |
|  | | | **Aggregate** |  | **Classification** |  | **Coverage** |  | **Number Of Coding References** | |  | **Reference Number** |  | **Coded By Initials** |  | **Modified On** |  |
|  | | | **Files\\Female\\R026 female** | | | | | | | | | | | | | |  |
|  |  |  | Yes |  |  |  |  |  |  | |  | | | | | |  |
|  | | |  |  |  |  |  |  |  | |  | | | | | | |
|  | | | | | | | | | | | |  |  |  |  |  |  |
|  | RESPONDENT: So, feedback we come out with a feedback format, usually a one page giving in simple language and overview of what we found. We did this result, looking at genes and we found that there are some types of body make up that makes some people more prone to TB than others or there is no body make up. So, we’ve done this only once by the way. Because you know it takes time to get results and they don’t do the analysis all the time, we store them and then when we are ready we do the analysis which is done by our colleagues in other countries, so it is not that we are doing it in real time | | | | | | | | | | | | | | | |  |
|  |  | | | | | | | | | | | | | | | |  |
|  | | | | | | | | | | | |  |  |  |  |  |  |
|  | INTERVIEWER: So, is this one page translated to the language they understand?  RESPONDENT: Yes, it is translated and approved by the IRB.  INTERVIEWER: One of the issues which is at the heart of participants sharing their autonomy is the idea of sharing meaning. I was wondering whether in the process of taking back feedback, whether you have had challenges in communication complex terms into an easier language that can be understood by the participants so that they can appreciate the gravity of what you are trying to communicate, whether you have encountered that and how you have been addressing it  RESPONDENT: Interestingly I don’t have an answer to that, reason: we come up with what we think we should, the language and the writing we use, we go over it and in a number of times back and forth to make sure the language is simple enough. The translation is harder, some times the translation becomes a little bit longer. Then once we have agreed on this, we give it to the home visitors to take it out and talk to participants. I don’t think we have gotten any specific feedback that tells us whether people have understood or not. Probably we have not asked for that, but we ask them to go to participants and talk to them and leave them a copy when they talk to them. Because we arrive at sometimes a complex language and you have to make it simple so that they understand and not too long | | | | | | | | | | | | | | | |  |
|  |  |  |  |  |  |  |  |  |  |  |  |  |  |  |  |  |  |
|  |  | | | | | | | | | | | | | | | |  |
|  | | | | | | | | | | | |  |  |  |  |  |  |
|  | RESPONDENT: When you have a clinically relevant finding definitely yes, otherwise its more of you tell people that we’ve finished, this is where we are and these are the next steps and again probably half the participants are interested the other half are not interested especially where the study objective was not primarily genetic. It was probably a clinical study objective and this was a research study so people are in a study to see whether drug A is as good as drug B, but in the process, you take off baseline samples which are used for genetics because we have a separate consent, I agree for my samples being used for genetic studies but usually genetic studies are not the primary studies. So, it is important for the primary one, but the genetic one, not at that stage. But it is not good to let people go without having told them of what came out | | | | | | | | | | | | | | | |  |
|  |  |  |  |  |  |  |  |  |  |  |  |  |  |  |  |  |  |
|  |  | | | | | | | | | | | | | | | |  |
|  | | | | | | | | | | | |  |  |  |  |  |  |
|  | I’ll give you an example one woman died of breast cancer, she was in her early 60’s. First forward, she had two daughters, one daughter was in another country, she went for genetic testing for breast cancer and they found that she had the gene for breast cancer, so she had double mastectomy. Now she has three daughters and I know she must have told her daughters to test for breast cancer because her mother died in her 60’s of breast cancer. But for her she did the test when she already has 6 children so she had the mastectomy but its impacting on her children. They are not yet married but what if they find that they have the gene for breast cancer, what do you tell them? Would you tell them to have mastectomy at 60? Coz for her now she’s in her late 60’s and she’s okay, no breasts but she’s fine. Then the other example, a person goes for paternity testing, a very good example, and you find that the child does not belong to this person. For example, one family got green cards, and daughter one failed the paternity test and the mother and daughter had to stay and others went, marriage broke up. Then on the other hand, I raise the child then for some reason we do paternity testing and the father says the child is not mine, I say impossible, let me also go, I actually find that the child is not mine either, the child is neither ours, this child was changed in the maternity ward 30 years ago. Now what does that mean? The child is now 25 years or 30, it means nothing. So, is it so important? So those are the situations whereas if my child is two years and I say we are not sure if this child is ours that is ok, there is still time to correct it, because we can go back to where we delivered from where there are records, which other child was born at that time and then try to trace that child, but if 25years, it is difficult. But there are certain situations where you are not supposed to conceal it. My grand father and senga both died of cancers, colon cancers, we want to know whether we have the gene for colon cancer, it is necessarily a yes or no, and you can’t tell me you don’t have it, but then what you are asking are the incidental findings, those are the complex ones and for the incidental findings you have three options before deciding: Will it impact on a persons life, what is the probability that they will get the disease? Is it preventable? Will they get the disease in the early years before 60, so that they should learn about it or they will get the problem at 80year? If you tell me you are at risk of getting ischemic heart disease in my 80’s, you don’t have to tell me its okay. But if you tell me that by the way you are prone at getting ischemic heart disease in your 40’s then it is good to know because then you can have regular check ups and family can know. And then the severity of the problem, you are at risk if getting vitelline, well so far it will not kill you but you will look not nice, while tell people a 20-year-old girl or 15-year-old that you have the probability of getting vitelline, and they ask what is the probability, oh 30, 40% probability. Why don’t you leave the person alone? | | | | | | | | | | | | | | | |  |
|  |  |  |  |  |  |  |  |  |  |  |  |  |  |  |  |  |  |
|  |  |  |  |  |  |  |  |  |  |  |  |  |  |  |  |  |  |
|  |  |  |  |  |  |  |  |  |  |  |  |  |  |  |  |  |  |
|  |  | | | | | | | | | | | | | | | |  |
|  | | | | | | | | | | | | | | | | | |
| Reports\\Coding Summary By Code Report | | | | | | | | | | Page 50 of 109 | | | | | | | |
|  | | | | | | | | | | | | | | | | | |
|  | | | **Aggregate** |  | **Classification** |  | **Coverage** |  | **Number Of Coding References** | |  | **Reference Number** |  | **Coded By Initials** |  | **Modified On** |  |
|  | | | **Files\\Female\\R027.female** | | | | | | | | | | | | | |  |
|  |  |  | Yes |  |  |  |  |  |  | |  | | | | | |  |
|  | | |  |  |  |  |  |  |  | |  | | | | | | |
|  | | | | | | | | | | | |  |  |  |  |  |  |
|  | RESPONDENT: I think I would want to ask the said patient before I put it in the consent. Do you want to know your results, if the results are sensitive, I will explain, you might discover things that are there but I would explain. I wouldn’t just go and tell them, it will distract families that is sensitive that if I discover such things I would put it in the consent and tell the patient before if they want to know or not. Some people don’t want to know, we can’t give information that I didn’t ask for.  INTERVIEWER: Because now like still for sickle cell thing someone may ask, in my family we may not have sickle cells, so how come this child has sickle cells so you may need to explain.  RESPONDENT: Then in that case because it is something obvious that they can see because the other one if it is something that has got to do with paternity they can’t know what you tell them but if you find out that someone is a Sickler they will see it so there you need to disclose to them but how you disclose, you must have a counsellor and involve the people involved. But still at the beginning if you know that in such study you can discover such things, you still need to inform them because even them or you need to say am not entering this study because I will get confusion in my home.  INTERVIEWER: I think that is kind of anticipated in paternity however, if we were to look at things that are may be related to disease like may be someone prone to a specific disease that for you, you are dealing with another line of disease then you realize that this gene or this person might get another condition so in that case it is not something you anticipated because it is not obvious but it can cover.  RESPONDENT: But in every research you know that there are somethings that can come up that are not anticipated so I think there is a way that you can cover and ask your patients if they will find anything that is not anticipated, would you like to be informed? Because not every one would want to know that they are prone to breast cancer, you might have gone for cervical cancer screening and they may find a gene that you are prone to sudden death like these people who just collapse, they don’t want to know, they want to enjoy life and they might make the quality of their life worse and say that I would still want to know at the beginning if there is anything which you would like to know. Or if I find it, I can call and say that I found some results which I need to inform you of the results of the genotype that I don’t think there is some information that some people are better off without. I will give you an example; people who go to test and see if their babies have abnormalities, they do that a lot either to terminate or to continue. In Uganda here, we don’t have that culture of terminating the baby because you hear the baby has Downs syndrome or whatever so if you do that test usually the probability is fifty and you can have down syndrome or that, so if a doctor tells you, you have a 50% chance of giving birth to a child with Downs syndrome, you can terminate the pregnancy but again there is a 50% chance of not having it. But for us we already have the knowledge and you might even terminate because of you don’t want to go down that road then you terminate when actually the child was normal so I can’t just dish out information, I need to tell you that there is this test, you can find out. Do you want to know? | | | | | | | | | | | | | | | |  |
|  |  |  |  |  |  |  |  |  |  |  |  |  |  |  |  |  |  |
|  |  |  |  |  |  |  |  |  |  |  |  |  |  |  |  |  |  |
|  |  |  |  |  |  |  |  |  |  |  |  |  |  |  |  |  |  |
|  |  | | | | | | | | | | | | | | | |  |
|  | | | **Files\\Female\\R028.female** | | | | | | | | | | | | | |  |
|  |  |  | Yes |  |  |  |  |  |  | |  | | | | | |  |
|  | | |  |  |  |  |  |  |  | |  | | | | | | |
|  | | | | | | | | | | | |  |  |  |  |  |  |
|  | INTERVIEWER: So, it depends on whether the results have a clinical significance?  RESPONDENT: Yes, or whether there is something you can change at that point. If you can’t fix a problem, you create less anxiety by not revealing | | | | | | | | | | | | | | | |  |
|  |  | | | | | | | | | | | | | | | |  |
|  | | | | | | | | | | | | | | | | | |
|  | | | | | | | | | | | | | | | | | |
|  | | | | | | | | | | | | | | | | | |
| Reports\\Coding Summary By Code Report | | | | | | | | | | Page 51 of 109 | | | | | | | |
|  | | | | | | | | | | | | | | | | | |
|  | | | **Aggregate** |  | **Classification** |  | **Coverage** |  | **Number Of Coding References** | |  | **Reference Number** |  | **Coded By Initials** |  | **Modified On** |  |
|  | | | | | | | | | | | | | | | | | |
|  | | | | | | | | | | | |  |  |  |  |  |  |
|  | INTERVIEWER: Okay you’ve talked of results having clinical significance?  RESPONDENT: Yes, clinical significance, and then the other one is when you have been mandated by the IRB that you return, because many times they may insist that you have to let patients know. Because the parallel situation with these researches we do is that besides them there are other clinical tests we make for example like to know how some one’s kidneys are functioning, blood, they are important in the even the ongoing clinical care, maybe blood group, those ones we always give but in genetics because they take long to get to know, actually they come back long probably after the woman gave birth. Still goes back to how useful is it in managing the patient, but for example if things change and it becames clear that this gene has become important people need to know, then you have to go back and let them know even if they are not in the active cohort, that’s what I think, or if it is even found to be associated with some thing different from what you had set out to investigate  INTERVIEWER: That would be like an incidental finding?  RESPONDENT: It is incidental, but if in your cohort you are able to tell that a certain group of people have a particular gene and for example am looking for hypertension but I find out that it is found to be associated by cancer of the liver, strongly clinically by another group, you might but I also know that is not practically easy. Because even when I look at my patients I still interact with them some 10 years down the road, I never really know what their genotype was, because I have not taken the interest to find out, so I talk to them things are there but I don’t know whether they have good or bad genes as we call them  INTERVIEWER: But if you are mandated to return results to individuals, what are those circumstances that can be in place? Would you advise to have a genetic counsellor or?  RESPONDENT: But why? Anyway, me I think it is because of the kinds of genes am dealing with, they are not disease genes, these are immune system genes, everybody has them but what they have is different. Its not like for example sickle cell disease, where there is clearly an abnormal variant. These immune system genes am studying are good for certain things and bad for others, they are not out right bad. So, it is like blood groups, people will say this group does this and this, but in the end, it doesn’t matter what blood group you have, other that the advantage that one is easier to be transfused. So far, I think that is not why I have encountered those situations. Because everyone has them | | | | | | | | | | | | | | | |  |
|  |  |  |  |  |  |  |  |  |  |  |  |  |  |  |  |  |  |
|  |  |  |  |  |  |  |  |  |  |  |  |  |  |  |  |  |  |
|  |  |  |  |  |  |  |  |  |  |  |  |  |  |  |  |  |  |
|  |  | | | | | | | | | | | | | | | |  |
|  | | | **Files\\Female\\R029 female** | | | | | | | | | | | | | |  |
|  |  |  | Yes |  |  |  |  |  |  | |  | | | | | |  |
|  | | |  |  |  |  |  |  |  | |  | | | | | | |
|  | | | | | | | | | | | |  |  |  |  |  |  |
|  | INTERVIEWER: What if you got incidental findings in the study how would you handle the results? You know what incidental findings are?  RESPONDENT: I think these are findings that you didn’t expect but then turn out as relevant. But then it impacts on the treatment of this participant and is clinically relevant, then I think I would first approach the attending doctor and because we enrolled theses participants from the Hospital, so I would first discuss with the attending doctor and discuss the results and then from there we can take it on.  INTERVIEWER: In your view as the researcher  RESPONDENT: in my view as a researcher?  INTERVIEWER: would you return or communicate to the participants?  RESPONDENT:I think I would, yaa if the conditions allow I think I would , because if their life will depend on that, or they are likely to improve their quality of life. I think its humane to communicate and if they can get treatment. | | | | | | | | | | | | | | | |  |
|  |  |  |  |  |  |  |  |  |  |  |  |  |  |  |  |  |  |
|  |  | | | | | | | | | | | | | | | |  |
|  | | | | | | | | | | | |  |  |  |  |  |  |
|  | INTERVIEWER: you had not included it in the consent form but you feel this person if not communicated to, he/she may die in the next year, or something like that or something bad might happen because it is something that might affect her life. These are risk factors.  RESPONDENT: I again being humane, I think I would report such a finding but the way handle such also matters. I think you need a counsellor to explain if you are not a counselor yourself. Maybe you need a genetic counsellor or a counsellor who can break it down to the patient. Again it may an advantage to the patient that they have found such results to prevent occurrence of such diseases. | | | | | | | | | | | | | | | |  |
|  |  | | | | | | | | | | | | | | | |  |
|  | | | **Files\\Female\\R030.female** | | | | | | | | | | | | | |  |
|  |  |  | Yes |  |  |  |  |  |  | |  | | | | | |  |
|  | | |  |  |  |  |  |  |  | |  | | | | | | |
|  | | | | | | | | | | | |  |  |  |  |  |  |
|  | INTERVIEWER: But then like you said, what if it is detrimental, it may not be having a solution?  RESPONDENT: Yes, but you also will have to, if it is completely outside what you thought again it is IRB and then it is also the patient, we may have to review the consent form and see how to communicate that information if it is necessary to the participant. So, either way, participants participation either emotionally, socially and other wise is the first thing that you must consider. | | | | | | | | | | | | | | | |  |
|  |  | | | | | | | | | | | | | | | |  |
| Reports\\Coding Summary By Code Report | | | | | | | | | | Page 52 of 109 | | | | | | | |
|  | | | | | | | | | | | | | | | | | |
|  | | | **Aggregate** |  | **Classification** |  | **Coverage** |  | **Number Of Coding References** | |  | **Reference Number** |  | **Coded By Initials** |  | **Modified On** |  |
|  | | | **Files\\Male\\R-001 male** | | | | | | | | | | | | | |  |
|  |  |  | Yes |  |  |  |  |  |  | |  | | | | | |  |
|  | | |  |  |  |  |  |  |  | |  | | | | | | |
|  | | | | | | | | | | | |  |  |  |  |  |  |
|  | I think it would be in the same conditions as i have given above; if this person is going to benefit or is going to be hurt by the findings, yes you need to return these results. And also it depends on why were you doing this work that you are doing. you know that some times we go and screen things, and screen more than we should screen and find something. so you should go back and find/establish whether its really significant, and go back to the person and talk to them again and find out if what you have found makes really sense to the person. | | | | | | | | | | | | | | | |  |
|  |  | | | | | | | | | | | | | | | |  |
|  | | | **Files\\Male\\R-002. male** | | | | | | | | | | | | | |  |
|  |  |  | Yes |  |  |  |  |  |  | |  | | | | | |  |
|  | | |  |  |  |  |  |  |  | |  | | | | | | |
|  | | | | | | | | | | | |  |  |  |  |  |  |
|  | Respondent: no I don’t think all am just trying to figure out what would be that big or that really important one that would be important to return, I think based on the areas I have worked I really if I have that clear one because other than what it would be looking for if any thing else shows up and its important that’s of course will be recorded in the process of the study and am not sure what kind of the incidental findings | | | | | | | | | | | | | | | |  |
|  |  | | | | | | | | | | | | | | | |  |
|  | | | **Files\\Male\\R-005.male** | | | | | | | | | | | | | |  |
|  |  |  | Yes |  |  |  |  |  |  | |  | | | | | |  |
|  | | |  |  |  |  |  |  |  | |  | | | | | | |
|  | | | | | | | | | | | |  |  |  |  |  |  |
|  | Respondent: Probably yes, but I don’t know what they use them | | | | | | | | | | | | | | | |  |
|  |  | | | | | | | | | | | | | | | |  |
|  | | | | | | | | | | | |  |  |  |  |  |  |
|  | Respondent: If it was me? I would return them, but with caution because you might be looking for something else and then what you get may have a…….depending on the implication of that incidental finding anyway, it has to be weighed. That’s the only thing I can say | | | | | | | | | | | | | | | |  |
|  |  | | | | | | | | | | | | | | | |  |
|  | | | | | | | | | | | |  |  |  |  |  |  |
|  | Respondent: Risks, what are the risks involved, for example, am doing my research on breast cancer, and then I discover that I have some controls who are supposed to be normal, I have these getting blood samples, biopsies, and then I discover that the people who are supposed to be normal, they have this mutation with probability of someone having breast cancer, and the person is so far still okay, am I going to just say that please you have this mutation? No I will not do that. Why? It doesn’t mean that mutation has actually….it has to be weighed on the probability of what is going to come out before I actually communicate it. If the risk is so high, if I have a 50% risk and then communicating it may end up causing the other person loose hope, and the psychology involved may be more deleterious than the communication am making, unless it is so high that if I don’t communicate it the risks are so much, then that is when I will communicate | | | | | | | | | | | | | | | |  |
|  |  |  |  |  |  |  |  |  |  |  |  |  |  |  |  |  |  |
|  |  | | | | | | | | | | | | | | | |  |
|  | | | | | | | | | | | |  |  |  |  |  |  |
|  | Respondent: For me basically it is about the risk to the participant | | | | | | | | | | | | | | | |  |
|  |  | | | | | | | | | | | | | | | |  |
|  | | | **Files\\Male\\R-006. male** | | | | | | | | | | | | | |  |
|  |  |  | Yes |  |  |  |  |  |  | |  | | | | | |  |
|  | | |  |  |  |  |  |  |  | |  | | | | | | |
|  | | | | | | | | | | | |  |  |  |  |  |  |
|  | So you say that if the ethics body doesn't approve then you cannot disseminate.  RESPONDENT:  Yes, because i believe the ethics bodies are in place to protect the right of research participants. | | | | | | | | | | | | | | | |  |
|  |  | | | | | | | | | | | | | | | |  |
| Reports\\Coding Summary By Code Report | | | | | | | | | | Page 53 of 109 | | | | | | | |
|  | | | | | | | | | | | | | | | | | |
|  | | | **Aggregate** |  | **Classification** |  | **Coverage** |  | **Number Of Coding References** | |  | **Reference Number** |  | **Coded By Initials** |  | **Modified On** |  |
|  | | | | | | | | | | | | | | | | | |
|  | | | | | | | | | | | |  |  |  |  |  |  |
|  | I believe if they’re affecting the individual in that moment, or if it is expected that they might affect the individual ,or the community in the near future, if it might lead to something significant in future in terms of ill health . | | | | | | | | | | | | | | | |  |
|  |  | | | | | | | | | | | | | | | |  |
|  | | | | | | | | | | | |  |  |  |  |  |  |
|  | So if someone is to develop a guidance for that one, how would it be ,its like when you get the incidental findings , you go back to the REC , or would there be a possibility in the informed consent form where you say, we are going to do this study but if we find something significant that might not necessarily related to this, we might get back to you.  RESPONDENT:  it sounds like a good and acceptable approach so if it is already pre-introduced in the consent form and the ethics have already approved, then absolutely. then you would be able to think at that point you can actually go back to the community and... But, still my suggestion would be that before this kind of information is taken back to the communities, a very clear assessment of the impact should be established because genetics do not always lead to Phenopic presentation, so might see a mutation somewhere- so you have to establish whether what you are observing is responsible for anything that would be impactful to individuals or to the communities before you go back to raise, to scare people, I think its better to establish the actual impact. | | | | | | | | | | | | | | | |  |
|  |  |  |  |  |  |  |  |  |  |  |  |  |  |  |  |  |  |
|  |  | | | | | | | | | | | | | | | |  |
|  | | | **Files\\Male\\R-007 male** | | | | | | | | | | | | | |  |
|  |  |  | Yes |  |  |  |  |  |  | |  | | | | | |  |
|  | | |  |  |  |  |  |  |  | |  | | | | | | |
|  | | | | | | | | | | | |  |  |  |  |  |  |
|  | Qn 3: INTERVIEWER: and incase of incidental findings, who handles such results?  RESPONDENT: So what do you mean by incidental findings for example?  INTERVIEWER: We went to, or we did this study to find out the kinetics of this particular drug in this individual, but as you do the simple thing you discover that the genome has maybe other important findings, they’re not related to your objective but since you do screening and you get the whole genome you’ve discovered there is something of significance to an individual or to your research participants. Do you report those ones, do you return those to the individuals?  RESPONDENT: Well, ahaa you can publish, and you, of course as I said it is all in the identified data and you shouldn’t be able to particularly know who is who, ahaa so for the start that can be published and people can find out about it. When it comes to returning to the individual, we again go back to the same situation we were in before which is really, you have to think carefully about what’s the implication to the individual, again family and society. | | | | | | | | | | | | | | | |  |
|  |  |  |  |  |  |  |  |  |  |  |  |  |  |  |  |  |  |
|  |  | | | | | | | | | | | | | | | |  |
|  | | | **Files\\Male\\R-009.male** | | | | | | | | | | | | | |  |
|  |  |  | Yes |  |  |  |  |  |  | |  | | | | | |  |
|  | | |  |  |  |  |  |  |  | |  | | | | | | |
|  | | | | | | | | | | | |  |  |  |  |  |  |
|  | INTERVIEWER: so how would you prepare for such issues for example in the consent form or in the protocol development stage because if you didn’t tell me you are going to look for this then you come back to me and turn around and tell me this, how should it be presented?  RESPONDENT: I think it is a difficult situation question because as researchers you have to see if you tell the participants that I am going to look for something but even me I don’t know what, so there could a chance that I don’t know that there could be results I didn’t expect to find but, in that case, I will come and tell you. That situation I think still you can disclose it to the participants that the technique am going to use has a capability of getting more information and it is exactly what am looking for which is the case and therefore in that case if there is something useful we will let you know, I think it can be put in the consent | | | | | | | | | | | | | | | |  |
|  |  |  |  |  |  |  |  |  |  |  |  |  |  |  |  |  |  |
|  |  | | | | | | | | | | | | | | | |  |
|  | | | | | | | | | | | | | | | | | |
|  | | | | | | | | | | | | | | | | | |
| Reports\\Coding Summary By Code Report | | | | | | | | | | Page 54 of 109 | | | | | | | |
|  | | | | | | | | | | | | | | | | | |
|  | | | **Aggregate** |  | **Classification** |  | **Coverage** |  | **Number Of Coding References** | |  | **Reference Number** |  | **Coded By Initials** |  | **Modified On** |  |
|  | | | **Files\\Male\\R-011.male** | | | | | | | | | | | | | |  |
|  |  |  | Yes |  |  |  |  |  |  | |  | | | | | |  |
|  | | |  |  |  |  |  |  |  | |  | | | | | | |
|  | | | | | | | | | | | |  |  |  |  |  |  |
|  | Incidental findings; I think it just depend, i think a lot of these things really, there is always a REC that reviews to see if what you are doing is ethical, if really there are some findings, i guess its not me as a research to decide if should release it, there’s somebody to decide the potential outcome of release of such results. Then there some communities that participate in genetic research, maybe this study is concerned with finding out their ancestry and migration patterns, and then somewhere along the way they find out oh actually what we know about this population, based on the genetic information is different from what is known, Group A ,occupied this land before Group B. Reporting such information to this community creates now land conflicts, so reporting such things is not for us, i wouldn’t say it’s for me to do, its really, i would say we have to be sensitive about the societies that are there | | | | | | | | | | | | | | | |  |
|  |  |  |  |  |  |  |  |  |  |  |  |  |  |  |  |  |  |
|  |  | | | | | | | | | | | | | | | |  |
|  | | | **Files\\Male\\R-013.male** | | | | | | | | | | | | | |  |
|  |  |  | Yes |  |  |  |  |  |  | |  | | | | | |  |
|  | | |  |  |  |  |  |  |  | |  | | | | | | |
|  | | | | | | | | | | | |  |  |  |  |  |  |
|  | RESPONDENT: I think the incidental findings are best to be handled through the clinicians seeing the patients so if at all your in a hospital setting and then you see a certain thing that could be a problem you need to consult the clinician lab however in many cases it is also good to trace back the patients to report the information directly yes depending on exactly what you have found because certain information could be really having an impact on the patient so you might need to track down the patient or the participant too, to inform them of such outcomes | | | | | | | | | | | | | | | |  |
|  |  | | | | | | | | | | | | | | | |  |
|  | | | | | | | | | | | |  |  |  |  |  |  |
|  | RESPONDENT: I think there is need for counselling to prepare these patients for the possible outcome of the study when your detecting for certain infections or conditions that are public concern you might need to prepare these participants and it should be part of the information you provide in your consent such that they are already aware that this could be  INTERVIEWER: some may not be related to health, may have social impacts, it could be paternity so we need to know so still you need to have a counsellor?  RESPONDENT: yes | | | | | | | | | | | | | | | |  |
|  |  | | | | | | | | | | | | | | | |  |
|  | | | **Files\\Male\\R-014 male** | | | | | | | | | | | | | |  |
|  |  |  | Yes |  |  |  |  |  |  | |  | | | | | |  |
|  | | |  |  |  |  |  |  |  | |  | | | | | | |
|  | | | | | | | | | | | |  |  |  |  |  |  |
|  | RESPONDENT: what do you mean incidental because for us, we are targeted, we are looking for schisto, we have a scope approved by IRB  INTERVIEWER: what if something presented itself along the way and the information you get is important, it has far reaching consequences…  RESPONDENT: that is a common question I think even at IRB.  INTERVIEWER: they usually ask you? | | | | | | | | | | | | | | | |  |
|  |  | | | | | | | | | | | | | | | |  |
|  | | | **Files\\Male\\R-017. male** | | | | | | | | | | | | | |  |
|  |  |  | Yes |  |  |  |  |  |  | |  | | | | | |  |
|  | | |  |  |  |  |  |  |  | |  | | | | | | |
|  | | | | | | | | | | | |  |  |  |  |  |  |
|  | Respondent: Like I said, you just have to go through the procedure of getting back, because incidental findings some of them might be fatal, and it’s a life and death situation, we don’t have them in our studies but there are studies which are like that, its life and death, and you have to go through the procedure of the hospital where you got it from, inform them and then they also have their unique procedures how they actually get to the individual who is affected, but me the researcher I cant directly interface or get a person’s number and tell them that you, you have this | | | | | | | | | | | | | | | |  |
|  |  | | | | | | | | | | | | | | | |  |
| Reports\\Coding Summary By Code Report | | | | | | | | | | Page 55 of 109 | | | | | | | |
|  | | | | | | | | | | | | | | | | | |
|  | | | **Aggregate** |  | **Classification** |  | **Coverage** |  | **Number Of Coding References** | |  | **Reference Number** |  | **Coded By Initials** |  | **Modified On** |  |
|  | | | | | | | | | | | | | | | | | |
|  | | | | | | | | | | | |  |  |  |  |  |  |
|  | Respondent: We would advise the hospital to have that kind of procedure, depending on the kind of research we are doing | | | | | | | | | | | | | | | |  |
|  |  | | | | | | | | | | | | | | | |  |
|  | | | **Files\\Male\\R-018.male** | | | | | | | | | | | | | |  |
|  |  |  | Yes |  |  |  |  |  |  | |  | | | | | |  |
|  | | |  |  |  |  |  |  |  | |  | | | | | | |
|  | | | | | | | | | | | |  |  |  |  |  |  |
|  | Respondent: Well I must admit have not done so much into feeding back of results right now, but that is our plan and we want to give those results that for which there are remedies, so may be my colleague would be in a better position to talk to us | | | | | | | | | | | | | | | |  |
|  |  | | | | | | | | | | | | | | | |  |
|  | | | **Files\\Male\\R-022. male** | | | | | | | | | | | | | |  |
|  |  |  | Yes |  |  |  |  |  |  | |  | | | | | |  |
|  | | |  |  |  |  |  |  |  | |  | | | | | | |
|  | | | | | | | | | | | |  |  |  |  |  |  |
|  | RESPONDENT: you know any research has a working group, so, whatever findings you get, unless you know the implications, these need to be discussed by the study working group e.g. clinical trials have a “data and safety management board” that frequently assesses results and will make a decision on which is beneficial and not and which should be reported. So, there has to be a lot of consultation. We also work with RECs and other regulatory bodies including UNCST. | | | | | | | | | | | | | | | |  |
|  |  | | | | | | | | | | | | | | | |  |
|  | | | | | | | | | | | |  |  |  |  |  |  |
|  | RESPONDENT: that is the importance of counseling; you are doing this interview, the consent for, you have given me this information. What is very important is really explaining; the situation, what is anticipated, the possible risks; you really have to give the information, you don’t have to hide any information. You really need to educate people so that they know what you are really talking about, what they should anticipate. If there are risks you tell them. So, I think what is very important is to give as much information as possible | | | | | | | | | | | | | | | |  |
|  |  | | | | | | | | | | | | | | | |  |
|  | | | | | | | | | | | |  |  |  |  |  |  |
|  | RESPONDENT: it depends because for example in the area where I am working with patients, if someone is going for an HIV test, you explain to them what HIV is, you assess what risks these people have and you anticipate possibly the positive and negative, explain to them what it means being positive and negative for your entire life, it will mean you will have treatment for the rest of your life, it will mean there are risks of taking medication, the side effects, what it means socially and households what it means for their lifestyle. So, really you are preparing this individual at all stages. So, again depending on the outcome of every test you explain to them; now you are going to get onto medication, this is what you should anticipate, this is what you should do. So, it is a whole process right from the start. | | | | | | | | | | | | | | | |  |
|  |  | | | | | | | | | | | | | | | |  |
|  | | | **Files\\Male\\R-023. male** | | | | | | | | | | | | | |  |
|  |  |  | Yes |  |  |  |  |  |  | |  | | | | | |  |
|  | | |  |  |  |  |  |  |  | |  | | | | | | |
|  | | | | | | | | | | | |  |  |  |  |  |  |
|  | Respondent: Well for me incidental findings, I prefer communicating when am sure, and when something has been verified by other people, if I just came up with an incidental finding during my study, its good I can point it out, but I would be more comfortable making a big deal of it if it has been replicated by atleast some other group that is not me, so in the timeline for dealing with incidental findings for me, one should be cautious, unless it is a life threatening event that one would error on the side of being wrong than right but if its not, that type, I would suggest it is handled like all the other things, go through peer review, let some people replicate it and then when you are really sure, as opposed to just rushing | | | | | | | | | | | | | | | |  |
|  |  | | | | | | | | | | | | | | | |  |
|  | | | | | | | | | | | | | | | | | |
| Reports\\Coding Summary By Code Report | | | | | | | | | | Page 56 of 109 | | | | | | | |
|  | | | | | | | | | | | | | | | | | |
|  | | | **Aggregate** |  | **Classification** |  | **Coverage** |  | **Number Of Coding References** | |  | **Reference Number** |  | **Coded By Initials** |  | **Modified On** |  |
|  | | | | | | | | | | | | | | | | | |
|  | | | | | | | | | | | |  |  |  |  |  |  |
|  | Respondent: Yeah that’s why it should be more than just the researchers themselves, it shouldn’t be only at the discretion of them, I think it should be, again depending on the side of nature of the study, some studies have a community advisory board, you have other advisors as part of the research, so all those can come together and help put, another opinion to the thing. So to me it needs to be more consulted widely I should say, involve more people maybe, involved in the implementation of the policies, etc like a wider group, not just the researchers. Most researchers are more focused on the data and getting the results, doing our publications and manuscripts and we rarely think beyond that there could be other important things that could be at the fore front | | | | | | | | | | | | | | | |  |
|  |  | | | | | | | | | | | | | | | |  |
|  | | | **Files\\Male\\R-024.male** | | | | | | | | | | | | | |  |
|  |  |  | Yes |  |  |  |  |  |  | |  | | | | | |  |
|  | | |  |  |  |  |  |  |  | |  | | | | | | |
|  | | | | | | | | | | | |  |  |  |  |  |  |
|  | Respondent: So that is not a rare situation, I have had to look at this thing all over again, I have not had a level of preparedness that I say that in case of incidental findings this is what I do. I have had to do this on a case by case basis for instance when we first discovered a child with sickle cell, a condition called sickle cell variant O, it was so rare and then how to say, how do you now say O because O means one of the parents has a deletion, completely there is nothing missing and the child inherited it, so it is a zero inheritance on one side and then with the trait on the other side giving you that and it is so difficult because now the parent who has got a deletion is normal and get anything in them. And when we first got that it was so difficult and I said do I go back to the IRB, to let them know that this is an incidental finding, and I need to document it separately? But what we’ve done is to go ahead and say to the parents, and tell them that look, we had set out to do this but the same test has also identified this, we didn’t do an extra test, the same test has also recognized this other way and we tell them that this is different from what is normally in the population, we’ve done that. There are few that sickle, they are only 6/2000 but still it is an incidental finding we didn’t expect it in the first place | | | | | | | | | | | | | | | |  |
|  |  |  |  |  |  |  |  |  |  |  |  |  |  |  |  |  |  |
|  |  | | | | | | | | | | | | | | | |  |
|  | | | | | | | | | | | |  |  |  |  |  |  |
|  | Respondent: Yes, when we deliver this result we have the social responsibility to ensure that patients go to social care so they are first referred to the pediatric clinic, so they are being followed up there as cases with that. Secondly we have only kept their contact information should we get additional interest. And then of course for what they originally consented we are going on with that part of the research and then the incidental finding since it was not even part of the research question we just left it up to the point of saying in case we develop interest we are following you up | | | | | | | | | | | | | | | |  |
|  |  | | | | | | | | | | | | | | | |  |
|  | | | **Files\\Male\\R-025 male..** | | | | | | | | | | | | | |  |
|  |  |  | Yes |  |  |  |  |  |  | |  | | | | | |  |
|  | | |  |  |  |  |  |  |  | |  | | | | | | |
|  | | | | | | | | | | | |  |  |  |  |  |  |
|  | Interviewer: So, in case of incidental findings, how do you handle such results?  Respondent: This is going to be difficult, we have been discussing it, we have been brainstorming on what to do with incidental findings we have not come up with a conclusive procedure. What we have just done is that if we think that that incidental finding affects the individual very much we just have to inform them, through counseling inform you and since ours is directed towards cancer, and offer him or link him to a nearby hospital for screening but it is just awareness, nothing much more | | | | | | | | | | | | | | | |  |
|  |  | | | | | | | | | | | | | | | |  |
|  | | | | | | | | | | | |  |  |  |  |  |  |
|  | Interviewer: So under what conditions do you or would you return such incidental findings?  Respondent: If you think that your finding is most likely to cause a disease, if you most likely think that your finding is going to lead to a certain disease, then surely that individual must be under surveillance | | | | | | | | | | | | | | | |  |
|  |  | | | | | | | | | | | | | | | |  |
|  | | | | | | | | | | | | | | | | | |
| Reports\\Coding Summary By Code Report | | | | | | | | | | Page 57 of 109 | | | | | | | |
|  | | | | | | | | | | | | | | | | | |
|  | | | **Aggregate** |  | **Classification** |  | **Coverage** |  | **Number Of Coding References** | |  | **Reference Number** |  | **Coded By Initials** |  | **Modified On** |  |
|  | **Nodes\\Return of results\\Opinion on return of results\Negative** | | | | | | | | | | | | | | | |  |
|  | | **Document** | | | | | | | | | | | | | | |  |
|  | | | **Files\\Female\\R-003.female** | | | | | | | | | | | | | |  |
|  |  |  | Yes |  |  |  |  |  |  | |  | | | | | |  |
|  | | |  |  |  |  |  |  |  | |  | | | | | | |
|  | | | | | | | | | | | |  |  |  |  |  |  |
|  | INTERVIEWER: do you think it is necessary to return genetics results to individuals who have participated in the genetics studies?  RESPONDENT: I think it is a yes and no, it is good if these individuals have really been given basic information for them to understand you know one of the things I have realized is that because of literacy, there are things which are difficult to explain but for me I know if it is beneficial really if it is not going to, its good for people to know because why would some one spend time in a study and then you get the information and you sit on it, if it is benefial certainly these individuals need to know but the other side of the coin is what happens if this is information that cannot be accepted for instance if it is a couple because it can also cause conflict in a home so it is like a fifty fifty but all the same I feel that individuals need to know | | | | | | | | | | | | | | | |  |
|  |  |  |  |  |  |  |  |  |  |  |  |  |  |  |  |  |  |
|  |  | | | | | | | | | | | | | | | |  |
|  | | | | | | | | | | | |  |  |  |  |  |  |
|  | INTERVIEWER: so, it shouldn’t be done  RESPONDENT: suppose the community is carrying a bad gene, in a sense that it is a gene that is related to obesity but is something that is ok but am trying to think of something weird maybe the tendency of, these days people tell us that lesbianism and gay is kind of runs in, there is some genetic inclination of, suppose they say that all those people in that community  INTERVIEWER: because when you return aggregate results is to maybe all the team or individuals in that community who have participated not that the outside community and probably when you return them it could help them plan or think about them, there is a way they can intervene some of them have no specific intervene or treatment that can help you understand your situation or adjust the environment so that it will not come back  RESPONDENT: may be | | | | | | | | | | | | | | | |  |
|  |  |  |  |  |  |  |  |  |  |  |  |  |  |  |  |  |  |
|  |  | | | | | | | | | | | | | | | |  |
|  | | | **Files\\Female\\R027.female** | | | | | | | | | | | | | |  |
|  |  |  | Yes |  |  |  |  |  |  | |  | | | | | |  |
|  | | |  |  |  |  |  |  |  | |  | | | | | | |
|  | | | | | | | | | | | |  |  |  |  |  |  |
|  | INTERVIEWER: You could not go back and give because you had this information that you are more susceptible or you may get more severe reactions compared to may be someone with another gene or something like that?  RESPONDENT: We could have but we didn’t. it is very scientific that some of those things don’t make sense to even scientists. | | | | | | | | | | | | | | | |  |
|  |  | | | | | | | | | | | | | | | |  |
|  | | | **Files\\Female\\R028.female** | | | | | | | | | | | | | |  |
|  |  |  | Yes |  |  |  |  |  |  | |  | | | | | |  |
|  | | |  |  |  |  |  |  |  | |  | | | | | | |
|  | | | | | | | | | | | |  |  |  |  |  |  |
|  | INTERVIEWER: So, in case of incidental findings, how do you handle them  RESPONDENT: Hmmm, well I don’t believe in opening unopened boxes | | | | | | | | | | | | | | | |  |
|  |  | | | | | | | | | | | | | | | |  |
|  | | | | | | | | | | | | | | | | | |
|  | | | | | | | | | | | | | | | | | |
| Reports\\Coding Summary By Code Report | | | | | | | | | | Page 58 of 109 | | | | | | | |
|  | | | | | | | | | | | | | | | | | |
|  | | | **Aggregate** |  | **Classification** |  | **Coverage** |  | **Number Of Coding References** | |  | **Reference Number** |  | **Coded By Initials** |  | **Modified On** |  |
|  | | | | | | | | | | | | | | | | | |
|  | | | | | | | | | | | |  |  |  |  |  |  |
|  | INTERVIEWER: Okay now for example you said that you link your findings to the ethnic group of participants, so you realize sometimes that the alleged father of that child is not the real father, how would you handle such a case?  RESPONDENT: Well I did not come across such a case but we found incompatibilism between the mother and the child, like definitely where this mother can’t be the mother of that baby. But usually is the first issue being that there were mixing up of samples in the laboratory. And it is actually, I know because I did my PhD at Cambridge, we had a few cases here but it was not even rare in the UK or US to happen, and even but I think they say there is even an acceptable percentage. Those studies where you have mother-baby incompatible, but remember I don’t have the father’s DNA. So, what I have is mother, but I can be able to work out whether this mother is really the mother, I don’t know the father, so I didn’t really have to deal with paternity issues because I only had mother. So, assuming whatever is not for the mother is for the father whom I didn’t know. So that is what makes it a big difference. If you know both DNA, father, mother and baby, then you are likely to face those challenges but even if you did, those samples we got we attributed them to a mix up and of course some are not analyzed further. For example if a woman has blood group BB, and her baby is OO, definitely that is not her baby but at that moment, when you are seeing this 6 months, probably when the baby is up and running, and you are in some lab in the UK and you are saying there is incompatibility here, so one thing I know is that some people have done studies where paternity has been a challenge, but one thing I know is that they are not obliged to give that information. Am an obstetrician, many times we do know that a certain man who is busy around the woman is not the father of the child but it is not our role, the best we tell the woman is that I think you are playing with fire, you need to deal with the situation, but we do not feel obliged that this woman is lying to you. So even if I had known that paternity was wrong, I was not obliged because of incidental findings to get them, the only thing you can ask is the person who knows. First of all, if both don’t know, that is their business, but what we also used to get information about ethnicity, we would then get to know, some times the woman would tell you that that man is not my husband, he’s not the father of this pregnancy. That information we have sometimes, but she would go on to tell us through ethnicity but we never though obliged to tell them  INTERVIEWER: Okay if you found that a patient was at risk, for example had a gene exposing them to a certain risk of cancer and may be there is something they can do about it….  RESPONDENT: That is why I said if there is anything you can do about it. But by the way remember, I know you have talked to many genetic researchers, when you are looking for genes, depending on what you are looking for, you are looking for your genes not all the genes, so it is not easy to come up with incidental findings because you are not scanning the entire genome, some of course are, the genome wide, but the kind I was doing, I was specific, all my focus was on a particular loci which makes the difference, but when you go looking for everything, then you are likely to come up with those issues | | | | | | | | | | | | | | | |  |
|  |  |  |  |  |  |  |  |  |  |  |  |  |  |  |  |  |  |
|  |  |  |  |  |  |  |  |  |  |  |  |  |  |  |  |  |  |
|  |  |  |  |  |  |  |  |  |  |  |  |  |  |  |  |  |  |
|  |  | | | | | | | | | | | | | | | |  |
|  | | | **Files\\Female\\R029 female** | | | | | | | | | | | | | |  |
|  |  |  | Yes |  |  |  |  |  |  | |  | | | | | |  |
|  | | |  |  |  |  |  |  |  | |  | | | | | | |
|  | | | | | | | | | | | |  |  |  |  |  |  |
|  | INTERVIEWER: What if it has not impact on their lives would it be good not to communicate it?  RESPONDENT: if they have no impact on their lives like what kind of scenario would have no impact? | | | | | | | | | | | | | | | |  |
|  |  | | | | | | | | | | | | | | | |  |
|  | | | **Files\\Male\\R-007 male** | | | | | | | | | | | | | |  |
|  |  |  | Yes |  |  |  |  |  |  | |  | | | | | |  |
|  | | |  |  |  |  |  |  |  | |  | | | | | | |
|  | | | | | | | | | | | |  |  |  |  |  |  |
|  | Respondent: Ahm, in terms of their genomics, ahm, at the moment I think it’s too risky, I don’t think its necessary at the moment, No. If I know that this kind of gene, XZY gene makes somebody more susceptible to XZY and , at the it doesn’t require me particularly to return back because I think the implications are still, well, the field is still very virgin I really wouldn’t want to do that kind of thing at the moment. I would want a general. | | | | | | | | | | | | | | | |  |
|  |  | | | | | | | | | | | | | | | |  |
|  | | | | | | | | | | | | | | | | | |
|  | | | | | | | | | | | | | | | | | |
| Reports\\Coding Summary By Code Report | | | | | | | | | | Page 59 of 109 | | | | | | | |
|  | | | | | | | | | | | | | | | | | |
|  | | | **Aggregate** |  | **Classification** |  | **Coverage** |  | **Number Of Coding References** | |  | **Reference Number** |  | **Coded By Initials** |  | **Modified On** |  |
|  | | | **Files\\Male\\R-008.male** | | | | | | | | | | | | | |  |
|  |  |  | Yes |  |  |  |  |  |  | |  | | | | | |  |
|  | | |  |  |  |  |  |  |  | |  | | | | | | |
|  | | | | | | | | | | | |  |  |  |  |  |  |
|  | INTERVIEWER: so, they always have that priority, so was your study or is your study of such a nature that you have to report back results? To the participants on what you discover on their genetics  RESPONDENT: I don’t, why would want to know  INTERVIEWER: what do you explain to them  RESPONDENT: you explain to them the study what the study is going to do but we don’t tell them that you’re coming back to tell you what we have found in your gene  INTERVIEWER: why, its not right? Because don’t you think I would want to know if I have that  RESPONDENT: then what?  INTERVIEWER: general curiosity  RESPONDENT: I have told you there is a policy called test and treat so for you do you want to defy that policy? that policy was brought because they realized some people were dying when we tell them go back and they would never come back so every one who is tested is treated. For you when you tell them to come I have realized this is what you were doing then when you tell them they stop taking the medication I think telling them is not right  INTERVIEWER: so, it is not necessary to return, what of if you give a general picture for example you go to a school or a community then you realise that of the people that have participated you give a general like a percentage instead of giving to an individual , you your like this, your stand is this  RESPONDENT: what I am telling you personally there is a publication I have written yes this one I think I have to do this but now the way people process or preserve that information can be dangerous, you may not have the right words to present it in what you want and then they get from others but here we are looking at the long term of the study but the immediate one test then get treatment then get the information about the disease but at the end of the day tell  INTERVIEWER: so, you let them know that probably these results won’t be disseminated to you, you just contributed to the know  RESPONDENT: yeah, they know from science and what | | | | | | | | | | | | | | | |  |
|  |  |  |  |  |  |  |  |  |  |  |  |  |  |  |  |  |  |
|  |  |  |  |  |  |  |  |  |  |  |  |  |  |  |  |  |  |
|  |  |  |  |  |  |  |  |  |  |  |  |  |  |  |  |  |  |
|  |  | | | | | | | | | | | | | | | |  |
|  | | | **Files\\Male\\R-010. male** | | | | | | | | | | | | | |  |
|  |  |  | Yes |  |  |  |  |  |  | |  | | | | | |  |
|  | | |  |  |  |  |  |  |  | |  | | | | | | |
|  | | | | | | | | | | | |  |  |  |  |  |  |
|  | RESPONDENT: I think the decision to return results is partly depends on the sensitivity of the matter and on the consent. That’s why my argument is that it is important that that is included in the consenting process because if a patient has not consented and the matter is controversial and it’s a matter that is not going to take a person’s life then you urge that; maybe No, you are not going to return the results. | | | | | | | | | | | | | | | |  |
|  |  | | | | | | | | | | | | | | | |  |
|  | | | **Files\\Male\\R-012.male** | | | | | | | | | | | | | |  |
|  |  |  | Yes |  |  |  |  |  |  | |  | | | | | |  |
|  | | |  |  |  |  |  |  |  | |  | | | | | | |
|  | | | | | | | | | | | |  |  |  |  |  |  |
|  | RESPONDENT: I don’t think I would return results but the generalized results yes, not the individual results in that way yes | | | | | | | | | | | | | | | |  |
|  |  | | | | | | | | | | | | | | | |  |
|  | | | **Files\\Male\\R-014 male** | | | | | | | | | | | | | |  |
|  |  |  | Yes |  |  |  |  |  |  | |  | | | | | |  |
|  | | |  |  |  |  |  |  |  | |  | | | | | | |
|  | | | | | | | | | | | |  |  |  |  |  |  |
|  | RESPONDENT: negative or positive | | | | | | | | | | | | | | | |  |
|  |  | | | | | | | | | | | | | | | |  |
| Reports\\Coding Summary By Code Report | | | | | | | | | | Page 60 of 109 | | | | | | | |
| 11/11/2020 1:42 PM | | | | | | | | | | | | | | | | | |
|  | | | **Aggregate** |  | **Classification** |  | **Coverage** |  | **Number Of Coding References** | |  | **Reference Number** |  | **Coded By Initials** |  | **Modified On** |  |
|  | | | **Files\\Male\\R-017. male** | | | | | | | | | | | | | |  |
|  |  |  | Yes |  |  |  |  |  |  | |  | | | | | |  |
|  | | |  |  |  |  |  |  |  | |  | | | | | | |
|  | | | | | | | | | | | |  |  |  |  |  |  |
|  | Respondent: I am not obliged to follow up an individual, and am not mandated to identify an individual in my research because one thing that happens to the data as we get it is that we index that data, anonymize the individual’s identity so that I can’t easily trace that this young girl in this village who is affected by this. So, I try to make sure that even if am disseminating this information to an outside collaborator, they do not know the actual identity of this individual | | | | | | | | | | | | | | | |  |
|  |  | | | | | | | | | | | | | | | |  |
|  | | | **Files\\Male\\R-022. male** | | | | | | | | | | | | | |  |
|  |  |  | Yes |  |  |  |  |  |  | |  | | | | | |  |
|  | | |  |  |  |  |  |  |  | |  | | | | | | |
|  | | | | | | | | | | | |  |  |  |  |  |  |
|  | RESPONDENT: not everything you see could be beneficial to the patients, certain things are entirely for research but in our setting, we are testing specifically for patients and that’s why it is important. | | | | | | | | | | | | | | | |  |
|  |  | | | | | | | | | | | | | | | |  |
|  | **Nodes\\Return of results\\Opinion on return of results\positive** | | | | | | | | | | | | | | | |  |
|  | | **Document** | | | | | | | | | | | | | | |  |
|  | | | **Files\\Female\\R-003.female** | | | | | | | | | | | | | |  |
|  |  |  | No |  |  |  |  |  |  | |  | | | | | |  |
|  | | |  |  |  |  |  |  |  | |  | | | | | | |
|  | | | | | | | | | | | |  |  |  |  |  |  |
|  | INTERVIEWER: do you think it is necessary to return genetics results to individuals who have participated in the genetics studies?  RESPONDENT: I think it is a yes and no, it is good if these individuals have really been given basic information for them to understand you know one of the things I have realized is that because of literacy, there are things which are difficult to explain but for me I know if it is beneficial really if it is not going to, its good for people to know because why would some one spend time in a study and then you get the information and you sit on it, if it is benefial certainly these individuals need to know but the other side of the coin is what happens if this is information that cannot be accepted for instance if it is a couple because it can also cause conflict in a home so it is like a fifty fifty but all the same I feel that individuals need to know | | | | | | | | | | | | | | | |  |
|  |  |  |  |  |  |  |  |  |  |  |  |  |  |  |  |  |  |
|  |  | | | | | | | | | | | | | | | |  |
|  | | | **Files\\Female\\R027.female** | | | | | | | | | | | | | |  |
|  |  |  | No |  |  |  |  |  |  | |  | | | | | |  |
|  | | |  |  |  |  |  |  |  | |  | | | | | | |
|  | | | | | | | | | | | |  |  |  |  |  |  |
|  | INTERVIEWER: So doctor still on that, what do you have to say about sharing of benefits of the research findings with the community for example; there is this study that has come and there are the research and may be twenty years later there is that drug for those who participated?  RESPONDENT: They should get it for free. | | | | | | | | | | | | | | | |  |
|  |  | | | | | | | | | | | | | | | |  |
|  | | | | | | | | | | | |  |  |  |  |  |  |
|  | RESPONDENT: Since you are talking about returning results  INTERVIEWER: It will influence issues and some of these guidelines will be incorporated in. | | | | | | | | | | | | | | | |  |
|  |  | | | | | | | | | | | | | | | |  |
| Reports\\Coding Summary By Code Report | | | | | | | | | | Page 61 of 109 | | | | | | | |
|  | | | | | | | | | | | | | | | | | |
|  | | | **Aggregate** |  | **Classification** |  | **Coverage** |  | **Number Of Coding References** | |  | **Reference Number** |  | **Coded By Initials** |  | **Modified On** |  |
|  | | | **Files\\Male\\R-006. male** | | | | | | | | | | | | | |  |
|  |  |  | No |  |  |  |  |  |  | |  | | | | | |  |
|  | | |  |  |  |  |  |  |  | |  | | | | | | |
|  | | | | | | | | | | | |  |  |  |  |  |  |
|  | so i asked that question because ,i think if someone has a pathogen that has genetically changed its structure and now cannot respond to the treatment that a person is getting, it would be important to tell this person that we have to change this treatment because your infection is resistant to the treatment you were on because some time when that happens you have to give treatment that has maybe more side effects or a drug that’s more expensive ,so you have to tell this person why you are giving this treatment. | | | | | | | | | | | | | | | |  |
|  |  | | | | | | | | | | | | | | | |  |
|  | | | **Files\\Male\\R-009.male** | | | | | | | | | | | | | |  |
|  |  |  | No |  |  |  |  |  |  | |  | | | | | |  |
|  | | |  |  |  |  |  |  |  | |  | | | | | | |
|  | | | | | | | | | | | |  |  |  |  |  |  |
|  | RESPONDENT: there are results which could make meaning for example if our hunt for genetic markers or genes which lead to rapid progression to HIV so we could if we found such genes and then we went and we knew who had genes in this population and for example if something could be done towards that then it makes a lot of sense to go back and tell such participants especially if there is some thing which could be done that is like in sicklers like I told you those really are either beneficial either prognostically or if something could be done | | | | | | | | | | | | | | | |  |
|  |  | | | | | | | | | | | | | | | |  |
|  | | | **Files\\Male\\R-013.male** | | | | | | | | | | | | | |  |
|  |  |  | No |  |  |  |  |  |  | |  | | | | | |  |
|  | | |  |  |  |  |  |  |  | |  | | | | | | |
|  | | | | | | | | | | | |  |  |  |  |  |  |
|  | RESPONDENT: us we recommend to the clinicians upon seeing the patients because then if I see this I should evaluate for signs of resistance like raising PSA then recurrence of the tumor size and all that and may be any signs of worsening of the disease those could show the clinical signs of resistance in the chemotherapy otherwise this is not something that we can just enforce for change of therapy so we need to recommend clinicians in the course to make sure that they see it fit to follow up these patients more closely the ones that could be with the signs | | | | | | | | | | | | | | | |  |
|  |  | | | | | | | | | | | | | | | |  |
|  | | | | | | | | | | | |  |  |  |  |  |  |
|  | RESPONDENT: very important , to return results to patients however it depends on the kind of study that you are doing most genetics studies here currently the one we do, don’t really involve results that are clinically important to the patient for example if you go and detect the kind of the TB genotypes in the population you’re not going to come and tell the TB patient that you have the Ugandan genotype or the berlin genotype because that information is not important to them because yes so what to the patient however you could say to the fellow clinicians that generally some of the patients have the Uganda genotype and this means these patients possibly are always prone to being resistant so to the clinical community it could more sense but individually it is very important that you think also about that kind of data information you’re going to because certain information could not be understandable to the patient and useful to them | | | | | | | | | | | | | | | |  |
|  |  |  |  |  |  |  |  |  |  |  |  |  |  |  |  |  |  |
|  |  | | | | | | | | | | | | | | | |  |
|  | | | **Files\\Male\\R-014 male** | | | | | | | | | | | | | |  |
|  |  |  | No |  |  |  |  |  |  | |  | | | | | |  |
|  | | |  |  |  |  |  |  |  | |  | | | | | | |
|  | | | | | | | | | | | |  |  |  |  |  |  |
|  | RESPONDENT: negative or positive | | | | | | | | | | | | | | | |  |
|  |  | | | | | | | | | | | | | | | |  |
|  | | | | | | | | | | | |  |  |  |  |  |  |
|  | RESPONDENT: that’s why we publish because the biggest limitation for researchers especially if you are working on a post mortem sample is failure to return results. They are like; the patients are dead but if you know you are going to use the dead’s samples, then the people who are still living and those who will live in the future hence publishing data is very important. | | | | | | | | | | | | | | | |  |
|  |  | | | | | | | | | | | | | | | |  |
| Reports\\Coding Summary By Code Report | | | | | | | | | | Page 62 of 109 | | | | | | | |
|  | | | | | | | | | | | | | | | | | |
|  | | | **Aggregate** |  | **Classification** |  | **Coverage** |  | **Number Of Coding References** | |  | **Reference Number** |  | **Coded By Initials** |  | **Modified On** |  |
|  | | | **Files\\Male\\R-017. male** | | | | | | | | | | | | | |  |
|  |  |  | No |  |  |  |  |  |  | |  | | | | | |  |
|  | | |  |  |  |  |  |  |  | |  | | | | | | |
|  | | | | | | | | | | | |  |  |  |  |  |  |
|  | Respondent: Yes, it’s the same was you go and seek medical help and they tell you that they are diagnosing certain things and you expect feed back about the sample that is taken. However, you have to have protocol of how you do this, you have to have special people like councilors who interface with the individuals, be it positive or negative results | | | | | | | | | | | | | | | |  |
|  |  | | | | | | | | | | | | | | | |  |
|  | | | **Files\\Male\\R-018.male** | | | | | | | | | | | | | |  |
|  |  |  | No |  |  |  |  |  |  | |  | | | | | |  |
|  | | |  |  |  |  |  |  |  | |  | | | | | | |
|  | | | | | | | | | | | |  |  |  |  |  |  |
|  | Respondent: Yes, it would be good to report to them what we found in them with respect to susceptibility, but we have not reached that stage, but at the same time there are what we call incidental findings, if we find some genetic effects for which there are remedies, then it becomes our duty to inform them. If you see that for example these days sickle cell carriage is an issue in Uganda. The carriage is rising which means that if a person knows it is more important than never before in Uganda, for people going to marry to know their sickle cell carriage status, so if you find this it would be our duty to inform the people that you see you have this trait, so be careful who you marry, because you might have problems with your kids. But there are those which you can’t tell them, it is something for which there is no remedy, why are you going to worry them? | | | | | | | | | | | | | | | |  |
|  |  |  |  |  |  |  |  |  |  |  |  |  |  |  |  |  |  |
|  |  | | | | | | | | | | | | | | | |  |
|  | | | | | | | | | | | |  |  |  |  |  |  |
|  | the results can help them get some information, like one for sickle cell testing, if you are able to tell some one that you are not a carrier, the mother is a carrier and you have a Sickler, how do you go about that?  Respondent: Depending on how much they know about carriage and whatever, we want to only look at it at the point of a young person who might want to marry some time, that information is most useful to that one rather than the parents | | | | | | | | | | | | | | | |  |
|  |  | | | | | | | | | | | | | | | |  |
|  | | | **Files\\Male\\R-022. male** | | | | | | | | | | | | | |  |
|  |  |  | No |  |  |  |  |  |  | |  | | | | | |  |
|  | | |  |  |  |  |  |  |  | |  | | | | | | |
|  | | | | | | | | | | | |  |  |  |  |  |  |
|  | RESPONDENT: of course it is necessary to report results and if someone has a condition that is going to affect their livelihood, you definitely need to report. And like I mentioned, our reports go directly to the clinicians who are helping out on these patients. | | | | | | | | | | | | | | | |  |
|  |  | | | | | | | | | | | | | | | |  |
|  | | | **Files\\Male\\R-024.male** | | | | | | | | | | | | | |  |
|  |  |  | No |  |  |  |  |  |  | |  | | | | | |  |
|  | | |  |  |  |  |  |  |  | |  | | | | | | |
|  | | | | | | | | | | | |  |  |  |  |  |  |
|  | Respondent: Yes its very necessary and I can tell you of two instances that have social connotations one is a child whom we managed some years ago and that time we referred them for tests but because this was genetic tests the results were brought back to me as clinician at a time and when I gave the parents the results they managed to manage this child smoothly until this year when the child passed on, but the child was in medical school, you see they managed to manage the child up to adulthood so it’s very important and so is for all other conditions that we diagnose we tell parents on how to manage children with thalassemia and then we tell them when they develop an illness what they should do. So, this very useful in terms of public health intervention but also very useful in terms of constructing cohorts of patients who have a condition like that. So, now like we have got like over 2000 well documented cases of sickle cell in the region, we have got like 530 well documented cases with G6PD deficiency like that. So, like we also tend to construct cohorts based on genetic material so it’s very useful for both clinical work and also the patients knowing their status. | | | | | | | | | | | | | | | |  |
|  |  |  |  |  |  |  |  |  |  |  |  |  |  |  |  |  |  |
|  |  | | | | | | | | | | | | | | | |  |
| Reports\\Coding Summary By Code Report | | | | | | | | | | Page 63 of 109 | | | | | | | |
|  | | | | | | | | | | | | | | | | | |
|  | | | **Aggregate** |  | **Classification** |  | **Coverage** |  | **Number Of Coding References** | |  | **Reference Number** |  | **Coded By Initials** |  | **Modified On** |  |
|  | **Nodes\\Return of results\\Recommendations** | | | | | | | | | | | | | | | |  |
|  | | **Document** | | | | | | | | | | | | | | |  |
|  | | | **Files\\Female\\R-003.female** | | | | | | | | | | | | | |  |
|  |  |  | Yes |  |  |  |  |  |  | |  | | | | | |  |
|  | | |  |  |  |  |  |  |  | |  | | | | | | |
|  | | | | | | | | | | | |  |  |  |  |  |  |
|  | INTERVIEWER: so, would it be necessary for example to recommend that every genomics study should have a genetics counsellor?  RESPONDENT: yeah, there should be someone who is at least knowledgeable who understands the terms and who can explain in simple terms the implications of such studies and what happens if indeed to an individual, a family or a community are carrying a certain gene that may not be very good in the eyes of he public so there should be this person who can talk to people | | | | | | | | | | | | | | | |  |
|  |  | | | | | | | | | | | | | | | |  |
|  | | | **Files\\Female\\R-021 female** | | | | | | | | | | | | | |  |
|  |  |  | Yes |  |  |  |  |  |  | |  | | | | | |  |
|  | | |  |  |  |  |  |  |  | |  | | | | | | |
|  | | | | | | | | | | | |  |  |  |  |  |  |
|  | Respondent: I think the general nurses and counselors can do that, the kind of information you are explaining to the person seated there, is not high-tech information, it can be explained by a general nurse or counselor, or even a field worker | | | | | | | | | | | | | | | |  |
|  |  | | | | | | | | | | | | | | | |  |
|  | | | **Files\\Female\\R027.female** | | | | | | | | | | | | | |  |
|  |  |  | Yes |  |  |  |  |  |  | |  | | | | | |  |
|  | | |  |  |  |  |  |  |  | |  | | | | | | |
|  | | | | | | | | | | | |  |  |  |  |  |  |
|  | INTERVIEWER: So, there is no way you could package it to make it simple for them?  RESPONDENT: They could have it but whether it could be useful in future yes, it could be but may be now the one we are about to start is to look at another gene to know if you can tolerate high doses of a certain TB drug depending on your genotype so that you allow that. So that one I will be more useful because that gene can affect many other drugs that are treating hypertension disease. So, if you get heart disease and tell the doctor that I have this genotype they will know that you react better or worse so that one may be more applicable because it extends to many other drugs. So, I guess it depends on what genes you are looking for, if it is a gene for a particular drug it may not be useful but if it extends on the drugs the patient may be on in daily life then it can be useful. | | | | | | | | | | | | | | | |  |
|  |  |  |  |  |  |  |  |  |  |  |  |  |  |  |  |  |  |
|  |  | | | | | | | | | | | | | | | |  |
|  | | | | | | | | | | | |  |  |  |  |  |  |
|  | INTERVIEWER: So now when you feel there is need to report some of those results, would you advise on a way how may be this should be done?  RESPONDENT: In a simplified way as possible, many times we complicate things and we stick to complicated words but we can break it down to something easier then pictorial to slides then people can understand better than just using big words. | | | | | | | | | | | | | | | |  |
|  |  | | | | | | | | | | | | | | | |  |
|  | | | | | | | | | | | |  |  |  |  |  |  |
|  | RESPONDENT: I think I would want to ask the said patient before I put it in the consent. Do you want to know your results, if the results are sensitive, I will explain, you might discover things that are there but I would explain. I wouldn’t just go and tell them, it will distract families that is sensitive that if I discover such things I would put it in the consent and tell the patient before if they want to know or not. Some people don’t want to know, we can’t give information that I didn’t ask for. | | | | | | | | | | | | | | | |  |
|  |  | | | | | | | | | | | | | | | |  |
|  | | | | | | | | | | | | | | | | | |
| Reports\\Coding Summary By Code Report | | | | | | | | | | Page 64 of 109 | | | | | | | |
|  | | | | | | | | | | | | | | | | | |
|  | | | **Aggregate** |  | **Classification** |  | **Coverage** |  | **Number Of Coding References** | |  | **Reference Number** |  | **Coded By Initials** |  | **Modified On** |  |
|  | | | **Files\\Male\\R-001 male** | | | | | | | | | | | | | |  |
|  |  |  | Yes |  |  |  |  |  |  | |  | | | | | |  |
|  | | |  |  |  |  |  |  |  | |  | | | | | | |
|  | | | | | | | | | | | |  |  |  |  |  |  |
|  | I think so, i think that before you really do. like you do HIV/AIDS. There should be some kind of counselling before you actually do these kinds of studies, so the people doing consent should be trained in genetics study counselling because you don't know how people are going to behave when you give them these results. | | | | | | | | | | | | | | | |  |
|  |  | | | | | | | | | | | | | | | |  |
|  | | | **Files\\Male\\R-002. male** | | | | | | | | | | | | | |  |
|  |  |  | Yes |  |  |  |  |  |  | |  | | | | | |  |
|  | | |  |  |  |  |  |  |  | |  | | | | | | |
|  | | | | | | | | | | | |  |  |  |  |  |  |
|  | Respondent: I think it is very important or that’s a one of the reasons I guess most of the research done in our country don’t seem to, the participants don’t seem to get feed back very often we don’t have a clear guideline on how to go about this so if we could have guidelines coming from the authorities on how this kind of information given back to the people would be important so we don’t have any guidelines at least I don’t know of one or probably there could be but I have not found one | | | | | | | | | | | | | | | |  |
|  |  | | | | | | | | | | | | | | | |  |
|  | | | | | | | | | | | |  |  |  |  |  |  |
|  | Respondent: we need to have some guidelines and polices in place especially for like when people do clinical research or studies that goes deep to look at the genetics of individuals in a hope of coming with better treatment options for these people and it means we will need to have kind of technology diagnosis in place which can quickly get us this kind of results and then we are able to tailor the treatment with in the timing of this person to get treatment | | | | | | | | | | | | | | | |  |
|  |  | | | | | | | | | | | | | | | |  |
|  | | | | | | | | | | | |  |  |  |  |  |  |
|  | Respondent: yeah, you’re not really expecting and then you land on this kind of information that is something, so incidental findings I think once you come across such information its important to because research is very determined by some policies ethics issues and stuff like that if it is not clear within the framework currently, I think it is best to may be discuss with the regulators | | | | | | | | | | | | | | | |  |
|  |  | | | | | | | | | | | | | | | |  |
|  | | | **Files\\Male\\R-006. male** | | | | | | | | | | | | | |  |
|  |  |  | Yes |  |  |  |  |  |  | |  | | | | | |  |
|  | | |  |  |  |  |  |  |  | |  | | | | | | |
|  | | | | | | | | | | | |  |  |  |  |  |  |
|  | So if someone is to develop a guidance for that one, how would it be, its like when you get the incidental findings, you go back to the REC , or would there be a possibility in the informed consent form where you say, we are going to do this study but if we find something significant that might not necessarily related to this, we might get back to you.  RESPONDENT:  it sounds like a good and acceptable approach so if it is already pre-introduced in the consent form and the ethics have already approved, then absolutely. then you would be able to think at that point you can actually go back to the community and... But, still my suggestion would be that before this kind of information is taken back to the communities, a very clear assessment of the impact should be established because genetics do not always lead to Phenopic presentation, so might see a mutation somewhere, so you have to establish whether what you are observing is responsible for anything that would be impactful to individuals or to the communities before you go back to raise, to scare people, I think its better to establish the actual impact. | | | | | | | | | | | | | | | |  |
|  |  |  |  |  |  |  |  |  |  |  |  |  |  |  |  |  |  |
|  |  | | | | | | | | | | | | | | | |  |
|  | | | **Files\\Male\\R-009.male** | | | | | | | | | | | | | |  |
|  |  |  | Yes |  |  |  |  |  |  | |  | | | | | |  |
|  | | |  |  |  |  |  |  |  | |  | | | | | | |
|  | | | | | | | | | | | |  |  |  |  |  |  |
|  | INTERVIEWER: do find it necessary to have specialized genetic counselors?  RESPONDENT: I think so there is room for that as these issues are coming up but as of course it requires for people like me and you to develop the guidelines, SOP to really put this knowledge in to context so then that makes a lot of sense that’s were the future is going | | | | | | | | | | | | | | | |  |
|  |  | | | | | | | | | | | | | | | |  |
| Reports\\Coding Summary By Code Report | | | | | | | | | | Page 65 of 109 | | | | | | | |
|  | | | | | | | | | | | | | | | | | |
|  | | | **Aggregate** |  | **Classification** |  | **Coverage** |  | **Number Of Coding References** | |  | **Reference Number** |  | **Coded By Initials** |  | **Modified On** |  |
|  | | | **Files\\Male\\R-010. male** | | | | | | | | | | | | | |  |
|  |  |  | Yes |  |  |  |  |  |  | |  | | | | | |  |
|  | | |  |  |  |  |  |  |  | |  | | | | | | |
|  | | | | | | | | | | | |  |  |  |  |  |  |
|  | RESPONDENT: I agree, recently I was talking to a journalist after the death of the Ugandan business man; they got the son in two weeks’ time asking him who is running the business right now and the son chases them way and one of the journalists stops me and says; you know, I am frustrated, I was writing a story and… I was like; you are being unethical and not being human, this person is still grieving. For me I have always wanted to ask; do some of these social scientists have ethics like do journalists have a specific ethics training!  INTERVIEWER: they don’t (laughs).  RESPONDENT: I think is important because these people do not understand. Let me give you an example; the information we could say of national interest, don’t just run around but somebody wants to make a story. Let me tell you, Western journalists are very sensitive to some of these things | | | | | | | | | | | | | | | |  |
|  |  |  |  |  |  |  |  |  |  |  |  |  |  |  |  |  |  |
|  |  | | | | | | | | | | | | | | | |  |
|  | | | **Files\\Male\\R-011.male** | | | | | | | | | | | | | |  |
|  |  |  | Yes |  |  |  |  |  |  | |  | | | | | |  |
|  | | |  |  |  |  |  |  |  | |  | | | | | | |
|  | | | | | | | | | | | |  |  |  |  |  |  |
|  | well for one, we have to know more about the communities we are studying, unfortunately we tend to focus more on the science. we focus so much on the science, of course it again costs, personnel, money, time to go and study what is sensitive and what is not sensitive. Having such information would be very helpful because it would prevent individuals from recklessly releasing such information. or you would identify, maybe one, group is more likely to be alcoholic, I don't know, maybe those things that are a little sensitive and may affect just releasing them may affect maybe the employer perception towards a certain group. i can think also, the responsibility how we report these findings, although these findings are associated with one group, it’s not that everyone in a particular group fits that profile, maybe its 3%. so it’s our responsibility on how we report our findings so they are not misinterpreted. | | | | | | | | | | | | | | | |  |
|  |  |  |  |  |  |  |  |  |  |  |  |  |  |  |  |  |  |
|  |  | | | | | | | | | | | | | | | |  |
|  | | | **Files\\Male\\R-016. male** | | | | | | | | | | | | | |  |
|  |  |  | Yes |  |  |  |  |  |  | |  | | | | | |  |
|  | | |  |  |  |  |  |  |  | |  | | | | | | |
|  | | | | | | | | | | | |  |  |  |  |  |  |
|  | RESPONDENT: no we have very robust consent form, consent form actually has a lot of things, our consenting process is about one and half hours, we tell the people that you are going to give us your saliva, we are going to look for DNA in that saliva you’re not going to see those results that information that we get from that DNA will be used in the future by scientists usually either to make medication or get treatment. if only we consider that their saliva is part of them, we tell them how we will store that better, who will access the data and for what purpose the data can only be accessed for purposes of medical research nothing else. All the PIs and everybody else who is involved in this research has signed collaborating agreements and documents which with have signed with universities and people who are funding us we know that if don’t not hold those standards we could be sued and go to prison for a long time so we take this as extremely serious we don’t expect that people will play around with this kind of information that can be used to identify individuals not just individuals but communities so we don’t want the world to come and say that there is a particular community in Uganda where the researcher has incidences of a mental illness so something like that so that is one then a stepping step of the consent where we also assess whether the person has understood the consent, the decisional capacity of that person it’s called UBAC university of California Berkley assessment of consent and capacity to consent, university of California on capacity to consent that is what we use the person must ensure capacity to consent, university of Berkley California of capacity to consent | | | | | | | | | | | | | | | |  |
|  |  |  |  |  |  |  |  |  |  |  |  |  |  |  |  |  |  |
|  |  | | | | | | | | | | | | | | | |  |
|  | | | | | | | | | | | | | | | | | |
|  | | | | | | | | | | | | | | | | | |
| Reports\\Coding Summary By Code Report | | | | | | | | | | Page 66 of 109 | | | | | | | |
|  | | | | | | | | | | | | | | | | | |
|  | | | **Aggregate** |  | **Classification** |  | **Coverage** |  | **Number Of Coding References** | |  | **Reference Number** |  | **Coded By Initials** |  | **Modified On** |  |
|  | **Nodes\\Return of results\\Results returned** | | | | | | | | | | | | | | | |  |
|  | | **Document** | | | | | | | | | | | | | | |  |
|  | | | **Files\\Female\\R-003.female** | | | | | | | | | | | | | |  |
|  |  |  | Yes |  |  |  |  |  |  | |  | | | | | |  |
|  | | |  |  |  |  |  |  |  | |  | | | | | | |
|  | | | | | | | | | | | |  |  |  |  |  |  |
|  | RESPONDENT: no, the information which we, well let me say what we did then you will figure out whether what I am saying is addressing your question, what we did after we completed the study the good thing is that I would go to the field quite often so I would update even my research assistance and, we were using Kagando hospital as our base so throughout the study there were kind of following really whenever we would analyse then we would tell them that these are some of the things that we have found out and then finally at the end after the study was completed, I provided a report followed with the permanent script which I took to kagando hospital but not generally to general community we did not have anything like a community meeting for dissemination it was not there | | | | | | | | | | | | | | | |  |
|  |  | | | | | | | | | | | | | | | |  |
|  | | | | | | | | | | | |  |  |  |  |  |  |
|  | INTERVIEWER: but for the individuals who had sickle cells they were given information back  RESPONDENT: back to their home? We did not go back to the homes | | | | | | | | | | | | | | | |  |
|  |  | | | | | | | | | | | | | | | |  |
|  | | | | | | | | | | | |  |  |  |  |  |  |
|  | RESPONDENT: we just gave generalized information | | | | | | | | | | | | | | | |  |
|  |  | | | | | | | | | | | | | | | |  |
|  | | | | | | | | | | | |  |  |  |  |  |  |
|  | INTERVIEWER: have you ever returned genetic results to any community or to any individuals?  RESPONDENT: like back to a home? | | | | | | | | | | | | | | | |  |
|  |  | | | | | | | | | | | | | | | |  |
|  | | | | | | | | | | | |  |  |  |  |  |  |
|  | INTERVIEWER: back to the participants, it could be to participants to a hospital or a community  RESPONDENT: not to specific individuals like I have already said that these results were really summarized in the sense that we were just saying that ok comparing this region in Mbarara will have these so many children who are having this trait | | | | | | | | | | | | | | | |  |
|  |  | | | | | | | | | | | | | | | |  |
|  | | | **Files\\Female\\R-021 female** | | | | | | | | | | | | | |  |
|  |  |  | Yes |  |  |  |  |  |  | |  | | | | | |  |
|  | | |  |  |  |  |  |  |  | |  | | | | | | |
|  | | | | | | | | | | | |  |  |  |  |  |  |
|  | Respondent: No, we don’t feed back genetic information to participants | | | | | | | | | | | | | | | |  |
|  |  | | | | | | | | | | | | | | | |  |
|  | | | | | | | | | | | |  |  |  |  |  |  |
|  | Respondent: We don’t have challenges as far as genetics because we don’t feed back genetics information, we get challenges as far as being male or female, I don’t know whether it is only us, but most people who participate in studies are females, so sometimes as you said you have some incidental where you want to find out more about the participant but you can go to the partner because you were studying with the female and sometimes the female is also uncomfortable because she never told the partner about it. So, you wanted to know more based on that but you can’t penetrate that area, you don’t want to create problems; you keep in your lane. But in genetics, it is investigational, we don’t go to those small details, we use the sample to explain the findings | | | | | | | | | | | | | | | |  |
|  |  | | | | | | | | | | | | | | | |  |
|  | | | **Files\\Female\\R026 female** | | | | | | | | | | | | | |  |
|  |  |  | Yes |  |  |  |  |  |  | |  | | | | | |  |
|  | | |  |  |  |  |  |  |  | |  | | | | | | |
|  | | | | | | | | | | | |  |  |  |  |  |  |
|  | INTERVIEWER: So, you mean the information you get you do not link it to the individuals  RESPONDENT: No, not really. So, we link it more to their profiles not to specific persons | | | | | | | | | | | | | | | |  |
|  |  | | | | | | | | | | | | | | | |  |
| Reports\\Coding Summary By Code Report | | | | | | | | | | Page 67 of 109 | | | | | | | |
|  | | | | | | | | | | | | | | | | | |
|  | | | **Aggregate** |  | **Classification** |  | **Coverage** |  | **Number Of Coding References** | |  | **Reference Number** |  | **Coded By Initials** |  | **Modified On** |  |
|  | | | | | | | | | | | | | | | | | |
|  | | | | | | | | | | | |  |  |  |  |  |  |
|  | INTERVIEWER: Qn.3 So, are your studies of such a nature that you have to report back to your participants about their genetic makeup?  RESPONDENT: Yes, we have to give them some information of what we found. Why I say some information? Explaining genetics to anybody is complex, but we have to go back to the participants and give them, not individual, but as a group. So, we don’t give individual responses. We don’t tell a person that your genes are like this and this, because we don’t have answers yet but we give them an overview of what the study entails and what we found in our studies | | | | | | | | | | | | | | | |  |
|  |  | | | | | | | | | | | | | | | |  |
|  | | | | | | | | | | | |  |  |  |  |  |  |
|  | INTERVIEWER: So, it is a probability?  RESPONDENT: Exactly, so that is why I said it is a process in evolution, clinically we know that some families are more prone to disease than others, now the genes seem to show probably there is a genetic background to this | | | | | | | | | | | | | | | |  |
|  |  | | | | | | | | | | | | | | | |  |
|  | | | **Files\\Female\\R027.female** | | | | | | | | | | | | | |  |
|  |  |  | Yes |  |  |  |  |  |  | |  | | | | | |  |
|  | | |  |  |  |  |  |  |  | |  | | | | | | |
|  | | | | | | | | | | | |  |  |  |  |  |  |
|  | INTERVIEWER: Qn.3 Was any of your studies of such a nature that you had to return back your results to your participants on what you had discovered about their genetic make up?  RESPONDENT: No, we didn’t.  INTERVIEWER: You could not go back and give because you had this information that you are more susceptible or you may get more severe reactions compared to may be someone with another gene or something like that?  RESPONDENT: We could have but we didn’t. it is very scientific that some of those things don’t make sense to even scientists. | | | | | | | | | | | | | | | |  |
|  |  | | | | | | | | | | | | | | | |  |
|  | | | **Files\\Female\\R028.female** | | | | | | | | | | | | | |  |
|  |  |  | Yes |  |  |  |  |  |  | |  | | | | | |  |
|  | | |  |  |  |  |  |  |  | |  | | | | | | |
|  | | | | | | | | | | | |  |  |  |  |  |  |
|  | RESPONDENT: I always tell my participants that there are no immediate benefits but they are making a good contribution towards what causes the disease and management. So, there are no direct benefits to either individuals or families, and in fact they never even get to know their genotype like as individuals, we just presented aggregated results, because it also has no clinical benefit to them at the moment, knowing your genotype even if we’ve seen that it may be at risk, these are still very early days. We can’t know yet what that means. So in terms of giving feedback, we are not telling them what the findings are, and in any case since they come back after many months or even a year, because most of this work starts here, but a lot is finished in universities outside the country | | | | | | | | | | | | | | | |  |
|  |  | | | | | | | | | | | | | | | |  |
|  | | | | | | | | | | | |  |  |  |  |  |  |
|  | INTERVIEWER: Haven’t you had cases where individuals come back to ask for their results probably since you collected samples from them?  RESPONDENT: yeah, they call but we always remind them because it is in their consent that we shall not give them their genetics because it is not for immediate benefit, so, I think there is one patient one time who called and asked about it, but when we explained to her, she had forgotten what she had signed, it was several years down the road | | | | | | | | | | | | | | | |  |
|  |  | | | | | | | | | | | | | | | |  |
|  | | | | | | | | | | | |  |  |  |  |  |  |
|  | INTERVIEWER: Qn.3 So, are your studies of such a nature that you have to report back to your participants about their genetic makeup?  RESPONDENT: Then as individuals? No | | | | | | | | | | | | | | | |  |
|  |  | | | | | | | | | | | | | | | |  |
|  | | | | | | | | | | | |  |  |  |  |  |  |
|  | INTERVIEWER: So, for the next questions that means you have never returned results?  RESPONDENT: No, we haven’t | | | | | | | | | | | | | | | |  |
|  |  | | | | | | | | | | | | | | | |  |
|  | | | | | | | | | | | | | | | | | |
| Reports\\Coding Summary By Code Report | | | | | | | | | | Page 68 of 109 | | | | | | | |
|  | | | | | | | | | | | | | | | | | |
|  | | | **Aggregate** |  | **Classification** |  | **Coverage** |  | **Number Of Coding References** | |  | **Reference Number** |  | **Coded By Initials** |  | **Modified On** |  |
|  | | | **Files\\Female\\R029 female** | | | | | | | | | | | | | |  |
|  |  |  | Yes |  |  |  |  |  |  | |  | | | | | |  |
|  | | |  |  |  |  |  |  |  | |  | | | | | | |
|  | | | | | | | | | | | |  |  |  |  |  |  |
|  | Qn.3 INTERVIEWER: thank you. Then are your studies of such a nature that you have to report results back to the participants what you have discovered about their genetic makeup?  RESPONDENT: not necessarily reporting back to individuals. No  INTERVIEWER: No  RESPONDENT: yes. But of course, we have to publish these results and maybe we go back to the hospital and tell them about our findings generally.  INTERVIEWER: But not to individuals.  RESPONDENT: Not to individual participants. | | | | | | | | | | | | | | | |  |
|  |  |  |  |  |  |  |  |  |  |  |  |  |  |  |  |  |  |
|  |  | | | | | | | | | | | | | | | |  |
|  | | | **Files\\Female\\R030.female** | | | | | | | | | | | | | |  |
|  |  |  | Yes |  |  |  |  |  |  | |  | | | | | |  |
|  | | |  |  |  |  |  |  |  | |  | | | | | | |
|  | | | | | | | | | | | |  |  |  |  |  |  |
|  | RESPONDENT: Yes, if they do, we have them in aggregate through our colleague and their medicine team because we already have information of what is it in the gene, we are due to get another presentation but it has been found that the genes of these people are like those of people who have high cholesterol because cholesterol is a gene since you and mean do not look big but we may have cholesterol so they are studying that further to understand why? Why is this relationship between the cholesterol gene and the long term progressors? | | | | | | | | | | | | | | | |  |
|  |  | | | | | | | | | | | | | | | |  |
|  | | | | | | | | | | | |  |  |  |  |  |  |
|  | RESPONDENT: So far, we have not but the principle is, if anybody wants to know it is their right and I think it is important. | | | | | | | | | | | | | | | |  |
|  |  | | | | | | | | | | | | | | | |  |
|  | | | | | | | | | | | |  |  |  |  |  |  |
|  | INTERVIEWER: You said it is really necessary to return results incase participants want to know but you have never returned results?  RESPONDENT: No, so far. | | | | | | | | | | | | | | | |  |
|  |  | | | | | | | | | | | | | | | |  |
|  | | | **Files\\Male\\R-002. male** | | | | | | | | | | | | | |  |
|  |  |  | Yes |  |  |  |  |  |  | |  | | | | | |  |
|  | | |  |  |  |  |  |  |  | |  | | | | | | |
|  | | | | | | | | | | | |  |  |  |  |  |  |
|  | Respondent: no, we have not done that  Interviewer: so, you did not get back to them with results?  Respondent: yes, because we didn’t necessarily study their genetics as such, we looked at the parasites and whenever we would find positives of course we had to go back to the doctor and eventually they would get treatment based on what we found because we had several levels of diagnosis as well | | | | | | | | | | | | | | | |  |
|  |  | | | | | | | | | | | | | | | |  |
|  | | | | | | | | | | | |  |  |  |  |  |  |
|  | Respondent: I have returned results but not genetic results | | | | | | | | | | | | | | | |  |
|  |  | | | | | | | | | | | | | | | |  |
|  | | | | | | | | | | | | | | | | | |
| Reports\\Coding Summary By Code Report | | | | | | | | | | Page 69 of 109 | | | | | | | |
|  | | | | | | | | | | | | | | | | | |
|  | | | **Aggregate** |  | **Classification** |  | **Coverage** |  | **Number Of Coding References** | |  | **Reference Number** |  | **Coded By Initials** |  | **Modified On** |  |
|  | | | **Files\\Male\\R-004.male** | | | | | | | | | | | | | |  |
|  |  |  | Yes |  |  |  |  |  |  | |  | | | | | |  |
|  | | |  |  |  |  |  |  |  | |  | | | | | | |
|  | | | | | | | | | | | |  |  |  |  |  |  |
|  | Respondent: yes there are a number of times we have had to inform them of the blood group expression, some we have no idea about the blood group they express or some we only stop on the usual ABO system and antigen D of their end system know that am B positive stop there but we do extend to grouping for a number of antigens about twenty the antigens that are commonest so they have got that information that they have to express, at other times when we have done molecular studies we discovered some rare genetic expressions so like some Sicklers in Mulago we got back and informed them of the anti-usual genetic makeup that possesses complications and there is also a time when students are at this university at MUST gave me blood samples to use as regent cells I also informed them about what antigens they have | | | | | | | | | | | | | | | |  |
|  |  | | | | | | | | | | | | | | | |  |
|  | | | **Files\\Male\\R-005.male** | | | | | | | | | | | | | |  |
|  |  |  | Yes |  |  |  |  |  |  | |  | | | | | |  |
|  | | |  |  |  |  |  |  |  | |  | | | | | | |
|  | | | | | | | | | | | |  |  |  |  |  |  |
|  | Respondent: For us we were not interested in genomics, we were interested in; this is the molecule, a person like you of this nature, probably | | | | | | | | | | | | | | | |  |
|  |  | | | | | | | | | | | | | | | |  |
|  | | | | | | | | | | | |  |  |  |  |  |  |
|  | Interviewer: Which is genetics, because it is specific to….  Respondent: Oh so we return those kind of messages, but the work was published  Interviewer: Yeah there is publishing and there is going to the individual participants and telling them the findings. Did you do that?  Respondent: We had to do that, because am attached to the group whose funding is tagged to that kind of information, this is individual funders and these funders are a collection of patients, and these patients are people who are somehow affected, may be you call it a foundation or what, so these people are interested in these results in order to encourage the people who are putting in donations and feel like this kind of work is going to benefit the people suffering from that disease | | | | | | | | | | | | | | | |  |
|  |  |  |  |  |  |  |  |  |  |  |  |  |  |  |  |  |  |
|  |  | | | | | | | | | | | | | | | |  |
|  | | | **Files\\Male\\R-006. male** | | | | | | | | | | | | | |  |
|  |  |  | Yes |  |  |  |  |  |  | |  | | | | | |  |
|  | | |  |  |  |  |  |  |  | |  | | | | | | |
|  | | | | | | | | | | | |  |  |  |  |  |  |
|  | So for most of these studies we haven’t had to go back to them to say that you have these and these problems, or you have this and this genetic makeup and this is why you experiencing such and such. No we haven't had to do that. And in part, its because there are no specific guidelines defined to send back this specific information. if you think about information about an infection, somebody ahs malaria or somebody has TB , its very clear this person you will be able to tell them that you have malaria or TB , and this is the treatment. But then for this genetic information, its still not clear, if you gave this genetic information to this person, what do you want them to do with it. what do you want them to do with this information. so its still not clear, because the other thing still you have to remember is that with Genetics, when you find a genetic makeup that could be responsible for what you are observing phenopuetically doesn't necessarily translate to that per say. so explaining genetic information to patients or study participants is still not very straight forward. | | | | | | | | | | | | | | | |  |
|  |  |  |  |  |  |  |  |  |  |  |  |  |  |  |  |  |  |
|  |  | | | | | | | | | | | | | | | |  |
|  | | | | | | | | | | | |  |  |  |  |  |  |
|  | No, I have never returned any genetic results to any research participant. The data we have collected has been strictly for research purposes. | | | | | | | | | | | | | | | |  |
|  |  | | | | | | | | | | | | | | | |  |
|  | | | **Files\\Male\\R-007 male** | | | | | | | | | | | | | |  |
|  |  |  | Yes |  |  |  |  |  |  | |  | | | | | |  |
|  | | |  |  |  |  |  |  |  | |  | | | | | | |
|  | | | | | | | | | | | |  |  |  |  |  |  |
|  | Respondent: Aha, we haven’t had, we haven’t done that yet as I said we haven’t really, we’ve done mostly the, let’s say cryptal hookup, or we are trying to do some things with TB, the effecting organism. The next step is to look at the host. We haven’t really done any host genetic analysis as yet. | | | | | | | | | | | | | | | |  |
|  |  | | | | | | | | | | | | | | | |  |
| Reports\\Coding Summary By Code Report | | | | | | | | | | Page 70 of 109 | | | | | | | |
|  | | | | | | | | | | | | | | | | | |
|  | | | **Aggregate** |  | **Classification** |  | **Coverage** |  | **Number Of Coding References** | |  | **Reference Number** |  | **Coded By Initials** |  | **Modified On** |  |
|  | | | | | | | | | | | | | | | | | |
|  | | | | | | | | | | | |  |  |  |  |  |  |
|  | Respondent: No it’s not necessary to me. | | | | | | | | | | | | | | | |  |
|  |  | | | | | | | | | | | | | | | |  |
|  | | | **Files\\Male\\R-008.male** | | | | | | | | | | | | | |  |
|  |  |  | Yes |  |  |  |  |  |  | |  | | | | | |  |
|  | | |  |  |  |  |  |  |  | |  | | | | | | |
|  | | | | | | | | | | | |  |  |  |  |  |  |
|  | RESPONDENT: the incidental findings are always there but I think we don’t give them back, there are always there are genetics study has the same findings as others but never get them back | | | | | | | | | | | | | | | |  |
|  |  | | | | | | | | | | | | | | | |  |
|  | | | **Files\\Male\\R-009.male** | | | | | | | | | | | | | |  |
|  |  |  | Yes |  |  |  |  |  |  | |  | | | | | |  |
|  | | |  |  |  |  |  |  |  | |  | | | | | | |
|  | | | | | | | | | | | |  |  |  |  |  |  |
|  | INTERVIEWER: was any of your studies of such a nature that you had to report back to your participants about what you had discovered?  RESPONDENT: not yet we have not yet gotten to those discoveries we hope but we have not | | | | | | | | | | | | | | | |  |
|  |  | | | | | | | | | | | | | | | |  |
|  | | | | | | | | | | | |  |  |  |  |  |  |
|  | RESPONDENT: no | | | | | | | | | | | | | | | |  |
|  |  | | | | | | | | | | | | | | | |  |
|  | | | **Files\\Male\\R-010. male** | | | | | | | | | | | | | |  |
|  |  |  | Yes |  |  |  |  |  |  | |  | | | | | |  |
|  | | |  |  |  |  |  |  |  | |  | | | | | | |
|  | | | | | | | | | | | |  |  |  |  |  |  |
|  | INTERVIEWER: have you ever returned aggregate results?  RESPONDENT: Like all results?  INTERVIEWER: Like you go to a community, you get some findings from your study but as you are returning you don’t go to individuals…  RESPONDENT: ideally that is very important. I have not done before because a lot of the studies I am involved in are not larger scale community studies except the CafGEN study actually it’s a very big study but we do a lot of engagement of community usually before we start the study we engage communities and even at the end of the study we engage communities because it is a requirement. Many times a lot of funders are very advanced, they demand that there are mechanisms for dissemination in which we engage communities as well. But apparently NO, we have not engaged in anything like that. Courts of law at times demand this kind of evidence even if there is no study eg; issues to do with paternity. | | | | | | | | | | | | | | | |  |
|  |  |  |  |  |  |  |  |  |  |  |  |  |  |  |  |  |  |
|  |  | | | | | | | | | | | | | | | |  |
|  | | | **Files\\Male\\R-011.male** | | | | | | | | | | | | | |  |
|  |  |  | Yes |  |  |  |  |  |  | |  | | | | | |  |
|  | | |  |  |  |  |  |  |  | |  | | | | | | |
|  | | | | | | | | | | | |  |  |  |  |  |  |
|  | Have you reached that stage already?  RESPONDENT:  yes we have, but we have not yet published our work so we can't really share our results. | | | | | | | | | | | | | | | |  |
|  |  | | | | | | | | | | | | | | | |  |
|  | | | | | | | | | | | | | | | | | |
| Reports\\Coding Summary By Code Report | | | | | | | | | | Page 71 of 109 | | | | | | | |
|  | | | | | | | | | | | | | | | | | |
|  | | | **Aggregate** |  | **Classification** |  | **Coverage** |  | **Number Of Coding References** | |  | **Reference Number** |  | **Coded By Initials** |  | **Modified On** |  |
|  | | | | | | | | | | | | | | | | | |
|  | | | | | | | | | | | |  |  |  |  |  |  |
|  | No, but us we don't report directly to individuals because in all genetic studies ,we have to de-identify the data, we cannot trace back the individuals, its one of the ethical aspect of this, we work with de-identified data. We can’t report directly to individuals but we can report to community. Yes maybe aggregated results but nothing personal. | | | | | | | | | | | | | | | |  |
|  |  | | | | | | | | | | | | | | | |  |
|  | | | **Files\\Male\\R-013.male** | | | | | | | | | | | | | |  |
|  |  |  | Yes |  |  |  |  |  |  | |  | | | | | |  |
|  | | |  |  |  |  |  |  |  | |  | | | | | | |
|  | | | | | | | | | | | |  |  |  |  |  |  |
|  | RESPONDENT: I don’t think so it is not part of the study because the things that we want to detect for are not validated for clear use so the things that we are trying to study are not that they are ready for clinical use so you don’t really have an obligation to report back about the clinical findings of the patient however the case of the Simplex virus that I have told you we can recommend to the clinicians to reevaluate their patients because at least the molecular tests that can detect resistance to chemotherapy however there is another way of detecting that clinically so we would recommend the clinician to tell that this patient experiences Simplex virus so we think that they might be resistant to the drugs so we recommend that you must teamly detect for clinical signs of resistance to the drug in these patients | | | | | | | | | | | | | | | |  |
|  |  | | | | | | | | | | | | | | | |  |
|  | | | | | | | | | | | |  |  |  |  |  |  |
|  | INTERVIEWER: have you ever returned such incidental findings?  RESPONDENT: yeah, there is a time I participated in clinical trial to do research things but still there was a system, where we had to follow up these patients we knew where the patients were, it was an HIV clinical trials in eastern part of the country we used to do that because we even had a GPS tracker of knowing each home of the patient and we had people who could go and track these patients deeply in the villages so its something possible but however it is very logistic and intensive to really do  INTERVIEWER: did you have like counsellors, how did you manage?  RESPONDENT: yes, we had counsellors, trackers, some it was a big team community linkage coordinators and many more | | | | | | | | | | | | | | | |  |
|  |  |  |  |  |  |  |  |  |  |  |  |  |  |  |  |  |  |
|  |  | | | | | | | | | | | | | | | |  |
|  | | | **Files\\Male\\R-014 male** | | | | | | | | | | | | | |  |
|  |  |  | Yes |  |  |  |  |  |  | |  | | | | | |  |
|  | | |  |  |  |  |  |  |  | |  | | | | | | |
|  | | | | | | | | | | | |  |  |  |  |  |  |
|  | RESPONDENT: feedback is given in terms of publications, you don’t expect a village guy there to read a publication but you are informing someone in the research world or someone who is already sensitized. And some of the genetic data is deposited into the MCDI data set. Its not for sell because you didn’t buy the samples, so you are just adding a brick for someone to have a starting point in future but we have this much of Ugandan data so if I wake up and I want to use, I can use it without going back to use invasive methods to collect samples from patients. Even time, that’s why we have this bio repository because you have very many samples here for any researcher and you don’t need to look at, perhaps the patients are even dead sometimes but the samples are there. | | | | | | | | | | | | | | | |  |
|  |  | | | | | | | | | | | | | | | |  |
|  | | | | | | | | | | | |  |  |  |  |  |  |
|  | INTERVIEWER: so, when you get your results you report them to the clinician?  RESPONDENT: yes and I guide them. | | | | | | | | | | | | | | | |  |
|  |  | | | | | | | | | | | | | | | |  |
|  | | | **Files\\Male\\R-015.male** | | | | | | | | | | | | | |  |
|  |  |  | Yes |  |  |  |  |  |  | |  | | | | | |  |
|  | | |  |  |  |  |  |  |  | |  | | | | | | |
|  | | | | | | | | | | | |  |  |  |  |  |  |
|  | RESPONDENT: return of results is in two ways; we have incidental findings and expected findings, usually expected findings you may not return them because you are expected to find them | | | | | | | | | | | | | | | |  |
|  |  | | | | | | | | | | | | | | | |  |
|  | | | | | | | | | | | | | | | | | |
| Reports\\Coding Summary By Code Report | | | | | | | | | | Page 72 of 109 | | | | | | | |
|  | | | | | | | | | | | | | | | | | |
|  | | | **Aggregate** |  | **Classification** |  | **Coverage** |  | **Number Of Coding References** | |  | **Reference Number** |  | **Coded By Initials** |  | **Modified On** |  |
|  | | | **Files\\Male\\R-016. male** | | | | | | | | | | | | | |  |
|  |  |  | Yes |  |  |  |  |  |  | |  | | | | | |  |
|  | | |  |  |  |  |  |  |  | |  | | | | | | |
|  | | | | | | | | | | | |  |  |  |  |  |  |
|  | RESPONDENT: no, we don’t report back results to the participants | | | | | | | | | | | | | | | |  |
|  |  | | | | | | | | | | | | | | | |  |
|  | | | | | | | | | | | |  |  |  |  |  |  |
|  | RESPONDENT: so, at the end of the study we will, you see the study is such hat we have genetic samples DNA samples from about five countries and then we have each country having like five sites so you have 40,000 DNA samples for you to make sense out of DNA samples genetic research you need 10,000s of results so reporting or knowing your results alone is not something that helps you directly because it is of no benefit you cannot use those results to do anything, the only way we can use those results is if we have that information from those 40,000 participants collected together I hope that helps | | | | | | | | | | | | | | | |  |
|  |  | | | | | | | | | | | | | | | |  |
|  | | | | | | | | | | | |  |  |  |  |  |  |
|  | RESPONDENT: yes, it may take like two to maybe come up with results so they have collected data for maybe about from like 2000 actually like from the whole of Africa we have like maybe over 1000 samples and the only results we have is 192 out of 10,000 so it takes a very long time to actually analyses this kind of data and make sense out of it | | | | | | | | | | | | | | | |  |
|  |  | | | | | | | | | | | | | | | |  |
|  | | | **Files\\Male\\R-017. male** | | | | | | | | | | | | | |  |
|  |  |  | Yes |  |  |  |  |  |  | |  | | | | | |  |
|  | | |  |  |  |  |  |  |  | |  | | | | | | |
|  | | | | | | | | | | | |  |  |  |  |  |  |
|  | Respondent: Yeah, there are different ways of reporting results, one through seminars, reporting to the community, publications-reporting to the international community about your findings and actually going back to the individuals or the communities where you obtained the samples from, reporting back to them | | | | | | | | | | | | | | | |  |
|  |  | | | | | | | | | | | | | | | |  |
|  | | | | | | | | | | | |  |  |  |  |  |  |
|  | Respondent: We don’t report the results directly, like I said there are different media of reporting back, publishing in international journals, seminars, but also to the community. You can report back to the community that we have these findings, however this being an health based research you find that there are always statistics that are obtained from the hospital about those particular diseases and our information plays part in those statistics, yes, like you go to a population and find that this number of individuals have this particular disease, it’s a report that is also used by the health demographic surveys, so those are different media through which we report our different results | | | | | | | | | | | | | | | |  |
|  |  | | | | | | | | | | | | | | | |  |
|  | | | | | | | | | | | |  |  |  |  |  |  |
|  | Respondent: Ahhh, me personally as a researcher I don’t have access to the individual, there is a protocol, because even if you are to get the sample, me the researcher, I don’t directly, there are procedures we have to follow, there are protocols we follow, ethical considerations passes by the ethics committee, and even dissemination of that information is through specific individuals | | | | | | | | | | | | | | | |  |
|  |  | | | | | | | | | | | | | | | |  |
|  | | | | | | | | | | | |  |  |  |  |  |  |
|  | Respondent: Yes, the researcher is the person who gets the individual sample sequences/sends it for sequencing, then me, as a bioinformatician, I analyze that data and disseminate it in a way that is understandable. So that information once it is obtained then it is also relayed to other individuals within the project who will go back to the community, like the team leader who is involved in the community engagement, he’s the one who sensitises the community and gives feed back to the community, but me as a researcher I don’t interface with the community | | | | | | | | | | | | | | | |  |
|  |  | | | | | | | | | | | | | | | |  |
|  | | | **Files\\Male\\R-019 male** | | | | | | | | | | | | | |  |
|  |  |  | Yes |  |  |  |  |  |  | |  | | | | | |  |
|  | | |  |  |  |  |  |  |  | |  | | | | | | |
|  | | | | | | | | | | | |  |  |  |  |  |  |
|  | Respondent: It completely depends on the findings, but we are obliged to feedback findings, and it’s in our strategy, of part of community engagement to feed back findings to the community in general, or specific individuals and those approaches depend on what the results are so we have to be sensitive to the culture  Interviewer: So, it’s not like a structured way already designed, it will depend on the outcome?  Respondent: Yes | | | | | | | | | | | | | | | |  |
|  |  | | | | | | | | | | | | | | | |  |
| Reports\\Coding Summary By Code Report | | | | | | | | | | Page 73 of 109 | | | | | | | |
|  | | | | | | | | | | | | | | | | | |
|  | | | **Aggregate** |  | **Classification** |  | **Coverage** |  | **Number Of Coding References** | |  | **Reference Number** |  | **Coded By Initials** |  | **Modified On** |  |
|  | | | | | | | | | | | | | | | | | |
|  | | | | | | | | | | | |  |  |  |  |  |  |
|  | Respondent: There are approaches we have that are general to the community, there are things we feedback, one the progress of the research, what are we doing, how far have we gone? General information. Next, we can feed back specific findings, do you have the genetic markers for susceptibility to disease? we have not yet identified them so we cannot yet feed this back. And then the other feed back would be incidental findings but in our kind of studies the incidental findings are very rare. Because for example, when we invite the participants for this study we don’t do things like hypertension, blood sugar, those are the kind of things which would bring you incidental findings, of course there are things which we don’t do, like if we do family genomics, we do not feedback about paternity. | | | | | | | | | | | | | | | |  |
|  |  | | | | | | | | | | | | | | | |  |
|  | | | | | | | | | | | |  |  |  |  |  |  |
|  | Respondent: Yes, but it’s not in our interest to feed that back, especially if there is a mismatch, it’s not in our interest, not even in the interest of the community, to them you are just causing problems in the community. The social issues there, there are some social issues which none of us can solve, otherwise we are causing more problems, and those ones we avoid, that’s the strategy | | | | | | | | | | | | | | | |  |
|  |  | | | | | | | | | | | | | | | |  |
|  | | | **Files\\Male\\R-022. male** | | | | | | | | | | | | | |  |
|  |  |  | Yes |  |  |  |  |  |  | |  | | | | | |  |
|  | | |  |  |  |  |  |  |  | |  | | | | | | |
|  | | | | | | | | | | | |  |  |  |  |  |  |
|  | RESPONDENT: we only report what is beneficial or useful to the patient and this information is reported to the respective doctor. So, we tell the doctors; this patient of yours has this type of virus so they will not be responding to this kind of treatment. We advise that you probably prescribe this other type of treatment. So, we only report what is medically beneficial to the patient.  INTERVIEWER: and psychologically beneficial?  RESPONDENT: they are both; they are all beneficial | | | | | | | | | | | | | | | |  |
|  |  | | | | | | | | | | | | | | | |  |
|  | | | | | | | | | | | |  |  |  |  |  |  |
|  | RESPONDENT: yes, we don’t report to patients because most likely they may not have the knowledge to understand what you are reporting or they may not have interpretations of this so you have to give reports to someone who can interpret what we are giving them | | | | | | | | | | | | | | | |  |
|  |  | | | | | | | | | | | | | | | |  |
|  | | | **Files\\Male\\R-023. male** | | | | | | | | | | | | | |  |
|  |  |  | Yes |  |  |  |  |  |  | |  | | | | | |  |
|  | | |  |  |  |  |  |  |  | |  | | | | | | |
|  | | | | | | | | | | | |  |  |  |  |  |  |
|  | Respondent: No, the one I was involved in was not necessary because I was looking for things at a population level, so it was not so much on the individuals but more on a population level | | | | | | | | | | | | | | | |  |
|  |  | | | | | | | | | | | | | | | |  |
|  | | | | | | | | | | | | | | | | | |
|  | | | | | | | | | | | | | | | | | |
|  | | | | | | | | | | | | | | | | | |
| Reports\\Coding Summary By Code Report | | | | | | | | | | Page 74 of 109 | | | | | | | |
|  | | | | | | | | | | | | | | | | | |
|  | | | **Aggregate** |  | **Classification** |  | **Coverage** |  | **Number Of Coding References** | |  | **Reference Number** |  | **Coded By Initials** |  | **Modified On** |  |
|  | | | | | | | | | | | | | | | | | |
|  | | | | | | | | | | | |  |  |  |  |  |  |
|  | Interviewer: And are there repercussions if you don’t?  Respondent: They don’t. I think for my particular kind of work, I think there are no repercussions because even if you did not, the people who would use that most are those who implement disease control, so people like AIDs control Programme, because we are looking at population level dynamics so for them it would be more to answer a question like what regimens should we may be adopt in our treatment policy for the country. so for them as long as they know, that’s almost good enough because they are the ones who initiate and provide the regimens and the rest of the people follow. But I would imagine a case like where it would be good for individuals to also know, so that they can do something about it. In my case the individuals, even if they knew, they were not gonna change a regimen because we already have what’s on the market, but if it were a study that was more targeted to the individual themselves and you find probably an individual with an actual predisposition that makes them susceptible and something could be done about it, then it would be unethical not to tell them that you know we found this making you predisposed to being susceptible to HIV or this drug not working in your case, and we think that you should be taking this but it is better you take this other thing, but my work was not at that level but am sure there are instances of the research which could apply and you would be not being very ethical if you didn’t do that. But on the other hand the challenge also is that also genetics is not an exact, coz you are saying this thing is predisposing you, so we are just saying maybe having this mutation make you at more at risk of getting this, but the way things end up working is not a direct, sometimes its not even that you got it, what we are saying will happen will happen, coz there are other factors that come in to play outside the genetics, genetics is one bit, and then we have all these other things playing a role. So, again on the other hand if I told you that I analyzed your sample from my work and I saw that you have maybe this mutation that may predispose you to maybe say running mad at a later stage, but I also know that it is not a one on one, because there other things that play. So, either two things happen, either you take it and have this worry that that is going to happen, yet in reality there is still a probability that it may not. So I don’t know where one draws the line, even in those cases, my work was not on that but am just opening up on a larger, that even if it is good to tell, because genetics is not necessarily a given that it will happen, even if you have the mutant gene, because the outcome is a factor of yes the gene plays a role, but there also other factors that would have come in | | | | | | | | | | | | | | | |  |
|  |  |  |  |  |  |  |  |  |  |  |  |  |  |  |  |  |  |
|  |  |  |  |  |  |  |  |  |  |  |  |  |  |  |  |  |  |
|  |  |  |  |  |  |  |  |  |  |  |  |  |  |  |  |  |  |
|  |  | | | | | | | | | | | | | | | |  |
|  | | | **Files\\Male\\R-024.male** | | | | | | | | | | | | | |  |
|  |  |  | Yes |  |  |  |  |  |  | |  | | | | | |  |
|  | | |  |  |  |  |  |  |  | |  | | | | | | |
|  | | | | | | | | | | | |  |  |  |  |  |  |
|  | RESPONDENT: We do report back for all our genetic studies, this is particularly very easy for us when we are doing prospective studies like those I have mentioned, however there are studies that we doing on samples that we collected in 2011 we cannot trace back those patients, these are samples that are stored and we have not been able to get back to the individual patients and studies that we developed where on the samples that we already collected so we developed a research idea based on the idea that we already have the samples. so those are the few studies that we have not been able to feedback, but all the rest patients consent for the genetic studies we do the analysis and then do the feedback. The beauty about what I am talking about is that we now have the real time genetic study tests and we are able to feed back to the patients as they come.  INTERVIEWER: What does that mean to the young ones physical health and social lives as individuals, families and communities?  RESPONDENT: On the whole it is very re-assuring to the parents that they have been able to know the genetic condition which is predispose to their children to illnesses so they comply on the management plan for some of these I also don’t manage I refer to my colleagues. we have been able to get in touch with these colleagues and tell us that the children have been attending the screenings. But there also instances where these tests have caused a bit of trouble and I am not ashamed of telling one particular case that you may have to reference that very prominent person in the region came with a very sick child, I handled the child and the child had sickle cell. so after consenting them we said lets first go through the screening test, the screening test was purely for management of the patient in the ward and to show the child may have a likelihood of sickle. then I told them, lets now do a confirmatory test because we are on the research test. so when the confirmatory test came back positive the father a very prominent person, said he doesn’t have sickle in his family and that is not his child therefore. So there those connotations, I should therefore say that this is one case out of over 3000 that we have managed but still it has its social impact and eventually it took long to convince that very prominent person and his wife to test together and when they tested carriers we were now good to explain how the child got sickle cell, but you can imagine the child had to go through stress first before eventually that is done. | | | | | | | | | | | | | | | |  |
|  |  |  |  |  |  |  |  |  |  |  |  |  |  |  |  |  |  |
|  |  |  |  |  |  |  |  |  |  |  |  |  |  |  |  |  |  |
|  |  | | | | | | | | | | | | | | | |  |
|  | | | | | | | | | | | |  |  |  |  |  |  |
|  | Respondent: Oh yeah, that one and you can imagine that the parents have to manage this child in a very special way but we’ve also had a child who developed recurrent dark urine, what is commonly called hemoglobinuria, so we eventually managed to tell the parents that look it is because the child has glucose-6-phosphate dehydrogenes, a genetic condition that has predisposed the child to this, and they were able to accept referral to Mulago and a colleague of mine managed that child in Mulago. Now the child is stable and they already know what they should do when the child develops fever or tests for malaria. So those are very out standing because they were on extreme. The other one managed to join medical school and was at medical school up to third year, then this one had over 70 blood transfusions by the time I saw the child. Now knowing this and cutting down on transfusions almost to nothing is a very, very outstanding this. So in between of course you get other mild cases | | | | | | | | | | | | | | | |  |
|  |  |  |  |  |  |  |  |  |  |  |  |  |  |  |  |  |  |
|  |  | | | | | | | | | | | | | | | |  |
| Reports\\Coding Summary By Code Report | | | | | | | | | | Page 75 of 109 | | | | | | | |
|  | | | | | | | | | | | | | | | | | |
|  | | | **Aggregate** |  | **Classification** |  | **Coverage** |  | **Number Of Coding References** | |  | **Reference Number** |  | **Coded By Initials** |  | **Modified On** |  |
|  | | | | | | | | | | | | | | | | | |
|  | | | | | | | | | | | |  |  |  |  |  |  |
|  | Interviewer: Did you also return results to these communities?  Respondent: yes for instance we typed over 2000 community samples for 3 conditions, sickle, beta-thalassemia and glucose-6-phosphate dehydrogenase and those that we return, we return all the results as individual results and those that we found positive, we’ve been able to refer them to specialized centers and I think maybe up to 80% of those who were positive are being followed up by the hospital clinic | | | | | | | | | | | | | | | |  |
|  |  | | | | | | | | | | | | | | | |  |
|  | | | **Files\\Male\\R-025 male..** | | | | | | | | | | | | | |  |
|  |  |  | Yes |  |  |  |  |  |  | |  | | | | | |  |
|  | | |  |  |  |  |  |  |  | |  | | | | | | |
|  | | | | | | | | | | | |  |  |  |  |  |  |
|  | Respondent: This is really to see if certain communities have been predisposed to certain genetic defects and thus disposed to certain genetic diseases, we have not yet connected. We have just said, okay these families are very close to each other, the genomes are very similar to each other, the other one are very heterogenous and that is the mix that is there, eg we did a study form west Nile, Acholi and Lango sub region, as you go toward the Lango sub region there is a lot of mix, eg in the Lango sub region there is a lot Bantu mix as well as the Nilotic mix, so we have not yet inferred anything whether it is which disease, whether to confer certain beneficial characteristic, but it is just general information, probably we already know, but we know there is a lot of mixture here, there is a lot of pure familial line here and then we are hoping that when the analysis of genetic is done, we can predict some disease itself | | | | | | | | | | | | | | | |  |
|  |  |  |  |  |  |  |  |  |  |  |  |  |  |  |  |  |  |
|  |  | | | | | | | | | | | | | | | |  |
|  | | | | | | | | | | | |  |  |  |  |  |  |
|  | Respondent: No, we haven’t. There is also another arm to that, there is a culture of Burkitt’s lymphoma, trying to see the various genetic distance with them but we haven’t. The only benefit we have given them is that we offer them, we have them with whatever treatment they will undergo. Let us say we bought those drugs and they are treated for free, they are given health education and then we connected them to a team that follows them up but we haven’t | | | | | | | | | | | | | | | |  |
|  |  | | | | | | | | | | | | | | | |  |
|  | | | | | | | | | | | |  |  |  |  |  |  |
|  | Interviewer: And what was the outcome from that reporting so far?  Respondent: Well they just showed interest that apart from that day they wanted to know, that being few or being mixed does it confer any advantage, but we don’t have an answer for this but it is just an information about themselves, maybe somebody can further it in future but we also find that there are many tribes which have already been matched but for the nilotics this might have been the first matching or let’s say the genetic mix in the society called this, so we use like ganda, we use there is a tribe Luo in this other eastern part, but the Luo blood was very few, then we also use the West African.. the results of the west African blood but the mix is not so bad, there is a little mix but the genetic mix is not so bad because those are the tribes which already have supplementary map | | | | | | | | | | | | | | | |  |
|  |  |  |  |  |  |  |  |  |  |  |  |  |  |  |  |  |  |
|  |  | | | | | | | | | | | | | | | |  |
|  | **Nodes\\Return of results\\Results returned\Depends** | | | | | | | | | | | | | | | |  |
|  | | **Document** | | | | | | | | | | | | | | |  |
|  | | | **Files\\Male\\R-019 male** | | | | | | | | | | | | | |  |
|  |  |  | No |  |  |  |  |  |  | |  | | | | | |  |
|  | | |  |  |  |  |  |  |  | |  | | | | | | |
|  | | | | | | | | | | | |  |  |  |  |  |  |
|  | Respondent: It completely depends on the findings, but we are obliged to feedback findings, and it’s in our strategy, as part of community engagement to feed back findings to the community in general, or specific individuals and those approaches depend on what the results are so we have to be sensitive to the culture  Interviewer: So, it’s not like a structured way already designed, it will depend on the outcome?  Respondent: Yes | | | | | | | | | | | | | | | |  |
|  |  | | | | | | | | | | | | | | | |  |
| Reports\\Coding Summary By Code Report | | | | | | | | | | Page 76 of 109 | | | | | | | |
|  | | | | | | | | | | | | | | | | | |
|  | | | **Aggregate** |  | **Classification** |  | **Coverage** |  | **Number Of Coding References** | |  | **Reference Number** |  | **Coded By Initials** |  | **Modified On** |  |
|  | | | **Files\\Male\\R-022. male** | | | | | | | | | | | | | |  |
|  |  |  | No |  |  |  |  |  |  | |  | | | | | |  |
|  | | |  |  |  |  |  |  |  | |  | | | | | | |
|  | | | | | | | | | | | |  |  |  |  |  |  |
|  | RESPONDENT: we only report what is beneficial or useful to the patient and this information is reported to the respective doctor. So, we tell the doctors; this patient of yours has this type of virus so they will not be responding to this kind of treatment. We advise that you probably prescribe this other type of treatment. So, we only report what is medically beneficial to the patient.  INTERVIEWER: and psychologically beneficial?  RESPONDENT: they are both; they are all beneficial | | | | | | | | | | | | | | | |  |
|  |  | | | | | | | | | | | | | | | |  |
|  | **Nodes\\Return of results\\Results returned\No** | | | | | | | | | | | | | | | |  |
|  | | **Document** | | | | | | | | | | | | | | |  |
|  | | | **Files\\Female\\R-003.female** | | | | | | | | | | | | | |  |
|  |  |  | No |  |  |  |  |  |  | |  | | | | | |  |
|  | | |  |  |  |  |  |  |  | |  | | | | | | |
|  | | | | | | | | | | | |  |  |  |  |  |  |
|  | RESPONDENT: no, the information which we, well let me say what we did then you will figure out whether what I am saying is addressing your question, what we did after we completed the study the good thing is that I would go to the field quite often so I would update even my research assistant and we were using Kagando hospital as our base so throughout the study there were kind of following really whenever we would analyse then we would tell them that these are some of the things that we have found out and then finally at the end after the study was completed, I provided a report followed with the permanent script which I took to kagando hospital but not generally to general community we did not have anything like a community meeting for dissemination it was not there | | | | | | | | | | | | | | | |  |
|  |  | | | | | | | | | | | | | | | |  |
|  | | | | | | | | | | | |  |  |  |  |  |  |
|  | INTERVIEWER: but for the individuals who had sickle cells they were given information back  RESPONDENT: back to their home? We did not go back to the homes | | | | | | | | | | | | | | | |  |
|  |  | | | | | | | | | | | | | | | |  |
|  | | | | | | | | | | | |  |  |  |  |  |  |
|  | INTERVIEWER: back to the participants, it could be to participants to a hospital or a community  RESPONDENT: not to specific individuals like I have already said that these results were really summarized in the sense that we were just saying that ok comparing this region in Mbarara will have these so many children who are having this trait | | | | | | | | | | | | | | | |  |
|  |  | | | | | | | | | | | | | | | |  |
|  | | | **Files\\Female\\R-021 female** | | | | | | | | | | | | | |  |
|  |  |  | No |  |  |  |  |  |  | |  | | | | | |  |
|  | | |  |  |  |  |  |  |  | |  | | | | | | |
|  | | | | | | | | | | | |  |  |  |  |  |  |
|  | Respondent: No, we don’t feed back genetic information to participants | | | | | | | | | | | | | | | |  |
|  |  | | | | | | | | | | | | | | | |  |
|  | | | | | | | | | | | |  |  |  |  |  |  |
|  | Respondent: We don’t have challenges as far as genetics because we don’t feed back genetics information, we get challenges as far as being male or female, I don’t know whether it is only us, but most people who participate in studies are females, so sometimes as you said you have some incidental where you want to find out more about the participant but you can go to the partner because you were studying with the female and sometimes the female is also uncomfortable because she never told the partner about it. So, you wanted to know more based on that but you can’t penetrate that area, you don’t want to create problems; you keep in your lane. But in genetics, it is investigational, we don’t go to those small details, we use the sample to explain the findings | | | | | | | | | | | | | | | |  |
|  |  | | | | | | | | | | | | | | | |  |
| Reports\\Coding Summary By Code Report | | | | | | | | | | Page 77 of 109 | | | | | | | |
|  | | | | | | | | | | | | | | | | | |
|  | | | **Aggregate** |  | **Classification** |  | **Coverage** |  | **Number Of Coding References** | |  | **Reference Number** |  | **Coded By Initials** |  | **Modified On** |  |
|  | | | **Files\\Female\\R027.female** | | | | | | | | | | | | | |  |
|  |  |  | No |  |  |  |  |  |  | |  | | | | | |  |
|  | | |  |  |  |  |  |  |  | |  | | | | | | |
|  | | | | | | | | | | | |  |  |  |  |  |  |
|  | INTERVIEWER: Qn.3 Was any of your studies of such a nature that you had to return back your results to your participants on what you had discovered about their genetic make up?  RESPONDENT: No, we didn’t.  INTERVIEWER: You could not go back and give because you had this information that you are more susceptible or you may get more severe reactions compared to may be someone with another gene or something like that?  RESPONDENT: We could have but we didn’t. it is very scientific that some of those things don’t make sense to even scientists. | | | | | | | | | | | | | | | |  |
|  |  | | | | | | | | | | | | | | | |  |
|  | | | **Files\\Female\\R028.female** | | | | | | | | | | | | | |  |
|  |  |  | No |  |  |  |  |  |  | |  | | | | | |  |
|  | | |  |  |  |  |  |  |  | |  | | | | | | |
|  | | | | | | | | | | | |  |  |  |  |  |  |
|  | RESPONDENT: I always tell my participants that there are no immediate benefits but they are making a good contribution towards what causes the disease and management. So, there are no direct benefits to either individuals or families, and in fact they never even get to know their genotype like as individuals, we just presented aggregated results, because it also has no clinical benefit to them at the moment, knowing your genotype even if we’ve seen that it may be at risk, these are still very early days. We can’t know yet what that means. So in terms of giving feedback, we are not telling them what the findings are, and in any case since they come back after many months or even a year, because most of this work starts here, but a lot is finished in universities outside the country | | | | | | | | | | | | | | | |  |
|  |  | | | | | | | | | | | | | | | |  |
|  | | | | | | | | | | | |  |  |  |  |  |  |
|  | INTERVIEWER: Qn.3 So, are your studies of such a nature that you have to report back to your participants about their genetic makeup?  RESPONDENT: Then as individuals? No | | | | | | | | | | | | | | | |  |
|  |  | | | | | | | | | | | | | | | |  |
|  | | | | | | | | | | | |  |  |  |  |  |  |
|  | INTERVIEWER: So, for the next questions that means you have never returned results?  RESPONDENT: No, we haven’t | | | | | | | | | | | | | | | |  |
|  |  | | | | | | | | | | | | | | | |  |
|  | | | **Files\\Female\\R029 female** | | | | | | | | | | | | | |  |
|  |  |  | No |  |  |  |  |  |  | |  | | | | | |  |
|  | | |  |  |  |  |  |  |  | |  | | | | | | |
|  | | | | | | | | | | | |  |  |  |  |  |  |
|  | Qn.3 INTERVIEWER: thank you. Then are your studies of such a nature that you have to report results back to the participants what you have discovered about their genetic makeup?  RESPONDENT: not necessarily reporting back to individuals. No  INTERVIEWER: No  RESPONDENT: yes. But of course, we have to publish these results and maybe we go back to the hospital and tell me about our findings generally.  INTERVIEWER: But not to individuals.  RESPONDENT: Not to individual participants. | | | | | | | | | | | | | | | |  |
|  |  |  |  |  |  |  |  |  |  |  |  |  |  |  |  |  |  |
|  |  | | | | | | | | | | | | | | | |  |
|  | | | **Files\\Female\\R030.female** | | | | | | | | | | | | | |  |
|  |  |  | No |  |  |  |  |  |  | |  | | | | | |  |
|  | | |  |  |  |  |  |  |  | |  | | | | | | |
|  | | | | | | | | | | | |  |  |  |  |  |  |
|  | RESPONDENT: So far, we have not but the principle is, if anybody wants to know it is their right and I think it is important. | | | | | | | | | | | | | | | |  |
|  |  | | | | | | | | | | | | | | | |  |
| Reports\\Coding Summary By Code Report | | | | | | | | | | Page 78 of 109 | | | | | | | |
|  | | | | | | | | | | | | | | | | | |
|  | | | **Aggregate** |  | **Classification** |  | **Coverage** |  | **Number Of Coding References** | |  | **Reference Number** |  | **Coded By Initials** |  | **Modified On** |  |
|  | | | | | | | | | | | | | | | | | |
|  | | | | | | | | | | | |  |  |  |  |  |  |
|  | INTERVIEWER: You said it is really necessary to return results incase participants want to know but you have never returned results?  RESPONDENT: No, so far. | | | | | | | | | | | | | | | |  |
|  |  | | | | | | | | | | | | | | | |  |
|  | | | **Files\\Male\\R-002. male** | | | | | | | | | | | | | |  |
|  |  |  | No |  |  |  |  |  |  | |  | | | | | |  |
|  | | |  |  |  |  |  |  |  | |  | | | | | | |
|  | | | | | | | | | | | |  |  |  |  |  |  |
|  | Respondent: no, we have not done that  Interviewer: so, you did not get back to them with results?  Respondent: yes, because we didn’t necessarily study their genetics as such, we looked at the parasites and whenever we would find positives of course we had to go back to the doctor and eventually they would get treatment based on what we found because we had several levels of diagnosis as well | | | | | | | | | | | | | | | |  |
|  |  | | | | | | | | | | | | | | | |  |
|  | | | | | | | | | | | |  |  |  |  |  |  |
|  | Respondent: I have returned results but not genetic results | | | | | | | | | | | | | | | |  |
|  |  | | | | | | | | | | | | | | | |  |
|  | | | **Files\\Male\\R-005.male** | | | | | | | | | | | | | |  |
|  |  |  | No |  |  |  |  |  |  | |  | | | | | |  |
|  | | |  |  |  |  |  |  |  | |  | | | | | | |
|  | | | | | | | | | | | |  |  |  |  |  |  |
|  | Respondent: For us we were not interested in genomics, we were interested in; this is the molecule, a person like you of this nature, probably | | | | | | | | | | | | | | | |  |
|  |  | | | | | | | | | | | | | | | |  |
|  | | | **Files\\Male\\R-006. male** | | | | | | | | | | | | | |  |
|  |  |  | No |  |  |  |  |  |  | |  | | | | | |  |
|  | | |  |  |  |  |  |  |  | |  | | | | | | |
|  | | | | | | | | | | | |  |  |  |  |  |  |
|  | So for most of these studies we haven’t had to go back to them to say that you have these and these problems, or you have this and this genetic makeup and this is why you experiencing such and such. No we haven't had to do that and in part, its because there no specific guidelines defined to send back this specific information. if you think about information about an infection, somebody has malaria or somebody has TB, its very clear this person you will be able to tell them that you have malaria or TB, and this is the treatment. But then for this genetic information, its still not clear- if you gave this genetic information to this person, what do you want them to do with it. what do you want them to do with this information. so its still not clear, because the other thing still you have to remember is that with Genetics, when you find a genetic makeup that could be responsible for what you are observing phenopuetically doesn't necessarily translate to that per say. so explaining genetic information to patients or study participants is still not very straight forward. | | | | | | | | | | | | | | | |  |
|  |  |  |  |  |  |  |  |  |  |  |  |  |  |  |  |  |  |
|  |  | | | | | | | | | | | | | | | |  |
|  | | | | | | | | | | | |  |  |  |  |  |  |
|  | No, I have never returned any genetic results to any research participant. The data we have collected has been strictly for research purposes. | | | | | | | | | | | | | | | |  |
|  |  | | | | | | | | | | | | | | | |  |
|  | | | **Files\\Male\\R-007 male** | | | | | | | | | | | | | |  |
|  |  |  | No |  |  |  |  |  |  | |  | | | | | |  |
|  | | |  |  |  |  |  |  |  | |  | | | | | | |
|  | | | | | | | | | | | |  |  |  |  |  |  |
|  | Respondent: Aha, we haven’t had, we haven’t done that yet as I said we haven’t really, we’ve done mostly the, let’s say cryptal hookup, or we are trying to do some things with TB, the affecting organism. The next step is to look at the host. We haven’t really done any host genetic analysis as yet. | | | | | | | | | | | | | | | |  |
|  |  | | | | | | | | | | | | | | | |  |
| Reports\\Coding Summary By Code Report | | | | | | | | | | Page 79 of 109 | | | | | | | |
|  | | | | | | | | | | | | | | | | | |
|  | | | **Aggregate** |  | **Classification** |  | **Coverage** |  | **Number Of Coding References** | |  | **Reference Number** |  | **Coded By Initials** |  | **Modified On** |  |
|  | | | | | | | | | | | | | | | | | |
|  | | | | | | | | | | | |  |  |  |  |  |  |
|  | Respondent: No it’s not necessary to me. | | | | | | | | | | | | | | | |  |
|  |  | | | | | | | | | | | | | | | |  |
|  | | | **Files\\Male\\R-008.male** | | | | | | | | | | | | | |  |
|  |  |  | No |  |  |  |  |  |  | |  | | | | | |  |
|  | | |  |  |  |  |  |  |  | |  | | | | | | |
|  | | | | | | | | | | | |  |  |  |  |  |  |
|  | RESPONDENT: the incidental findings are always there but I think we don’t give them back, there are always there are genetics study has the same findings as other but never get them back | | | | | | | | | | | | | | | |  |
|  |  | | | | | | | | | | | | | | | |  |
|  | | | **Files\\Male\\R-009.male** | | | | | | | | | | | | | |  |
|  |  |  | No |  |  |  |  |  |  | |  | | | | | |  |
|  | | |  |  |  |  |  |  |  | |  | | | | | | |
|  | | | | | | | | | | | |  |  |  |  |  |  |
|  | INTERVIEWER: was any of your studies of such a nature that you had to report back to your participants about what you had discovered?  RESPONDENT: not yet we have not yet gotten to those discoveries we hope but we have not | | | | | | | | | | | | | | | |  |
|  |  | | | | | | | | | | | | | | | |  |
|  | | | | | | | | | | | |  |  |  |  |  |  |
|  | RESPONDENT: no | | | | | | | | | | | | | | | |  |
|  |  | | | | | | | | | | | | | | | |  |
|  | | | **Files\\Male\\R-010. male** | | | | | | | | | | | | | |  |
|  |  |  | No |  |  |  |  |  |  | |  | | | | | |  |
|  | | |  |  |  |  |  |  |  | |  | | | | | | |
|  | | | | | | | | | | | |  |  |  |  |  |  |
|  | INTERVIEWER: have you ever returned aggregate results?  RESPONDENT: Like all results?  INTERVIEWER: Like you go to a community, you get some findings from your study but as you are returning you don’t go to individuals…  RESPONDENT: ideally that is very important. I have not done before because a lot of the studies I am involved in are not larger scale community studies except the CafGEN study actually it’s a very big study but we do a lot of engagement of community usually before we start the study we engage communities and even at the end of the study we engage communities because it is a requirement. Many times a lot of funders are very advanced, they demand that there are mechanisms for dissemination in which we engage communities as well. But apparently NO, we have not engaged in anything like that. Courts of law at times demand this kind of evidence even if there is no study eg; issues to do with paternity. | | | | | | | | | | | | | | | |  |
|  |  |  |  |  |  |  |  |  |  |  |  |  |  |  |  |  |  |
|  |  | | | | | | | | | | | | | | | |  |
|  | | | **Files\\Male\\R-011.male** | | | | | | | | | | | | | |  |
|  |  |  | No |  |  |  |  |  |  | |  | | | | | |  |
|  | | |  |  |  |  |  |  |  | |  | | | | | | |
|  | | | | | | | | | | | |  |  |  |  |  |  |
|  | Have you reached that stage already?  RESPONDENT:  yes we have, but we have not yet published our work so we can't really shared our results. | | | | | | | | | | | | | | | |  |
|  |  | | | | | | | | | | | | | | | |  |
|  | | | | | | | | | | | | | | | | | |
| Reports\\Coding Summary By Code Report | | | | | | | | | | Page 80 of 109 | | | | | | | |
|  | | | | | | | | | | | | | | | | | |
|  | | | **Aggregate** |  | **Classification** |  | **Coverage** |  | **Number Of Coding References** | |  | **Reference Number** |  | **Coded By Initials** |  | **Modified On** |  |
|  | | | | | | | | | | | | | | | | | |
|  | | | | | | | | | | | |  |  |  |  |  |  |
|  | No, but us we don't report directly to individuals because in all genetic studies, we have to de-identify the data, we cannot trace back the individuals, its one of the ethical aspects of this, we work with de-identified data. We can’t report directly to individuals but we can report to the community. Yes maybe aggregated results but nothing personal. | | | | | | | | | | | | | | | |  |
|  |  | | | | | | | | | | | | | | | |  |
|  | | | **Files\\Male\\R-013.male** | | | | | | | | | | | | | |  |
|  |  |  | No |  |  |  |  |  |  | |  | | | | | |  |
|  | | |  |  |  |  |  |  |  | |  | | | | | | |
|  | | | | | | | | | | | |  |  |  |  |  |  |
|  | RESPONDENT: I don’t think so it is not part of the study because the things that we want to detect for are not validated for clear use so the things that we are trying to study are not that they are ready for clinical use so you don’t really have an obligation to report back about the clinical findings of the patient however the case of the Simplex virus that I have told you we can recommend to the clinicians to re-evaluate their patients because at least the molecular tests that can detect resistance to chemotherapy however there is another way of detecting that clinically so we would recommend the clinician to tell that this patient experiences Simplex virus so we think that they might be resistant to the drugs so we recommend that you must timely detect for clinical signs of resistance to the drug in these patients | | | | | | | | | | | | | | | |  |
|  |  | | | | | | | | | | | | | | | |  |
|  | | | **Files\\Male\\R-015.male** | | | | | | | | | | | | | |  |
|  |  |  | No |  |  |  |  |  |  | |  | | | | | |  |
|  | | |  |  |  |  |  |  |  | |  | | | | | | |
|  | | | | | | | | | | | |  |  |  |  |  |  |
|  | RESPONDENT: return of results is in two ways; we have incidental findings and expected findings, usually expected findings you may not return them because you are expected to find them | | | | | | | | | | | | | | | |  |
|  |  | | | | | | | | | | | | | | | |  |
|  | | | **Files\\Male\\R-016. male** | | | | | | | | | | | | | |  |
|  |  |  | No |  |  |  |  |  |  | |  | | | | | |  |
|  | | |  |  |  |  |  |  |  | |  | | | | | | |
|  | | | | | | | | | | | |  |  |  |  |  |  |
|  | RESPONDENT: no, we don’t report back results to the participants | | | | | | | | | | | | | | | |  |
|  |  | | | | | | | | | | | | | | | |  |
|  | | | | | | | | | | | |  |  |  |  |  |  |
|  | RESPONDENT: so, at the end of the study we will, you see the study is such that we have genetic samples, DNA samples from about five countries and then we have each country having like five sites so you have 40,000 DNA samples for you to make sense out of DNA samples genetic research you need 10,000s of results so reporting or knowing your results alone is not something that helps you directly because it is of no benefit you cannot use those results to do anything, the only way we can use those results is if we have that information from those 40,000 participants collected together I hope that helps | | | | | | | | | | | | | | | |  |
|  |  | | | | | | | | | | | | | | | |  |
|  | | | **Files\\Male\\R-017. male** | | | | | | | | | | | | | |  |
|  |  |  | No |  |  |  |  |  |  | |  | | | | | |  |
|  | | |  |  |  |  |  |  |  | |  | | | | | | |
|  | | | | | | | | | | | |  |  |  |  |  |  |
|  | Respondent: We don’t report the results directly, like I said there are different media of reporting back, publishing in international journals, seminars, but also to the community. You can report back to the community that we have these findings, however this being an health based research you find that there are always statistics that are obtained from the hospital about those particular diseases and our information plays part in those statistics, yes, like you go to a population and find that these number of individuals have this particular disease, it’s a report that is also used by the health demographic surveys, so those are different media through which we report our different results | | | | | | | | | | | | | | | |  |
|  |  | | | | | | | | | | | | | | | |  |
| Reports\\Coding Summary By Code Report | | | | | | | | | | Page 81 of 109 | | | | | | | |
|  | | | | | | | | | | | | | | | | | |
|  | | | **Aggregate** |  | **Classification** |  | **Coverage** |  | **Number Of Coding References** | |  | **Reference Number** |  | **Coded By Initials** |  | **Modified On** |  |
|  | | | | | | | | | | | | | | | | | |
|  | | | | | | | | | | | |  |  |  |  |  |  |
|  | Respondent: Ahhh, me personally as a researcher I don’t have access to the individual, there is a protocol, because even if you are to get the sample, me the researcher, I don’t directly, there are procedures we have to follow, there are protocols we follow, ethical considerations passes by the ethics committee, and even dissemination of that information is through specific individuals | | | | | | | | | | | | | | | |  |
|  |  | | | | | | | | | | | | | | | |  |
|  | | | | | | | | | | | |  |  |  |  |  |  |
|  | Respondent: Yes, the researcher is the person who gets the individual sample sequences/sends it for sequencing, then me, as a bioinformatician, I analyze that data and disseminate it in a way that is understandable. So that information once it is obtained then it is also relayed to other individuals within the project who will go back to the community, like the team leader who is involved in the community engagement, he’s the one who sensitises the community and gives feed back to the community, but me as a researcher I don’t interface with the community | | | | | | | | | | | | | | | |  |
|  |  | | | | | | | | | | | | | | | |  |
|  | | | **Files\\Male\\R-019 male** | | | | | | | | | | | | | |  |
|  |  |  | No |  |  |  |  |  |  | |  | | | | | |  |
|  | | |  |  |  |  |  |  |  | |  | | | | | | |
|  | | | | | | | | | | | |  |  |  |  |  |  |
|  | Respondent: Yes, but it’s not in our interest to feed that back, especially if there is a mismatch, it’s not in our interest, not even in the interest of the community, to them you are just causing problems in the community. The social issues there, there are some social issues which none of us can solve, otherwise we are causing more problems, and those ones we avoid, that’s the strategy | | | | | | | | | | | | | | | |  |
|  |  | | | | | | | | | | | | | | | |  |
|  | | | **Files\\Male\\R-022. male** | | | | | | | | | | | | | |  |
|  |  |  | No |  |  |  |  |  |  | |  | | | | | |  |
|  | | |  |  |  |  |  |  |  | |  | | | | | | |
|  | | | | | | | | | | | |  |  |  |  |  |  |
|  | RESPONDENT: yes, we don’t report to patients because most likely they may not have the knowledge to understand what you are reporting or they may not have interpretations of this so you have to give reports to someone who can interpret what we are giving them | | | | | | | | | | | | | | | |  |
|  |  | | | | | | | | | | | | | | | |  |
|  | | | **Files\\Male\\R-023. male** | | | | | | | | | | | | | |  |
|  |  |  | No |  |  |  |  |  |  | |  | | | | | |  |
|  | | |  |  |  |  |  |  |  | |  | | | | | | |
|  | | | | | | | | | | | |  |  |  |  |  |  |
|  | Respondent: No, the one I was involved in was not necessary because I was looking things at a population level, so it was not so much on the individuals but more on a population level | | | | | | | | | | | | | | | |  |
|  |  | | | | | | | | | | | | | | | |  |
|  | | | | | | | | | | | | | | | | | |
|  | | | | | | | | | | | | | | | | | |
|  | | | | | | | | | | | | | | | | | |
| Reports\\Coding Summary By Code Report | | | | | | | | | | Page 82 of 109 | | | | | | | |
|  | | | | | | | | | | | | | | | | | |
|  | | | **Aggregate** |  | **Classification** |  | **Coverage** |  | **Number Of Coding References** | |  | **Reference Number** |  | **Coded By Initials** |  | **Modified On** |  |
|  | | | | | | | | | | | | | | | | | |
|  | | | | | | | | | | | |  |  |  |  |  |  |
|  | Interviewer: And are there repercussions if you don’t?  Respondent: They don’t. I think for my particular kind of work, I think there are no repercussions because even if you did not, the people who would use that most are those who implement disease control, so people like AIDs control Programme, because we are looking at population level dynamics so for them it would be more to answer a question like what regimens should we may be adopt in our treatment policy for the country, so for them as long as they know, that’s almost good enough because they are the ones who initiate and provide the regimens and the rest of the people follow. But I would imagine a case like where it would be good for individuals to also know, so that they can do something about it. In my case the individuals, even if they knew, they were not gonna change a regimen because we already have what’s on the market, but if it were a study that was more targeted to the individual themselves and you find probably an individual with an actual predisposition that makes them susceptible and something could be done about it, then it would be unethical not to tell them that you know we found this making you predisposed to being susceptible to HIV or this drug not working in your case, and we think that you should be taking this but it is better you take this other thing, but my work was not at that level but am sure there are instances of the research which could apply and you would be not being very ethical if you didn’t do that. But on the other hand the challenge also is that also genetics is not an exact, coz you are saying this thing is predisposing you, so we are just saying maybe having this mutation make you at more at risk of getting this, but the way things end up working is not a direct, sometimes its not even that you got it, what we are saying will happen will happen, coz there are other factors that come in to play outside the genetics, genetics is one bit, and then we have all these other things playing a role. So, again on the other hand if I told you that I analyzed your sample from my work and I saw that you have maybe this mutation that may predispose you to maybe say running mad at a later stage, but I also know that it is not a one on one, because there other things that play. So, either two things happen, either you take it and have this worry that that is going to happen, yet in reality there is still a probability that it may not. So I don’t know where one draws the line, even in those cases, my work was not on that but am just opening up on a larger, that even if it is good to tell, because genetics is not necessarily a given that it will happen, even if you have the mutant gene, because the outcome is a factor of yes the gene plays a role, but there also other factors that would have come in | | | | | | | | | | | | | | | |  |
|  |  |  |  |  |  |  |  |  |  |  |  |  |  |  |  |  |  |
|  |  |  |  |  |  |  |  |  |  |  |  |  |  |  |  |  |  |
|  |  |  |  |  |  |  |  |  |  |  |  |  |  |  |  |  |  |
|  |  | | | | | | | | | | | | | | | |  |
|  | | | **Files\\Male\\R-025 male..** | | | | | | | | | | | | | |  |
|  |  |  | No |  |  |  |  |  |  | |  | | | | | |  |
|  | | |  |  |  |  |  |  |  | |  | | | | | | |
|  | | | | | | | | | | | |  |  |  |  |  |  |
|  | Respondent: No, we haven’t. There is also another arm to that, there is a culture of Burkitt’s lymphoma, trying to see the various genetic distance with them but we haven’t. The only benefit we have given them is that we offer them, we have them with whatever treatment they will undergo. Let us say we bought those drugs and they are treated for free, they are given health education and then we connected them to a team that follows them up but we haven’t | | | | | | | | | | | | | | | |  |
|  |  | | | | | | | | | | | | | | | |  |
|  | **Nodes\\Return of results\\Results returned\Yes** | | | | | | | | | | | | | | | |  |
|  | | **Document** | | | | | | | | | | | | | | |  |
|  | | | **Files\\Female\\R-003.female** | | | | | | | | | | | | | |  |
|  |  |  | No |  |  |  |  |  |  | |  | | | | | |  |
|  | | |  |  |  |  |  |  |  | |  | | | | | | |
|  | | | | | | | | | | | |  |  |  |  |  |  |
|  | RESPONDENT: we just gave generalized information | | | | | | | | | | | | | | | |  |
|  |  | | | | | | | | | | | | | | | |  |
|  | | | | | | | | | | | | | | | | | |
| Reports\\Coding Summary By Code Report | | | | | | | | | | Page 83 of 109 | | | | | | | |
|  | | | | | | | | | | | | | | | | | |
|  | | | **Aggregate** |  | **Classification** |  | **Coverage** |  | **Number Of Coding References** | |  | **Reference Number** |  | **Coded By Initials** |  | **Modified On** |  |
|  | | | **Files\\Female\\R026 female** | | | | | | | | | | | | | |  |
|  |  |  | No |  |  |  |  |  |  | |  | | | | | |  |
|  | | |  |  |  |  |  |  |  | |  | | | | | | |
|  | | | | | | | | | | | |  |  |  |  |  |  |
|  | INTERVIEWER: Qn.3 So, are your studies of such a nature that you have to report back to your participants about their genetic makeup?  RESPONDENT: Yes, we have to give them some information of what we found. Why I say some information? Explaining genetics to anybody is complex, but we have to go back to the participants and give them, not individual, but as a group. So, we don’t give individual responses. We don’t tell a person that your genes are like this and this, because we don’t have answers yet but we give them an overview of what the study entails and what we found in our studies | | | | | | | | | | | | | | | |  |
|  |  | | | | | | | | | | | | | | | |  |
|  | | | **Files\\Female\\R028.female** | | | | | | | | | | | | | |  |
|  |  |  | No |  |  |  |  |  |  | |  | | | | | |  |
|  | | |  |  |  |  |  |  |  | |  | | | | | | |
|  | | | | | | | | | | | |  |  |  |  |  |  |
|  | INTERVIEWER: Haven’t you had cases where individuals come back to ask for their results probably since you collected samples from them?  RESPONDENT: yeah, they call but we always remind them because it is in their consent that we shall not give them their genetics because it is not for immediate benefit, so, I think there is one patient one time who called and asked about it, but when we explained to her, she had forgotten what she had signed, it was several years down the road | | | | | | | | | | | | | | | |  |
|  |  | | | | | | | | | | | | | | | |  |
|  | | | **Files\\Male\\R-004.male** | | | | | | | | | | | | | |  |
|  |  |  | No |  |  |  |  |  |  | |  | | | | | |  |
|  | | |  |  |  |  |  |  |  | |  | | | | | | |
|  | | | | | | | | | | | |  |  |  |  |  |  |
|  | Respondent: yes there are a number of times we have had to inform them of the blood group expression, some we have no idea about the blood group they express or some we only stop on the usual ABO system and antigen D of their end system know that am B positive stop there but we do extend to grouping for a number of antigens about twenty the antigens that are commonest so they have got that information that they have to express, at other times when we have done molecular studies we discovered some rare genetic expressions so like some Sicklers in Mulago we got back and informed them of the anti-usual genetic makeup that possesses complications and there is also a time when students are at this university at MUST gave me blood samples to use as regent cells I also informed them about what antigens they have | | | | | | | | | | | | | | | |  |
|  |  | | | | | | | | | | | | | | | |  |
|  | | | **Files\\Male\\R-005.male** | | | | | | | | | | | | | |  |
|  |  |  | No |  |  |  |  |  |  | |  | | | | | |  |
|  | | |  |  |  |  |  |  |  | |  | | | | | | |
|  | | | | | | | | | | | |  |  |  |  |  |  |
|  | Interviewer: Which is genetics, because it is specific to….  Respondent: Oh so we return those kind of messages, but the work was published  Interviewer: Yeah there is publishing and there is going to the individual participants and telling them the findings. Did you do that?  Respondent: We had to do that, because am attached to the group whose funding is tagged to that kind of information, this is individual funders and these funders are a collection of patients, and these patients are people who are somehow affected, may be you call it a foundation or what, so these people are interested in these results in order to encourage the people who are putting in donations and feel like this kind of work is going to benefit the people suffering from that disease | | | | | | | | | | | | | | | |  |
|  |  |  |  |  |  |  |  |  |  |  |  |  |  |  |  |  |  |
|  |  | | | | | | | | | | | | | | | |  |
|  | | | | | | | | | | | | | | | | | |
| Reports\\Coding Summary By Code Report | | | | | | | | | | Page 84 of 109 | | | | | | | |
|  | | | | | | | | | | | | | | | | | |
|  | | | **Aggregate** |  | **Classification** |  | **Coverage** |  | **Number Of Coding References** | |  | **Reference Number** |  | **Coded By Initials** |  | **Modified On** |  |
|  | | | **Files\\Male\\R-013.male** | | | | | | | | | | | | | |  |
|  |  |  | No |  |  |  |  |  |  | |  | | | | | |  |
|  | | |  |  |  |  |  |  |  | |  | | | | | | |
|  | | | | | | | | | | | |  |  |  |  |  |  |
|  | INTERVIEWER: have you ever returned such incidental findings?  RESPONDENT: yeah, there is a time I participated in clinical trial to do research things but still there was a system, where we had to follow up these patients we knew where the patients were, it was an HIV clinical trials in eastern part of the country we used to do that because we even had a GPS tracker of knowing each home of the patient and we had people who could go and track these patients deeply in the villages so its something possible but however it is very logistic in the intensive to really do  INTERVIEWER: did you have like counsellors, how did you manage?  RESPONDENT: yes, we had counsellors, trackers, some it was a big team community linkage coordinators and many more | | | | | | | | | | | | | | | |  |
|  |  |  |  |  |  |  |  |  |  |  |  |  |  |  |  |  |  |
|  |  | | | | | | | | | | | | | | | |  |
|  | | | **Files\\Male\\R-014 male** | | | | | | | | | | | | | |  |
|  |  |  | No |  |  |  |  |  |  | |  | | | | | |  |
|  | | |  |  |  |  |  |  |  | |  | | | | | | |
|  | | | | | | | | | | | |  |  |  |  |  |  |
|  | RESPONDENT: feedback is given in terms of publications, you don’t expect a village guy there to read a publication but you are informing someone in the research world or someone who is already sensitized. And some of the genetic data is deposited into the MCDI data set. Its not for sell because you didn’t buy the samples, so you are just adding a brick for someone to have a starting point in future but we have this much of Ugandan data so if I wake up and I want to use, I can use it without going back to use invasive methods to collect samples from patients. Even time, that’s why we have this bio repository because you have very many samples here for any researcher and you don’t need to look at, perhaps the patients are even dead sometimes but the samples are there. | | | | | | | | | | | | | | | |  |
|  |  | | | | | | | | | | | | | | | |  |
|  | | | **Files\\Male\\R-017. male** | | | | | | | | | | | | | |  |
|  |  |  | No |  |  |  |  |  |  | |  | | | | | |  |
|  | | |  |  |  |  |  |  |  | |  | | | | | | |
|  | | | | | | | | | | | |  |  |  |  |  |  |
|  | Respondent: Yeah, there are different ways of reporting results, one through seminars, reporting to the community, publications-reporting to the international community about your findings and actually going back to the individuals or the communities where you obtained the samples from, reporting back to them | | | | | | | | | | | | | | | |  |
|  |  | | | | | | | | | | | | | | | |  |
|  | | | **Files\\Male\\R-019 male** | | | | | | | | | | | | | |  |
|  |  |  | No |  |  |  |  |  |  | |  | | | | | |  |
|  | | |  |  |  |  |  |  |  | |  | | | | | | |
|  | | | | | | | | | | | |  |  |  |  |  |  |
|  | Respondent: There are approaches we have that are general to the community, there are things we feedback, one the progress of the research, what are we doing, how far have we gone? General information. Next, we can feed back specific findings, do you have the genetic markers for susceptibility to disease? we have not yet identified them so we cannot yet feed this back. And then the other feed back would be incidental findings but in our kind of studies the incidental findings are very rare. Because for example, when we invite the participants for this study we don’t do things like hypertension, blood sugar, those are the kind of things which would bring you incidental findings, of course there are things which we don’t do, like if we do family genomics, we do not feedback about paternity. | | | | | | | | | | | | | | | |  |
|  |  | | | | | | | | | | | | | | | |  |
|  | | | | | | | | | | | | | | | | | |
| Reports\\Coding Summary By Code Report | | | | | | | | | | Page 85 of 109 | | | | | | | |
|  | | | | | | | | | | | | | | | | | |
|  | | | **Aggregate** |  | **Classification** |  | **Coverage** |  | **Number Of Coding References** | |  | **Reference Number** |  | **Coded By Initials** |  | **Modified On** |  |
|  | | | **Files\\Male\\R-024.male** | | | | | | | | | | | | | |  |
|  |  |  | No |  |  |  |  |  |  | |  | | | | | |  |
|  | | |  |  |  |  |  |  |  | |  | | | | | | |
|  | | | | | | | | | | | |  |  |  |  |  |  |
|  | RESPONDENT: We do report back for all our genetic studies, this is particularly very easy for us when we are doing prospective studies like those I have mentioned, however there are studies that we doing on samples that we collected in 2011 we cannot trace back those patients, these are samples that are stored and we have not been able to get back to the individual patients and studies that we developed where on the samples that we already collected so we developed a research idea based on the idea that we already have the samples so those are the few studies that we have not been able to feedback, but all the rest patients consent for the genetic studies we do the analysis and then do the feedback. The beauty about what I am talking about is that we now have the real time genetic study tests and we are able to feed back to the patients as they come.  INTERVIEWER: What does that mean to the young’s physical health and social lives as individuals, families and communities?  RESPONDENT: On the whole it is very re-assuring to the parents that they have been able to know the genetic condition which is predispose to their children to illnesses so they comply on the management plan for some of these I also don’t manage I refer to my colleagues we have been able to touch these with the colleagues and tell us that the children have been attending the screenings. But there also instances where these tests have caused a bit of trouble and I am not ashamed of telling one particular case that you may have to reference that very prominent person in the region came with a very sick child, I handled the child and the child had sickle cell, so after consenting them we said lets first go through the screening test, the screening test was purely for management of the patient in the ward and to show the child may have a likelihood of sickle, then I told them, lets now do a confirmatory test because we are on the research test, so when the confirmatory test came back positive the father a very prominent person, said he doesn’t have sickle in his family and that is not his child therefore. So there those connotations, I should therefore say that this is one case out of over 3000 that we have managed but still it has its social impact and eventually it took long to convince that very prominent person and his wife to test together and when they tested carriers we were now good to explain how the child got sickle cell, but you can imagine the child had to go through stress first before eventually that is done. | | | | | | | | | | | | | | | |  |
|  |  |  |  |  |  |  |  |  |  |  |  |  |  |  |  |  |  |
|  |  |  |  |  |  |  |  |  |  |  |  |  |  |  |  |  |  |
|  |  | | | | | | | | | | | | | | | |  |
|  | | | | | | | | | | | |  |  |  |  |  |  |
|  | Respondent: Oh yeah, that one and you can imagine that the parents have to manage this child in a very special way but we’ve also had a child who developed recurrent dark urine, what is commonly called hemoglobinuria, so we eventually managed to tell the parents that look it is because the child has glucose-6-phosphate dehydrogenase, a genetic condition that has predisposed the child to this, and they were able to accept referral to Mulago and a colleague of mine managed that child in Mulago. Now the child is stable and they already know what they should do when the child develops fever or tests for malaria. So those are very out standing because they were on extreme. The other one managed to join medical school and was at medical school up to third year, then this one had over 70 blood transfusions by the time I saw the child. Now knowing this and cutting down on transfusions almost to nothing is a very, very outstanding this. So in between of course you get other mild cases | | | | | | | | | | | | | | | |  |
|  |  |  |  |  |  |  |  |  |  |  |  |  |  |  |  |  |  |
|  |  | | | | | | | | | | | | | | | |  |
|  | | | | | | | | | | | |  |  |  |  |  |  |
|  | Interviewer: Did you also return results to these communities?  Respondent: yes for instance we typed over 2000 community samples for 3 conditions, sickle, beta-thalassemia and glucose-6-phosphate dehydrogenase and those that we return, we return all the results as individual results and those that we found positive, we’ve been able to refer them to specialized centers and I think maybe up to 80% of those who were positive a being followed up by the hospital clinic | | | | | | | | | | | | | | | |  |
|  |  | | | | | | | | | | | | | | | |  |
|  | | | **Files\\Male\\R-025 male..** | | | | | | | | | | | | | |  |
|  |  |  | No |  |  |  |  |  |  | |  | | | | | |  |
|  | | |  |  |  |  |  |  |  | |  | | | | | | |
|  | | | | | | | | | | | |  |  |  |  |  |  |
|  | Respondent: This is really to see if certain communities have been predisposed to certain genetic defects and thus disposed to certain genetic diseases, we have not yet connected. We have just said, okay these families are very close to each other, the genomes are very similar to each other, the other one are very heterogenous and that is the mix that is there, eg we did a study form west Nile, Acholi and Lango sub regions, as you go toward the Lango sub region there is a lot of mix, eg in the Lango sub region there is a lot Bantu mix as well as the Nilotic mix, so we have not yet inferred anything whether it is which disease, whether to confer certain beneficial characteristic, but it is just general information, probably we already know, but we know there is a lot of mixture here, there is a lot of pure familial line here and then we are hoping that when the analysis of genetic is done, we can predict some disease itself | | | | | | | | | | | | | | | |  |
|  |  |  |  |  |  |  |  |  |  |  |  |  |  |  |  |  |  |
|  |  | | | | | | | | | | | | | | | |  |
|  | | | | | | | | | | | | | | | | | |
| Reports\\Coding Summary By Code Report | | | | | | | | | | Page 86 of 109 | | | | | | | |
|  | | | | | | | | | | | | | | | | | |
|  | | | **Aggregate** |  | **Classification** |  | **Coverage** |  | **Number Of Coding References** | |  | **Reference Number** |  | **Coded By Initials** |  | **Modified On** |  |
|  | | | | | | | | | | | | | | | | | |
|  | | | | | | | | | | | |  |  |  |  |  |  |
|  | Interviewer: And what was the outcome from that reporting so far?  Respondent: Well they just showed interest that apart from that day they wanted to know, that being few or being mixed does it confer any advantage, but we don’t have an answer for this but it is just an information about themselves, maybe somebody can further it in future but we also find that there are many tribes which have already been matched but for the nilotics this might have been the first matching or let’s say the genetic mix in the society called this, so we use like ganda, we use there is a tribe Luo in this other eastern part, but the Luo blood was very few, then we also use the West African. the results of the west African blood but the mix is not so bad, there is a little mix but the genetic mix is not so bad because those are the tribes which already have supplementary map | | | | | | | | | | | | | | | |  |
|  |  |  |  |  |  |  |  |  |  |  |  |  |  |  |  |  |  |
|  |  | | | | | | | | | | | | | | | |  |
|  | **Nodes\\Return of results\\Results returned\Yes\Conditions of return** | | | | | | | | | | | | | | | |  |
|  | | **Document** | | | | | | | | | | | | | | |  |
|  | | | **Files\\Female\\R-003.female** | | | | | | | | | | | | | |  |
|  |  |  | No |  |  |  |  |  |  | |  | | | | | |  |
|  | | |  |  |  |  |  |  |  | |  | | | | | | |
|  | | | | | | | | | | | |  |  |  |  |  |  |
|  |  | | | | | | | | | | | | | | | |  |
|  |  | | | | | | | | | | | | | | | |  |
|  | | | **Files\\Female\\R028.female** | | | | | | | | | | | | | |  |
|  |  |  | No |  |  |  |  |  |  | |  | | | | | |  |
|  | | |  |  |  |  |  |  |  | |  | | | | | | |
|  | | | | | | | | | | | |  |  |  |  |  |  |
|  | INTERVIEWER: Haven’t you had cases where individuals come back to ask for their results probably since you collected samples from them?  RESPONDENT: yeah, they call but we always remind them because it is in their consent that we shall not give them their genetics because it is not for immediate benefit, so, I think there is one patient one time who called and asked about it, but when we explained to her, she had forgotten what she had signed, it was several years down the road | | | | | | | | | | | | | | | |  |
|  |  | | | | | | | | | | | | | | | |  |
|  | | | | | | | | | | | |  |  |  |  |  |  |
|  | INTERVIEWER: But if you are to return results, under what conditions do you think results should be returned?  RESPONDENT: I think it is actually the same question | | | | | | | | | | | | | | | |  |
|  |  | | | | | | | | | | | | | | | |  |
|  | | | **Files\\Male\\R-002. male** | | | | | | | | | | | | | |  |
|  |  |  | No |  |  |  |  |  |  | |  | | | | | |  |
|  | | |  |  |  |  |  |  |  | |  | | | | | | |
|  | | | | | | | | | | | |  |  |  |  |  |  |
|  | Respondent: well, we haven’t done that as well but me as me what I think what would be important would be to hopefully would be having contacts of these people or the community where the study was done so we can inform them and I think that’s where we need to go especially we research at some point to inform the local leaders to get the people because in this case the information we will be giving will be general not particular person but something that the community can take would be good to involve local leaders to get people together and then go and give them feedback | | | | | | | | | | | | | | | |  |
|  |  | | | | | | | | | | | | | | | |  |
| Reports\\Coding Summary By Code Report | | | | | | | | | | Page 87 of 109 | | | | | | | |
|  | | | | | | | | | | | | | | | | | |
|  | | | **Aggregate** |  | **Classification** |  | **Coverage** |  | **Number Of Coding References** | |  | **Reference Number** |  | **Coded By Initials** |  | **Modified On** |  |
|  | | | | | | | | | | | | | | | | | |
|  | | | | | | | | | | | |  |  |  |  |  |  |
|  | Respondent: yes, community engagement | | | | | | | | | | | | | | | |  |
|  |  | | | | | | | | | | | | | | | |  |
|  | | | | | | | | | | | |  |  |  |  |  |  |
|  | Respondent: health service providers or health care workers as well because it will be very important to have both the community and the health care service providers that is working in the community | | | | | | | | | | | | | | | |  |
|  |  | | | | | | | | | | | | | | | |  |
|  | | | | | | | | | | | |  |  |  |  |  |  |
|  | Respondent: am now thinking something different from what am working on at the moment because for me am looking at genetics of the bacteria and other things but now we are talking about genetics of human beings | | | | | | | | | | | | | | | |  |
|  |  | | | | | | | | | | | | | | | |  |
|  | | | | | | | | | | | |  |  |  |  |  |  |
|  | Respondent: we need to have some guidelines and polices in place especially for like when people do clinical research or studies that goes deep to look at the genetics of individuals in a hope of coming with better treatment options for these people and it means we will need to have kind of technology diagnosis in place which can quickly get us this kind of results and then we are able to tailor the treatment with in the timing of this person to get treatment | | | | | | | | | | | | | | | |  |
|  |  | | | | | | | | | | | | | | | |  |
|  | | | | | | | | | | | |  |  |  |  |  |  |
|  | Interviewer: so, you think it will discuss with regulators to tell you its ok to return results to an individual or to give you the procedure to follow  Respondent: yes, I think, I will imagine it will be good to get to know if there are procedures in place how to handle this kind of information as down to people | | | | | | | | | | | | | | | |  |
|  |  | | | | | | | | | | | | | | | |  |
|  | | | **Files\\Male\\R-004.male** | | | | | | | | | | | | | |  |
|  |  |  | No |  |  |  |  |  |  | |  | | | | | |  |
|  | | |  |  |  |  |  |  |  | |  | | | | | | |
|  | | | | | | | | | | | |  |  |  |  |  |  |
|  | Respondent: procedures this was easy the population was determined to get back to the record to inform the parents in case of the Sicklers to get back the record and inform the students for the sake of the regent cell donors just using the records we had kept in the data collection | | | | | | | | | | | | | | | |  |
|  |  | | | | | | | | | | | | | | | |  |
|  | | | | | | | | | | | |  |  |  |  |  |  |
|  | Respondent: there is a consent form that has been designed before the consent the study is explained to the participants, say the adults or parents of the minors that genetic aspect is talked about for example that there is, individuals are different because of the genes and because of this, this could occur after a transfusion and that why we are doing this research so there is that basic information and still I have not had community involvement to give them the information it stays that it is confidential to the individual and the family not taking it to the community level | | | | | | | | | | | | | | | |  |
|  |  | | | | | | | | | | | | | | | |  |
|  | | | **Files\\Male\\R-005.male** | | | | | | | | | | | | | |  |
|  |  |  | No |  |  |  |  |  |  | |  | | | | | |  |
|  | | |  |  |  |  |  |  |  | |  | | | | | | |
|  | | | | | | | | | | | |  |  |  |  |  |  |
|  | Respondent: What do you mean conditions? When results are good or bad? Fortunately, the results were good, people were excited and they had to go and tell everyone that you know what this has potential…but we had to tell them that that is potential not that it is the solution, maybe it could improve your life because some of the diseases like, if survival is increased may be one year or so | | | | | | | | | | | | | | | |  |
|  |  | | | | | | | | | | | | | | | |  |
|  | | | | | | | | | | | | | | | | | |
| Reports\\Coding Summary By Code Report | | | | | | | | | | Page 88 of 109 | | | | | | | |
|  | | | | | | | | | | | | | | | | | |
|  | | | **Aggregate** |  | **Classification** |  | **Coverage** |  | **Number Of Coding References** | |  | **Reference Number** |  | **Coded By Initials** |  | **Modified On** |  |
|  | | | | | | | | | | | | | | | | | |
|  | | | | | | | | | | | |  |  |  |  |  |  |
|  | Interviewer: In other words, you must be well trained, and understanding what you are communicating and you have to be sensitive to the participants. And then the other way?  Respondent: Yeah I was talking about preparing yourself and the community, the kind of information they are going to receive, and probably you don’t need to be the one communicating, probably you can use a third person, people who are ready. We used to have in the other, those guys had a genetic counseling group but those guys were under psychology not like us in basic sciences, in Norway. So those are the guys who need to do that kind of communication. So they are usually people trained to do that kind of work  Interviewer: And how do you prepare the community?  Respondent: That’s a question I don’t know, how do you prepare the community?  Interviewer: Would informed consent before you do the study be part of that preparation?  Respondent: I think that is may be the study design from the beginning. Like the informed consent, like these are the possible results we are going to get, these are the possible implications on what we are going to research on, maybe if we are getting this it may mean this, am testing you but it may be affecting your community, your children e.t.c. I think this preparation is done before the project starts, so long long before the actual results come out | | | | | | | | | | | | | | | |  |
|  |  |  |  |  |  |  |  |  |  |  |  |  |  |  |  |  |  |
|  |  | | | | | | | | | | | | | | | |  |
|  | | | **Files\\Male\\R-006. male** | | | | | | | | | | | | | |  |
|  |  |  | No |  |  |  |  |  |  | |  | | | | | |  |
|  | | |  |  |  |  |  |  |  | |  | | | | | | |
|  | | | | | | | | | | | |  |  |  |  |  |  |
|  | I Think ,for these kind of genetics i am not really sure yet for research information like this, i am not sure what. if it is something that is directly responsible for a condition or if a person has Thalassemia or somebody has any of a kind of genetic diseases, like i mentioned, it’s not something we have had to do here. But if its a genetic condition for sure and the persons is experiencing what they are experiencing because of a genetic make-up, like causing ill health to them. i think it would have to be treated the same way you communicate issues to do with non-communicable diseases say diabetes or hypertension. you explain to the person the condition they have, where they are coming from, what can be done to have them live a long and fruitful life, those would be my thoughts really. | | | | | | | | | | | | | | | |  |
|  |  | | | | | | | | | | | | | | | |  |
|  | | | | | | | | | | | |  |  |  |  |  |  |
|  | So, if the condition the person is presenting with is actually directly linked to the genetics, yes, and if the person is reacting to either treatment or the environment because of the genetic formulation, I think that would be necessary to send back these results to them. | | | | | | | | | | | | | | | |  |
|  |  | | | | | | | | | | | | | | | |  |
|  | | | **Files\\Male\\R-007 male** | | | | | | | | | | | | | |  |
|  |  |  | No |  |  |  |  |  |  | |  | | | | | |  |
|  | | |  |  |  |  |  |  |  | |  | | | | | | |
|  | | | | | | | | | | | |  |  |  |  |  |  |
|  | So at the moment, ohoo, the plan, if we do that, we want to entirely strictly use the identified data. We don’t want people to, we don’t want that data to be in any way connected to an individual directly, so we can make conclusions basing on the data we have, but we don’t have to be able to connect it to any particular individual. At the moment that’s what we are trying to do. We don’t, we’re not looking at the individual or the family or anything to come back and report to them, if we are to report, we’d report in a general term in a general manner and we wouldn’t have, even we wouldn’t have the information to link a particular individual at all. That’s what we are thinking about. | | | | | | | | | | | | | | | |  |
|  |  | | | | | | | | | | | | | | | |  |
|  | | | | | | | | | | | |  |  |  |  |  |  |
|  | RESPONDENT: Uhmm, so ahaa, obviously you must think about what those results mean? What do they mean? Do they mean that this particular population you know, ends up being targeted as a disadvantaged population or an inferior population in one way or the other, then again also, if you look at the other side also if you want to generalize that community as superior kind of population in one way, also it has implications, so at the moment really I think we are still in the early phases and its going to take a process of really getting to determine what can we really pass on to the participants or to a researcher when it comes to a genetic. But for the moment we are trying to avoid, well even if it to be identified, the whole community can be identified but the individuals and families may not be. So we need to decide and say okay, what can we really pass on to the community and that’s not yet we determine conclusively on what should be. And I only have a direct answer on what can we and what can we not? | | | | | | | | | | | | | | | |  |
|  |  |  |  |  |  |  |  |  |  |  |  |  |  |  |  |  |  |
|  |  | | | | | | | | | | | | | | | |  |
|  | | | | | | | | | | | | | | | | | |
| Reports\\Coding Summary By Code Report | | | | | | | | | | Page 89 of 109 | | | | | | | |
|  | | | | | | | | | | | | | | | | | |
|  | | | **Aggregate** |  | **Classification** |  | **Coverage** |  | **Number Of Coding References** | |  | **Reference Number** |  | **Coded By Initials** |  | **Modified On** |  |
|  | | | **Files\\Male\\R-009.male** | | | | | | | | | | | | | |  |
|  |  |  | No |  |  |  |  |  |  | |  | | | | | |  |
|  | | |  |  |  |  |  |  |  | |  | | | | | | |
|  | | | | | | | | | | | |  |  |  |  |  |  |
|  | RESPONDENT: the research team according to the way we have designed that if we were to report back we do have the community advisory board. they would whatever the results then the community advisory board would guide in terms of how the sensitivity of the matter, the impact and how we go about this disclosure and of course that is the mechanism we do have to disclose of course I have not gone through that but that’s how we have designed our studies | | | | | | | | | | | | | | | |  |
|  |  | | | | | | | | | | | | | | | |  |
|  | | | | | | | | | | | |  |  |  |  |  |  |
|  | RESPONDENT: some of them do others really don’t but we just hope that we genuinely explain to them what it could mean that they understand the sensitivities in the society and what our implication is and guide how best to do that for sure these are very difficult discussions in terms of genetics and which I don’t know they are very difficult for us to have any correct or wrong | | | | | | | | | | | | | | | |  |
|  |  | | | | | | | | | | | | | | | |  |
|  | | | | | | | | | | | |  |  |  |  |  |  |
|  | INTERVIEWER: so, does it mean that the community advisory board should work with communities to do some form of community engagement?  RESPONDENT: yes, there is a lot of community engagement in the studies we are doing especially lately I will have to be sincere that this field is growing because by the time we started the relapses of TB it wasn’t that advanced but H3Africa we do have community advisory board not only that but the community where we have been involved for example radio programs, we have booklets to explain about what are genomics studies, efforts to people to discover need and talk about this so that the community is sensitized so that there are somehow prepared there have been debates for H3African we have engaged the ethical review board they have been there because they are the experts there are anthropologists, there are provided in the consent process the constitution of the community advisory board so we were not experts in that area, this consortium has helped to how best to guide us in this, this framework I think by trying to empower the ethical committees and ethical boards are probably try really to translated that in our own country I remember I was once invited by Uganda national council for science and technology to give a talk we think that way the counties and the communities get better prepared to handle this information where there is no easy answer | | | | | | | | | | | | | | | |  |
|  |  |  |  |  |  |  |  |  |  |  |  |  |  |  |  |  |  |
|  |  | | | | | | | | | | | | | | | |  |
|  | | | | | | | | | | | |  |  |  |  |  |  |
|  | RESPONDENT: well like I have told you for me the studies I have gotten actually we are the people who do those studies in Baylor college where we take those children and we do those studies we have not been able to get some discoveries to report back so again if we had as we said that is the mechanism, we are looking forward to using | | | | | | | | | | | | | | | |  |
|  |  | | | | | | | | | | | | | | | |  |
|  | | | | | | | | | | | |  |  |  |  |  |  |
|  | RESPONDENT: we could return results may be if we think that there has been argument of course may be if there is a product which could be commercialized and then if you are in that case coz still again the result which makes a meaning so in my view for now I think that is it and it depends coz I don’t I don’t what to a community it means for example I don’t know if we collected a sample and brought it to the university like here then we analyzed the results it is very important that results get to be known in the institution and they get to be known in the country because that is public information in a certain way especially if it does not breach confidentiality and there is no meaning so anyway for me in my view that is what I think | | | | | | | | | | | | | | | |  |
|  |  | | | | | | | | | | | | | | | |  |
|  | | | | | | | | | | | |  |  |  |  |  |  |
|  | meaning it differs from one person to another  RESPONDENT: and you can’t be the one to judge that’s what this community advisory board might tell you on sensitivities may be the community is likely to behave like this | | | | | | | | | | | | | | | |  |
|  |  | | | | | | | | | | | | | | | |  |
|  | | | | | | | | | | | |  |  |  |  |  |  |
|  | RESPONDENT: and these things are found and those are the issues which I think they have legal implications and probably people may not have consented for them so again you really have to rethink and then probably get advice on how best to handle these things like you have said context matters | | | | | | | | | | | | | | | |  |
|  |  | | | | | | | | | | | | | | | |  |
|  | | | | | | | | | | | |  |  |  |  |  |  |
|  | RESPONDENT: I have been reading right now for example if you can look at the country guidelines you might be knowing very well these guidelines they are not there they are may be in development but they are not there when we look at the research protocol again they are not clear they are just saying if something could have meaning so it leaves to your interpretation so it is not clear and of course even those frameworks are very hard that we need to keep thinking through, last time I was reading about a mother who was told a child was not theirs so this is where we think about the father but the mother was told this child is not theirs and it turned out actually that the child was not yours but the way the genetic information was, people who are two in one person so they have one half genetic information and another half genetic information | | | | | | | | | | | | | | | |  |
|  |  |  |  |  |  |  |  |  |  |  |  |  |  |  |  |  |  |
|  |  | | | | | | | | | | | | | | | |  |
| Reports\\Coding Summary By Code Report | | | | | | | | | | Page 90 of 109 | | | | | | | |
|  | | | | | | | | | | | | | | | | | |
|  | | | **Aggregate** |  | **Classification** |  | **Coverage** |  | **Number Of Coding References** | |  | **Reference Number** |  | **Coded By Initials** |  | **Modified On** |  |
|  | | | **Files\\Male\\R-010. male** | | | | | | | | | | | | | |  |
|  |  |  | No |  |  |  |  |  |  | |  | | | | | |  |
|  | | |  |  |  |  |  |  |  | |  | | | | | | |
|  | | | | | | | | | | | |  |  |  |  |  |  |
|  | They have consented that you should provide this information. You providing this information is likely to break up this family. I think this scenario is going to be examined and the consent of the participant is very important because no matter what you found, unless it’s of public health concern or it is such that it is fatal to the patient because we’ve had genetic diseases like Marphan’s syndrome; this is a disease of connective tissue and these patients’ biggest problem is that their valves of the heart are affected and they are usually children so if you leave them to just go and perform duties, exercise and things, it’s possible that the child will…these are preventable things. If you know this child is pre-disposed and you tell the family. | | | | | | | | | | | | | | | |  |
|  |  | | | | | | | | | | | | | | | |  |
|  | | | | | | | | | | | |  |  |  |  |  |  |
|  | RESPONDENT: of course you have to go back looking through the consent. If you find something that is really important, you back look into the consent if the patient consented you have to invite the patient back. Literally in a normal context this is supposed to be a genetic counsellor to give this information and genetic counselling is not just psychosocial support but it’s about talking about the mechanisms different disease or things come about so that the patient can understand the options for testing, treatment before you even begin telling them the problem has been found. Once that has been found, you need health support, psychosocial and reveal whatever you found. So, psychosocial support is a very small section of genetic counselling. Ideally this should be a genetic counsellor to offer this information. I have a PhD student who actually asked me to offer any loose out spark to the patients. I have done some genetic counselling training for nurses. By the way, in the USA genetic counselling is a super specialty and it’s a place for nurses especially. Why do we use nurses because they have already medical background and they have the legal right to interact with patients so you just can’t pick anybody and they turn into a genetic counsellor because there are things to do with things that govern medical practice that also govern this practice of genetic counselling. Recently we trained nurses in the Baylor Pediatric Centre of Excellence because that is our recruitment site for the CafGEN study which is looking at children who are born with HIV and how genetics is affecting disease progression both for HIV and TB. The idea was that we could find some information that we need to give back to these participants. I tried to convince the cancer institute to also take up genetic some genetic counseling because we have some cancers which are genetic and other cancers are superionic so if you have a genetic counselor, it is important that you form your…that they can begin…because as you…like I said, you get an incidental finding but is of interest to the public to the wider community so sometimes the decision to hold on to this information is very difficult even if the patient has not consented. You wonder if there should be scenarios where the IRB should take over the mandate to override and make sure this information is given to these people.  INTERVIEWER: how about community consent?  RESPENDENT: the idea about community consent is sometime that evolved in the ideal of the African context because we got to realize that beyond the individual level, the leadership within the community is very important so the consent was just not limited to the individual subjects in the study, we had to move on to the leadership. Yes, I agree, maybe community consent might be of use in the context of this kind of thing then communities have to deal with issues of return of results. | | | | | | | | | | | | | | | |  |
|  |  |  |  |  |  |  |  |  |  |  |  |  |  |  |  |  |  |
|  |  |  |  |  |  |  |  |  |  |  |  |  |  |  |  |  |  |
|  |  |  |  |  |  |  |  |  |  |  |  |  |  |  |  |  |  |
|  |  | | | | | | | | | | | | | | | |  |
|  | | | | | | | | | | | |  |  |  |  |  |  |
|  | RESPENDENT: it depends, I mean you see, you want to say breach of confidentiality, No. if you see these are two parties; one party says NO the other party says lets go for the test which can come back either positive or negative, in fact by the time you finish with your Brac because we don’t each and every test on the Brac gene of somebody whom the woman is suspected to be. So, they should consent in the process that’s why we’ve always urged that even for paternity tests. There are a lot of genetic tests that are clinical for other purposes, there should be genetic counselling more before. So, what you are trying to say probably is not a breach, people are not given enough information. | | | | | | | | | | | | | | | |  |
|  |  | | | | | | | | | | | | | | | |  |
|  | | | | | | | | | | | |  |  |  |  |  |  |
|  | RESPONDENT: I agree, recently I was talking to a journalist after the death of the Ugandan business man; they got the son in two weeks’ time asking him who is running the business right now and the son chases them way and one of the journalists stops me and says; you know, I am frustrated, I was writing a story and… I was like; you are being unethical and not being human, this person is still grieving. For me I have always wanted to ask; do some of these social scientists have ethics like do journalists have a specific ethics training! | | | | | | | | | | | | | | | |  |
|  |  | | | | | | | | | | | | | | | |  |
|  | | | | | | | | | | | |  |  |  |  |  |  |
|  | RESPENDENT: okay, what I am trying to argue here is that it is important even social scientists like journalists really should involve an ethical code. If a journalist comes with a case which is affecting people, it has a medical issues and they are busy disseminating this information anywhere. | | | | | | | | | | | | | | | |  |
|  |  | | | | | | | | | | | | | | | |  |
|  | | | | | | | | | | | | | | | | | |
| Reports\\Coding Summary By Code Report | | | | | | | | | | Page 91 of 109 | | | | | | | |
|  | | | | | | | | | | | | | | | | | |
|  | | | **Aggregate** |  | **Classification** |  | **Coverage** |  | **Number Of Coding References** | |  | **Reference Number** |  | **Coded By Initials** |  | **Modified On** |  |
|  | | | **Files\\Male\\R-011.male** | | | | | | | | | | | | | |  |
|  |  |  | No |  |  |  |  |  |  | |  | | | | | |  |
|  | | |  |  |  |  |  |  |  | |  | | | | | | |
|  | | | | | | | | | | | |  |  |  |  |  |  |
|  | No, but us we don't report directly to individuals because in all genetic studies ,we have to de-identify the data, we cannot trace back the individuals, its one of the ethical aspect of this, we work with de-identified data. We can’t report directly to individuals but we can report to community. Yes maybe aggregated results but nothing personal. | | | | | | | | | | | | | | | |  |
|  |  | | | | | | | | | | | | | | | |  |
|  | | | | | | | | | | | |  |  |  |  |  |  |
|  | its de-identified, so anything that can identify that individual because based on your DNA profile, I can know who you are even if you don’t tell me your name. so we, there is no name, the only thing we have is gender, age and maybe the clinical perspective, nothing more. anything that can identify the person is removed from the data. | | | | | | | | | | | | | | | |  |
|  |  | | | | | | | | | | | | | | | |  |
|  | | | | | | | | | | | |  |  |  |  |  |  |
|  | RESPONDENT:  so our samples all came from the same clinic, the center of excellence clinic in Mulago, we report our results to those clinics because those clinics are working with us. Going back to the individuals who collected these is six years after we got samples is not an easy thing to do, but because the patients go to the clinics, they can receive whatsoever information. | | | | | | | | | | | | | | | |  |
|  |  | | | | | | | | | | | | | | | |  |
|  | | | **Files\\Male\\R-012.male** | | | | | | | | | | | | | |  |
|  |  |  | No |  |  |  |  |  |  | |  | | | | | |  |
|  | | |  |  |  |  |  |  |  | |  | | | | | | |
|  | | | | | | | | | | | |  |  |  |  |  |  |
|  | RESPONDENT: for my kind of work really like I have told you gene expression there is no circumstance that I have across or that I would think of that necessitate me returning results | | | | | | | | | | | | | | | |  |
|  |  | | | | | | | | | | | | | | | |  |
|  | | | | | | | | | | | |  |  |  |  |  |  |
|  | RESPONDENT: actually that’s why it is important that the participants are meant to know so I think it depends on how the patients have consented at least from my training for me to review an incidental finding especially to the patient maybe to the clinic, to the patient it would depend on how you’ve consented but I for one if I was involved in a genetic study I wouldn’t want to them to tell me. | | | | | | | | | | | | | | | |  |
|  |  | | | | | | | | | | | | | | | |  |
|  | | | | | | | | | | | |  |  |  |  |  |  |
|  | RESPONDENT: me as a researcher depending on the consent time I think I would report first to the clinician then if at all the patient considered being told I would tell them | | | | | | | | | | | | | | | |  |
|  |  | | | | | | | | | | | | | | | |  |
|  | | | **Files\\Male\\R-013.male** | | | | | | | | | | | | | |  |
|  |  |  | No |  |  |  |  |  |  | |  | | | | | |  |
|  | | |  |  |  |  |  |  |  | |  | | | | | | |
|  | | | | | | | | | | | |  |  |  |  |  |  |
|  | RESPONDENT: us we recommend to the clinicians upon seeing the patients because then if I see this I should evaluate for signs of resistance like raising PSA then recurrence of the tumor size and all that and may be any signs of worsening of the disease those could show the clinical signs of resistance in the chemotherapy otherwise this is not something that we can just enforce for change of therapy so we need to recommend clinicians in the course to make sure that they see it fit to follow up these patients more closely the ones that could be with the signs | | | | | | | | | | | | | | | |  |
|  |  | | | | | | | | | | | | | | | |  |
|  | | | | | | | | | | | |  |  |  |  |  |  |
|  | RESPONDENT: to inform to summarize that inform in a way to inform them the benefit of the study visa veer the outcomes so the community engagement component to show them ok this is what we did for example the kind of work that I did for the neuropathogenes it could really be reported back to the patient and this is something really applicable to the patients provide the results all the time tell them of what drug they are resistant to and how to guide on treatment that is what we did because the information was easily understandable | | | | | | | | | | | | | | | |  |
|  |  | | | | | | | | | | | | | | | |  |
| Reports\\Coding Summary By Code Report | | | | | | | | | | Page 92 of 109 | | | | | | | |
|  | | | | | | | | | | | | | | | | | |
|  | | | **Aggregate** |  | **Classification** |  | **Coverage** |  | **Number Of Coding References** | |  | **Reference Number** |  | **Coded By Initials** |  | **Modified On** |  |
|  | | | | | | | | | | | | | | | | | |
|  | | | | | | | | | | | |  |  |  |  |  |  |
|  | RESPONDENT: but you see in the ethics bit of it do no harm which is the one of the cardinal principles of ethics is that if you’re doing a study and that one of the outcomes of the study is that showing something that could affect the patient you’re obliged to give the results you’re obliged to do the reporting however like I said certain studies many of them that we do don’t necessitate or directly affect the patient so in such a scenario its normally extrapolate and just say briefly this study, in the consent you try to explain to your patient why your study is important and you say possibly this study could help do ABCD depending but in such a scenario, it might not necessarily have to report back because the outcome of the study do not affect the clinical | | | | | | | | | | | | | | | |  |
|  |  | | | | | | | | | | | | | | | |  |
|  | | | **Files\\Male\\R-014 male** | | | | | | | | | | | | | |  |
|  |  |  | No |  |  |  |  |  |  | |  | | | | | |  |
|  | | |  |  |  |  |  |  |  | |  | | | | | | |
|  | | | | | | | | | | | |  |  |  |  |  |  |
|  | RESPONDENT: they have their contacts so they give them feedback.  INTERVIEWER: through phone  RESPONDENT: yes, in most cases. | | | | | | | | | | | | | | | |  |
|  |  | | | | | | | | | | | | | | | |  |
|  | | | | | | | | | | | |  |  |  |  |  |  |
|  | RESPONDENT: you know people are educated at different levels so you can’t honestly call someone and you are like; your mutation is G, what. So, just be straight to the point like; the good news is your TB is treatable, that is enough. So, you tell them the good news, if it is bad news resort to palliative care. | | | | | | | | | | | | | | | |  |
|  |  | | | | | | | | | | | | | | | |  |
|  | | | | | | | | | | | |  |  |  |  |  |  |
|  | RESPONDENT: when you look at the principles of ethics, at least the society from which the samples came, you can’t reach out to every one because they consented that you are using their data to help the community. At times they may never even understand what you are saying but you are just thinking that maybe in future their kids, relatives or whichever relation or you as a research are availing things for the benefit. | | | | | | | | | | | | | | | |  |
|  |  | | | | | | | | | | | | | | | |  |
|  | | | | | | | | | | | |  |  |  |  |  |  |
|  | RESPONDENT: you need to tell those who are affected because remember, things like stigmatization. If you say the following ten have HIV, you are creating problems for those people because they will be segregated. But you can find a way of reaching out to them but how should you do it, get their contacts call them wherever they are, invite them over and explain the results and give possible recommendation. | | | | | | | | | | | | | | | |  |
|  |  | | | | | | | | | | | | | | | |  |
|  | | | | | | | | | | | |  |  |  |  |  |  |
|  | RESPONDENT: you will agree with me that the language used in basic science is a little complicated. There is always a way you can communicate but it’s better to communicate that effect in the most understandable language to the patient. For publication purposes yes we do. You have seen sections of publications where even though we were looking at this, we also noticed that these populations have these certain characteristics. It is out of the scope of this study but it is also important to note. You can still propose and get other funding for that but you didn’t sit on that data. You are also highlighting for someone who didn’t know. | | | | | | | | | | | | | | | |  |
|  |  | | | | | | | | | | | | | | | |  |
|  | | | | | | | | | | | |  |  |  |  |  |  |
|  | RESPONDENT: yeah. But communication is very key. Actually even a mentor in science will tell you; don’t sit on anything interesting, tell it someone as you pursue it. Some old researchers used to say “its my thing” | | | | | | | | | | | | | | | |  |
|  |  | | | | | | | | | | | | | | | |  |
|  | | | **Files\\Male\\R-016. male** | | | | | | | | | | | | | |  |
|  |  |  | No |  |  |  |  |  |  | |  | | | | | |  |
|  | | |  |  |  |  |  |  |  | |  | | | | | | |
|  | | | | | | | | | | | |  |  |  |  |  |  |
|  | RESPONDENT: yes, so for us the only thing that we report is not particularly related to the genetics study for example if you’re assessing the patient and you find out that the participant is suicidal then you must report to health workers if you find that the participant is homicidal we measure blood pressure if we find that the blood pressure is extremely high then you must report to the health care worker and then that person needs to get treatment but that is not an incidental finding it is not a finding that we stumble across that is a finding that we expect, we expect that some people may be suicidal, some people may be homicidal, some having high blood pressure and some people maybe psychologically stressed | | | | | | | | | | | | | | | |  |
|  |  | | | | | | | | | | | | | | | |  |
| Reports\\Coding Summary By Code Report | | | | | | | | | | Page 93 of 109 | | | | | | | |
|  | | | | | | | | | | | | | | | | | |
|  | | | **Aggregate** |  | **Classification** |  | **Coverage** |  | **Number Of Coding References** | |  | **Reference Number** |  | **Coded By Initials** |  | **Modified On** |  |
|  | | | **Files\\Male\\R-017. male** | | | | | | | | | | | | | |  |
|  |  |  | No |  |  |  |  |  |  | |  | | | | | |  |
|  | | |  |  |  |  |  |  |  | |  | | | | | | |
|  | | | | | | | | | | | |  |  |  |  |  |  |
|  | Respondent: Ahhh, me personally as a researcher I don’t have access to the individual, there is a protocol, because even if you are to get the sample, me the researcher, I don’t directly, there are procedures we have to follow, there are protocols we follow, ethical considerations passes by the ethics committee, and even dissemination of that information is through specific individuals | | | | | | | | | | | | | | | |  |
|  |  | | | | | | | | | | | | | | | |  |
|  | | | **Files\\Male\\R-018.male** | | | | | | | | | | | | | |  |
|  |  |  | No |  |  |  |  |  |  | |  | | | | | |  |
|  | | |  |  |  |  |  |  |  | |  | | | | | | |
|  | | | | | | | | | | | |  |  |  |  |  |  |
|  | Respondent: Yes, we have not started giving this but our colleague is the one responsible for this, he is actually thinking of going for such things such as genetic counselling, because we need this person special, he wants to take short courses. We have a platform in H3Afica which provides such opportunities, so he wants to do that, because we need to take a professional touch to it | | | | | | | | | | | | | | | |  |
|  |  | | | | | | | | | | | | | | | |  |
|  | | | **Files\\Male\\R-019 male** | | | | | | | | | | | | | |  |
|  |  |  | No |  |  |  |  |  |  | |  | | | | | |  |
|  | | |  |  |  |  |  |  |  | |  | | | | | | |
|  | | | | | | | | | | | |  |  |  |  |  |  |
|  | Respondent: No, the results will definitely tell us because we have sequenced all the genomes. It will be very obvious. The only thing we will do for that family especially Schistosomiasis, we will just take there sample out, we cannot progress with it, yes. That’s the only way to manage it | | | | | | | | | | | | | | | |  |
|  |  | | | | | | | | | | | | | | | |  |
|  | | | **Files\\Male\\R-023. male** | | | | | | | | | | | | | |  |
|  |  |  | No |  |  |  |  |  |  | |  | | | | | |  |
|  | | |  |  |  |  |  |  |  | |  | | | | | | |
|  | | | | | | | | | | | |  |  |  |  |  |  |
|  | Interviewer: So, if you intend to communicate back, what procedures should be in place?  Respondent: For me I would rather there is a proper system like we had for HIV, the way we do HIV testing like before they give you the results, they first sit you down and explain to you that really this is what it really means even though we are saying this, in genetics it is not a given that it may come out like that, but may be these are the things you can do to make that outcome not materialize so that you are well prepared, than someone just coming and telling you, you know we found this mutation that predisposes you to this so it means you are likely to get this in the next 10 years, but it is still a likelihood and it could ruin your life forcefully, you don’t know how people take things, but also the other issue is that, see I tell that in community, if it is not kept confidential it can also ruin your life in other ways, sometimes you could be discriminated against, am just imagining. SO if your employers knew that they genotyped you and you have a job that could predispose you to say running mad in the next ten years, they don’t want to give you a job, they do not invest a lot in you because they know you might run mad and so waste the investment, so there are all those things that have to be. SO I think the right is there, but I think for me for genetics because it is not an exact science and even if it were, it has those implications that can go beyond just you, coz it can also affect your life in society, you are not married and then your potential partners may fear you, so I think it is not a matter of just giving people results | | | | | | | | | | | | | | | |  |
|  |  |  |  |  |  |  |  |  |  |  |  |  |  |  |  |  |  |
|  |  | | | | | | | | | | | | | | | |  |
|  | | | | | | | | | | | |  |  |  |  |  |  |
|  | Respondent: That is my thinking, for those which you have good solution, and things can be fixed that are achievable maybe you can just give and tell them this is what you need to do, but for those where there is no solution then you might need to add another layer for counselling and explaining things properly so that they put it into proper perspective and not to blow it out of proportion, so I think it may mean more than what the current study funding allows because the way projects come, they don’t come with those other component that am suggesting, usually we are more focused on the data and then we have the results and then what we do with the results, maybe we can tell people, maybe we don’t. | | | | | | | | | | | | | | | |  |
|  |  | | | | | | | | | | | | | | | |  |
| Reports\\Coding Summary By Code Report | | | | | | | | | | Page 94 of 109 | | | | | | | |
|  | | | | | | | | | | | | | | | | | |
|  | | | **Aggregate** |  | **Classification** |  | **Coverage** |  | **Number Of Coding References** | |  | **Reference Number** |  | **Coded By Initials** |  | **Modified On** |  |
|  | | | **Files\\Male\\R-024.male** | | | | | | | | | | | | | |  |
|  |  |  | No |  |  |  |  |  |  | |  | | | | | |  |
|  | | |  |  |  |  |  |  |  | |  | | | | | | |
|  | | | | | | | | | | | |  |  |  |  |  |  |
|  | RESPONDENT: The participants who come through our research program go through a very long process of counseling, for instance one of the studies that I can tell you, the rich study we first of all take two months and during that time we have been counseling we have been giving them opportunity to ask after the two months is when we do the decisive tests and now by the time we give them the results, it’s much easier, however that’s not the case for patients that we get who are already admitted to the ward they come with an acute illness and then from there you find that this patient is eligible even when the parent think they are able to give you consent, occasionally we take you to the reservation. So, we have to allow them first to recover a little bit more, and when they are stable, we take them. But still we notice that’s a short time to give somebody genetic information, but those who are stable and we have prepared them for a long time like three month, two months it’s very easy so patients go through some preparation. | | | | | | | | | | | | | | | |  |
|  |  |  |  |  |  |  |  |  |  |  |  |  |  |  |  |  |  |
|  |  | | | | | | | | | | | | | | | |  |
|  | | | | | | | | | | | |  |  |  |  |  |  |
|  | Respondent: Yes, the other condition is for them to understand the context under which they’ve got the result. Some of the genetic work is a process, so you might get an early result and say look I have given you this result but wait until I do the rest of the tests. So that requires somebody to understand that there is progressive delivery of results, or divulging of the patient condition. So that is also a very very, key thing, because just getting one genetic condition is not enough for you to say that this patient has this and so this is how the prognosis will be  Interviewer: So, you give the results when you have adequate interpretation of what it means to the individual  Respondent: Yes exactly, thank you  Interviewer: And how about the family, how do you prepare the family, I know these are children their consent is usually from the parent who brings them, how do you prepare the parents, the siblings, coz it is a genetic condition and probably the wider family  Respondent: In research setting before you start any protocol, you will have gone through that so it is much easier to have a guideline that 1. Give people time of a minimum of 60 days, which is two months, that is what we do here, and then during that time you prepare them peacefully and also see if they are interested in information and then you quickly gauge how much information you are able to give and that also applies to the child directly if the child is able to understand and then you use a specific, either a cartoon, like we’ve done it before or use a specific method or even use teachers, there are situations where we’ve used teachers especially after knowing that a child responds very well to the teacher, we’ve brought in a teacher, the teachers signed in a form and said we want to be very confidential on the child’s part but am very interested in the child’s health. So that type of preparation does happen but I should quickly add, preparation for patients who come when they are acutely sick, it is very difficult, why, normally the grand mother is the one who brings the child or if it is a parent it is one parent, usually the mother. So if you are talking about a genetic condition and you need the whole family to know, it is a bit difficult, that is why it is much easier to do it in a research setting on a benign condition than an acute condition, at least that is my experience  Interviewer: Are there aspects like where you have to go to the community to do engagement for the community to be aware that there is this type of work and it could yield this?  Respondent: Yes, we’ve done that on two major occasions, 1. When we needed to know the burden of disease in the community, we first told them what the protocol is all about and then how the community would contribute. This is particularly when we wanted to take samples that are community based to compare with samples which are the hospital-based surveys. So under that circumstance we’ve had to go through the gate keepers who are mainly the leaders and then the elders and then we eventually reach the homesteads. Now once we’ve reached that level, we have also given time after talking to the leaders and elders, for some information to spread first, then we go to the communities and meet at an agreed venue and then the community asks questions. And at that point, the community will be stimulated to understand what they have. We’ve done this and even collected samples  Interviewer: Did you also return results to these communities?  Respondent: yes for instance we typed over 2000 community samples for 3 conditions, sickle cell anemia, beta thalassemia and glucose-6-phosphate dehydrogenase and those that we return, we return all the results as individual results and those that we found positive, we’ve been able to refer them to specialized centers and I think maybe up to 80% of those who were positive a being followed up by the hospital clinic | | | | | | | | | | | | | | | |  |
|  |  |  |  |  |  |  |  |  |  |  |  |  |  |  |  |  |  |
|  |  |  |  |  |  |  |  |  |  |  |  |  |  |  |  |  |  |
|  |  |  |  |  |  |  |  |  |  |  |  |  |  |  |  |  |  |
|  |  |  |  |  |  |  |  |  |  |  |  |  |  |  |  |  |  |
|  |  | | | | | | | | | | | | | | | |  |
|  | | | | | | | | | | | |  |  |  |  |  |  |
|  | Interviewer: So, you would document it in your consent that during the conduct of our test we discovered anything significant  Respondent: No that I have to tell you that was not there in the consent, we just set out, basing on the literature by the way, this is what we are going to do, so when we ran a test is when we found that. However, for those 6, because of the interest we decided that since they were recruited to reconsent and say yours was completely different and in case we do another test of interest to this particular group, we are going to call upon you, so we have achieved their bioinformation | | | | | | | | | | | | | | | |  |
|  |  | | | | | | | | | | | | | | | |  |
|  | | | | | | | | | | | | | | | | | |
| Reports\\Coding Summary By Code Report | | | | | | | | | | Page 95 of 109 | | | | | | | |
|  | | | | | | | | | | | | | | | | | |
|  | | | **Aggregate** |  | **Classification** |  | **Coverage** |  | **Number Of Coding References** | |  | **Reference Number** |  | **Coded By Initials** |  | **Modified On** |  |
|  | | | **Files\\Male\\R-025 male..** | | | | | | | | | | | | | |  |
|  |  |  | No |  |  |  |  |  |  | |  | | | | | |  |
|  | | |  |  |  |  |  |  |  | |  | | | | | | |
|  | | | | | | | | | | | |  |  |  |  |  |  |
|  | Respondent: For the community, there are two packs, usually for the community, we have a community advisory board, the PI call the CABs and let them know that there is something we want to discuss with the community. And we brainstorm and we see who we should call from the community because …so we then decide to coordinate with opinion leaders, and then some people who have ever recovered from this and formulate populations. That is the procedure we follow. | | | | | | | | | | | | | | | |  |
|  |  | | | | | | | | | | | | | | | |  |
|  | **Nodes\\Return of results\\Specilaized genetic counsellor** | | | | | | | | | | | | | | | |  |
|  | | **Document** | | | | | | | | | | | | | | |  |
|  | | | **Files\\Female\\R-003.female** | | | | | | | | | | | | | |  |
|  |  |  | No |  |  |  |  |  |  | |  | | | | | |  |
|  | | |  |  |  |  |  |  |  | |  | | | | | | |
|  | | | | | | | | | | | |  |  |  |  |  |  |
|  | RESPONDENT: no, are they even there in Uganda we just have generalized social workers and counsellors but we have our community psychologists those are ones we use but it is not that they are really specialized in genomics but it something good but also one of the things I have realized is that the nurses even those ones who are diploma holders there are certain level of understanding when it comes to genetic conditions, they don’t know the molecular bit of it but given the kind of their work they know that ok if this man probably has this problem it can be either a man, woman or a couple that they can easily pass it on so they easily go to give that kind of counsel not that they specialize but given their biology or nature of their work sort of provide counselling but am not very sure whether in the country we have counsellors who are specialized in genetics | | | | | | | | | | | | | | | |  |
|  |  |  |  |  |  |  |  |  |  |  |  |  |  |  |  |  |  |
|  |  | | | | | | | | | | | | | | | |  |
|  | | | **Files\\Female\\R-021 female** | | | | | | | | | | | | | |  |
|  |  |  | No |  |  |  |  |  |  | |  | | | | | |  |
|  | | |  |  |  |  |  |  |  | |  | | | | | | |
|  | | | | | | | | | | | |  |  |  |  |  |  |
|  | Respondent: No, I think we have three consent forms, so they are all explained separately, there is a consent to participate, consent to store the samples for studies and you name the studies, then there is a consent for genetic testing and you tell them how, so am thinking the person doing the consent giving will explain these consents to them  Interviewer: DO you think they are competent enough to explain? They have a genetics background?  Respondent: Yes, it is not so, so in depth, at least we provide the information in relation to the objectives so they are able to explain it | | | | | | | | | | | | | | | |  |
|  |  | | | | | | | | | | | | | | | |  |
|  | | | | | | | | | | | | | | | | | |
|  | | | | | | | | | | | | | | | | | |
| Reports\\Coding Summary By Code Report | | | | | | | | | | Page 96 of 109 | | | | | | | |
|  | | | | | | | | | | | | | | | | | |
|  | | | **Aggregate** |  | **Classification** |  | **Coverage** |  | **Number Of Coding References** | |  | **Reference Number** |  | **Coded By Initials** |  | **Modified On** |  |
|  | | | | | | | | | | | | | | | | | |
|  | | | | | | | | | | | |  |  |  |  |  |  |
|  | Interviewer: What of some of the implications, like social, emotional, and some other aspects coz like genetics it is a bit different, since it can inform, you can just include one person but get information about a person’s family, their community, like the wider community, so could it be something?  Respondent: That has no impact, coz the way we design our consent form we tell them that there is going to be no feed back on your genetics and it has no impact on your clinical management, so those results will not change the way you are treated or cared for, so we don’t really need to provide it, because even if we provided it what are we going to do for the person, we should continue the way the general public is, we are all different, we go to the hospital and we are treated and it is not based on any genetics, its based on the action of the drug and the micro organism  Interviewer: What of the bigger science community that does this kind of work, do you think it is necessary to have a genetic counselor?  Respondent: So, what would he or she be counseling? The investigators? Because then investigators understand it scientifically and they know why they choose a certain gene, so they know the explanations they don’t need to be counseled. The counseling might be for the participants but I also don’t think, because you tell them, this has no impact on your treatment and it won’t change the way you are managed, so the nurses should be able to do it | | | | | | | | | | | | | | | |  |
|  |  |  |  |  |  |  |  |  |  |  |  |  |  |  |  |  |  |
|  |  | | | | | | | | | | | | | | | |  |
|  | | | **Files\\Female\\R026 female** | | | | | | | | | | | | | |  |
|  |  |  | No |  |  |  |  |  |  | |  | | | | | |  |
|  | | |  |  |  |  |  |  |  | |  | | | | | | |
|  | | | | | | | | | | | |  |  |  |  |  |  |
|  | INTERVIEWER: So, we talked about the conditions under which results ought to be returned, so the idea is when the results may have far reaching consequences. You give me these results and I don’t know how to deal with the new reality that you present in light of those results. In light of that do you think there is need for a specialized genetic councilor on your research team while doing GGR?  RESPONDENT: You know GGR studies can be broad, I can have my question but I can look at other issues, lets say my question was TB but someone else comes and says do you know what I want to look at this to look at whether Ugandans are prone to colon cancer, now I start looking at genes for colon cancer that I have again seen else where to see whether they are in Ugandans, and then I find that 20% of your people have a gene for colon cancer, that is slightly different and it would be handled differently, but that one unless they were done with approval that this is completely anonymous and you wont link it to a community, but if it is linked and you need to bring those results back that a completely different situation, and that is where we would discuss even before we do that study, what do we do if we find that these people have a gene for colon cancer, and these don’t? Do we then advise them to go to the doctor to check and see? So that is why genetic study is always depending on the purpose and that is the complexity of it | | | | | | | | | | | | | | | |  |
|  |  |  |  |  |  |  |  |  |  |  |  |  |  |  |  |  |  |
|  |  | | | | | | | | | | | | | | | |  |
|  | | | | | | | | | | | | | | | | | |
|  | | | | | | | | | | | | | | | | | |
|  | | | | | | | | | | | | | | | | | |
|  | | | | | | | | | | | | | | | | | |
| Reports\\Coding Summary By Code Report | | | | | | | | | | Page 97 of 109 | | | | | | | |
|  | | | | | | | | | | | | | | | | | |
|  | | | **Aggregate** |  | **Classification** |  | **Coverage** |  | **Number Of Coding References** | |  | **Reference Number** |  | **Coded By Initials** |  | **Modified On** |  |
|  | | | | | | | | | | | | | | | | | |
|  | | | | | | | | | | | |  |  |  |  |  |  |
|  | INTERVIEWER: or is there a need of a genetic councilor who is conversant with the issues to do with that such that this person is prepared for either the good or the bad news just like the advent of HIV/AIDs, we had specialized councilors who understood how to deal with disclosure of all that  RESPONDENT: I’ll give you an example one woman died of breast cancer, she was in her early 60’s. First forward, she had two daughters, one daughter was in another country, she went for genetic testing for breast cancer and they found that she had the gene for breast cancer, so she had double mastectomy. Now she has three daughters and I know she must have told her daughters to test for breast cancer because her mother died in her 60’s of breast cancer, but for her she did the test when she already has 6 children so she had the mastectomy but its impacting on her children. They are not yet married but what if they find that they have the gene for breast cancer, what do you tell them? Would you tell them to have mastectomy at 60? Coz for her now she’s in her late 60’s and she’s okay, no breasts but she’s fine. Then the other example, a person goes for paternity testing, a very good example, and you find that the child does not belong to this person. For example, one family got green cards, and daughter one failed the paternity test and the mother and daughter had to stay and others went, marriage broke up. Then on the other hand, I raise the child then for some reason we do paternity testing and the father says the child is not mine, I say impossible, let me also go, I actually find that the child is not mine either, the child is neither ours, this child was changed in the maternity ward 30 years ago. Now what does that mean? The child is now 25 years or 30, it means nothing. So, is it so important? So those are the situations whereas if my child is two years and I say we are not sure if this child is ours that is ok, there is still time to correct it, because we can go back to where we delivered from where there are records, which other child was born at that time and then try to trace that child, but if 25years, it is difficult. But there are certain situations where you are not supposed to conceal it. My grand father and senga both died of cancers, colon cancers, we want to know whether we have the gene for colon cancer, it is necessarily a yes or no, and you cant tell me you don’t have it, but then what you are asking are the incidental findings, those are the complex ones and for the incidental findings you have three options before deciding: Will it impact on a persons life, what is the probability that they will get the disease? Is it preventable? Will they get the disease in the early years before 60, so that they should learn about it or they will get the problem at 80year? If you tell me you are at risk of getting ischemic heart disease in my 80’s, you don’t have to tell me its okay. But if you tell me that by the way you are prone at getting ischemic heart disease in your 40’s then it is good to know because then you can have regular check ups and family can know. And then the severity of the problem, you are at risk if getting viti line, well so far it will not kill you but you will look not nice, while tell people a 20-year-old girl or 15-year-old that you have the probability of getting viti line, and they ask what is the probability, oh 30 40% probability. Why don’t you leave the person alone?  INTERVIEWER: So, do you have any specialized genetic councilor on your research team?  RESPONDENT: No. We train them, we read about it, we get information. Actually, we did a lot of reading, looking at the risks and so, then we train our people. What is this about genetic counseling that makes it so special? SO, we came up with some power point, we discussed it and then we taught people why genetic counselling has some risk, people need to understand what is the risk, knowing that you have the gene BRS is at greater risk, so that they know what are we talking about | | | | | | | | | | | | | | | |  |
|  |  |  |  |  |  |  |  |  |  |  |  |  |  |  |  |  |  |
|  |  |  |  |  |  |  |  |  |  |  |  |  |  |  |  |  |  |
|  |  |  |  |  |  |  |  |  |  |  |  |  |  |  |  |  |  |
|  |  |  |  |  |  |  |  |  |  |  |  |  |  |  |  |  |  |
|  |  | | | | | | | | | | | | | | | |  |
|  | | | | | | | | | | | |  |  |  |  |  |  |
|  | INTERVIEWER: So, meaning that since you do the training you believe it is important to do genetic counselling  RESPONDENT: Yes, but it is important for the counsellors to understand, what its all about, so that they know how to approach individual people | | | | | | | | | | | | | | | |  |
|  |  | | | | | | | | | | | | | | | |  |
|  | | | **Files\\Female\\R027.female** | | | | | | | | | | | | | |  |
|  |  |  | No |  |  |  |  |  |  | |  | | | | | |  |
|  | | |  |  |  |  |  |  |  | |  | | | | | | |
|  | | | | | | | | | | | |  |  |  |  |  |  |
|  | INTERVIEWER: Do you have any specialized genetic counsellor on your research team?  RESPONDENT: No.  INTERVIEWER: Why not?  RESPONDENT: Well, I think because our research has not been thought to be so sensitive like the ones, we have been talking about are not so sensitive. The information is really about enzymes of drugs, it is not something that we considered sensitive that need to be counselled about. | | | | | | | | | | | | | | | |  |
|  |  | | | | | | | | | | | | | | | |  |
|  | | | | | | | | | | | | | | | | | |
|  | | | | | | | | | | | | | | | | | |
| Reports\\Coding Summary By Code Report | | | | | | | | | | Page 98 of 109 | | | | | | | |
|  | | | | | | | | | | | | | | | | | |
|  | | | **Aggregate** |  | **Classification** |  | **Coverage** |  | **Number Of Coding References** | |  | **Reference Number** |  | **Coded By Initials** |  | **Modified On** |  |
|  | | | **Files\\Female\\R028.female** | | | | | | | | | | | | | |  |
|  |  |  | No |  |  |  |  |  |  | |  | | | | | |  |
|  | | |  |  |  |  |  |  |  | |  | | | | | | |
|  | | | | | | | | | | | |  |  |  |  |  |  |
|  | INTERVIEWER: But if you are mandated to return results to individuals, what are those circumstances that can be in place? Would you advise to have a genetic counsellor or?  RESPONDENT: But why? Anyway, me I think it is because of the kinds of genes am dealing with, they are not disease genes, these are immune system genes, everybody has them but what they have is different. It not like for example sickle cell disease, where there is clearly an abnormal variant. These immune system genes am studying are good for certain things and bad for others, they are not outright bad. So, it is like blood groups, people will say this group does this and this, but in the end, it doesn’t matter what blood group you have, other that the advantage that one is easier to be transfused. So far, I think that is not why I have encountered those situations. Because everyone has them  INTERVIEWER: There are no special cases?  RESPONDENT: Yeah in fact those who are special die at birth or at the early age when they are children. Otherwise I don’t want to imagine what other genetic researchers are doing, am sure if for example, you were doing a genetic study and then you realize some rare variant associated with some bad outcome especially in future, and it is known, some people I know people have been asking this, but any bad news requires a counselor, whether genetics or not | | | | | | | | | | | | | | | |  |
|  |  |  |  |  |  |  |  |  |  |  |  |  |  |  |  |  |  |
|  |  | | | | | | | | | | | | | | | |  |
|  | | | | | | | | | | | |  |  |  |  |  |  |
|  | INTERVIEWER: So, do you have any specialized genetic councilor on your research team?  RESPONDENT: No  INTERVIEWER: Why not?  RESPONDENT: because I was not giving back any result, so why, why would I employ people am not going to use? I don’t have that money | | | | | | | | | | | | | | | |  |
|  |  | | | | | | | | | | | | | | | |  |
|  | | | **Files\\Female\\R029 female** | | | | | | | | | | | | | |  |
|  |  |  | No |  |  |  |  |  |  | |  | | | | | |  |
|  | | |  |  |  |  |  |  |  | |  | | | | | | |
|  | | | | | | | | | | | |  |  |  |  |  |  |
|  | INTERVIEWER: you had not included it in the consent form but you feel this person if not communicated to, he/she may die in the next year, or something like that or something bad might happen because it is something that might affect her life. These are risk factors.  RESPONDENT: I again being humane, I think I would report such a finding but the way I handle such also matters. I think you need a counsellor to explain if you are not a counselor yourself. Maybe you need a genetic counsellor or a counsellor who can break it down to the patient. Again it maybe, an advantage to the patient that they have found such results to prevent occurrence of such diseases.  INTERVIEWER: In your current study do you have a genetic counsellor?  RESPONDENT: No  INTERVIEWER: Why | | | | | | | | | | | | | | | |  |
|  |  |  |  |  |  |  |  |  |  |  |  |  |  |  |  |  |  |
|  |  | | | | | | | | | | | | | | | |  |
|  | | | | | | | | | | | |  |  |  |  |  |  |
|  | RESPONDENT:This is something that I learnt about genetic counsellors along the way, but during my study I have not come across one or even round the mulago hill. do they even exist, but along the way as you read you realize there are genetic counsellors. and may be if they are not there like in our community here of research, may be you can use a normal counsellor as long as they can fully explain and make the patient come to terms or understand what they are talking about. | | | | | | | | | | | | | | | |  |
|  |  | | | | | | | | | | | | | | | |  |
|  | | | **Files\\Female\\R030.female** | | | | | | | | | | | | | |  |
|  |  |  | No |  |  |  |  |  |  | |  | | | | | |  |
|  | | |  |  |  |  |  |  |  | |  | | | | | | |
|  | | | | | | | | | | | |  |  |  |  |  |  |
|  | INTERVIEWER: Do you have like procedures in place that your research team is supposed to follow in case they are giving back those results?  RESPONDENT: We do have procedures SOPs on how to give results to clients whether they are genetics or HIV because we have specific counsellors that are designated for specific results so that they know how to live with each study. | | | | | | | | | | | | | | | |  |
|  |  | | | | | | | | | | | | | | | |  |
|  | | | | | | | | | | | | | | | | | |
| Reports\\Coding Summary By Code Report | | | | | | | | | | Page 99 of 109 | | | | | | | |
|  | | | | | | | | | | | | | | | | | |
|  | | | **Aggregate** |  | **Classification** |  | **Coverage** |  | **Number Of Coding References** | |  | **Reference Number** |  | **Coded By Initials** |  | **Modified On** |  |
|  | | | | | | | | | | | | | | | | | |
|  | | | | | | | | | | | |  |  |  |  |  |  |
|  | INTERVIEWER: Do you have any specialized genetic counsellor on your research team?  RESPONDENT: I would say they are specialized through practice not through training. So, they have gone through the protocol and the standard procedures for counselling but they have not general training on genetic counselling. And our Botswana team also developed like a booklet, so that also helped because they engaged the community so that also helped inform how counsellors because we take them through how we should be speaking to the participants.  INTERVIEWER: I think the one I saw was how to consent in different languages.  RESPONDENT: So, you see because you cannot consent because you don’t know how to counsel.  INTERVIEWER: Do you think that is sufficient or you think you need to train first?  RESPONDENT: I think we need training just like the way you see training in HIV counselling I think we also need training in genetic counselling, short courses. It is very important and some of those are some of the courses we think we could have added in, in capturing like capacity building but when you have more scientists than social, some of those things tend to come up but still it is important. | | | | | | | | | | | | | | | |  |
|  |  |  |  |  |  |  |  |  |  |  |  |  |  |  |  |  |  |
|  |  | | | | | | | | | | | | | | | |  |
|  | | | **Files\\Male\\R-001 male** | | | | | | | | | | | | | |  |
|  |  |  | No |  |  |  |  |  |  | |  | | | | | |  |
|  | | |  |  |  |  |  |  |  | |  | | | | | | |
|  | | | | | | | | | | | |  |  |  |  |  |  |
|  | OF course AS YOU SAID, THIS IS A NEW FIELD, THEY ARE VERY FEW PEOPLE WHO CAN UNDERSTAND WHAT IT IS, I WOULD THINK THAT FIELDS DEVELOP, WE HAVE HIV Counsellors which was not there several years ago, we just had general counselors. so i was talking to a young lady, she has finished a degree, masters in counselling and she told me that there is industrial psychology and all these different types of psychologies, so fields develop, at the moment there aren't so many people in our setting, its relying mainly on people who are giving consent to give a little more training to deliver this. i have seen this in people dealing with paternity tests . | | | | | | | | | | | | | | | |  |
|  |  | | | | | | | | | | | | | | | |  |
|  | | | | | | | | | | | |  |  |  |  |  |  |
|  | I think so, if they are necessary in HIV/AIDS research, then they are necessary in this kind of research. we saw that in the beginning, HIV people were given results and they would just go hang themselves because they were probably not well prepared. so i think that it is a field that needs alot of input because it is developing much fast, and we need to develop the capacity of people to offer this type of service. | | | | | | | | | | | | | | | |  |
|  |  | | | | | | | | | | | | | | | |  |
|  | | | **Files\\Male\\R-002. male** | | | | | | | | | | | | | |  |
|  |  |  | No |  |  |  |  |  |  | |  | | | | | |  |
|  | | |  |  |  |  |  |  |  | |  | | | | | | |
|  | | | | | | | | | | | |  |  |  |  |  |  |
|  |  | | | | | | | | | | | | | | | |  |
|  |  | | | | | | | | | | | | | | | |  |
|  | | | | | | | | | | | |  |  |  |  |  |  |
|  | Respondent: no, are just beginning and still very young | | | | | | | | | | | | | | | |  |
|  |  | | | | | | | | | | | | | | | |  |
|  | | | | | | | | | | | |  |  |  |  |  |  |
|  | Interviewer: so that’s the reason you don’t have  Respondent: that’s the reason we don’t have  Interviewer: how come you have researchers if it is very young  Respondent: the researchers may not have been so much involved in the genetics studies and stuff so the genetics study area I think it is just beginning so we still have some long way but we still need to have some systems in place already as we take on | | | | | | | | | | | | | | | |  |
|  |  | | | | | | | | | | | | | | | |  |
|  | | | **Files\\Male\\R-004.male** | | | | | | | | | | | | | |  |
|  |  |  | No |  |  |  |  |  |  | |  | | | | | |  |
|  | | |  |  |  |  |  |  |  | |  | | | | | | |
|  | | | | | | | | | | | |  |  |  |  |  |  |
|  | Respondent: I don’t have one, I feel we can handle along with the research team, the counselling is really done by the nurses and the research assistants so I feel the genetic counselling can be filled under the general counselling | | | | | | | | | | | | | | | |  |
|  |  | | | | | | | | | | | | | | | |  |
| Reports\\Coding Summary By Code Report | | | | | | | | | | Page 100 of 109 | | | | | | | |
|  | | | | | | | | | | | | | | | | | |
|  | | | **Aggregate** |  | **Classification** |  | **Coverage** |  | **Number Of Coding References** | |  | **Reference Number** |  | **Coded By Initials** |  | **Modified On** |  |
|  | | | | | | | | | | | | | | | | | |
|  | | | | | | | | | | | |  |  |  |  |  |  |
|  | Respondent: there is a consent form that has been designed before the consent for the study is explained to the participants, say the adults or parents of the minors that genetic aspect is talked about for example that there is, individuals are different because of the genes and because of this, this could occur after a transfusion and that’s why we are doing this research so there is that basic information and still I have not had community involvement to give them the information it says that it is confidential to the individual and the family not taking it to the community level | | | | | | | | | | | | | | | |  |
|  |  | | | | | | | | | | | | | | | |  |
|  | | | **Files\\Male\\R-005.male** | | | | | | | | | | | | | |  |
|  |  |  | No |  |  |  |  |  |  | |  | | | | | |  |
|  | | |  |  |  |  |  |  |  | |  | | | | | | |
|  | | | | | | | | | | | |  |  |  |  |  |  |
|  | Respondent: My kind of work did not involve those, so I did not have because I was a small person on the big project | | | | | | | | | | | | | | | |  |
|  |  | | | | | | | | | | | | | | | |  |
|  | | | | | | | | | | | |  |  |  |  |  |  |
|  | Respondent: Probably they’ve been having, and those are mostly in the clinical part of it not these guys doing the basic sciences in the lab, those who interact with the patients.  Interviewer: So you don’t know the reason why they had them or not?  Respondent: I did not know because they designed that part.  Interviewer: But do you think it is important to have genetic counselors on the team?  Respondent: Very very important, because of the possible outcomes, we should have genetic counselors, whenever you talk about genetics first of all there is a lot of misunderstandings, so presence of genetic counselors would help us sort out those possible misunderstandings | | | | | | | | | | | | | | | |  |
|  |  |  |  |  |  |  |  |  |  |  |  |  |  |  |  |  |  |
|  |  | | | | | | | | | | | | | | | |  |
|  | | | **Files\\Male\\R-006. male** | | | | | | | | | | | | | |  |
|  |  |  | No |  |  |  |  |  |  | |  | | | | | |  |
|  | | |  |  |  |  |  |  |  | |  | | | | | | |
|  | | | | | | | | | | | |  |  |  |  |  |  |
|  | No, we do not. | | | | | | | | | | | | | | | |  |
|  |  | | | | | | | | | | | | | | | |  |
|  | | | | | | | | | | | |  |  |  |  |  |  |
|  | So why don't you have them?  RESPONDENT:  Like i have said before we haven't been involved in research where we were required or where we needed to give this information back to the participants. that’s the main reason really. | | | | | | | | | | | | | | | |  |
|  |  | | | | | | | | | | | | | | | |  |
|  | | | | | | | | | | | |  |  |  |  |  |  |
|  | RESPONDENT:  so that is true, on the other hand, to explain to the participant the concept of what you are going to do, you don’t need to necessarily be a counselor, you need to be someone who understands the concept. We haven't really needed it, because the counselling bit to my understanding is a, this person would have to be good at giving back news that came from the analysis at the end of the day. you prepare them for the news when the testing is ready. And then how to give news back, i think that’s what would require special counseling skills to make sure that this person doesn’t handle the news badly. but of course, it is more like any other disease, if someone has TB, you don't need a counselor to tell someone you have TB. A trained doctor, a trained medical person would actually tell a person you have TB, but these persons are trained to say, give the patient comfort, its TB, but its not the end of the world, its treatable .  INTERVIEWER:  there special diseases that have issues where specialized individuals like in HIV, because we had the doctors and nurses, but they didn’t seem to be adequate.  RESPONDENT:  i essentially agree, that’s where i started from. Right now, we haven’t needed to do that because we haven't needed to give back these results to participants. if that was necessary and we got to a point where did research, and did activities where we needed to give back results to participants, we need the counsellors, that’s what i think.  INTERVIEWER: | | | | | | | | | | | | | | | |  |
|  |  |  |  |  |  |  |  |  |  |  |  |  |  |  |  |  |  |
|  |  |  |  |  |  |  |  |  |  |  |  |  |  |  |  |  |  |
|  |  | | | | | | | | | | | | | | | |  |
| Reports\\Coding Summary By Code Report | | | | | | | | | | Page 101 of 109 | | | | | | | |
|  | | | | | | | | | | | | | | | | | |
|  | | | **Aggregate** |  | **Classification** |  | **Coverage** |  | **Number Of Coding References** | |  | **Reference Number** |  | **Coded By Initials** |  | **Modified On** |  |
|  | | | | | | | | | | | | | | | | | |
|  | | | | | | | | | | | |  |  |  |  |  |  |
|  | Do you really get in touch with the participants directly, or after the clinicians take on the sample then for you, you only deal with the samples.  RESPONDENT:  so in the Lab, we largely deal with the samples, sometimes we have to do the sample collection ourselves, but we are not involved in any counselling of participants or the giving back of results to them, so we give the results to the clinicians.  INTERVIEWR:  But its policy if it’s a project, you have teams, you are the one who does everything. so each team has its roles, that’s why we are asking, there these counselors who have the role. There individuals with specific roles, the clinicians have their role, but it seems for now the clinicians are also taking on that role of communicating.  RESPONDENT:  and that’s is not for everything, like you said the national guidelines say that if its an HIV Person, you need the counselor to give back the results those are clear, they are defined like that, its the same thing here, if they are HIV results, they are not given by anybody, and are not given by the doctor, but if its any other illness like malaria, the Doctor will tell the patient, you have TB.  INTERVIEWER:  But for GGR, do you think there should be a specific counsellor not the general counselor?  RESPONDENT:  i absolutely think so, i think for Genetics, and Genomics, you would definitely need a special counselor. i think you would need someone with a genetics background that would be able to understand complicated questions if they arose from the client they are trying to counsel, it could be basics of genetics. | | | | | | | | | | | | | | | |  |
|  |  |  |  |  |  |  |  |  |  |  |  |  |  |  |  |  |  |
|  |  |  |  |  |  |  |  |  |  |  |  |  |  |  |  |  |  |
|  |  | | | | | | | | | | | | | | | |  |
|  | | | **Files\\Male\\R-007 male** | | | | | | | | | | | | | |  |
|  |  |  | No |  |  |  |  |  |  | |  | | | | | |  |
|  | | |  |  |  |  |  |  |  | |  | | | | | | |
|  | | | | | | | | | | | |  |  |  |  |  |  |
|  | RESPONDENT: Aha no, as we speak right now, NO, I’ve not seen any genetic counselors, it’s not in this institution.  INTERVIEWER: why are they not, you used not to pay them?  RESPONDENT: Well so right now like I said before, the plan really isn’t to have, like I said we want to understand that in the near future but none of that really has been done so much. And yet, ahaa there isn’t a plan to return to the individuals directly to say okay this is what you have excelled in terms of genome. And so there wasn’t a need as yet but there’s going to be a need as time goes on. Aha but as we speak right now, no body is doing that kind of thing, so I think it will be in the future that we will need genetic counselors. But at a moment, we, every time we get such specimens they are not even run here anyway, they are run out of here but then we insist that all that is de-identified information that we cannot trace back to the individual. So at the moment it cannot be traced back to the individual anyway, so you wouldn’t counsel in any case. | | | | | | | | | | | | | | | |  |
|  |  |  |  |  |  |  |  |  |  |  |  |  |  |  |  |  |  |
|  |  | | | | | | | | | | | | | | | |  |
|  | | | **Files\\Male\\R-008.male** | | | | | | | | | | | | | |  |
|  |  |  | No |  |  |  |  |  |  | |  | | | | | |  |
|  | | |  |  |  |  |  |  |  | |  | | | | | | |
|  | | | | | | | | | | | |  |  |  |  |  |  |
|  | RESPONDENT: no, how many are they in Uganda?  INTERVIEWER: you don’t know of any?  RESPONDENT: if you ever find them  INTERVIEWER: but do you think it is necessary to have them?  RESPONDENT: very much so, personally that is one thing I have also known from a similar course like that so I didn’t have time so I failed there  INTERVIEWER: so, it was on line, do you know of any? People have done such courses  RESPONDENT: I have not seen but there is a school in South Africa that has started a course at the university so this is the one I have known for, it was there it is called genetic counselling it knew it from 29 July though it is necessary  INTERVIEWER: why is it so necessary?  RESPONDENT: like for you in genomics so much but since you’re a researcher you’re not in a position to interface with patients in a clinic, you just picked your samples you left but a genetic counsellor is always there is always interfacing them so as I easily find the things there you are never going to interface those people however as you’re cross checking the study you picked samples you never went back but a genetic counsellor will always be there when you need them so they can keep so it is very important to have a genetic counsellor | | | | | | | | | | | | | | | |  |
|  |  |  |  |  |  |  |  |  |  |  |  |  |  |  |  |  |  |
|  |  | | | | | | | | | | | | | | | |  |
|  | | | | | | | | | | | | | | | | | |
| Reports\\Coding Summary By Code Report | | | | | | | | | | Page 102 of 109 | | | | | | | |
|  | | | | | | | | | | | | | | | | | |
|  | | | **Aggregate** |  | **Classification** |  | **Coverage** |  | **Number Of Coding References** | |  | **Reference Number** |  | **Coded By Initials** |  | **Modified On** |  |
|  | | | **Files\\Male\\R-009.male** | | | | | | | | | | | | | |  |
|  |  |  | No |  |  |  |  |  |  | |  | | | | | |  |
|  | | |  |  |  |  |  |  |  | |  | | | | | | |
|  | | | | | | | | | | | |  |  |  |  |  |  |
|  | RESPONDENT: probably am not the one on the interface of participants am more in the lab but as a team, I have been invited to train the counselors on genetics so you can imagine the counselor has studied counselling but they don’t know genetics so I have been invited to equip them with genetic knowledge and probably blend that genetic knowledge with their counselling experience that is what we have done with the institution | | | | | | | | | | | | | | | |  |
|  |  | | | | | | | | | | | | | | | |  |
|  | | | **Files\\Male\\R-010. male** | | | | | | | | | | | | | |  |
|  |  |  | No |  |  |  |  |  |  | |  | | | | | |  |
|  | | |  |  |  |  |  |  |  | |  | | | | | | |
|  | | | | | | | | | | | |  |  |  |  |  |  |
|  | RESPONDENT: of course you have to go back looking through the consent. If you find something that is really important, you back look into the consent if the patient consented you have to invite the patient back. Literally in a normal context this is supposed to be a genetic counsellor to give this information and genetic counselling is not just psychosocial support but it’s about talking about the mechanisms different disease or things come about so that the patient can understand the options for testing, treatment before you even begin telling them the problem has been found. Once that has been found, you need health support, psychosocial and reveal whatever you found. So, psychosocial support is a very small section of genetic counselling. Ideally this should be a genetic counsellor to offer this information. I have a PhD student who actually asked me to offer any loose out spark to the patients. I have done some genetic counselling training for nurses. By the way, in the USA genetic counselling is a super specialty and it’s a place for nurses especially. Why do we use nurses because they have already medical background and they have the legal right to interact with patients so you just can’t pick anybody and they turn into a genetic counsellor because there are things to do with things that govern medical practice that also govern this practice of genetic counselling. Recently we trained nurses in the Pediatric Centre of Excellence because that is our recruitment site for the CafGEN study which is looking at children who are born with HIV and how genetics is affecting disease progression both for HIV and TB. The idea was that we could find some information that we need to give back to these participants. I tried to convince the cancer institute to also take up genetic some genetic counseling because we have some cancers which are genetic and other cancers are superionic so if you have a genetic counselor, it is important that you form your…that they can begin…because as you…like I said, you get an incidental finding but is of interest to the public to the wider community so sometimes the decision to hold on to this information is very difficult even if the patient has not consented. You wonder if there should be scenarios where the IRB should take over the mandate to override and make sure this information is given to these people. | | | | | | | | | | | | | | | |  |
|  |  |  |  |  |  |  |  |  |  |  |  |  |  |  |  |  |  |
|  |  |  |  |  |  |  |  |  |  |  |  |  |  |  |  |  |  |
|  |  | | | | | | | | | | | | | | | |  |
|  | | | **Files\\Male\\R-011.male** | | | | | | | | | | | | | |  |
|  |  |  | No |  |  |  |  |  |  | |  | | | | | |  |
|  | | |  |  |  |  |  |  |  | |  | | | | | | |
|  | | | | | | | | | | | |  |  |  |  |  |  |
|  | we don't have a genetic counselor on our team because our study doesn't need one. a genetic counselor would be important for a diagnostic clinic, a patient comes in with a condition, needs to be diagnosed. so ours is more of a population study, so on the side of the clinic, they have their counselors as they are collecting the samples, as i said we are more on the data analysis side, we are in lab, and in our side of the study we don't need counselors. | | | | | | | | | | | | | | | |  |
|  |  | | | | | | | | | | | | | | | |  |
|  | | | | | | | | | | | |  |  |  |  |  |  |
|  | Yes, i would definitely think we would need one. it’s just that like i said, our study is de-identified, results could never go back to the particular individual because we just can't find it. it was part of the ethical approval process that we can’t go back to those specific individuals. If we can, then there is something flawed in our study, to make it ethical, then we just tighten the mechanism of de-identifying these individuals. we can go back to the community, maybe we need a counselor for the community, but for the individual we can't. | | | | | | | | | | | | | | | |  |
|  |  | | | | | | | | | | | | | | | |  |
|  | | | **Files\\Male\\R-012.male** | | | | | | | | | | | | | |  |
|  |  |  | No |  |  |  |  |  |  | |  | | | | | |  |
|  | | |  |  |  |  |  |  |  | |  | | | | | | |
|  | | | | | | | | | | | |  |  |  |  |  |  |
|  | RESPONDENT: at least not one that I am aware of | | | | | | | | | | | | | | | |  |
|  |  | | | | | | | | | | | | | | | |  |
| Reports\\Coding Summary By Code Report | | | | | | | | | | Page 103 of 109 | | | | | | | |
|  | | | | | | | | | | | | | | | | | |
|  | | | **Aggregate** |  | **Classification** |  | **Coverage** |  | **Number Of Coding References** | |  | **Reference Number** |  | **Coded By Initials** |  | **Modified On** |  |
|  | | | | | | | | | | | | | | | | | |
|  | | | | | | | | | | | |  |  |  |  |  |  |
|  | RESPONDENT: there is one I talked of HIV, the HIV one is multicentered actually they are looking at HIV sequences part of them are from here in Uganda like the the Rakai some from Botswana, south Africa so for those ones I think Rakai they have something coz I have attended their meetings, I have heard there are some genetic counselors but at least the one where am mostly involved the one of the IRB, I have not heard of a genetic counselor, I know the nurses have tried of some clinical medicine in genetic counseling but at least I have not heard of this person who is completely designated, I may not be aware but at least I have not heard about it, in every communications I have not heard of genetic counseling | | | | | | | | | | | | | | | |  |
|  |  | | | | | | | | | | | | | | | |  |
|  | | | | | | | | | | | |  |  |  |  |  |  |
|  | RESPONDENT: not for everyone but for RNA work I don’t think genetic counseling is really important. The RNA is going to be used for expression even RNA work is; you work backwards and find those mutations in genes but with most studies which do mutation, so there I would not recommend a genetic counselor but for any like DNA work they must need a genetic counselor if it has anything to do with DNA  INTERVIEWER: why DNA specifically?  RESPONDENT: as compared to RNA its more viable so the patient you can trace back the individuals, its unique to the individual  INTERVIEWER: so how does the counsellor come in?  RESPONDENT: so, the counsellor comes in to inform the potential results but also what exactly happens what these genes have, these mutations, to teach them | | | | | | | | | | | | | | | |  |
|  |  |  |  |  |  |  |  |  |  |  |  |  |  |  |  |  |  |
|  |  | | | | | | | | | | | | | | | |  |
|  | | | | | | | | | | | |  |  |  |  |  |  |
|  | INTERVIEWER: if you maybe take any psychosocial implications to what  RESPONDENT: exactly I think the genetic counsellor because anyone can consent coz even the nurses can the psychosocial effect of having a certain mutation that’s how a genetic counsellor comes in, | | | | | | | | | | | | | | | |  |
|  |  | | | | | | | | | | | | | | | |  |
|  | | | **Files\\Male\\R-013.male** | | | | | | | | | | | | | |  |
|  |  |  | No |  |  |  |  |  |  | |  | | | | | |  |
|  | | |  |  |  |  |  |  |  | |  | | | | | | |
|  | | | | | | | | | | | |  |  |  |  |  |  |
|  | RESPONDENT: no | | | | | | | | | | | | | | | |  |
|  |  | | | | | | | | | | | | | | | |  |
|  | | | | | | | | | | | |  |  |  |  |  |  |
|  | RESPONDENT: I think it is necessary however I don’t think we have any yet in the country, I have not heard of any genetic counsellor around but I think its timely in this raising issue of paternity and men trying to get to know whether they are fathers or not  INTERVIEWER: it is important, how about the practice what would be their role?  RESPONDENT: I think with the increase in the use of genetics in health care it is important because we are now trying to detect for many things even in ultra before a mother gives birth, genetics is also being used in point of care and treatment so I think it is important really in this kind of environment to counsel patients that you are now going for the gene therapy we are moving to gene therapy and personalized medicine we need people who can cancel our patients guide them in what could happen and what will be its implications | | | | | | | | | | | | | | | |  |
|  |  |  |  |  |  |  |  |  |  |  |  |  |  |  |  |  |  |
|  |  | | | | | | | | | | | | | | | |  |
|  | | | **Files\\Male\\R-014 male** | | | | | | | | | | | | | |  |
|  |  |  | No |  |  |  |  |  |  | |  | | | | | |  |
|  | | |  |  |  |  |  |  |  | |  | | | | | | |
|  | | | | | | | | | | | |  |  |  |  |  |  |
|  | INTERVIEWER: do you have a specialized genetic counselor on your research team?  RESPONDENT: yeah, he is around; so and so, he is a genetic counselor  INTERVIEWER: why is it important you should have him on your team?  RESPONDENT: because that’s his specialty.  INTERVIEWER: do you think moving forward, as we look at GGR is it important that we have a counselor with a specialty in genetics?  RESPONDENT: actually we need many.  INTERVIEWER: if you were to advocate for such a position in genomics research, why should we have them on board? What would they be doing as genetic counselors?  RESPONDENT: they should have the background of a medical doctor to understand the whole concept of. I don’t understand you question, you ask why we should have them? | | | | | | | | | | | | | | | |  |
|  |  |  |  |  |  |  |  |  |  |  |  |  |  |  |  |  |  |
|  |  | | | | | | | | | | | | | | | |  |
| Reports\\Coding Summary By Code Report | | | | | | | | | | Page 104 of 109 | | | | | | | |
|  | | | | | | | | | | | | | | | | | |
|  | | | **Aggregate** |  | **Classification** |  | **Coverage** |  | **Number Of Coding References** | |  | **Reference Number** |  | **Coded By Initials** |  | **Modified On** |  |
|  | | | | | | | | | | | | | | | | | |
|  | | | | | | | | | | | |  |  |  |  |  |  |
|  | INTERVIEWER: why are they important?  RESPONDENT: for us we are researchers, we are collecting data, we understand our thing, we are even publishing papers. There is still a gap in research in genetics and application in daily life. So, we are assuming this data will help train people who are applying in daily life because you cant do everything. So, you need to train a critical mass of people who are intRESPONDENT: IRB should be more stringent. Someone should be put to task to explain.  INTERVIEWER: you previously talked about communication and now you talk about consent. Do you feel there is a gap between the researcher, what they are communicating in the GGR and what actually the participant is consenting to? Do you feel they actually understand what you are saying or?  RESPONDENT: these are things which up to date they are still updating; the interpretation. Someone will be speaking a lot of Luganda, how DNA is called in Luganda, how RNA is called or a protein. Now packaging those things and telling someone that; you have TB and we shall change you treatment pattern blah blah blah and then asking them to sign that their sample will be used for any other research, now by law that patient will never win a case which ever research you do with that sample because you will be like; other research. Now what is hidden in other research; whatever you are manipulating, no one knows. That’s why IRBs have community representatives. Now whether the old woman or mother will not understand, at least an educated IRB will because you have experts of different fields at IRB; they are like can you kindly detail what is this other research? Even when the patient has gone, for them they should go ahead and pull out that component. Use whatever language in which ever composition you want and package what you are doing. Probably you can attach another appendix of a consent from IRB that decides the approval on the whole study has understood that additional part.  INTERVIEWER: so, are you pointing to the fact that probably even the IRBs don’t have enough capacity to appreciate the concerns of GG  RESPONDENT: they have because there is a good number of people who understand the language we are speaking but they should take an extra mile to squeeze these people. I have done researches where you just go to the archives, get people’s samples, they are like, they said “some samples will be used for other research” and that’s enough but did you ever tell a patient that you are going into the IRP obigene regime of their mycobacterium tuberculosis to look for extra mutations that can used in their gene X? Which research has ever done that! So, that’s the language. Even if you know what you are going to do, do you think you will go to a patient and you are like ‘madam, after getting this organism from you, we shall amplify that gene’ this is a bored woman who is probably on oxygen consenting to give you their sample. Whatever you are communicating, they are not understanding or they are not until they hear ‘later we shall give you 30,000’ then they are like ‘okay, take the sample’. Let’s communicate to the maximum to those people. For the good of research, communicate. Don’t even hide anything from IRB because IRB has a community representative. If community itself didn’t understand, that guy will be like ‘okay, now explain to us’. On the consent, for the future studies, it is hard to predict the future, I agree and IRB will give you a waiver in most cases but if know what you are going to do, you highlight it in the consent form.  interpreting that data and bringing a package to patient level. At the same time you run a parallel system; another group that generates the data and does innovative research. | | | | | | | | | | | | | | | |  |
|  |  |  |  |  |  |  |  |  |  |  |  |  |  |  |  |  |  |
|  |  |  |  |  |  |  |  |  |  |  |  |  |  |  |  |  |  |
|  |  |  |  |  |  |  |  |  |  |  |  |  |  |  |  |  |  |
|  |  |  |  |  |  |  |  |  |  |  |  |  |  |  |  |  |  |
|  |  | | | | | | | | | | | | | | | |  |
|  | | | **Files\\Male\\R-015.male** | | | | | | | | | | | | | |  |
|  |  |  | No |  |  |  |  |  |  | |  | | | | | |  |
|  | | |  |  |  |  |  |  |  | |  | | | | | | |
|  | | | | | | | | | | | |  |  |  |  |  |  |
|  | INTERVIEWER: so speaking of negative connotation do you think it is important to have a genetic counselor on the studies you have done, have you had such a person on your team?  RESPONDENT: we have not had because again there are two limitations, one we don’t have people with that knowledge and two we don’t know how to equip them with the knowledge although our colleagues that we are working with in the northern countries they had that knowledge and they had the people who do that but we try to explore various ways of how we could do that but in my research group that I know we did not acquire that person it required specific training  INTERVIEWER: why would you think it would be important for future genetic and genomics researches to have a genetic counsellor?  RESPONDENT: I think it is important because of the issue of incidental findings and we know that those incidental findings are probably an incidental finding is usually wondered you don’t want to communicate so if it is incidental and you say that the Baganda now we know that they cannot acquire HIV everybody would be running to get but usually when you say incidental and you’re going to say that it must be something bad so then there it increases the role of such people like who have and I think they require some kind of constraining psychology and that kind of stuff so regardless they are few but in my research group that I know we don’t have so we decided to hand over that responsibility to the PIs to take back the information in whichever way after all you are the ones to make decisions on whether or not to train a genetic counsellor but of course when it comes to incidentals I know it is the bad thing | | | | | | | | | | | | | | | |  |
|  |  |  |  |  |  |  |  |  |  |  |  |  |  |  |  |  |  |
|  |  |  |  |  |  |  |  |  |  |  |  |  |  |  |  |  |  |
|  |  | | | | | | | | | | | | | | | |  |
|  | | | **Files\\Male\\R-016. male** | | | | | | | | | | | | | |  |
|  |  |  | No |  |  |  |  |  |  | |  | | | | | |  |
|  | | |  |  |  |  |  |  |  | |  | | | | | | |
|  | | | | | | | | | | | |  |  |  |  |  |  |
|  | RESPONDENT: no, we don’t have | | | | | | | | | | | | | | | |  |
|  |  | | | | | | | | | | | | | | | |  |
| Reports\\Coding Summary By Code Report | | | | | | | | | | Page 105 of 109 | | | | | | | |
|  | | | | | | | | | | | | | | | | | |
|  | | | **Aggregate** |  | **Classification** |  | **Coverage** |  | **Number Of Coding References** | |  | **Reference Number** |  | **Coded By Initials** |  | **Modified On** |  |
|  | | | | | | | | | | | | | | | | | |
|  | | | | | | | | | | | |  |  |  |  |  |  |
|  | INTERVIEWER: but do you think it will be necessary?  RESPONDENT: I don’t think so because we are not, usually you want to have genetic counselling if you’re going to bring their results and you’re going to tell somebody you are like to have children with sickle cell disease so if you’re looking for a spouse make sure that spouse doesn’t have the genes of sickle cells disease you are likely to have a disease like may be whatever it is so when you’re providing that kind of information, that kind of a service you definitely need a genetic counsellor but when you’re collecting information that is purely for research purposes abstract research purposes you will never see these people again so you don’t need that kind of information | | | | | | | | | | | | | | | |  |
|  |  | | | | | | | | | | | | | | | |  |
|  | | | | | | | | | | | |  |  |  |  |  |  |
|  | RESPONDENT: no we have very robust consent form, consent form actually has a lot of things, our consenting process is about one and half hours, we tell the people that you are going to give us your saliva, we are going to look for DNA in that saliva you’re not going to see those results that information that we get from that DNA will be used in the future by scientists usually either to make medication or get treatment, if only we consider that their saliva is part of them, we tell them how we will store that better, who will access the data and for what purpose the data can only be accessed for purposes of medical research nothing else, all the PIs and everybody else who is involved in this research has signed collaborating agreements and documents which with have signed with universities and people who are funding us we know that if don’t not hold those standards we could be sued and go to prison for a long time so we take this as extremely serious we don’t expect that people will play around with this kind of information that can be used to identify individuals not just individuals but communities so we don’t want the world to come and say that there is a particular community in Uganda where the researcher has incidences of a mental illness so something like that so that is one then a stepping step of the consent where we also assess whether the person has understood the consent, the decisional capacity of that person it’s called UBAC university of California Berkley assessment of consent and capacity to consent, university of California on capacity to consent that is what we use the person must ensure capacity to consent, university of Berkley California of capacity to consent  INTERVIEWER: is that what you’re using?  RESPONDENT: that’s what we are using and the person must score a minimum of fourteen and they are given an attempt three times, people will fail those things that they have not understood the results and once they have not understood we don’t recruit them  INTERVIEWER: so, doctor that is fourteen out of?  RESPONDENT: out of twenty | | | | | | | | | | | | | | | |  |
|  |  |  |  |  |  |  |  |  |  |  |  |  |  |  |  |  |  |
|  |  |  |  |  |  |  |  |  |  |  |  |  |  |  |  |  |  |
|  |  | | | | | | | | | | | | | | | |  |
|  | | | | | | | | | | | |  |  |  |  |  |  |
|  | INTERVIEWER: that takes me back to some question we asked don’t you think there is more to genetic counselling than normal counseling?  RESPONDENT: no, this information has been collected from psychiatric hospitals so we have people who are equipped with skills to handle severe forms of physiological distress that’s the risk that we have to the participants the other risks of course is a bit remote and that the risk that somebody may steal this information whatever they use it for I don’t know it is unlikely that they can use it to blackmail the participants but they can use it for their own financial needs I hope not | | | | | | | | | | | | | | | |  |
|  |  | | | | | | | | | | | | | | | |  |
|  | | | **Files\\Male\\R-017. male** | | | | | | | | | | | | | |  |
|  |  |  | No |  |  |  |  |  |  | |  | | | | | |  |
|  | | |  |  |  |  |  |  |  | |  | | | | | | |
|  | | | | | | | | | | | |  |  |  |  |  |  |
|  | Respondent: We do not have. But what would you have defined as a specialized genetic counselor? | | | | | | | | | | | | | | | |  |
|  |  | | | | | | | | | | | | | | | |  |
|  | | | | | | | | | | | |  |  |  |  |  |  |
|  | Respondent: No, however, we are trying to attend online courses on genetic counselling, we the researchers on an individual basis, we are encouraging each other to have knowledge on genetic counseling, what is required of us | | | | | | | | | | | | | | | |  |
|  |  | | | | | | | | | | | | | | | |  |
|  | | | | | | | | | | | | | | | | | |
|  | | | | | | | | | | | | | | | | | |
| Reports\\Coding Summary By Code Report | | | | | | | | | | Page 106 of 109 | | | | | | | |
|  | | | | | | | | | | | | | | | | | |
|  | | | **Aggregate** |  | **Classification** |  | **Coverage** |  | **Number Of Coding References** | |  | **Reference Number** |  | **Coded By Initials** |  | **Modified On** |  |
|  | | | | | | | | | | | | | | | | | |
|  | | | | | | | | | | | |  |  |  |  |  |  |
|  | Respondent: The kind of information we are handling is human data, not mouse data, so it would be quite important, especially for incidental findings, how do you best relay this information especially for those settings which do not have genetic counselors, so as a researcher, its good to know even if you stop at the lab, its good to have this information for just that one time when you need to interface with the individual or sensitize the physicians or nurses who see the patients  Interviewer: So, you encourage researchers to do this genetic counseling?  Respondent: Yeah because much as you are behind that laptop analyzing looking for these loci, that are responsible for this susceptibility, you are also deep-down reading papers and seeing, where is this information, how is it applying, if a person has this mutation, which diseases are they likely to suffer from? So, it is always good in case you have such incidental findings, how best would I relay it to the affected people  Interviewer: Would you recommend a counselor for even other non-genetic studies?  Respondent: Yeah, because counselors have traditionally been around for a very long time, in other fields. Because a counselor is a person who interacts with your mind, in most cases they are interacting with your mind to change your perception on something, so even in this context of genetics, or the genetic diseases, it is highly paramount to have counseling | | | | | | | | | | | | | | | |  |
|  |  |  |  |  |  |  |  |  |  |  |  |  |  |  |  |  |  |
|  |  | | | | | | | | | | | | | | | |  |
|  | | | **Files\\Male\\R-018.male** | | | | | | | | | | | | | |  |
|  |  |  | No |  |  |  |  |  |  | |  | | | | | |  |
|  | | |  |  |  |  |  |  |  | |  | | | | | | |
|  | | | | | | | | | | | |  |  |  |  |  |  |
|  | Interviewer: So currently there is no specialized genetic counselor on the team?  Respondent: No, we don’t have, and we will not hire one, maybe we could ask a part time person to do it whenever necessary, but we do not have a professional genetic counsellor | | | | | | | | | | | | | | | |  |
|  |  | | | | | | | | | | | | | | | |  |
|  | | | | | | | | | | | |  |  |  |  |  |  |
|  |  | | | | | | | | | | | | | | | |  |
|  |  | | | | | | | | | | | | | | | |  |
|  | | | **Files\\Male\\R-019 male** | | | | | | | | | | | | | |  |
|  |  |  | No |  |  |  |  |  |  | |  | | | | | |  |
|  | | |  |  |  |  |  |  |  | |  | | | | | | |
|  | | | | | | | | | | | |  |  |  |  |  |  |
|  | Respondent: We don’t have genetic counsellors, the only thing is that when we discover genes which are relevant to the disease, not about parentage or whatever inheritance in terms of wealth, we will not, but if it is relevant to the disease or any associated diseases, we will always pass on this information to the medical personnel in the hospital and we will call back the participant, brief the medical doctor, and he is the one to handle those things, we don’t have genetic counsellors but the medical doctor has to advise you, you know if you have a problem, we cannot handle it in this hospital, Can you go and check in the other hospital? Refer, but who meets the cost? So, we give advise but we do not have a budget to meet cost for solving those problems. | | | | | | | | | | | | | | | |  |
|  |  | | | | | | | | | | | | | | | |  |
|  | | | | | | | | | | | |  |  |  |  |  |  |
|  | Respondent: Yes, you need to make these people understand, why did A marry B, you know it goes back into that, those people fall in love in a different way, but did I check that you have sickle cell for example, but after two years we have sickle cell in the family, or even worse the albino which is very visible and this causes problems also. Somebody will think it is you are the one who brought the problem, and yet the problem, both of you have the problem.  Interviewer: So, now these genetic counselors will come in to explain?  Respondent: Of course, we also come in to explain but how do you explain this in a simple language which is comprehensible to a not highly educated person in mendelian genetics, just about inheritance, a man meets a woman they have a baby, the 1st one is okay, the 2nd is okay, but the 3rd one has a problem, an observable obvious problem. In most of our cultures you blame relatives but how do you deal with this conflict in the family, and as you know in our African culture, its not just me, its me plus my extended family who decide. And my brother can say that is not the right woman for you, she the one bringing problems to our family and yet we generally have the same problem, so a genetic councilor would come, as you see now the ministry of health has made it a policy that before you get married is try to check for sickle cell. That one is now, because we accept that the prevalence of a recessive gene is high, and in some areas it’s as high as 18%. So, part of genetic counseling is test before you get married. But if you find them already married, how do you deal with it, you have to just let them understand that out of the four children, may be one will have a problem but its your child and you can’t blame this one and exclude the other one, it a combination. So that’s part of genetic counseling. So, you the researcher has to have some basic knowledge but we really need professional genetic counselors to take on these kind of things | | | | | | | | | | | | | | | |  |
|  |  |  |  |  |  |  |  |  |  |  |  |  |  |  |  |  |  |
|  |  |  |  |  |  |  |  |  |  |  |  |  |  |  |  |  |  |
|  |  | | | | | | | | | | | | | | | |  |
| Reports\\Coding Summary By Code Report | | | | | | | | | | Page 107 of 109 | | | | | | | |
|  | | | | | | | | | | | | | | | | | |
|  | | | **Aggregate** |  | **Classification** |  | **Coverage** |  | **Number Of Coding References** | |  | **Reference Number** |  | **Coded By Initials** |  | **Modified On** |  |
|  | | | **Files\\Male\\R-022. male** | | | | | | | | | | | | | |  |
|  |  |  | No |  |  |  |  |  |  | |  | | | | | |  |
|  | | |  |  |  |  |  |  |  | |  | | | | | | |
|  | | | | | | | | | | | |  |  |  |  |  |  |
|  | INTERVIEWER: do you have a specialized genetic counselor on your team?  RESPONDENT: of course, it is a whole cascade of care, depending on the study, there is a whole range of people. You are giving consent, information, for someone going to the clinic there is a counselor, and there are clinicians. So, all those people are there and they have to be part of the entire research.  INTERVIEWER: so, in that case you may not require a specialized someone who…  RESPONDENT: it really depends on the kind of study you are doing. For us we are doing medical genetics so you definitely need all sorts of counselors; the counselors that prepare participants, clinicians and the researchers themselves. So, yes, of course there has to be a counselor who understands genomics, who understands the implications of the kind of research you are doing. So you need those kinds of people. | | | | | | | | | | | | | | | |  |
|  |  |  |  |  |  |  |  |  |  |  |  |  |  |  |  |  |  |
|  |  | | | | | | | | | | | | | | | |  |
|  | | | **Files\\Male\\R-023. male** | | | | | | | | | | | | | |  |
|  |  |  | No |  |  |  |  |  |  | |  | | | | | |  |
|  | | |  |  |  |  |  |  |  | |  | | | | | | |
|  | | | | | | | | | | | |  |  |  |  |  |  |
|  | Respondent: No, and I even don’t now whether those people are here, you know, I’ve never seen one, | | | | | | | | | | | | | | | |  |
|  |  | | | | | | | | | | | | | | | |  |
|  | | | | | | | | | | | |  |  |  |  |  |  |
|  | Interviewer: But would you recommend such people on GGR teams?  Respondent: I think it depends on the question that is being asked, you know if it has a human aspect and you are looking at the whole genome not specifically one targeted thing, and especially you are probing all over, and you start finding findings that could imply one thing or another, at that stage it would be good to bring them on board. I wouldn’t say it is all, but I would say it is something you would consider on a case by case basis it really depends on the study at hand and which research they are doing | | | | | | | | | | | | | | | |  |
|  |  | | | | | | | | | | | | | | | |  |
|  | | | | | | | | | | | |  |  |  |  |  |  |
|  | Interviewer: Like in which kind of research would you recommend them and not in the other?  Respondent: No because for example all genetic studies are not equal, and for some maybe the issue at stake is not necessarily life threatening or if there are things that we already have a cure for and things like that, them for those we may not worry so much because you know whatever you find can be fixed or can be normalized or one can cope, but then there are those for which we have no solutions or cure, then for those the stakes are much higher, and so then that’s where I would say those kinds of people may be needed | | | | | | | | | | | | | | | |  |
|  |  | | | | | | | | | | | | | | | |  |
|  | | | **Files\\Male\\R-024.male** | | | | | | | | | | | | | |  |
|  |  |  | No |  |  |  |  |  |  | |  | | | | | |  |
|  | | |  |  |  |  |  |  |  | |  | | | | | | |
|  | | | | | | | | | | | |  |  |  |  |  |  |
|  | Interviewer: So, did you have any genetic counselor in your research to help the participants understand?  Respondent: No, not a geneticist as a counselor but people who have been taught to counsel in genetics, they just taught but not somebody who is a geneticist  Interviewer: What level are they?  Respondent: They are graduates, we have a social worker who is our, we call her Public engagement officer and she does public engage for science. So, she’s trained on all of that but in addition we have trained her on legal things.  Interviewer: So what level of genetic counseling training?  Respondent: That one does social, then we have got clinical officers and medical officers who when they are meeting the patients at clinical interface, they are trained but not as geneticists  Interviewer: They may not be geneticist but we can say, you are going to be a counselor in genetics, so you do 6 months course, or three months intensive training in genetic counseling  Respondent: yes, they are trained but not beyond three months, but they are trained and we train maybe sister organizations at Welcome trust where they have got better genetic tests, and actually even our lab people are trained but at desk level | | | | | | | | | | | | | | | |  |
|  |  |  |  |  |  |  |  |  |  |  |  |  |  |  |  |  |  |
|  |  | | | | | | | | | | | | | | | |  |
| Reports\\Coding Summary By Code Report | | | | | | | | | | Page 108 of 109 | | | | | | | |
|  | | | | | | | | | | | | | | | | | |
|  | | | **Aggregate** |  | **Classification** |  | **Coverage** |  | **Number Of Coding References** | |  | **Reference Number** |  | **Coded By Initials** |  | **Modified On** |  |
|  | | | **Files\\Male\\R-025 male..** | | | | | | | | | | | | | |  |
|  |  |  | No |  |  |  |  |  |  | |  | | | | | |  |
|  | | |  |  |  |  |  |  |  | |  | | | | | | |
|  | | | | | | | | | | | |  |  |  |  |  |  |
|  | Interviewer: So, did you have any specialized genetic councilor on your team?  Respondent: I don’t think we have to call them specialized genetic councilors, what we have been using are mainly social workers, psychologists, and then the doctors, the pediatricians and then ones for the terminal disease, we have just been using the palliative care team but we don’t have a specific genetic councilor | | | | | | | | | | | | | | | |  |
|  |  | | | | | | | | | | | | | | | |  |
|  | | | | | | | | | | | |  |  |  |  |  |  |
|  | Interviewer: So how have you been explaining the interpretation of the genetic findings?  Respondent: Extremely difficult, what we have been using, we also developed a video to try to explain to lay people what we think the gene are, something which is sort of understandable for them, so we use examples for example we start with a cell, we tell them what a cell is, we tell them a cell is just like a building block of the body so it is like when you are putting blocks together then that is the cell. Then inside the cell there, there are things that decide how the cell should multiply, and then if something goes wrong then these cells multiply without control. So, this is what we have been trying but we go deep down to really below the building block and say there is something inside there, we mention that we are looking for the defect in this something inside and it is very difficult. But I think in future we need to have councilors in this area | | | | | | | | | | | | | | | |  |
|  |  |  |  |  |  |  |  |  |  |  |  |  |  |  |  |  |  |
|  |  | | | | | | | | | | | | | | | |  |
|  | | | | | | | | | | | | | | | | | |
|  | | | | | | | | | | | | | | | | | |
|  | | | | | | | | | | | | | | | | | |
|  | | | | | | | | | | | | | | | | | |
|  | | | | | | | | | | | | | | | | | |
|  | | | | | | | | | | | | | | | | | |
| Reports\\Coding Summary By Code Report | | | | | | | | | | Page 109 of 109 | | | | | | | |
