# Supplementary material for: Perspectives and experiences of researchers regarding feedback of incidental genomic research findings: A qualitative study
Source: PLoS One. 2022 Aug 29;17(8):e0273657. doi: 10.1371/journal.pone.0273657 (PMC9423610; doi:10.1371/journal.pone.0273657)
Supplement: Attachment — Submitted filename: Response to Review Comments.pdf [file pone.0273657.s003.pdf]

February 6, 2022

The Editor,
PLOS ONE

Dear Sir/Madam

Ref: [PONE-D-21-28187] - [EMID:6867e1a975ba2bdf]. Perspectives and Experiences of Researchers Regarding Feedback of Incidental Genomic Research Findings: a qualitative study

Following is the point-by-point response to review comments.

1. Please ensure that your manuscript meets PLOS ONE's style requirements, including those for file naming. The PLOS ONE style templates can be found

https://journals.plos.org/plosone/s/file?id=wjVg/PLOSOne_formatting_sample_main_body.pdf and

https://journals.plos.org/plosone/s/file?id=ba62/PLOSOne_formatting_sample_title_authors_affiliations.pdf".

2. Please include additional information regarding the interview guide used in the study and ensure that you have provided sufficient details that others could replicate the analyses. For instance, if you developed an interview guide as part of this study and it is not under a copyright more restrictive than CC-BY, please include a copy, in both the original language and English, as Supporting Information.

Response: An interview guide has been uploaded as part of the supporting information.

3. Thank you for stating the following financial disclosure:

"Research reported in this publication was supported by the National Human Genome Research Institute of the National Institutes of Health under Award Number U01HG009822. The content is solely the responsibility of the authors and does not necessarily represent the official views of the National Institutes of Health."

Please state what role the funders took in the study. If the funders had no role, please state: ""The funders had no role in study design, data collection and analysis, decision to publish, or preparation of the manuscript.""

If this statement is not correct you must amend it as needed.

Please include this amended Role of Funder statement in your cover letter; we will change the online submission form on your behalf.

Response: This has been addressed as follows:

Research reported in this publication was supported by the National Human Genome Research Institute of the National Institutes of Health under Award Number U01HG009822. The content is solely the responsibility of the authors and does not necessarily represent the official views of the National Institutes of Health. The funders had no role in study design, data collection and analysis, decision to publish, or preparation of the manuscript.

4. Thank you for stating the following in the Funding Section of your manuscript:

“Research reported in this publication was supported by the National Human Genome Research Institute of the National Institutes of Health under Award Number U01HG009822. The content is solely the responsibility of the authors and does not necessarily represent the official views of the National Institutes of Health.”

We note that you have provided funding information that is not currently declared in your Funding Statement. However, funding information should not appear in the Acknowledgments section or other areas of your manuscript. We will only publish funding information present in the Funding Statement section of the online submission form.

Please remove any funding-related text from the manuscript and let us know how you would like to update your Funding Statement. Currently, your Funding Statement reads as follows:

“Research reported in this publication was supported by the National Human Genome Research Institute of the National Institutes of Health under Award Number U01HG009822. The content is solely the responsibility of the authors and does not necessarily represent the official views of the National Institutes of Health.”

Please include your amended statements within your cover letter; we will change the online submission form on your behalf.

Response: Thank you. The funding statement has been included in the cover letter.

5. We note that you have stated that you will provide repository information for your data at acceptance. Should your manuscript be accepted for publication, we will hold it until you provide the relevant accession numbers or DOIs necessary to access your data. If you wish to make changes to your Data Availability statement, please describe these changes in your cover letter and we will update your Data Availability statement to reflect the information you provide.

Response: There is no change in the Data Availability statement.

Reviewers' comments:

Comments to the Author

1. Is the manuscript technically sound, and do the data support the conclusions?

The manuscript must describe a technically sound piece of scientific research with data that supports the conclusions. Experiments must have been conducted rigorously, with appropriate controls, replication, and sample sizes. The conclusions must be drawn appropriately based on the data presented.

Reviewer #1: Yes

2. Has the statistical analysis been performed appropriately and rigorously?

Reviewer #1: I Don't Know

3. Have the authors made all data underlying the findings in their manuscript fully available?

The PLOS Data policy requires authors to make all data underlying the findings described in their manuscript fully available without restriction, with rare exception (please refer to the Data Availability Statement in the manuscript PDF file). The data should be provided as part of the manuscript or its supporting information, or deposited to a public repository. For example, in addition to summary statistics, the data points behind means, medians and variance measures should be available. If there are restrictions on publicly sharing data—e.g. participant privacy or use of data from a third party—those must be specified.

Reviewer #1: Yes

4. Is the manuscript presented in an intelligible fashion and written in standard English?

PLOS ONE does not copyedit accepted manuscripts, so the language in submitted articles must be clear, correct, and unambiguous. Any typographical or grammatical errors should be corrected at revision, so please note any specific errors here.

Reviewer #1: Yes

5. Review Comments to the Author

Please use the space provided to explain your answers to the questions above. You may also include additional comments for the author, including concerns about dual publication, research ethics, or publication ethics. (Please upload your review as an attachment if it exceeds 20,000 characters)

Reviewer #1: The authors present a qualitative discussion of the perceptions, opinions, and (limited) experiences of Ugandan researchers on the subject of returning incidental findings to participants in genetic and genomic research projects. Their methodology is sound, and their findings are clearly presented. They provide many interesting and important relevant references. They could improve by explaining in the discussion exactly what new information this study brings to that discussion. As well, some specific suggestions or questions below.

Response: Thank you for this guidance. We have improved on the discussion with addition of what our findings add to the debate on incidental findings.

Introduction: Might be worth mentioning that GGR has come leaps and bounds forward in the ten years

since these references on incidental findings were published, and that an updated discussion on this topic is therefore much needed.

Response: Thank you. The introduction has been improved.

They highlight the specific need for expanding these discussions in African populations, which is important. But I'm not sure they adequately synthesize at the end how their findings might be important specifically for African researchers and African populations.

Response: Information has been added in the manuscript at the end of the discussion section on how this study may be important to others on the continent.

The sentence at lines 115-117 (reference 17) brings up a very interesting and complicated topic (about patient and family confidentiality in the context of genetic testing). Perhaps just one or two more sentences expanding on what is meant by this debate would be helpful to add.

Response: The introduction has been revised with addition of more information towards the end of the section.

They mention several times the possibility of genomic analysis uncovering an infectious disease or something that would be a public health concern. Could the authors expand on what such situations they envision, giving specific examples?

Response: Information has been added on the genetic predisposition to some infectious diseases.

I am not sure about this sentence (at line 323-324): "unless it is a life-threatening event that one would error on the side of being wrong than right." Would the possibility of reporting a life-threatening event that turned out to be an error not be severely damaging? I would think all results should have a high degree of certainty before being reported, and I felt the excerpts they include at this section support this as well.

Response: The write-up has been clarified as one would need a high degree of certainty unless it's a life-threatening condition where early warning can be permitted.

Sincerely,

Dr. Joseph Ochieng
