# Supplementary material for: Perspectives and experiences of researchers regarding feedback of incidental genomic research findings: A qualitative study
Source: PLoS One. 2022 Aug 29;17(8):e0273657. doi: 10.1371/journal.pone.0273657 (PMC9423610; doi:10.1371/journal.pone.0273657)
Supplement: Attachment — Submitted filename: PONE-D-21-28187_reviewer.pdf [file pone.0273657.s004.pdf]

Comments for the author:

This is a manuscript addressing researchers' perspectives and experiences regarding feedback of incidental genomics findings to participants performing a qualitative study on 30 respondents selected based on their role as genetics and genomics researchers in Uganda. They highlighted the lack of ethical guidelines and uncertainty about the findings. This manuscript raises an interesting clinical concern, but there are concerns that need to be addressed precisely.

Major points

My main concern is lack of new findings compared to what has been published so far. The authors stated that they explore genetics and genomics researchers' perspectives and experiences regarding sharing incidental findings during the conduct of genetics and genomics research (GGR) in Uganda; however, most of their results are similar to previous published findings in the same setting. They concluded the importance of genetic consolers, ethical guidelines for conducting GGR with challenges including communicating incidental findings, proper evaluation of the risks and implications anticipated from sharing the results on the individual, family members and the entire community in addition to lack of clear ethical guidelines in having any systematic approach, procedures or standards. Additionally, they mentioned that there is not a comprehensive guideline for the determination of clinical validity and relevancy of incidental findings. However, the African Academy of Sciences (AAS) published a policy paper, which included recommendations for integration of genomic medicine research and service delivery (African Academy of Sciences. A framework for the implementation of genomic medicine for public health in Africa, open research, 2021). Moreover, in a publication by Matimba et al. (BMJ Glob Health. 2022, PMID: 35017180), they discussed comprehensively about decisions whether and what findings ought to be fed back. They stated that the decisions should be based on analytical and clinical validity of the results, their potential value and utility as well as participant volition. They defined a decision-making flow-chart along with recommended criteria to consider. Based on such comprehensive guidelines and previous publication, it is hard to make a conclusion about additional points of current manuscript.

Minor comment

There is not any discussion about cconsidering vulnerable populations such as children and fetuses.
