# Supplementary material for: Perspectives and experiences of researchers regarding feedback of incidental genomic research findings: A qualitative study
Source: PLoS One. 2022 Aug 29;17(8):e0273657. doi: 10.1371/journal.pone.0273657 (PMC9423610; doi:10.1371/journal.pone.0273657)
Supplement: Attachment — Submitted filename: Cover letter-PONE-D-21-28187_reviewer.pdf [file pone.0273657.s005.pdf]

Professor Emily Chenette

Editor-in-Chief

PLOS ONE Journal

Dear Professor Chenette, 2022-03-19

The current manuscript does not add any extra original findings to what is already in the published literature. Overall, it only confirms previous observations rather than suggest any original findings.

Yours sincerely,

Mojgan Yazdanpanah MD PhD
