# Supplementary material for: Perspectives and experiences of researchers regarding feedback of incidental genomic research findings: A qualitative study
Source: PLoS One. 2022 Aug 29;17(8):e0273657. doi: 10.1371/journal.pone.0273657 (PMC9423610; doi:10.1371/journal.pone.0273657)
Supplement: Attachment — Submitted filename: Point by point response to review comments May 28, 2022.docx [file pone.0273657.s006.docx]

**May 28, 2022**

**The Editor,**

**PLOS ONE**

**Dear Sir/Madam,**

**Ref: [PONE-D-21-28187] - [EMID:6867e1a975ba2bdf]. Perspectives and Experiences of Researchers Regarding Feedback of Incidental Genomic Research Findings: a qualitative study**

**Point by point response to review comments**

**Comment:**

We note that your Data Availability Statement reads: "A data set has been uploaded on the figshare website.

**Response:**

Following the upload of the data set in figshare, the DOI generated failed to link to the document.

Correspondence with the editorial team resulted in the following statement. "Thank you for reaching out to us regarding this concern. We note that you may need to activate the link and make it publicly available for the DOI to work. If that doesn't work, you may also upload your dataset as a Supporting Information file."

Following guidance of the editorial team, we have revised the manuscript to read **“Data sources have been uploaded as Supporting Information files”.**

Sincerely,

**Dr. Joseph Ochieng**
